# Linking niche size and phylogenetic signals to predict future soil microbial relative abundances

**DOI:** 10.3389/fmicb.2023.1097909

**Published:** 2023-08-14

**Authors:** Andrew Bissett, Steven D. Mamet, Eric G. Lamb, Steven D. Siciliano

**Affiliations:** ^1^CSIRO Oceans and Atmosphere, Hobart, TAS, Australia; ^2^University of Saskatchewan, Saskatoon, SK, Canada

**Keywords:** soil bacteria, microbial traits, phylogenetic signal, environmental change, structural equation model, soil

## Abstract

Bacteria provide ecosystem services (e.g., biogeochemical cycling) that regulate climate, purify water, and produce food and other commodities, yet their distribution and likely responses to change or intervention are difficult to predict. Using bacterial 16S rRNA gene surveys of 1,381 soil samples from the Biomes of Australian Soil Environment (BASE) dataset, we were able to model relative abundances of soil bacterial taxonomic groups and describe bacterial niche space and optima. Hold out sample validated hypothetical causal networks (structural equation models; SEM) were able to predict the relative abundances of bacterial taxa from environmental data and elucidate soil bacterial niche space. By using explanatory SEM properties as indicators of microbial traits, we successfully predicted soil bacterial response, and in turn potential ecosystem service response, to near-term expected changes in the Australian climate. The methods developed enable prediction of continental-scale changes in bacterial relative abundances, and demonstrate their utility in predicting changes in bacterial function and thereby ecosystem services. These capabilities will be strengthened in the future with growing genome-level data.

## Introduction

Soil bacteria provide ecosystem services that regulate climate, purify water, and produce food and other commodities. However, despite their central role in regulating terrestrial carbon dynamics, nutrient cycles, and plant productivity, much remains unknown about even general bacterial traits, including distributions, habitat preferences, and life histories (Fierer and Jackson, 2006; Fierer, 2017; Delgado-Baquerizo et al., 2018). This deficiency limits opportunities to incorporate trait parameters into ecosystem and process models designed to predict responses to climate and land-use changes (Coles et al., 2017).

Some progress is, however, being made. Investigators have used drivers of bacterial community assembly and turnover (e.g., Hanson et al., 2012; Stegen et al., 2012; Nemergut et al., 2013) to model how preferred niche space (the ecological space occupied by an organism) connects to bacterial spatial dispersal (Philippot et al., 2009; Koeppel and Wu, 2012; Barberán et al., 2014). The link between niche and distribution arises from the interplay between organisms shared evolutionary history and the resulting correlations in functional traits (Losos, 2008; Barberán et al., 2014). Thus, exploring the processes that give rise to niche phylogenetic conservation may enable prediction of bacterial spatial distribution and ecosystem functioning from response traits of ecosystem members (for an example, see Barberán et al., 2014).

If niche preferences are phylogenetically conserved, they may interact with abiotic and biotic filtering mechanisms to result in patterns of phylogenetically clustered spatial distributions (Goberna et al., 2014). Koeppel and Wu (2012), using categorical habitat definitions, suggest four possible hierarchical patterns of habitat association, where “parent” and “subtaxa” refer to the relative taxonomic level, e.g., “phylum” is the parent of the subtaxa “class” and “habitat association” refers to an affinity for a given habitat: (1) habitat association maintained among subtaxa, (2) parent taxa exhibit habitat association, but subtaxa do not, (3) habitat association not shared among subtaxa, or (4) parent has no habitat association, but subtaxa do. Simple continuous traits (e.g., activity at different temperatures) often exhibit weak phylogenetic signals, while binary (e.g., presence or absence of a metabolic pathway) and complex traits (e.g., abiotic stress or competition related) often demonstrate strong signals (Goberna and Verdú, 2015). Weak phylogenetic signals arising due to random events in the shallow phylogenetic tree may be less useful in predicting microbial distributions than traits conserved in deep divisions (Lu et al., 2016). For example, complex traits linked to abiotic filtering, such as pH optima, are likely conserved deep in the phylogenetic tree (Goberna and Verdú, 2015; Keck et al., 2016b). Indeed, recent work has identified effects of abiotic factors, such as pH, on phylogenetic clustering, with neutral soils leading to phylogenetic dispersion (Tripathi et al., 2018).

Conserved responses to pH, carbon, moisture, and temperature shape diversity (Siciliano et al., 2014), functional gene distribution (Nelson et al., 2016), and enzyme activity (Waldrop et al., 2017) in soil microbial communities. In turn, climate and plant communities affect these soil parameters and combined climate, plant, and soil properties explain significant components of soil microbial composition (Tedersoo et al., 2014; Maestre et al., 2015; Slessarev et al., 2016; Waldrop et al., 2017). These relationships allow investigators to model how bacteria respond to plants (Piper et al., 2015; Mamet et al., 2017), grazing (Philippot et al., 2009), and aridity changes (Delgado-Baquerizo et al., 2017). Researchers commonly model alpha and/or beta diversity when investigating drivers of microbial community structure, rather than taxonomic unit relative abundances, because a single descriptor is more statistically tractable. While redundancy analysis and correspondence analyses have been used to model community level responses, they have not been widely applied to individual taxa. Much information is lost, however, when the multitude of species present in a soil sample are reduced to a single number. Novel multivariate (Mamet et al., 2017) and geostatistical (Philippot et al., 2009) techniques can identify bacteria from complex communities for which specific models need to be created, however a broad-scale approach to modeling the relative abundances of soil bacteria is yet to be developed.

Given, (1) the clear importance of environmental parameters in explaining microbial diversity, (2) the phylogenetic signal associated with many microbial traits, and (3) the idea that selection generates an adaptive fit of organisms to their environment, we postulated that bacterial relative abundances can be modeled using climate, plant, and soil factors derived from structural equation models (SEM) as indicators of response traits (as defined by Suding et al., 2008 for plants, as the traits that allow organisms to respond to their environment). The community that will inhabit an environment, results from sorting processes among individuals with appropriate response traits. We, therefore, expect that these SEM derived links between bacterial relative abundance and environmental properties would be indicative of soil bacterial traits and niche preference, and path coefficients for these links could, therefore, be treated as indicators of bacterial traits. The rationale for using an SEM-based approach is that it expressly addresses collinearity within a network of causal factors (see Grace, 2006) and outlines biologically plausible postulated causal links (unlike unstructured machine learning approaches which typically evaluate all possible links), allowing SEM to test mechanisms (for example Grace et al., 2016). Further, SEM can predict responses to diffuse global and local changes (*sensu*; Mason et al., 2014; Piper et al., 2015), which would allow policy makers to better understand how environmental changes may result in long term changes to bacterial communities and ecosystem services provision.

Bacterial relative abundance does not necessarily predict ecosystem service provision, due to historical contingencies (Evans and Wallenstein, 2014; Hawkes and Keitt, 2015), substrate availability, temperature, and competition. However, climate, plant, and soil parameters have successfully predicted enzyme activity in soil using microbial composition and SEM (Waldrop et al., 2017). Using genomic models of bacterial functional prediction (e.g., PICRUST2; Langille et al., 2013) bacterial relative abundances can be linked to broad ecosystem functions (Eng and Borenstein, 2018; Mushinski et al., 2018). Here, we modeled bacterial relative abundances, across 1,381 soil samples from the Biomes of Australian Soil Environment (BASE) dataset (Bissett et al., 2016) to describe bacterial niche space and the determinants of bacterial community structure in Australian soils. We hypothesized that: (i) postulated causal networks could predict the relative abundance of bacterial taxa from environmental data at large scales, (ii) these networks would be indicative of the niche space inhabited by the bacteria modeled, and (iii) indicators of certain ecological response traits would display a phylogenetic signal. Further, to demonstrate the promise of these networks, we linked them to climate change models to predict how soil bacteria, and in turn potential microbial function, may respond to short-term expected changes in the Australian climate.

## Methods

### Sample collection/data acquisition

#### Study site and bacterial diversity data

We used bacterial 16S rRNA gene amplicon data from 1,381 samples contained in the publicly available Biomes of Australian Soil Environment (BASE) microbial database (Bissett et al., 2016) to describe microbial niche space and determinants of bacterial distribution in Australian soils (Supplementary Table S1; Supplementary Figure S1). Full sampling and data generation methods are described in detail elsewhere (Bissett et al., 2016). Briefly, soils were collected as 9 cm × 10 cm deeps cores, across a 25 m × 25 m, and pooled to a single sample per site. Sites sampled covered 27 IBRA 7 regions (Interim Biogeographic Regionalisation for Australia; https://www.Environment.Gov.Au/land/nrs/science/ibra#ibra) and many land-use categories, representing most key vegetation types (Native restoration sites and production landscapes, including orchards and cereal croplands). Approximately 50% of samples came from conservation reserves. Urban landscapes were not sampled. Genomic DNA was extracted in triplicate from each sample using Mobio Power Soil DNA extraction kits according to manufacturer’s instructions. Bacterial 16S rRNA gene (27F—519R; Lane et al., 1985; Lane, 1991) amplicons were sequenced using Illumina MiSeq (300 bp PE) and 97% sequence similarity operational taxonomic unit (OTU) tables generated using open OTU picking in the UPARSE software (Edgar, 2013). Representative sequences were classified using the RDP classifier (Wang et al., 2007) and the Green Genes database (13_5), after which chloroplast and mitochondrial sequences were removed. The resultant OTU abundance table was rarefied at 22,500 reads per sample, to standardize per sample sampling effort, and corrected for 16S rRNA gene copy number using copyrighter (Angly et al., 2014). All sequence data are available from the BASE data portal^1^ and from the Sequence Read Archive under NCBI bioproject ID PRJNA317932. R code to run the analyses below is available from the authors on request.

#### Environmental and physicochemical analyses

Edaphic data [soil pH, Organic Carbon, Ammonium, Nitrate, Phosphorus, Potassium, Sulfur, trace elements (Cu, Fe, Mn, and Zn), exchangeable cations (Mg, K, Na, and Ca), conductivity, and soil particle size] were collected as detailed in Bissett et al. (2016).

Present and future (2030 projections) climate data (mean annual temperature, total annual precipitation, and mean humidity) and plant productivity data (C3 megatherm and mesotherm to describe C3 photsynthetic plants and C4 macrotherm to describe C4 plants, NDVI) were downloaded from climate spatial layers hosted by the Atlas of Living Australia’s spatial portal.^2^ The variation over these data captured by sample locations is shown in Supplementary Figure S64.

### Structural equation modeling

We used SEM (Grace, 2006) to investigate a range of potential mechanisms underlying climate-vegetation-soil-microbial community relationships. Whereas a number of multivariate methods are largely descriptive and more appropriate for exploratory analyses, SEM is able to test a network of causal hypotheses and is recommended for evaluation of multivariate hypotheses (Grace, 2006; Grace et al., 2012). Specifically, we used SEM because it allows the evaluation of simultaneous influences (for instance, humidity may influence bacterial relative abundance both directly and through altering vegetation characteristics) rather than individual causes (for example, humidity influences bacterial relative abundance only directly). The method is thus appropriate for establishing probable causality at the system (for example, climate-vegetation-soil-bacteria), rather than the individual level (for example, climate–bacteria). SEM relies on researcher specification of a network of *a priori* causal assumptions based on a scientific body of evidence (for example, pH is an important driver of bacterial richness and not vice versa), and then testing whether that causal network is consistent with empirical data (Grace, 2006; Lefcheck, 2016; Shipley, 2016). The postulated causal network gives rise to a series of linear equations, which in turn give rise to a modeled covariance matrix. The modeled covariance matrix is then compared with the observed covariance matrix arising from the data. A statistically acceptable congruence between the modeled (causal model implied) and observed covariance matrices is thus an empirical validation of the causal assumptions used. In other words, an SEM is an ecological theory describing a particular system, and if congruent with the data, the theory is supported. SEM is a well-established tool widely used in the sciences for testing causal inferences with correlative data sets; however, it is critically important that the causal assumptions made by the researchers be well-grounded in prior work, scientific knowledge, logical arguments, and/or other evidence. An SEM model that fits the data does not prove the causal assumptions used, but replication of a given model across many systems represents a very strong test of the underlying theory (Grace et al., 2012).

We built on the SEM (Grace, 2006) outlined in Siciliano et al. (2014), which utilized edaphic, climate, and spatial variables to explain soil bacterial diversity and community structure. To compare the relative strength of relationships within the SEM, we used standardized path coefficients. Unstandardized path coefficients reflect the expected (linear) change in the response with each unit change in the predictor, though are influenced by the unit of measurement of each variable which precludes direct comparison of coefficients within the model (Grace, 2006). In contrast, standardized path coefficients are expressed in equivalent units, regardless of the original measurements, so may be used to make inferences about the relative strength of relationships (Grace et al., 2018). For example, two paths with a standardized coefficient of 0.6 are equivalent in terms of relative influence on the mean of the response. Herein, we limited exogenous variables to those available in future climate predictions: three edaphic variables (pH, conductivity, and organic matter); three climatic variables [maximum annual temperature (MAT), average humidity, and average precipitation]; and three vegetation variables (C3 macrothermal, C3 mesothermal, and C4 megathermal plant coverages—see descriptions of these terms above). Note that organic carbon (OC) was dependent on conductivity, pH, humidity, precipitation, and C3 macrothermal plant abundance. Our decision to use an observed variable model, rather than a latent variable model (as per Siciliano et al., 2014), was based on the need to provide a transparent link between bacterial relative abundances and predicted changes in exogenous variables. Latent variable in SEM parlance refers to an unobserved variable that cannot be measured directly (e.g., intelligence), though an observed variable hypothesized to represent the unobserved (e.g., SAT scores) may be used in its place. In other words, we wanted to use tangible variables readily measurable in the real world that may link biotic and abiotic variables with bacterial relative abundance. Thus, when future climatic scenarios become available, the expected changes in bacterial relative abundances can be readily recalculated. Variances of select variables were adjusted by taking the log_10_ of conductivity, OC, humidity, precipitation, and C3 vegetation coverage to meet the linearity requirements of SEM.

We used several common goodness-of-fit measures to evaluate how well the SEM-estimated variance–covariance matrix matched the observed variance–covariance matrix (Grace and Keeley, 2006). The *Χ*^2^ statistic from the SEM follows the *Χ*^2^-distribution and may be used to derive a confidence level in the SEM fit. Contrary to typical assessment of the *Χ*^2^ statistic, in SEM-parlance, failing to reject the null hypothesis that the *Χ*^2^ is different from 0 (i.e., perfect model fit) implies a generally good representation of the data (*p* > 0.05). Therefore, larger *Χ*^2^ statistics indicate a rejection of the null hypothesis (i.e., a large discrepancy between the observed and modeled variance–covariance matrices; *p* < 0.05). *Χ*^2^ is affected by sample size. With larger sample sizes more likely to generate poor fit due to small absolute deviations. Therefore, *Χ*^2^ should be interpreted cautiously and with several other fit indices that attempt to correct for sample-size biases. For instance, the Comparative fit index (CFI) considers the deviation from the ‘null’ model and typically the null model estimates all variances but sets the covariance to zero. CFIs closer to one are considered a good model fit. The root-mean squared error of approximation (RMSEA) statistic penalizes models based on sample size (i.e., parsimony-adjusted). The standardized root mean square residual (SRMR) represents the difference between the residuals of the sample covariance matrix and the hypothesized model. SRMR and RMSEA values closer to zero represent good model fit.

Bacterial relative abundances were calculated at all levels of biological organization, however only class and phylum level relative abundances are discussed. SEMs were calculated for 57 phyla (CFI = 0.98, *R*^2^ = 0.24), 200 classes (0.98, 0.23), 416 orders (0.98, 0.21), 676 families (0.98, 0.20), and 1,937 genera (0.98, 0.14) using MPlus version 8.3 (Muthén and Muthén, 2017). We evaluated the predictive accuracy of SEMs using a random sub-sampling of 1,000 samples and then predicting bacterial relative abundance for the remaining 381 samples. Predictions were made using “fitted_lavaan” v. 0.6-11 in R (Rosseel, 2011; Siciliano et al., 2014) and compared to observed bacterial relative abundances.

To evaluate bacterial phyla abundance responses along soil ecological gradients, we compared clustered SEM path coefficients to Huisman–Olff–Fresco (HOF) hierarchical regression models (Huisman et al., 1993). Standardized path coefficients of bacterial phyla links to climate, vegetation, and soil were hierarchically clustered into 10 groups based on Ward’s minimum variance method (Borcard et al., 2011). The optimal number of groups (10) was assessed on average silhouette width and misclassification. Extended eHOF models were used to calculate the niche space ranges and optima, where niche optimum was present for soil parameters.

It should be noted that, as samples are typically separated at greater than km scale, our models assume limited geospatial dependency between samples. Structural Equation Models, by their very nature, assume deterministic processes dominate and are thus ill suited to stochastic modeling. While we acknowledge that stochastic processes are likely often important at some level in microbial community assembly, failure to incorporate them explicitly in the model does not make the model invalid, it merely constrains our ability to assign causation to stochastic process should the deterministic processes modeled not adequately explain the system under investigation.

### Huisman–Olff–Fresco models

Huisman–Olff–Fresco hierarchical regression models (Huisman et al., 1993), as applied in the R package “eHOF” v.1.12 (Jansen et al., 2013), were used to calculate niche optima, maximum, and minimum for each exogenous environmental variable linked to bacterial relative abundance. The best HOF model was identified with Akaike Information Criterion and boot strapping (*n* = 5) with a minimum occurrence value of 10 in a sample used to determine which samples were used in the regression model. Only those models showing an optimum, (Type I model is flat with no optimum) were analyzed further. Further, every model was visually inspected and those HOF models with two niche optima (i.e., Types VI, and VII), at the range edges (e.g., pH’s 4 or 10) were interpreted as indicating no optima. Niche edges were interpreted as the inflection point of the fitted curve.

### Phylogenetic signal

It has been observed that traits of closely related organisms are often similar, particularly when these traits are under environmental selection pressure. The tendency for related organisms to display similar trait values as a consequence of their phylogenetic proximity is referred to as “phylogenetic signal” (Keck et al., 2016a). To visualize statistically significant conservation of edaphic traits (response to pH and conductivity) at the genera level, we calculated Local Indicators of Phylogenetic Association (LIPA) using the package “phylosignal” v.1.2 (Keck et al., 2016a). Only genera available in the Greengenes database (*n* = 1,431) and genera that occurred in more than 200 soil samples (*n* = 303, mean relative abundance of 0.0003 (>10,000 reads across all samples), and mean frequency of 465 samples out of 1,381) were analyzed. Phylogenetic trees were constructed by aligning sequences to the SILVA alignment (Pruesse et al., 2007; v128) using the SINA aligner (Pruesse et al., 2012) followed by manual curation. The phylogeny was then constructed by adding the short sequences to the SILVA tree using the ARB (Ludwig et al., 2004) maximum parsimony method, resulting in a phylogeny congruent with the SILVA full 16S rRNA gene based phylogeny. LIPA is derived from a class of statistical tools used to analyze local spatial patterns called Local Indicators of Spatial Association (LISA). Within LISA, “Moran’s I” (Anselin, 1995) and Abouheif’s C mean (Abouheif, 1999) are used to detect hotspots of positive and negative correlation. This same statistic, when applied to phylogenetic data (as LIPA), detects species with similar and different neighbors. We plotted traits and LIPA where *p* < 0.05, along with phylogeny.

### Bacterial response to change

Input data used to impute responses to predicted change comprised the data describing current soil bacterial communities and predicted microbial relative abundances for future climate scenarios. Current data were prepared from the normalized OTU table described above. Microbial relative abundances under future climate, were predicted with coefficients of the climate-sensitive SEM (above) yielding predicted OTU relative abundances based on projected values of vegetation, climate, and soil. To estimate the change in relative abundance between present and future Cyanobacteria and Firmicutes distributions, we calculated ln-fold changes (“gtools” v.3.5.0 in R). To avoid infinite changes due to relative abundance values below detection limits (i.e., zero), we replaced zero values with half the lowest current relative abundance of Cyanobacteria, class Synechococcophycideae, and Firmicutes, class Bacilli (0.5*0.066 = 0.033). After calculating ln-fold changes, we used ANUDEM interpolation to create spatial maps to visualize these changes (Hutchinson, 1989). ANUDEM takes irregular point data and creates square-grid surfaces at user-defined resolutions (0.2° here).

To visualize the predicted community level changes of all taxa, we used Principal Coordinate Analysis (PCoA) on Bray-Curtis dissimilarities (BC) within the combined present and future microbial relative abundance matrix. PCoA produces orthogonal axes whose importance is measured by eigenvalues, and since it is based on a dissimilarity matrix (Borcard et al., 2011), here it represents relationships among current and future microbial relative abundances. We calculated the difference between the first and second axes for current and future microbial distributions from the PCoA matrix, and used these data to determine the change in n-dimensional space between time-periods. We visualized changes microbial composition using the interpolation method described above. BC was computed using “vegan” v.2.4–2 (Oksanen et al., 2016) and PCoA using “ape” v.4.1 (Paradis et al., 2004).

Lastly, we investigated how community composition may change soil functional potential under the projected climate changes. We used imputed metagenomic analysis to predict KEGG Ortholog (KO) functional profiles using PICRUST (Langille et al., 2013). We then used these profiles to estimate relative abundance of CH_4_ cycling genes. For each (current and predicted) OTU matrix, OTU’s were given Greengenes (ver. 13.5) identifications as per PICRUST documentation. OTU’s without a Greengenes’ match were omitted (approx. 15% of OTUs). We acknowledge that functional assignment is only as good as the database and annotation available at the time of analysis and that future efforts will likely improve the reliability of modeled data. Functional prediction proceeded on the remaining OTUs according to the PICRUST documentation. Once we obtained current and future gene relative abundances, we performed a PCoA analysis on BC within the combined present and future metagenomics matrices. Changes in microbial composition and specific gene relative abundances were visualized as described above.

## Results

Our postulated causal network, from 1,381 soils taken across the breadth of the Australian continent, was congruent with observed data (CFI ≈ 0.99, RMSEA ≈ 0.05 X, *R*^2^ ≈ 0.3, Figure 1A; Supplementary Figures S4–S60). Networks from 1,000 random samples successfully predicted bacterial relative abundance in the 381 hold-out samples (Figure 1B). Using this framework, we interpolated the relative abundance of Proteobacteria across Australia (Figure 1C). In general, the strongest drivers of soil Proteobacteria relative abundance were vegetation type, with C4 Megathermal plants and C3 Macrothermal plants having nearly equal, but opposite, effects on Proteobacteria relative abundance. Climate variables were all equally linked to Proteobacteria relative abundance, as were soil variables (Figure 1A). Other than C4 Megathermal plants, only conductivity was negatively linked to Proteobacteria relative abundance.

**Figure 1:**
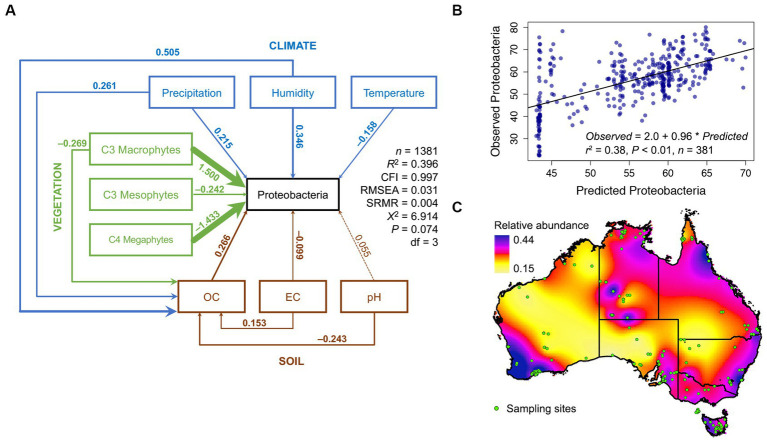
Structural equation model of Proteobacteria relative abundance across Australia. A SEM with nine endogenous and one exogenous variable was constructed and found congruent with the observations from 1,381 soil samples from across Australia. **(A)** The SEM model for the phylum Proteobacteria, see Supplementary Figures S1–S56 for SEMS of other phyla. Solid arrows represent significant relationships (*p* < 0.05, dashed lines are non-significant) and the thickness of the arrow indicates the strength of the relationship, and the color indicates the origin of the path (blue = climate, green = vegetation, and brown = soil). Standardized path coefficients are shown next to each path. Standardized path coefficients can be interpreted as follows: if, for example, temperature increases by one standard deviation from the mean, then Proteobacterial relative abundance would increase by 0.25 standard deviations from its own mean. **(B)** Evaluation of SEM predictive ability, using a SEM calibrated from 1,000 samples to predict Proteobacterial relative abundance across 381 not in the calibration data set, **(C)** Spatial dispersal of Australian Proteobacterial relative abundance in 2016.

**Figure 2:**
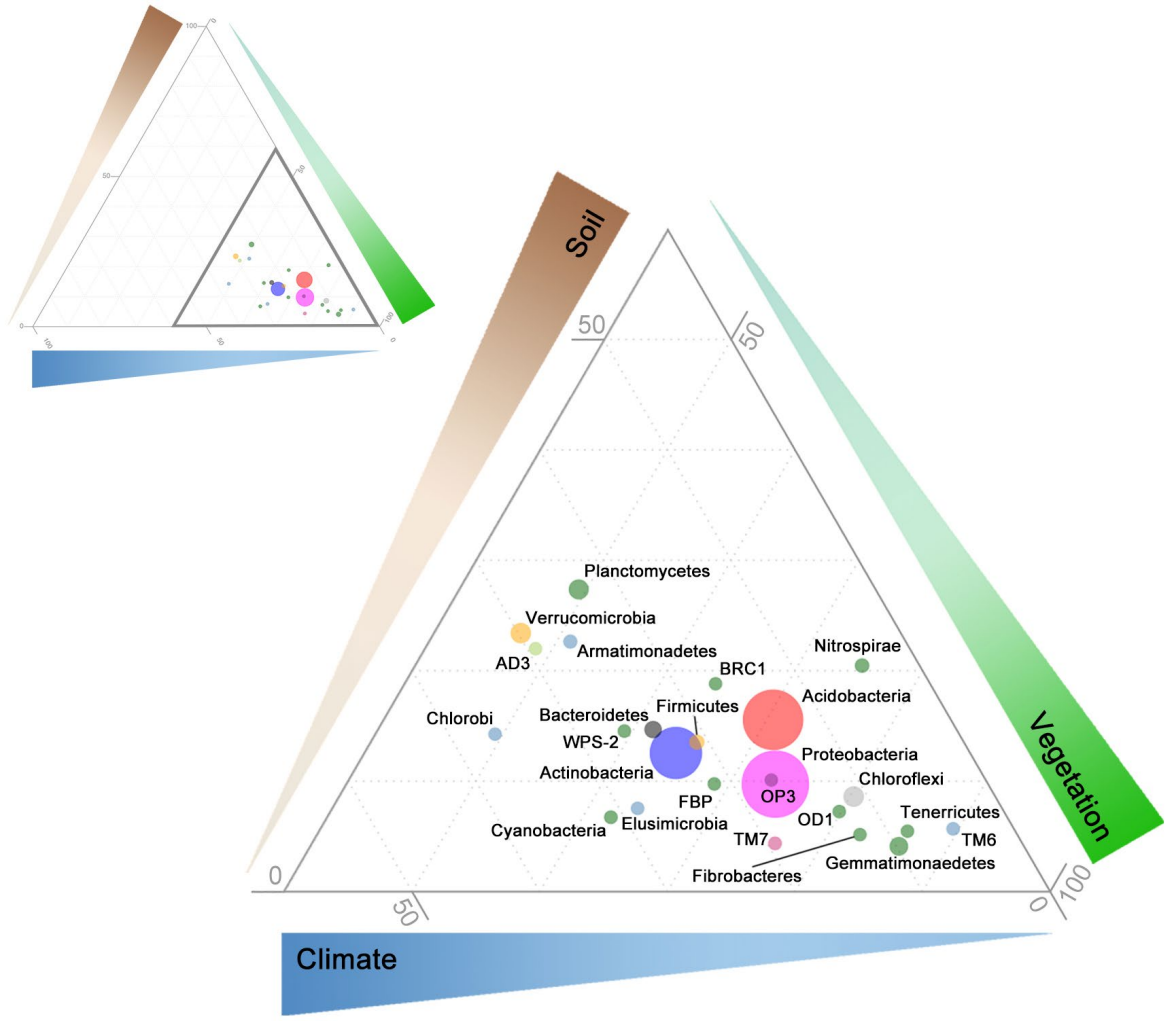
Ternary diagram of relative influence of soil, vegetation, and climate on bacterial relative abundance at the phyla level. Path coefficients were calculated between each phyla and endogenous variables: soil properties (brown; OC, conductivity, and pH), vegetation characteristics (green; C3 macrothermal plants, C3 mesothermal plants, and C4 megathermal plants), and climate (blue; maximum annual temperature, humidity, and precipitation). Relative influence of soil, vegetation, and climate is indicated by the thickness and contrast of the triangles along each axis (darker, thicker = greater influence; range from 0 to 100). Upper left: full ternary diagram with thick, dark triangle highlighting the region enlarged in the lower right. Point positions represent absolute values of standardized direct-path SEM coefficients scaled to sum to 100. For example, the Acidobacteria soil path coefficients were: OC = 0.051, EC = −0.358, pH = −0.165, and the sum of all 9 was 3.689. Therefore: ∑[(0.051/3.689)*100 + (0.358/3.689)*100 + (0.165/3.689)*100] = 15.6. Doing the same for vegetation and climate yields 70.5 and 13.9, respectively, the sum of all three equates to 100—15.6 + 70.5 + 13.9—which equates to the ternary coordinates for Acidobacteria. Point sizes represent median relative abundance. Only phyla with a median relative abundance greater or equal to 1 across 1,381 samples are included here. Point colors correspond to the 10 groups determined through hierarchical clustering (see Figure 3).

Structural equation models fits (Supplementary Figures S4–S60) were robust across many bacterial phyla, with CFIs that ranged between 0.972 and 0.996, Root Mean Square Error of Approximations (RMSEA) that remained at 0.050, and Standardized Root Mean Square Residuals (SRMR) at 0.002. The effects of soil, vegetation, and climate drivers varied across phyla (Figure 2), with coefficients of variation ranging from 400% for the link between MAT and phylum relative abundance, to 1,100% for the link between conductivity and phylum relative abundance (Table 1). Most phyla did not display a strong response to soil conductivity. Only Acidobacteria (standardized path coefficient of −0.33; SE = 0.02) and Verrucomicrobia (−0.15; SE = −0.01) displayed a strong negative response, while only Bacteroidetes (0.27; SE = 0.03), displayed a positive response. Acidobacteria preferred acidic soils (−0.26; SE = 0.04), but indices of vegetation composition were also large drivers of Acidobacteria relative abundance. Chloroflexi also preferred acidic soils, while the common phyla Actinobacteria and Gemmatimonaedetes preferred alkaline soils. Unlike Acidobacteria, Actinobacteria did not display strong links to vegetation and instead preferred low precipitation. The Actinobacteria link to precipitation was of an equal, but opposite, magnitude to that observed for Proteobacteria. Firmicutes also showed strong negative links to both precipitation and OC. In general, phyla were linked to either climate and a soil factor, or climate and a vegetation factor.

**Table 1:** Phylum characteristics.

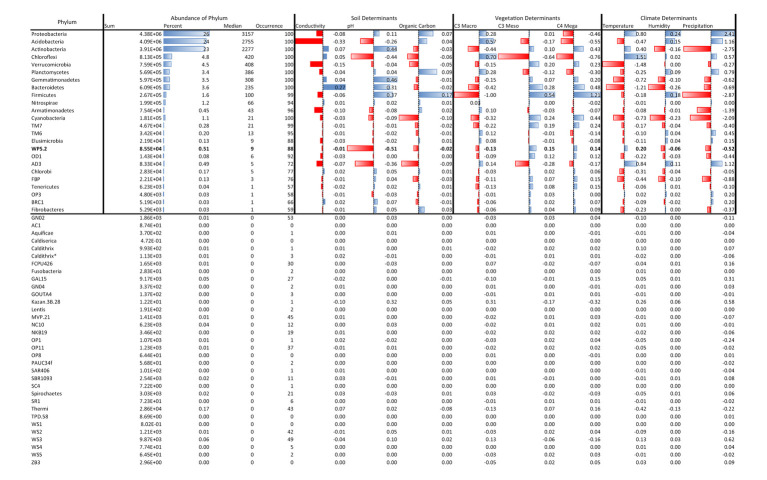

A consistent group of bacteria were highly responsive to climatic, compared to vegetation parameters (Supplementary Table S2). For example, seven bacterial classes that were consistently in the top 15 of 200, were responsive to MAT, humidity, and precipitation (Phylum in parentheses): Chloracidobacteria (Acidobacteria), Actinobacteria (Acidobacteria), Chthonomonadetes (Armatimonadetes), Gitt.GS.136 (Chloroflexi), Nitrospira (Nitrospirae), Betaproteobacteria (Proteobacteria), and Deltaproteobacteria (Proteobacteria). However, we would note that in general the importance of direct links on bacterial relative abundance descended from vegetation to climate to soil.

**Figure 3:**
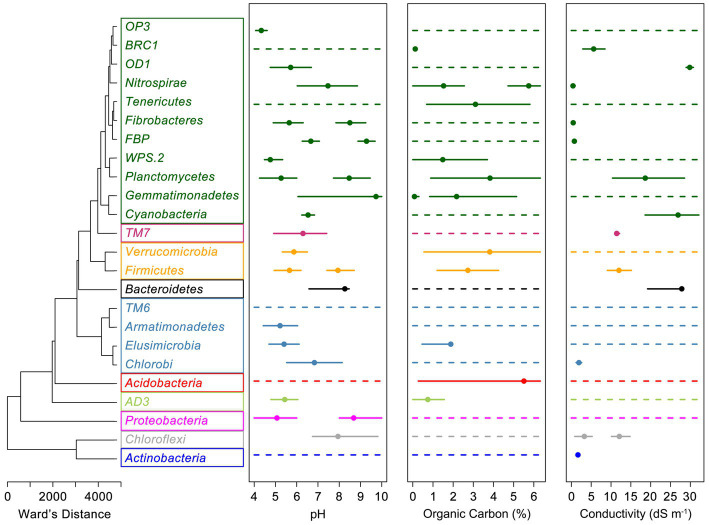
Clustering of soil, vegetation and climate drivers of bacterial relative abundances and links to soil niche space of bacterial genera. Standardized path coefficients of bacterial links to temperature, humidity, precipitation, C3 mesothermal plants, C3 macrothermal plants, C4 megathermal plants, OC, conductivity, and pH were associated based on Euclidean distance and then hierarchically clustered into 10 groups (represented by colored boxes) based on Ward’s minimum variance method. Extended Huisman-Olff-Fresco models were used to calculate the niche space ranges (solid lines), niche optimum (closed circles) where niche optimum was present for soil parameters. A continuous dashed line indicates a phylum for which a niche optimum was not detected.

Clustering of phyla based on all nine direct links between climate, vegetation, and soil variables modeled using SEM formed 10 groups based on their combined environmental traits (Figure 3). SEM derived associations between environmental variables and relative abundances were often congruent with eHOF calculated niche widths and optima. For example, Bacteroidetes showed positive associations with soil conductivity, pH and a weak negative association with soil OC (Table 1), all of which are reflected in modeled niche space (Figure 3). This was not always the case, however, and often no optima could be determined. In such cases, the relationship between the variable and microbial relative abundance was either linear (increasing or decreasing) or no relationship was apparent (for example, Supplementary Figures S2, S3). Similar results were observed for eHOF models of climate drivers (Supplementary Figure S4).

Highly abundant taxa and phylogenetically broad taxa, such as Acidobacteria, Actinobacteria, Proteobacteria, and Verrucomicrobia often exhibited wide niche spaces encompassing the majority of the range of normal soils, though within those ranges clear optima were frequently evident. Occasionally, despite being present in the majority of soils, phyla exhibited very defined niches for specific edaphic factors, for example Actinobacteria and conductivity (Figure 3; Supplementary Figure S3). Niche space was congruent down taxonomic ranks to varying degrees. Bacterial classes within a phylum with a defined niche space were consistent with the phylum, whereas classes within a phylum without a defined niche space, variably exhibited a defined niche space. For example, the phylum Actinobacteria had a niche optimum for salinity of “Very Saline” (1.6 dS m^−1^). Actinobacterial sub-taxa (classes) all showed defined salinity niche optima varying from the Highly Saline (3.5 dS m^−1^, Acidimicrobia and *A. OPB41*) to the Slightly Saline (0.5 dS m^−1^ for *A. actinobacteria*). In contrast, the phylum Verrucomicrobia had no defined salinity optimum nor did five of the six sub-taxa classes, with only Opitutae showing an optimum of “Moderately Saline” (0.98 dS m^−1^). Similar, classes in Verrucomicrobia that did not have a clearly defined niche for a particular environmental variable did not have a niche at the sub-taxa order level, and ones that did, i.e., Opitutae, continued to have niche consistency at this sub-taxa level. For example, Methylacidiphilae did not have a niche optimum for salinity nor did the four orders contained within this class. In contrast, all of the orders within Opitutae exhibited defined saline niches, except Opitutae HA64 which was too rare to reliably estimate a niche optimum.

**Figure 4:**
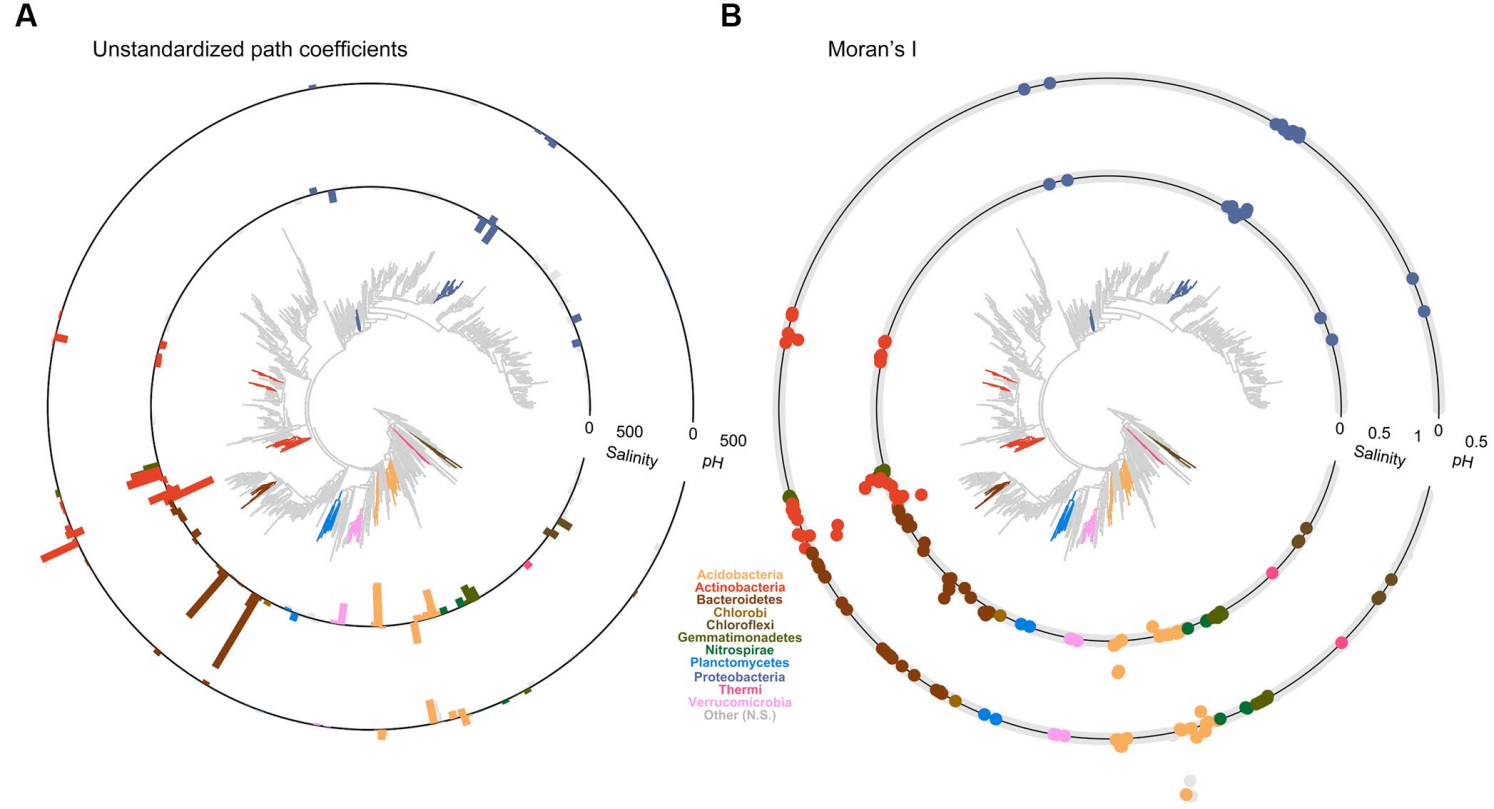
Microbial functional traits related to soil salinity and pH are more commonly shared among related taxa. **(A)** Standardized path coefficients (bars) between salinity and pH of the SEM model presented in Figure 1 applied to genus level relative abundances that displayed significant local areas of phylogenetic association are displayed in color, gray indicates no significant phylogenetic associations. Branch tips with no associated bar were present in less than 200 samples and were therefore excluded. **(B)** Local areas of phylogenetic conservation or dispersion significantly different from zero for all genera present in more than 200 samples (*p* < 0.05). Points indicate local Moran’s I for each genus for salinity (inner ring) or pH (outer-ring). Points on the ring = 0, points outside that vary in value (>0–1) and are indicated approximately by the scale indicators for salinity and pH. Bars, points, and *de novo* trees are color-coded according to phylum.

Structural equation model coefficients had global phylogenetic signals (*p* < 0.01) for soil (conductivity and pH) and climate (MAT) with Abouheif’s C_mean_ of 0.18 for conductivity, 0.11 for pH, and 0.06 for MAT (Supplementary Table S3). Vegetation drivers had weak and non-significant phylogenetic signals, as did the other climate drivers. Two key soil variables, conductivity and pH, differed greatly among genera and showed strong local areas of phylogenetic conservation or divergence (Figure 4). Phyla such as Acidobacteria and Actinobacteria had areas of conservation and divergence within their subtaxa. Salinity responses within 21 different Actinobacteria genera were locally phylogenetically conserved, although the nature of the response depended on the class, with Actinobacteria displaying a phylogenetically conserved (LIPA = 0.02) negative response to salinity (path = −52, stdev = 21). In comparison, Actinobacteria and Acidimicrobiia displayed a phylogenetically conserved (LIPA = 0.21) positive response to salinity (path = 300, stdev = 230).

**Figure 5:**
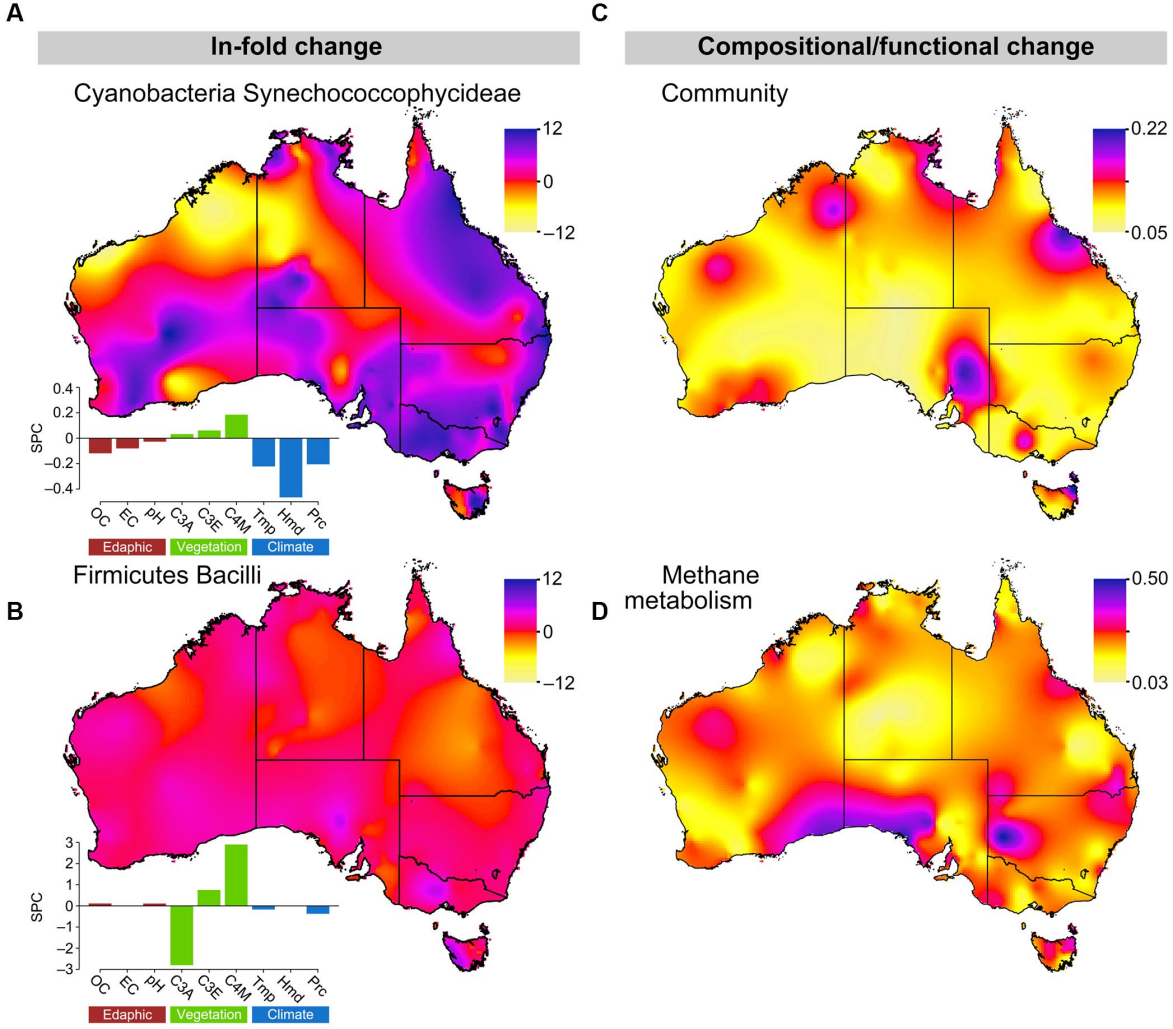
Changes in key microbial community composition and ecological function across Australia from 2016 to 2030. **(A,B)** ln-fold changes in Cyanobacteria Synechococcophycideae and Firmicutes Bacilli relative abundance. Change in n-dimensional space of PCoAs Bray–Curtis dissimilarity matrices of current and future **(C)** microbial composition and **(D)** methane metabolism. Color indicates the magnitude of change between time periods: **(A,B)** pink/violet = increased and yellow/orange = decreased future relative abundances; **(C,D)** pink/violet indicates relatively large changes and yellow/orange relatively small changes in community composition/methane metabolism. Standardized path coefficients from SEMs of soil factors, vegetation, and climate on bacterial relative abundance are presented for Cyanobacteria Synechococcophycideae and Firmicutes Bacilli. Soil influences (brown): OC, soil OC; EC, electrical conductivity. Vegetation influences (green): C3A, C3 macrothermal plants, C3E, C3 mesothermal plants; C4M, C4 megathermal plants. Climatic influences (blue): Tmp, temperature; Hmd, humidity; and Prc, precipitation.

Using 2030 climate prediction scenarios for the sample locations, we predicted significant climate change driven shifts in microbial community structure and function, likely driven by the responses of a few key microbial ecotypes (Figure 5). For example, we predict considerable changes in Synechococcophycideae relative abundance patterns across Australian soils (Poisson regression: *β* = 2.98, *F*_1,1807_ = 117.74, *p* < 0.001; Figure 5A). Synechococcophycideae relative abundance increases are projected to be greatest in Queensland, New South Wales, Victoria, South Australia, and eastern Tasmania, whereas Western Australia and the Northern Territory will experience a mix of increases and decreases. These changes are largely driven by changes in the climate related factors humidity, temperature and precipitation. Photosynthetic cyanobacteria from soils have been shown to prefer arid, low organic matter conditions (Cano-Díaz et al., 2019). These conditions are expected to increase in the regions of increased predicted Synechococcophycideae relative abundance in our models. Relative to Synechococcophycideae, Bacilli relative abundances show less change under 2030 climate predictions (*β* = 1.29, *F*_1,2057_ = 307.80, *p* < 0.001; Figure 5B). Given Bacilli are spore forming organisms and able to resist unfavorable conditions, it was expected that changes in their relative abundance would be less evident over the short time frame of our future predictions. Indeed the model results show little effects of climate and edaphic factors on Bacilli numbers, which responded only to changes in overlying plant community, changes to which are expected to be relatively small compared to changing climate in the coming decade. Changes predicted for these bacterial classes reflect the relative strength of the climate drivers of Synechococcophycideae relative abundance, which include MAT (−0.22 standardized path coefficient), humidity (−0.46), and precipitation (−0.21) compared to weaker effects from vegetation [C3 Macrothermal plants: 0.03, C3 Mesothermal plants: 0.06, and C4 Megathermal plants (0.19), and soil factors (Conductivity: −0.08, pH: −0.03, and OC: −0.12)]. In contrast, predicted changes in Firmicutes Bacilli relative abundance were much smaller and driven by vegetation [C3 Macrothermal plants: −2.8, C3 Mesothermal plants: 0.74, and C4 Megathermal plants (2.9), compared to climate (temperature: −0.22, humidity: 0.00 and precipitation: −0.37) and soil factors (Conductivity: −0.01, pH: 0.11, and OC: 0.11)].

Combining 2030 predictions across taxa suggests that there will be non-significant changes in overall community composition (one-sample *t*-test: *t*_1380_ = 0.04, *p* = 0.52), as indicated by PCoA analyses of Percentage Difference (alias Bray-Curtis), though there were limited “hot spots” of change scattered around the coastal regions of Australia (Figure 5C). Relative abundance changed 5.2% (Standard deviation = 2.6% across 297 OTUs) between 2016 and 2030 for the 297 OTUs with relative abundances greater than 0.0003 per gram of soil, which was similar to the 6.6% (Standard deviation = 9.1%) change across the 2,284 OTUs with relative abundances between 1,000 and 10,000 per gram of soil.

Finally, using phylogenetic reconstruction methods (PICRUST) with our 2030 relative abundance predictions, we suggest the potential for relatively large changes in methane metabolism functional potential across the Australian continent. Comparison of shotgun metagenomic methane metabolism gene data for 370 of the 1,381 samples used in this study and methane metabolism genes predicted by PICRUSt in the same samples showed high congruence (Supplementary Figure S63).

## Discussion

Soil bacterial community structure and ecosystem function change with both natural and experimental perturbations and many soil, climate, and stochastic drivers of change have been identified (Bissett et al., 2010; Siciliano et al., 2014; Fierer, 2017; Delgado-Baquerizo et al., 2018). Utilizing this knowledge to predict bacterial community structure and relative abundances has proven difficult, however. Herein, we used continental scale SEMs that incorporate components of climate, vegetation, and edaphic properties to successfully model bacterial relative abundances in Australian soils by defining environmental niche spaces. We then applied these models to predict soil bacterial relative abundances under future climate scenarios, revealing localized hotspots of climate driven change.

Identification of phylogenetically relevant indicators of bacterial relative abundance response traits—environmental driver relationships quantified as SEM path coefficients—provides a novel set of indications of bacterial response traits for environmental predictions. We demonstrate that the SEM links derived from models are largely consistent with other methods of analysis, such as niche optima, and often display phylogenetic signal and conservancy. Thus, it is reasonable to consider SEM links as indicators of response traits (Suding et al., 2008) defining organism niche space. Importantly, those trait indicators most important in our models (e.g., those associated with salinity, pH, and water availability) likely have complex genetic underpinnings (Evans and Wallenstein, 2014; Barberán et al., 2017).

We utilized projected environmental conditions for 2030 to demonstrate the utility of using response traits derived from SEM links among climate, vegetation, and soil to predict potential changes in bacterial relative abundance and ecosystem function. The principal drivers in our predicted shifts in specific taxa (Figures 5A,B), bacterial dispersal (Figure 5C), and potential methane metabolism gene relative abundance changes (Figure 5D) depended on variations in climate, edaphic and associated vegetation changes. While the imputation of bacterial function from taxonomic information is not without caveats (Louca et al., 2018), we chose to show results for a function for which we had strong independent support for the validity of the imputed results (Supplementary Figure S63). Analysis of shotgun metagenomic data for 374 of the soil samples used herein showed high congruence with PICRUST imputed results. We were able to make similar predictions for other environmentally significant functions, but the robustness of these predictions does depend upon the validity of the other models on which they are based (e.g., climate and functional attribution models). These shortcomings do not, in our opinion, negate the opportunities created by these models, but do draw attention to the importance of understanding the underlying models and data being used. Australian soils have been shown to be important sinks for methane (Livesley et al., 2009; Bissett et al., 2011; Fest et al., 2017), and the majority (92%) of genes predicted to be involved in the methane metabolism pathway in these soils were associated with methane oxidation. The predicted changes to methane metabolism associated genes occur as hotspots of change at the continental scale, associated with predicted changes in aridity and plant productivity. These predicted changes concur with previous work that has shown methane oxidation activity is influenced by moisture (Fest et al., 2017) and that methane oxidisers have exhibited niche partitioning across salinity and moisture gradients in Australian soils (Bissett et al., 2011), and support the utilization of our modeling approach to identify environmental processes likely to influenced by change.

The responses of individual taxa were taxa dependent, with Synechococcophycideae showing localized hotspots of response, which may have important effects on soil structure and N fixation in these areas. Bacilli, on the other hand, showed less response, perhaps a reflection of specific lifestyle traits, such as spore forming ability (Bissett et al., 2010), which may confer resilience to the relatively short term changes modeled here. The ability to predict hotspots of response to change also identifies areas of likely high value in programs seeking to monitor and understand likely responses of bacteria and their ecosystem functions to change. The dominance of direct climate drivers and indirect climate-vegetation drivers are likely due to the relatively short timeframe of our forward predictions (~12 years to 2030). The potential response of bacterial communities to precipitation, MAT, and humidity is likely rapid, whereas vegetation changes will likely lag by some years behind climate (Corlett and Westcott, 2013; Ash et al., 2017). Soil properties (e.g., pH, OC) will likely have long time lags before climate impacts become evident. The ability to associate changes in these environmental factors with bacterial relative abundances and to infer, in this case, the likely effect on functional gene dispersal, will be useful for the prediction of potential environmental function and the incorporation of microbial information into ecosystem models (Coles et al., 2017). Community responses to perturbation will likely be driven by both the environmental niche preferences of organisms, and by within-community dynamics (Evans and Wallenstein, 2014). The models presented are only able to predict responses based on environmental change; including competitive and other interactions among OTUs and incorporating other microbial interactions (e.g., Fungal-Bacterial interrelationships) into the SEM models (e.g., Mamet et al., 2017) will enable stronger predictions of potential soil function under a changing climate.

Predictions of niche space and optima were dependent on taxonomic level, and were not always congruent through subtaxa or with SEM path coefficients (Figure 2; Table 1). These results support the ecological coherence patterns 1, 3, and 4 described in the introduction (Koeppel and Wu, 2012). While it is more difficult to define and observe these patterns with continuous, rather than binary, habitat data (e.g., pH, salinity, etc.), conceptually at least we have identified various combinations of these patterns in our niche modeling. Generally, once a habitat association was defined, subtaxa continued to exhibit the association, lending support for all of the above patterns except 2, where parent taxa show an association, but subtaxa do not. We additionally found cases where both parent and subtaxa did not show an association with the habitat variables presented. It seems likely that the taxonomic level at which habitat associations become evident is related to the diversity within the taxon in question. The Proteobacteria and the Verrucomicrobia, for example, are very diverse phyla and it not surprising that they do not exhibit clear patterns of habitat association at the phylum level, but often did at higher taxonomic resolution, e.g., at the Class level, Proteobacteria did begin to exhibit more defined niche preferences. Presently much soil microbial data is presented either at relatively course taxonomic levels or grouped to indicate categorical habitat associations (e.g., Delgado-Baquerizo et al., 2018), a necessity given the sparse observations available for many individual taxa at finer taxonomic resolution. This situation is likely to change as more observations become available and models such as those presented here will help define likely useful starting taxonomic resolutions to more firmly assign associations. The benefits of working at higher resolution, once enough observations are available is evidenced, for example, in efforts to characterize ecotypes of marine Prochlorococcus and SAR11 (Brown et al., 2012; Kashtan et al., 2014), something not yet achieved in soils.

The inability to define niche width and/or optima does not imply no association with environmental traits, merely that there were no inflection points in the relative abundances across the range of that environmental factor in the soils studied. For example, Acidobacteria showed nearly linear, negative, relationships with pH and conductivity (Supplementary Figure S2), but because they were ubiquitous across all pH and conductivity conditions and no distribution inflection points were found (compare with Actinobacteria and conductivity; Supplementary Figure S3) it was not possible to define a phylum level niche space for these organisms. These patterns were observed for both edaphic and climate related variables; for example, Bacteroidetes showed strong negative relationships with temperature and humidity, but no defined optima. The strong association of these phyla with predictor variables did, however, allow successful prediction of their relative abundances.

Many currently poorly defined phyla (e.g., candidate phyla) were present in >50% of our samples and our models suggest their preferred habitat associations and distributional drivers. The reduced genome, putative symbiotic phyla in TM6, TM7, and Elusimicrobia were found in >88% of soils and while they often did not display habitat optima for edaphic drivers they showed high associations with plant drivers. The WPS2 and AD3, recently suggested to be ecologically important in Antarctic oligotrophic soils by scavenging atmospheric trace gases (Ji et al., 2016), were also prevalent in Australian soils (88 and 72% of samples respectively) and showed preferences for lower pH, low OC, dryer, and warmer soils. Their high prevalence and association with many warm soils suggest they are likely to be important in nutrient depleted soils globally and not restricted to polar habitats. The relationships of bacterial phyla with edaphic, climate and vegetation variables in Figures 2, 4 allow estimations of habitat preferences for cryptic and difficult to culture phyla and their potential to extend and investigate their putative ranges. Our statistical approach thus provides a novel method for understanding the environmental preferences and likely ranges of cryptic phylotypes. Such information will enhance both cultivation efforts and opportunities to “capture” population genomes (Hug et al., 2016; Parks et al., 2017). Extending this approach to lower taxonomic levels in continental-scale datasets provides a way to generate OTU-level habitat-preference summaries for the large numbers of currently uncharacterized organisms. In turn this will enable a better predictive understanding of how soil bacterial communities assemble and variation in their functional potential across space, time, and in response to anthropogenic change.

The SEM diagrams represent aggregate responses of complex bacterial systems to abiotic drivers, and as such, it is not surprising that many of these links were relatively weak or weakly significant (*p* < 0.05). Simple, continuous traits are less likely to be strongly phylogenetically conserved (Goberna and Verdú, 2015) and, given the relatively coarse phylogenetic resolution used in our models, the weak signal from some continuous environmental variables was expected. One of the problems facing microbial ecology is the increasing size of the phylogenetic and niche space being interrogated. The models we present require reasonable levels of environmental trait conservatism at the various resolutions used, though it is likely that there is also valuable information regarding soil microbial distributions that is not resolvable using coarse taxonomic groups, but rather requires detailed information regarding microbial ecotypes (for example Kashtan et al., 2014). Weak signals in our models can thus be interpreted as evidence for either a diversity of bacteria-environment responses that may be detectable at finer phylogenetic resolutions, or of an environmental driver that is generally unimportant for the phylotype in question. As larger integrated datasets (providing more observations in time and space) are assembled at higher taxonomic resolution, the application of predictive models to specific microbial taxa and functions will become more practical. In order to have enough observations the models we present are largely limited to high taxonomic ranks (e.g., phylum), a situation likely to change as larger, coordinated datasets are assembled (Bissett et al., 2016; Thompson et al., 2017).

The models we have employed are able to predict continental-scale changes in bacterial relative abundances by utilizing indicators of response traits (model coefficients). The application of these models has been shown in their utility in predicting changes in potential function, and by extension ecosystem services. The reliability of these predictions is dependent on the validity of the future climate and edaphic models used to predict future scenarios, as well as the sequence data used herein. Both of which will be strengthened in the future with growing genome-level sequence data and more robust future environmental predictions. Bacteria are key regulators of biogeochemical cycles at both local and global scales, thus understanding their responses to environmental perturbation is important in predicting and managing change. We provide postulated causal networks able to predict bacterial relative abundances and niche preference across large scales. Using model derived coefficients as microbial traits allows prediction and understanding of microbial response to change, identifies likely hotspots of change and vulnerability and enables more targeted attempts to culture and isolate cryptic and difficult to culture organisms.

## Data availability statement

The datasets presented in this study can be found in online repositories. The names of the repository/repositories and accession number(s) can be found at: https://www.ncbi.nlm.nih.gov/, PRJNA317932.

## Author contributions

AB, SM, EL, and SS: conceptualization, methodology, data curation, analysis, and manuscript production and writing. All authors contributed to the article and approved the submitted version.

## Conflict of interest

The authors declare that the research was conducted in the absence of any commercial or financial relationships that could be construed as a potential conflict of interest.

## Publisher’s note

All claims expressed in this article are solely those of the authors and do not necessarily represent those of their affiliated organizations, or those of the publisher, the editors and the reviewers. Any product that may be evaluated in this article, or claim that may be made by its manufacturer, is not guaranteed or endorsed by the publisher.

## Footnotes

^1^ https://data.bioplatforms.com/organization/about/australian-microbiome

^2^ https://spatial.ala.org.au/
