# Supplementary material for: Linking niche size and phylogenetic signals to predict future soil microbial relative abundances
Source: Front Microbiol. 2023 Aug 14;14:1097909. doi: 10.3389/fmicb.2023.1097909 (PMC10461061; doi:10.3389/fmicb.2023.1097909)
Supplement: Supplementary file 1 [file Data_Sheet_1.zip › FigureS6-62Supplemental Phyla Direct SEM Observations.R1.pptx]

## Slide 1
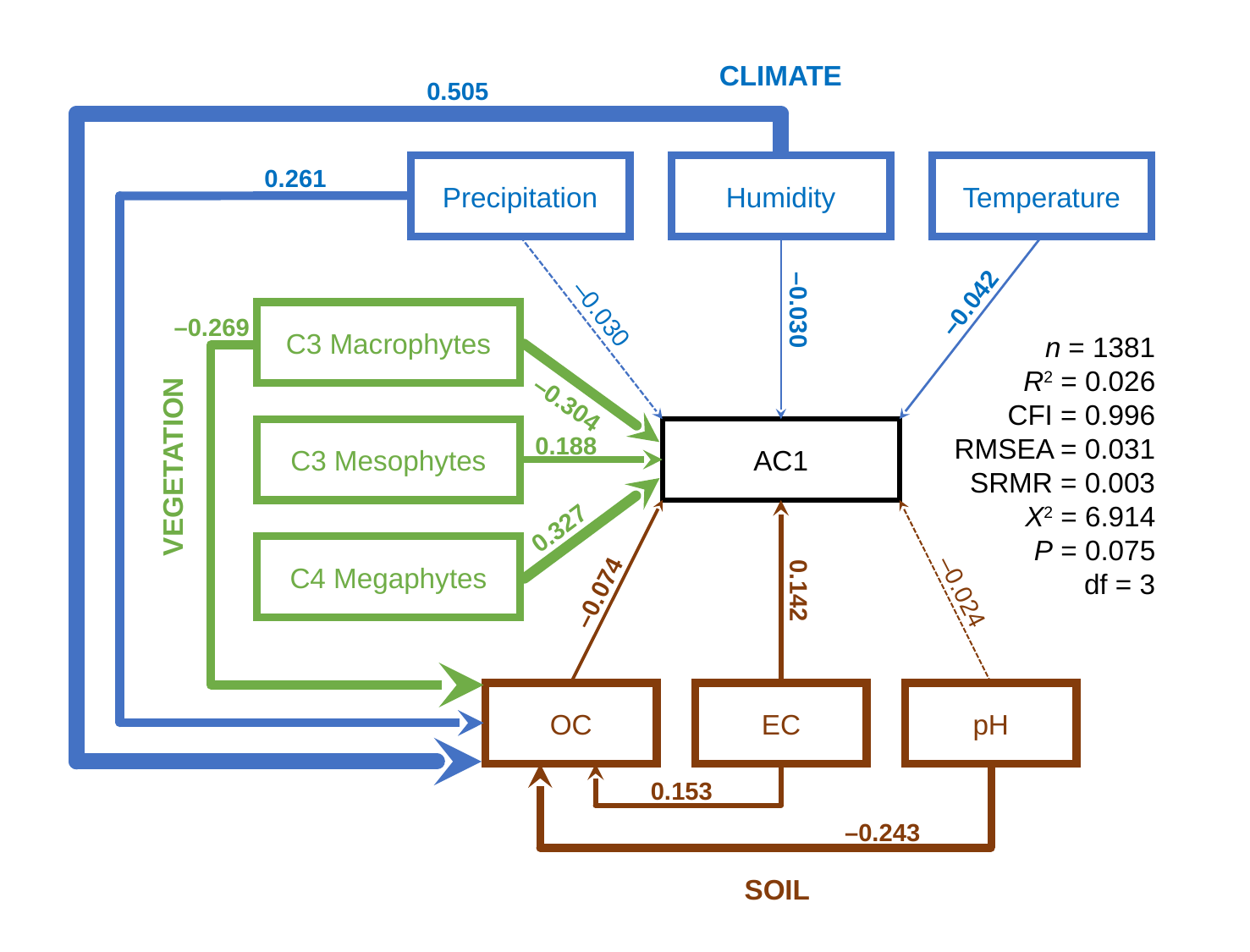

CLIMATE
0.505
Precipitation
Humidity
Temperature
0.261
–0.042
–0.030
–0.030
C3 Macrophytes
–0.269
n = 1381
R2 = 0.026
CFI = 0.996
RMSEA = 0.031
SRMR = 0.003
Χ2 = 6.914
P = 0.075
df = 3
–0.304
C3 Mesophytes
AC1
0.188
VEGETATION
0.327
C4 Megaphytes
0.142
–0.024
–0.074
OC
EC
pH
0.153
–0.243
SOIL

## Slide 2
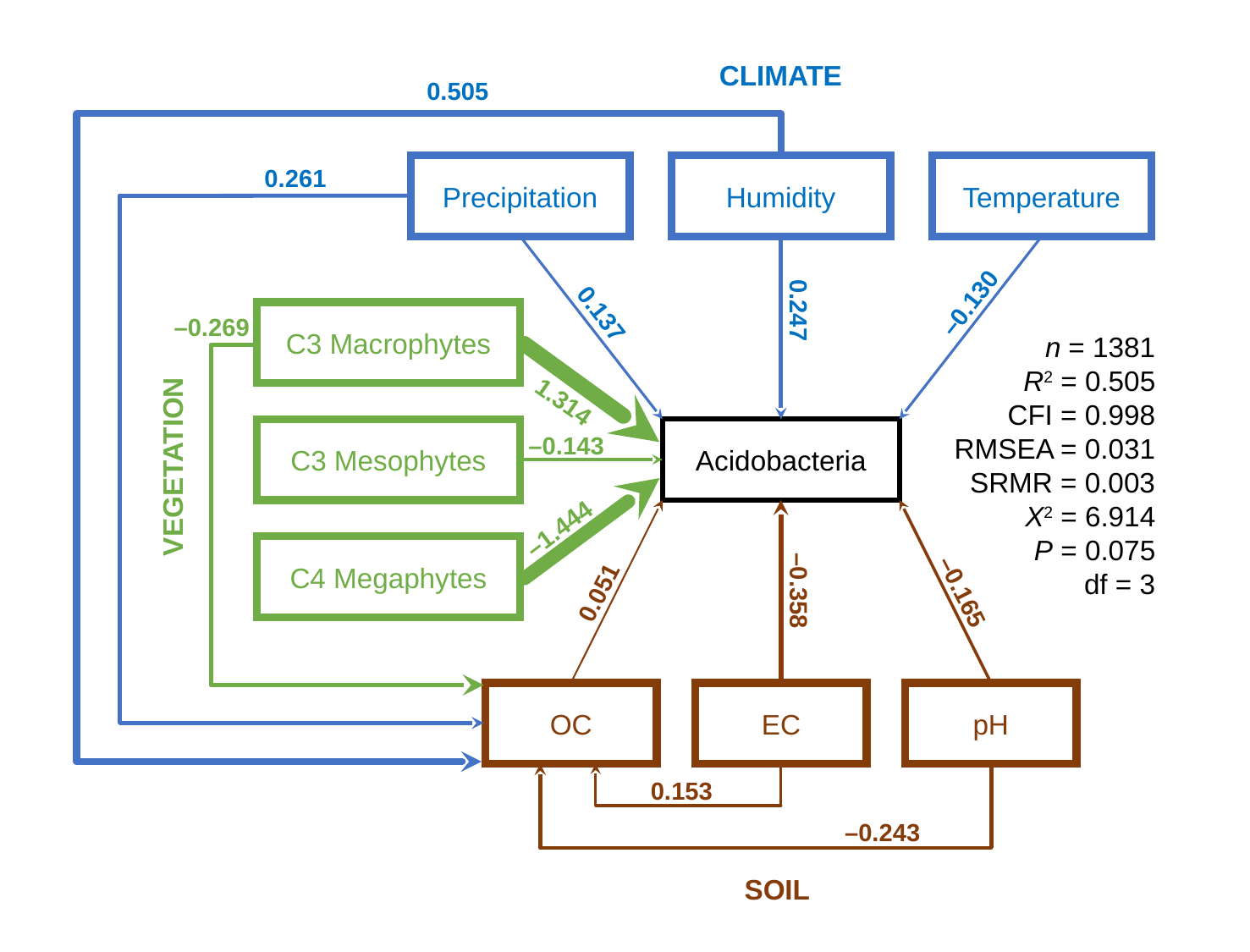

CLIMATE
0.505
Precipitation
Humidity
Temperature
0.261
–0.130
0.247
0.137
C3 Macrophytes
–0.269
n = 1381
R2 = 0.505
CFI = 0.998
RMSEA = 0.031
SRMR = 0.003
Χ2 = 6.914
P = 0.075
df = 3
1.314
C3 Mesophytes
Acidobacteria
–0.143
VEGETATION
–1.444
C4 Megaphytes
–0.358
–0.165
0.051
OC
EC
pH
0.153
–0.243
SOIL

## Slide 3
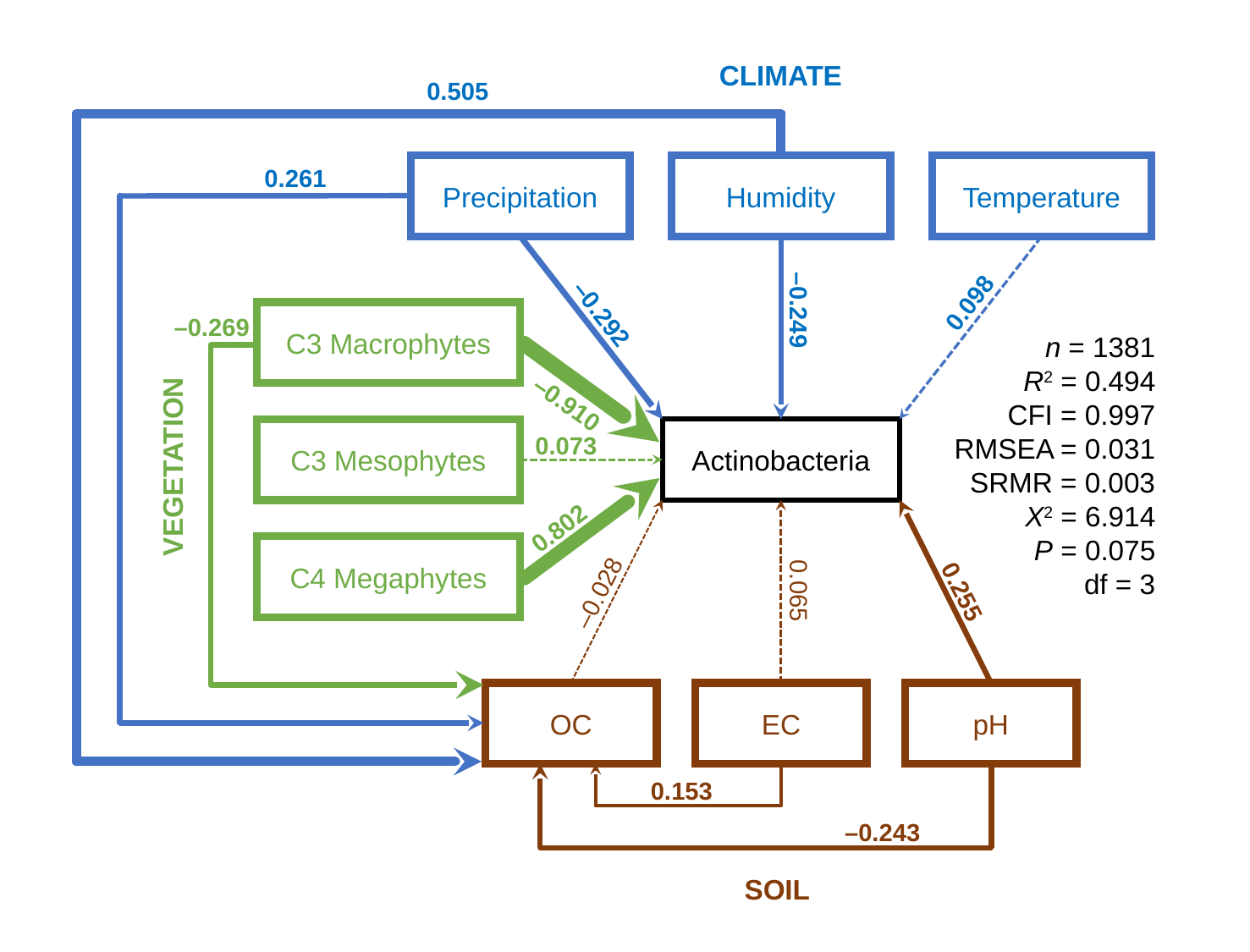

CLIMATE
0.505
Precipitation
Humidity
Temperature
0.261
0.098
–0.249
–0.292
C3 Macrophytes
–0.269
n = 1381
R2 = 0.494
CFI = 0.997
RMSEA = 0.031
SRMR = 0.003
Χ2 = 6.914
P = 0.075
df = 3
–0.910
C3 Mesophytes
Actinobacteria
0.073
VEGETATION
0.802
C4 Megaphytes
0.065
0.255
–0.028
OC
EC
pH
0.153
–0.243
SOIL

## Slide 4
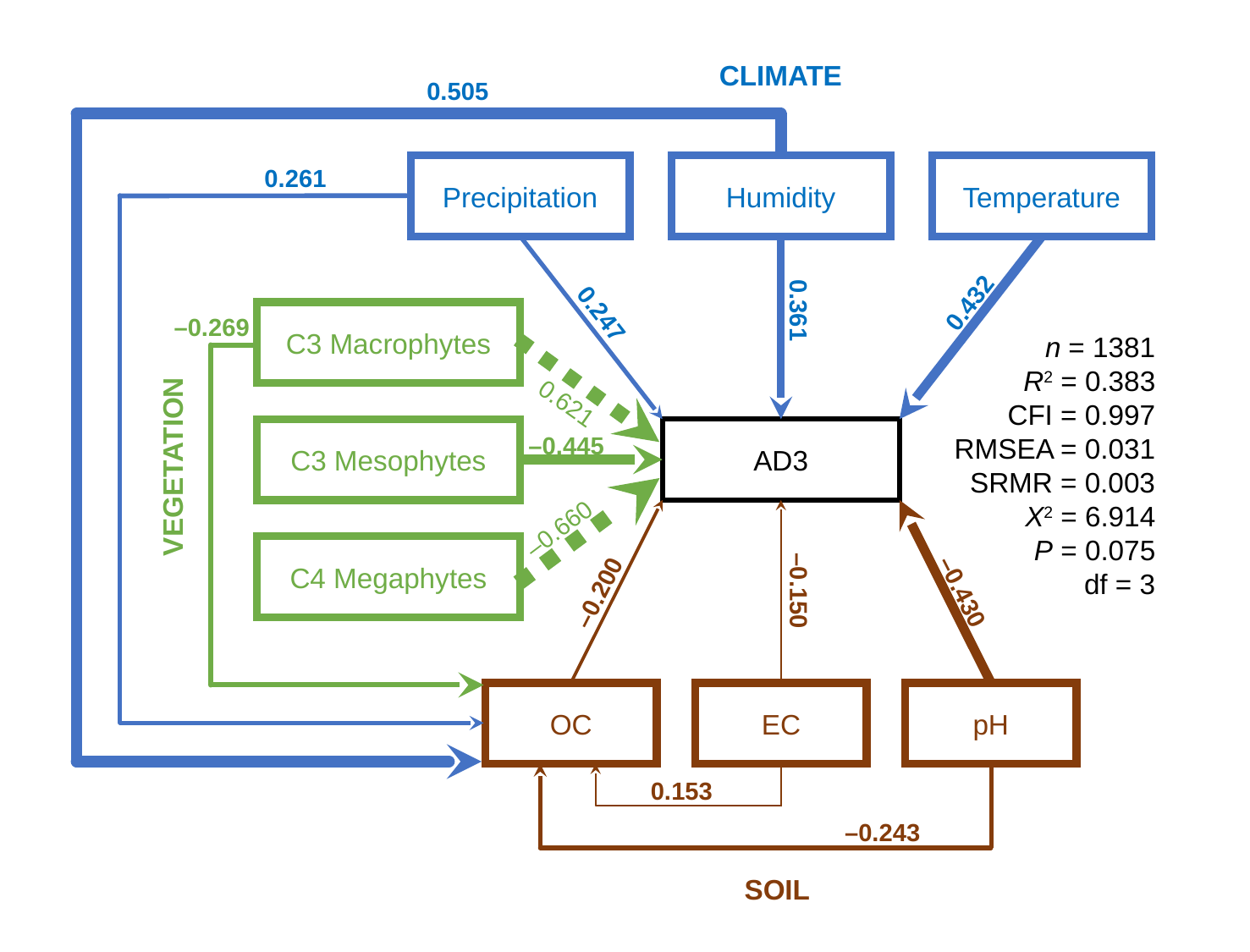

CLIMATE
0.505
Precipitation
Humidity
Temperature
0.261
0.432
0.361
0.247
C3 Macrophytes
–0.269
n = 1381
R2 = 0.383
CFI = 0.997
RMSEA = 0.031
SRMR = 0.003
Χ2 = 6.914
P = 0.075
df = 3
0.621
C3 Mesophytes
AD3
–0.445
VEGETATION
–0.660
C4 Megaphytes
–0.150
–0.430
–0.200
OC
EC
pH
0.153
–0.243
SOIL

## Slide 5
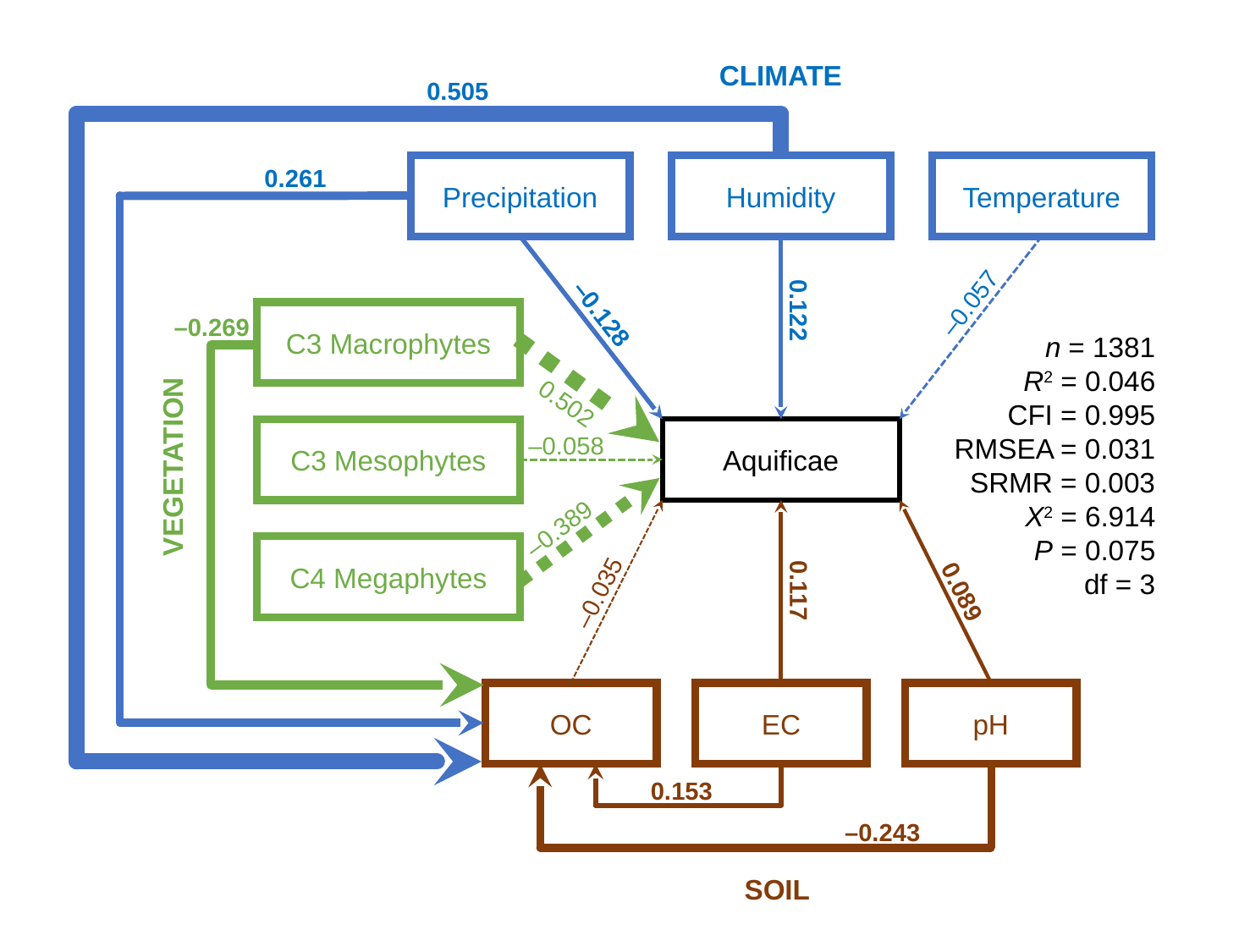

CLIMATE
0.505
Precipitation
Humidity
Temperature
0.261
–0.057
0.122
–0.128
C3 Macrophytes
–0.269
n = 1381
R2 = 0.046
CFI = 0.995
RMSEA = 0.031
SRMR = 0.003
Χ2 = 6.914
P = 0.075
df = 3
0.502
C3 Mesophytes
Aquificae
–0.058
VEGETATION
–0.389
C4 Megaphytes
0.117
0.089
–0.035
OC
EC
pH
0.153
–0.243
SOIL

## Slide 6
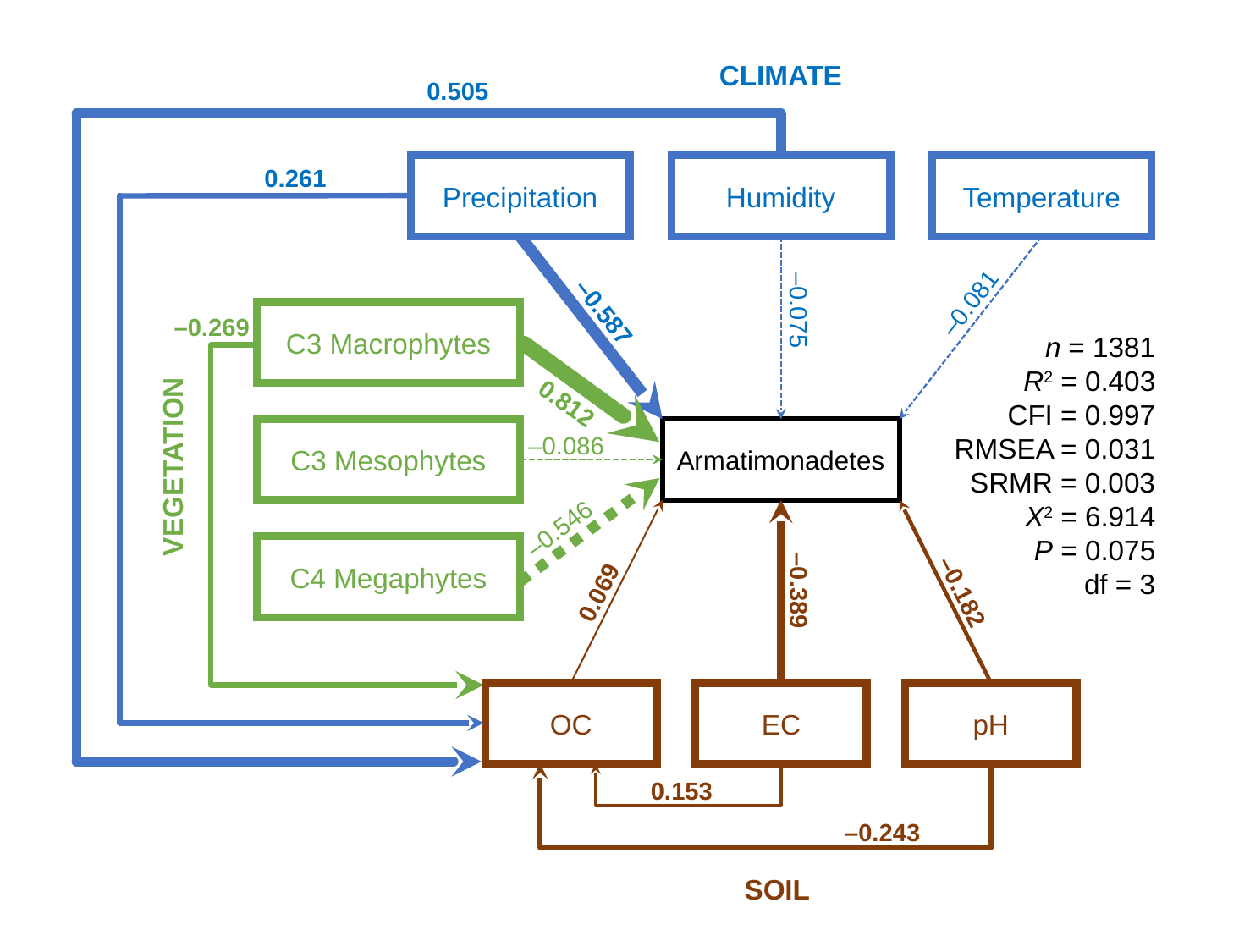

CLIMATE
0.505
Precipitation
Humidity
Temperature
0.261
–0.081
–0.075
–0.587
C3 Macrophytes
–0.269
n = 1381
R2 = 0.403
CFI = 0.997
RMSEA = 0.031
SRMR = 0.003
Χ2 = 6.914
P = 0.075
df = 3
0.812
C3 Mesophytes
Armatimonadetes
–0.086
VEGETATION
–0.546
C4 Megaphytes
–0.389
–0.182
0.069
OC
EC
pH
0.153
–0.243
SOIL

## Slide 7
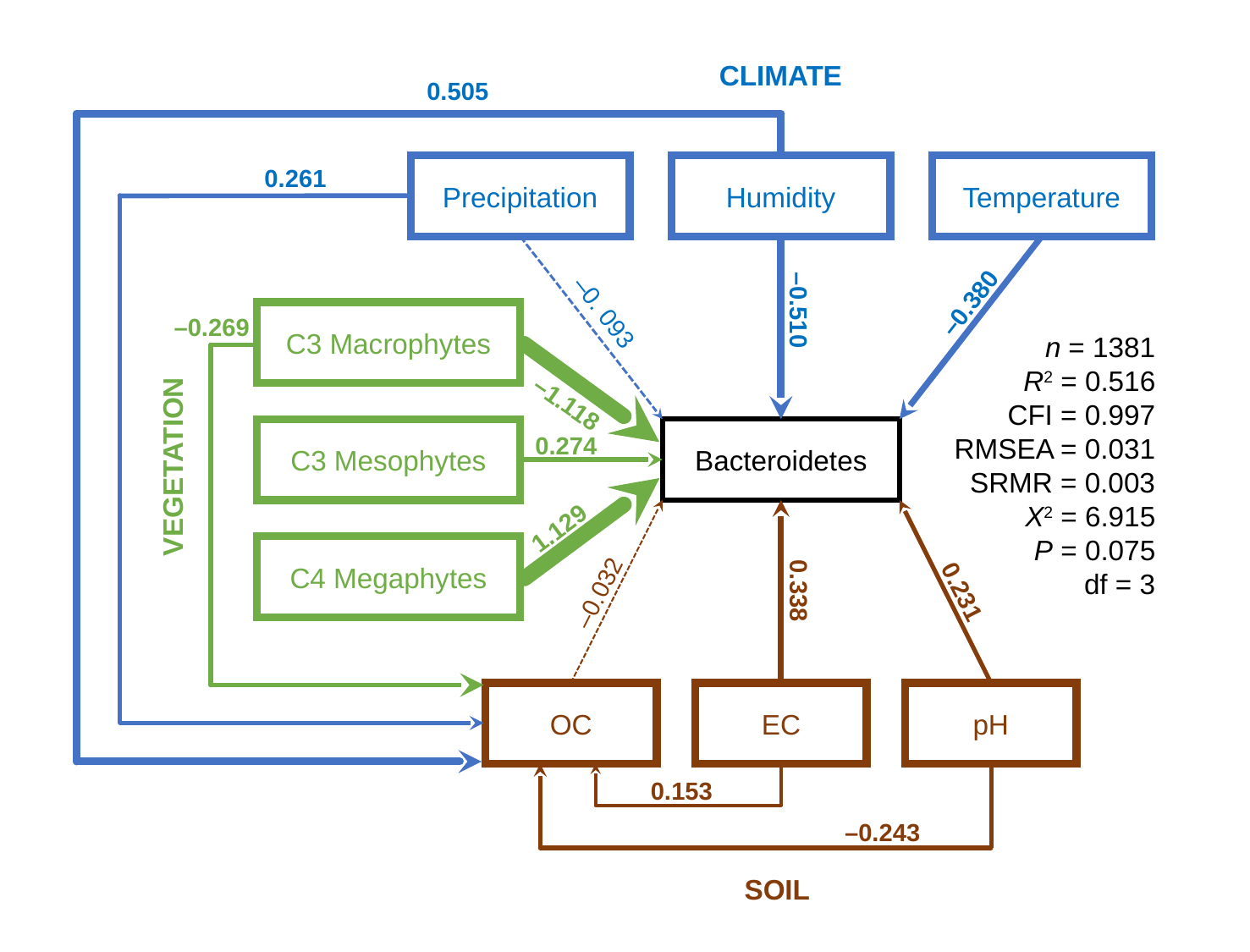

CLIMATE
0.505
Precipitation
Humidity
Temperature
0.261
–0.380
–0.510
–0. 093
C3 Macrophytes
–0.269
n = 1381
R2 = 0.516
CFI = 0.997
RMSEA = 0.031
SRMR = 0.003
Χ2 = 6.915
P = 0.075
df = 3
–1.118
C3 Mesophytes
Bacteroidetes
0.274
VEGETATION
1.129
C4 Megaphytes
0.338
0.231
–0.032
OC
EC
pH
0.153
–0.243
SOIL

## Slide 8
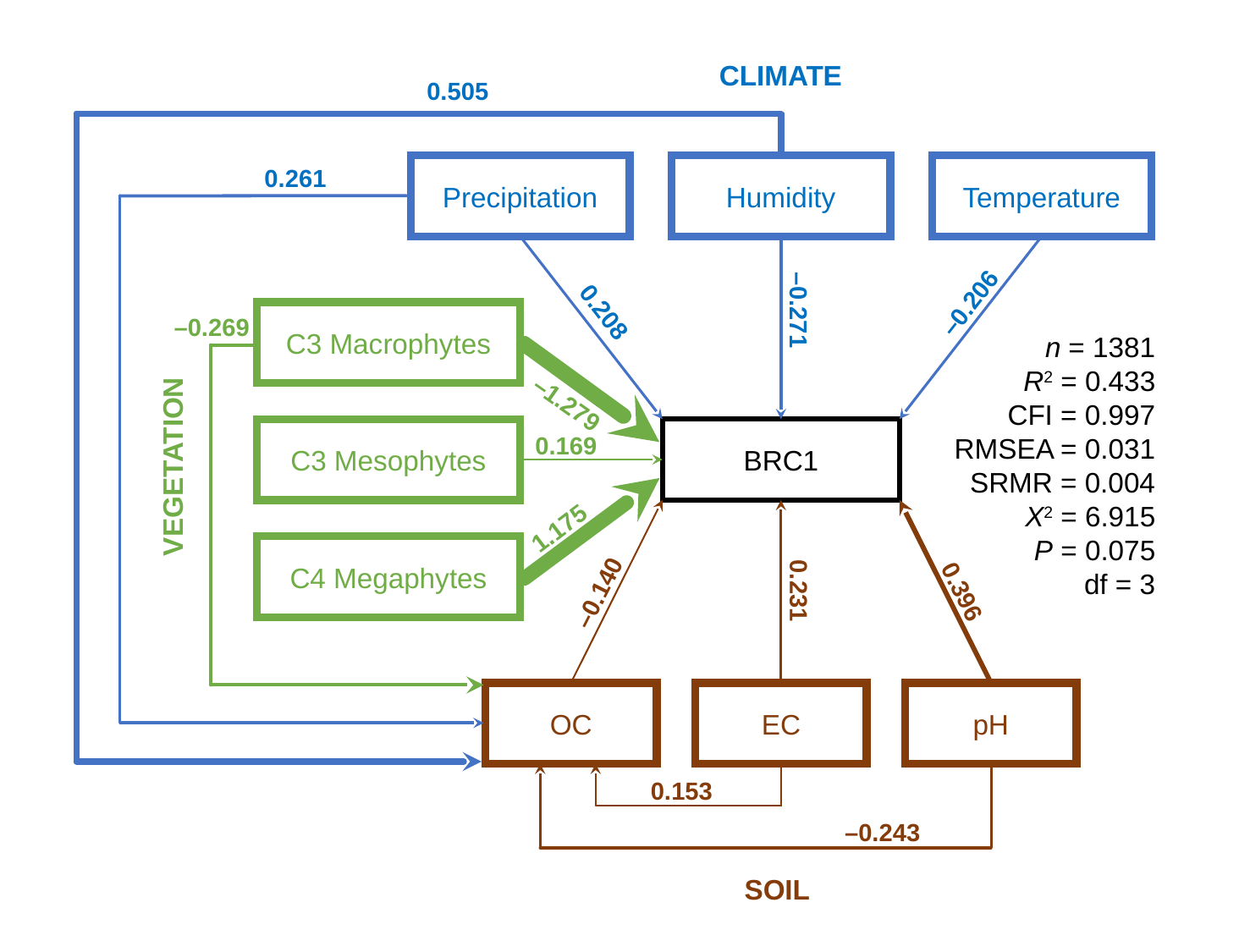

CLIMATE
0.505
Precipitation
Humidity
Temperature
0.261
–0.206
–0.271
0.208
C3 Macrophytes
–0.269
n = 1381
R2 = 0.433
CFI = 0.997
RMSEA = 0.031
SRMR = 0.004
Χ2 = 6.915
P = 0.075
df = 3
–1.279
C3 Mesophytes
BRC1
0.169
VEGETATION
1.175
C4 Megaphytes
0.231
0.396
–0.140
OC
EC
pH
0.153
–0.243
SOIL

## Slide 9
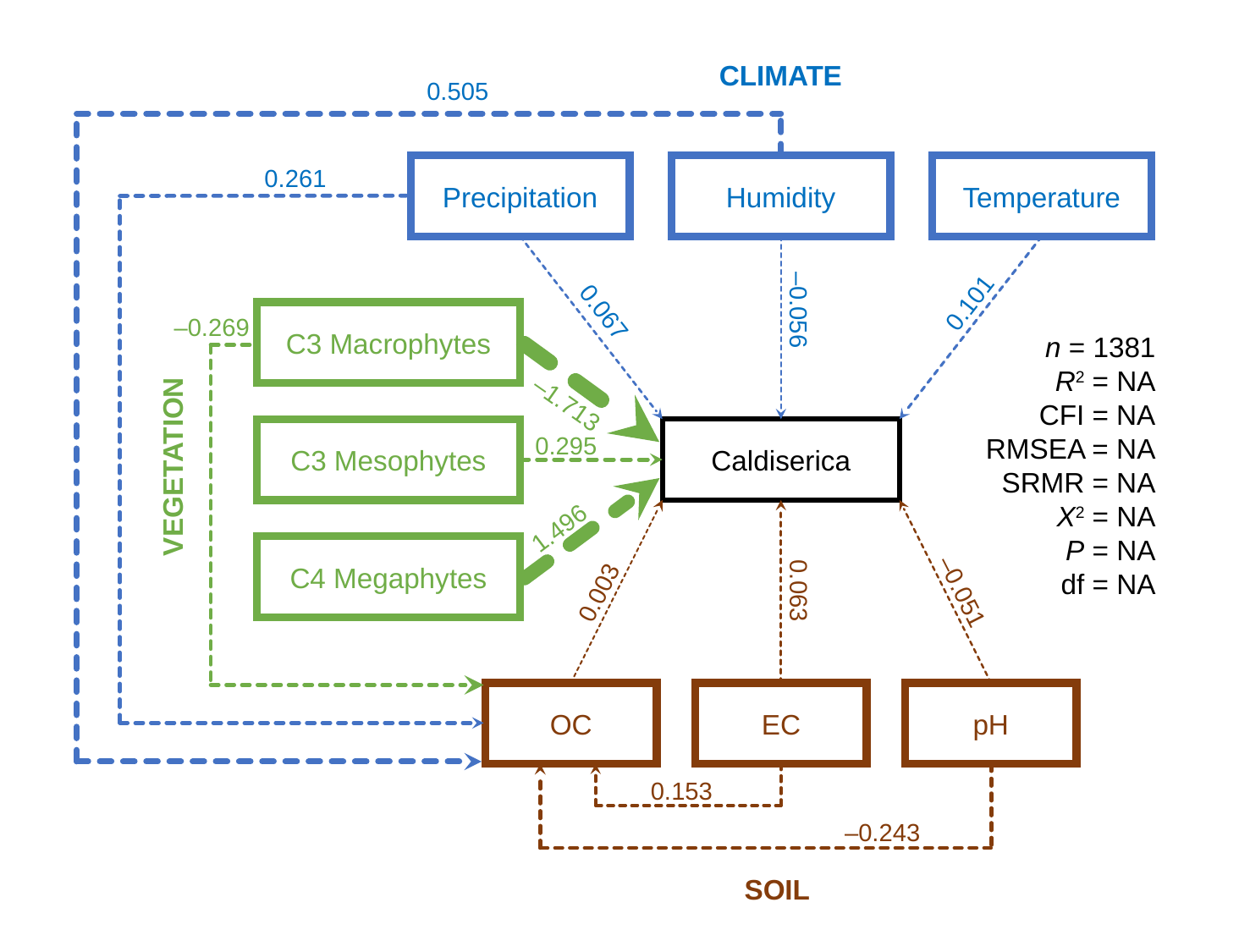

CLIMATE
0.505
Precipitation
Humidity
Temperature
0.261
0.101
–0.056
0.067
C3 Macrophytes
–0.269
n = 1381
R2 = NA
CFI = NA
RMSEA = NA
SRMR = NA
Χ2 = NA
P = NA
df = NA
–1.713
C3 Mesophytes
Caldiserica
0.295
VEGETATION
1.496
C4 Megaphytes
0.063
–0.051
0.003
OC
EC
pH
0.153
–0.243
SOIL

## Slide 10
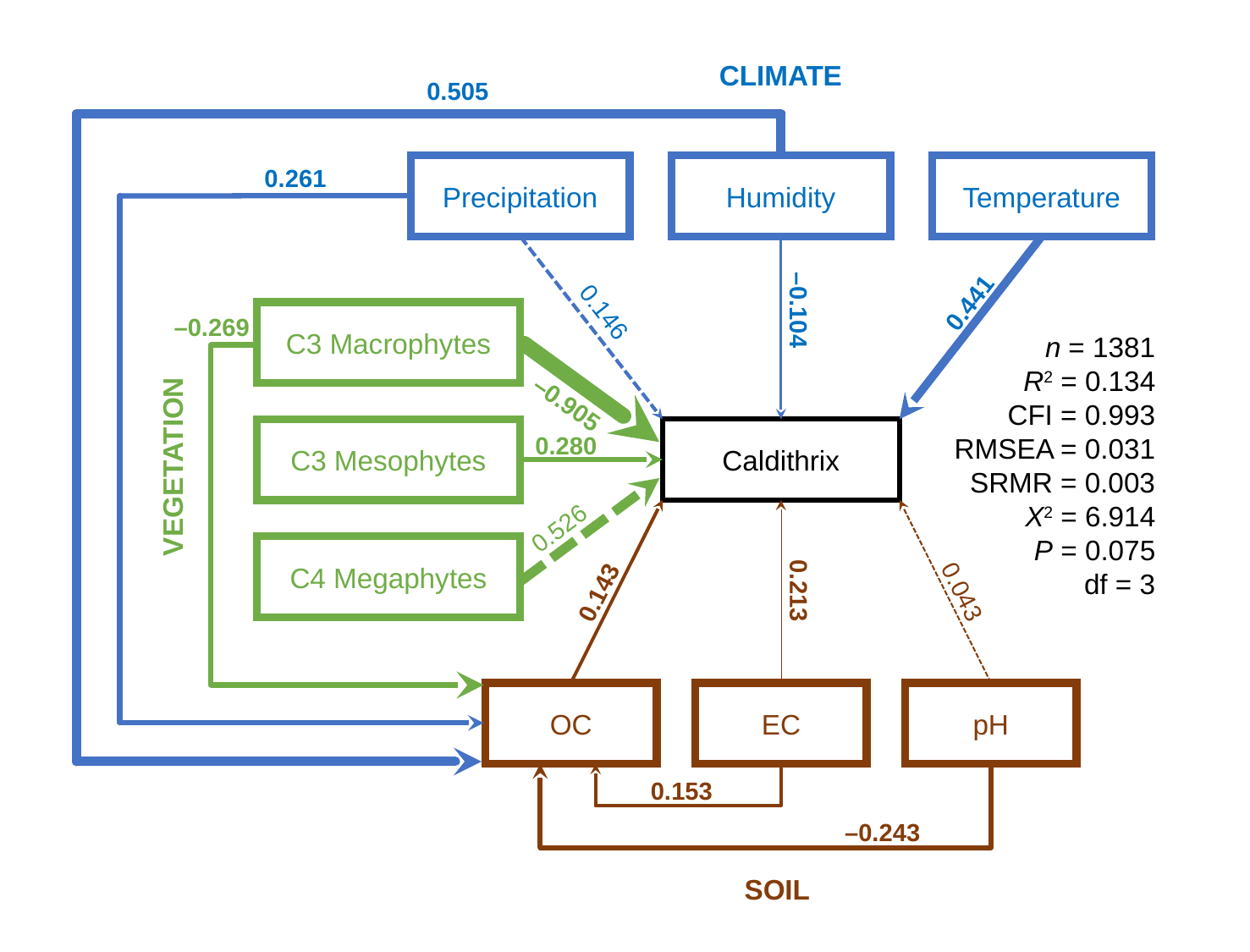

CLIMATE
0.505
Precipitation
Humidity
Temperature
0.261
0.441
–0.104
0.146
C3 Macrophytes
–0.269
n = 1381
R2 = 0.134
CFI = 0.993
RMSEA = 0.031
SRMR = 0.003
Χ2 = 6.914
P = 0.075
df = 3
–0.905
C3 Mesophytes
Caldithrix
0.280
VEGETATION
0.526
C4 Megaphytes
0.213
0.043
0.143
OC
EC
pH
0.153
–0.243
SOIL

## Slide 11
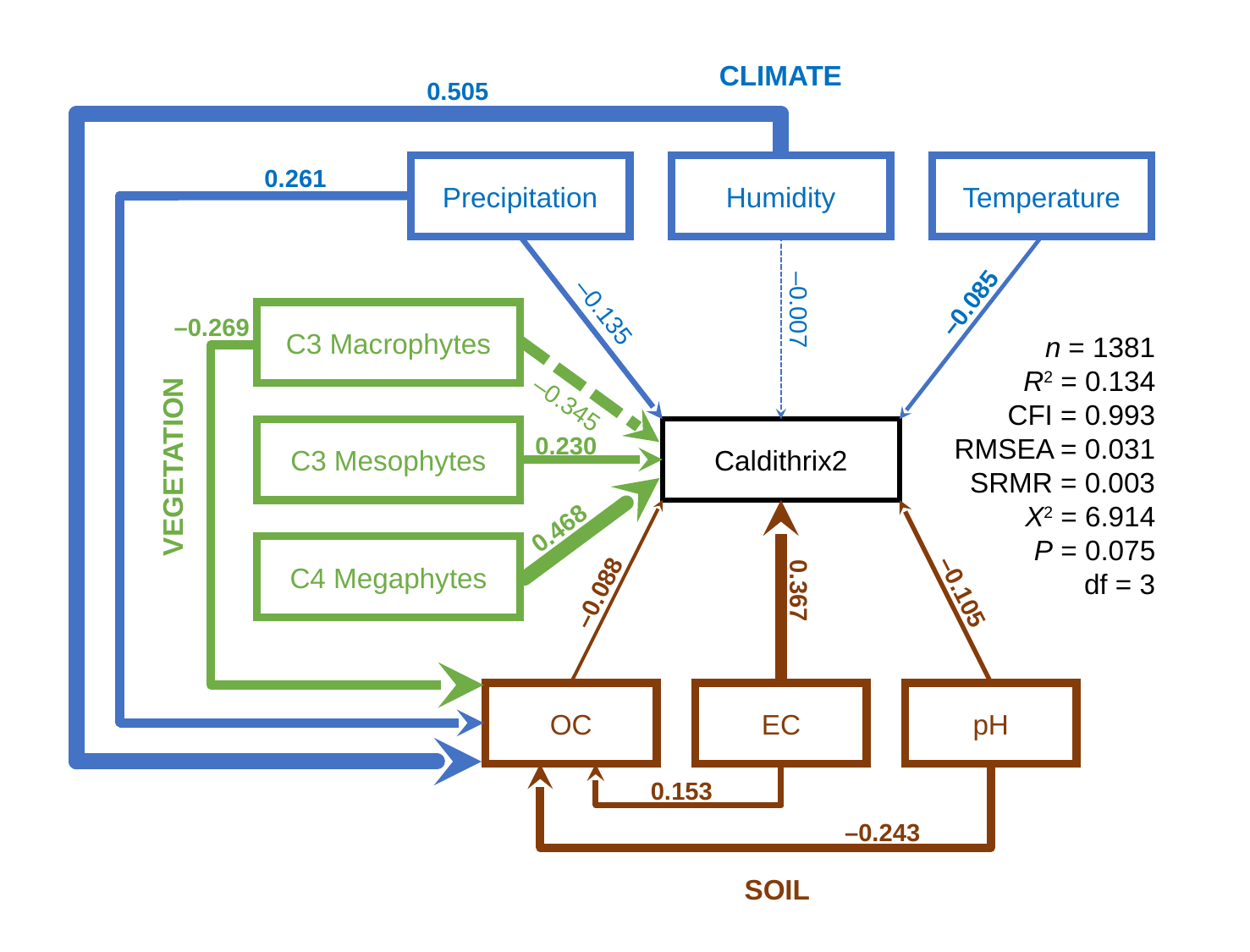

CLIMATE
0.505
Precipitation
Humidity
Temperature
0.261
–0.085
–0.007
–0.135
C3 Macrophytes
–0.269
n = 1381
R2 = 0.134
CFI = 0.993
RMSEA = 0.031
SRMR = 0.003
Χ2 = 6.914
P = 0.075
df = 3
–0.345
C3 Mesophytes
Caldithrix2
0.230
VEGETATION
0.468
C4 Megaphytes
0.367
–0.105
–0.088
OC
EC
pH
0.153
–0.243
SOIL

## Slide 12
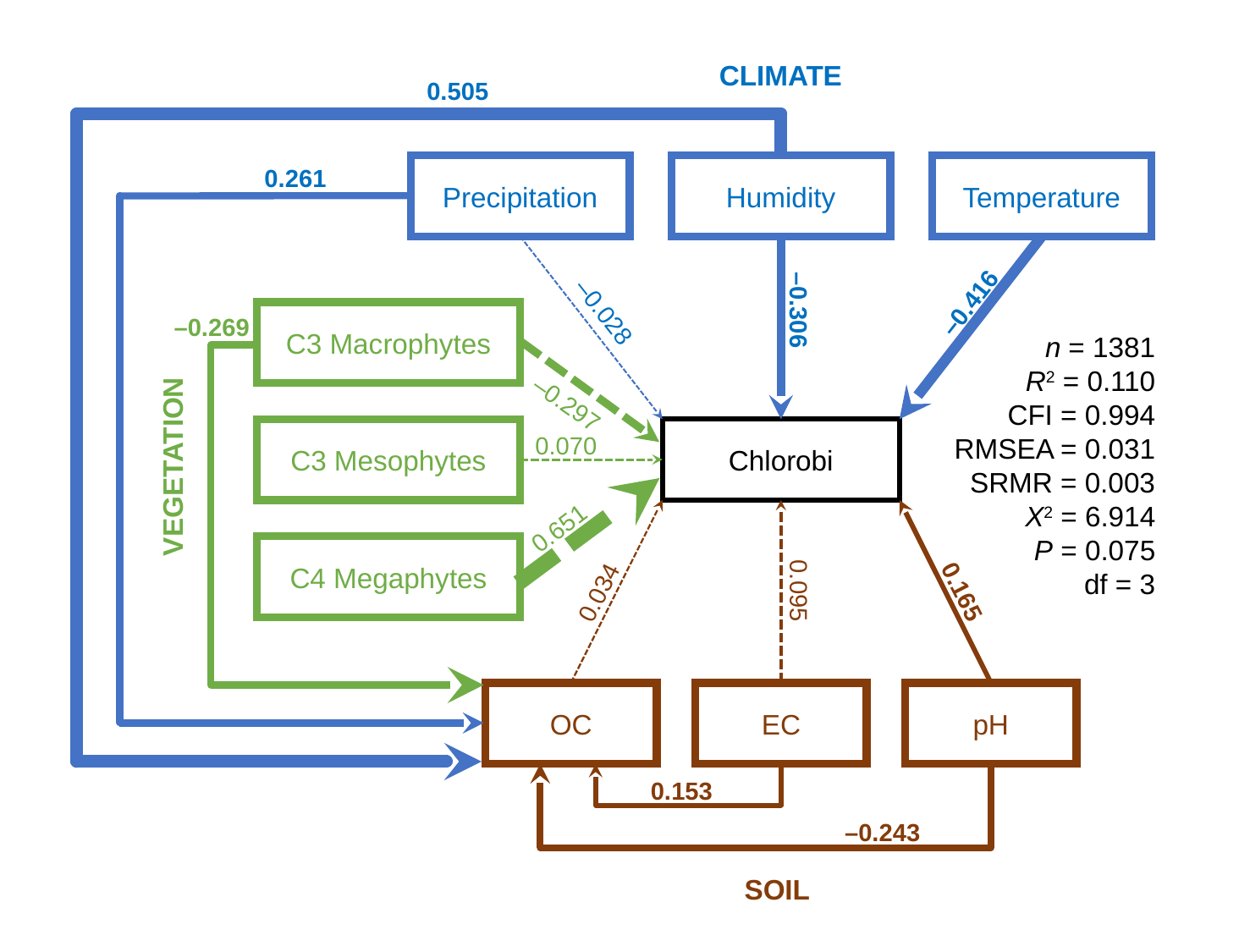

CLIMATE
0.505
Precipitation
Humidity
Temperature
0.261
–0.416
–0.306
–0.028
C3 Macrophytes
–0.269
n = 1381
R2 = 0.110
CFI = 0.994
RMSEA = 0.031
SRMR = 0.003
Χ2 = 6.914
P = 0.075
df = 3
–0.297
C3 Mesophytes
Chlorobi
0.070
VEGETATION
0.651
C4 Megaphytes
0.095
0.165
0.034
OC
EC
pH
0.153
–0.243
SOIL

## Slide 13
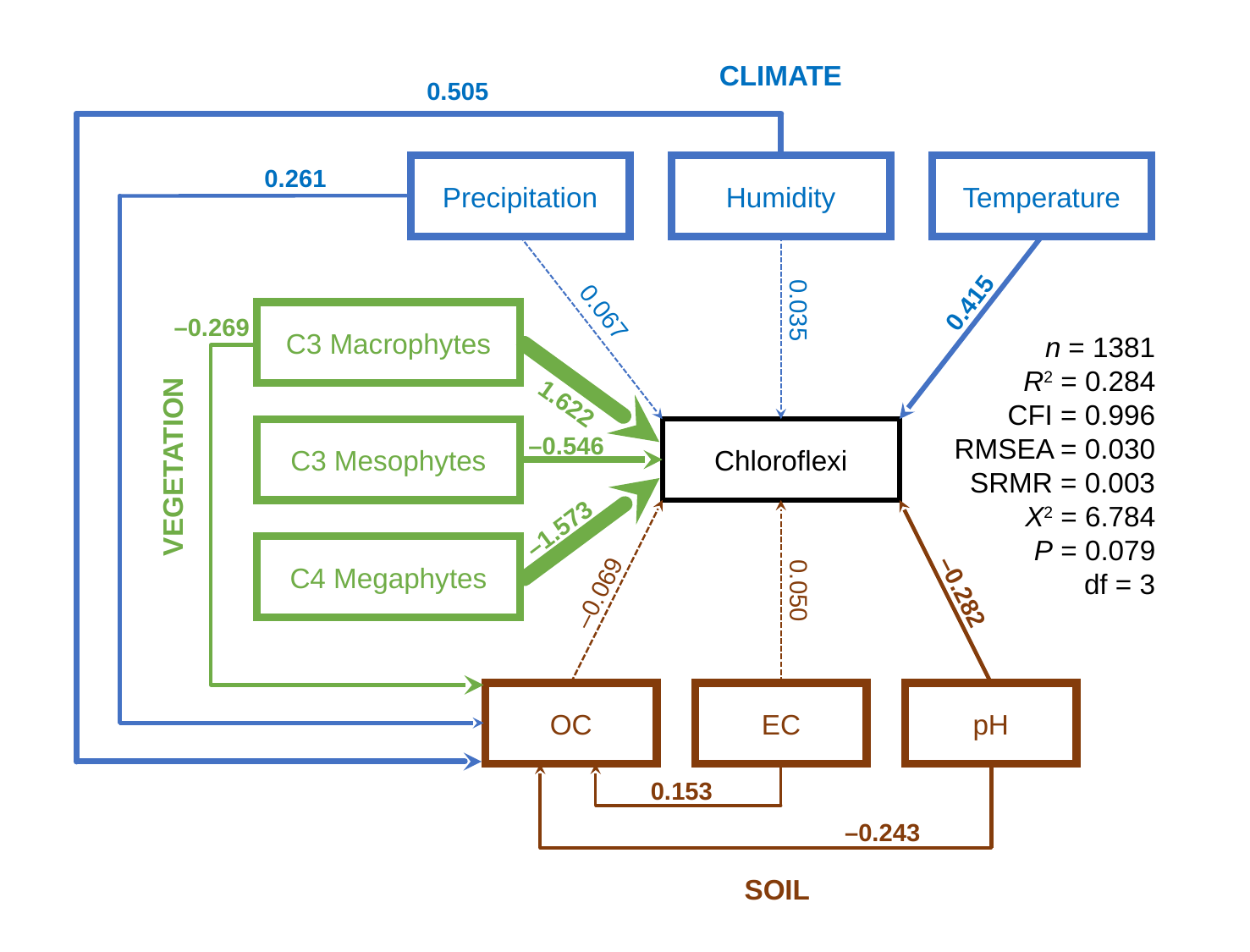

CLIMATE
0.505
Precipitation
Humidity
Temperature
0.261
0.415
0.035
0.067
C3 Macrophytes
–0.269
n = 1381
R2 = 0.284
CFI = 0.996
RMSEA = 0.030
SRMR = 0.003
Χ2 = 6.784
P = 0.079
df = 3
1.622
C3 Mesophytes
Chloroflexi
–0.546
VEGETATION
–1.573
C4 Megaphytes
0.050
–0.282
–0.069
OC
EC
pH
0.153
–0.243
SOIL

## Slide 14
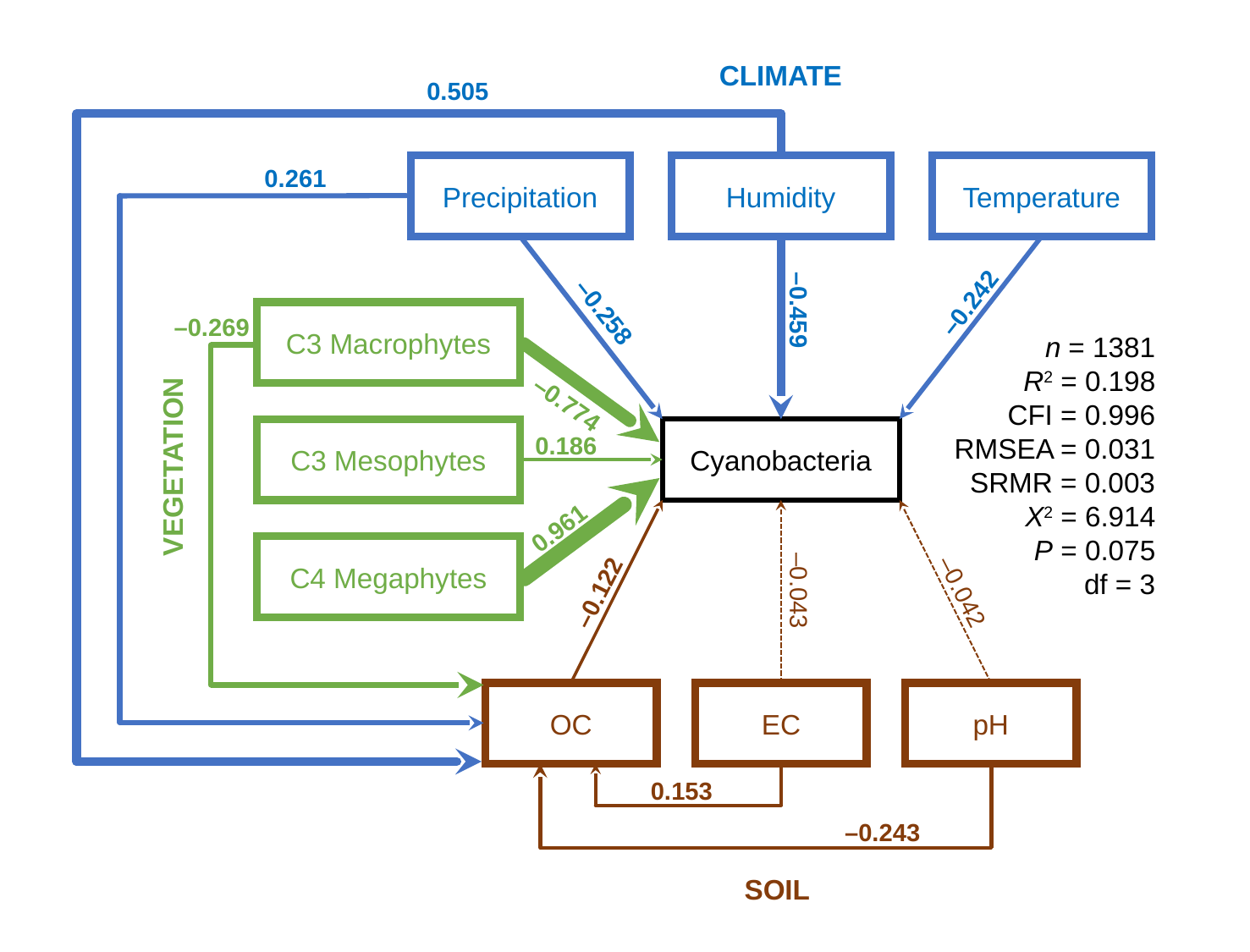

CLIMATE
0.505
Precipitation
Humidity
Temperature
0.261
–0.242
–0.459
–0.258
C3 Macrophytes
–0.269
n = 1381
R2 = 0.198
CFI = 0.996
RMSEA = 0.031
SRMR = 0.003
Χ2 = 6.914
P = 0.075
df = 3
–0.774
C3 Mesophytes
Cyanobacteria
0.186
VEGETATION
0.961
C4 Megaphytes
–0.043
–0.042
–0.122
OC
EC
pH
0.153
–0.243
SOIL

## Slide 15
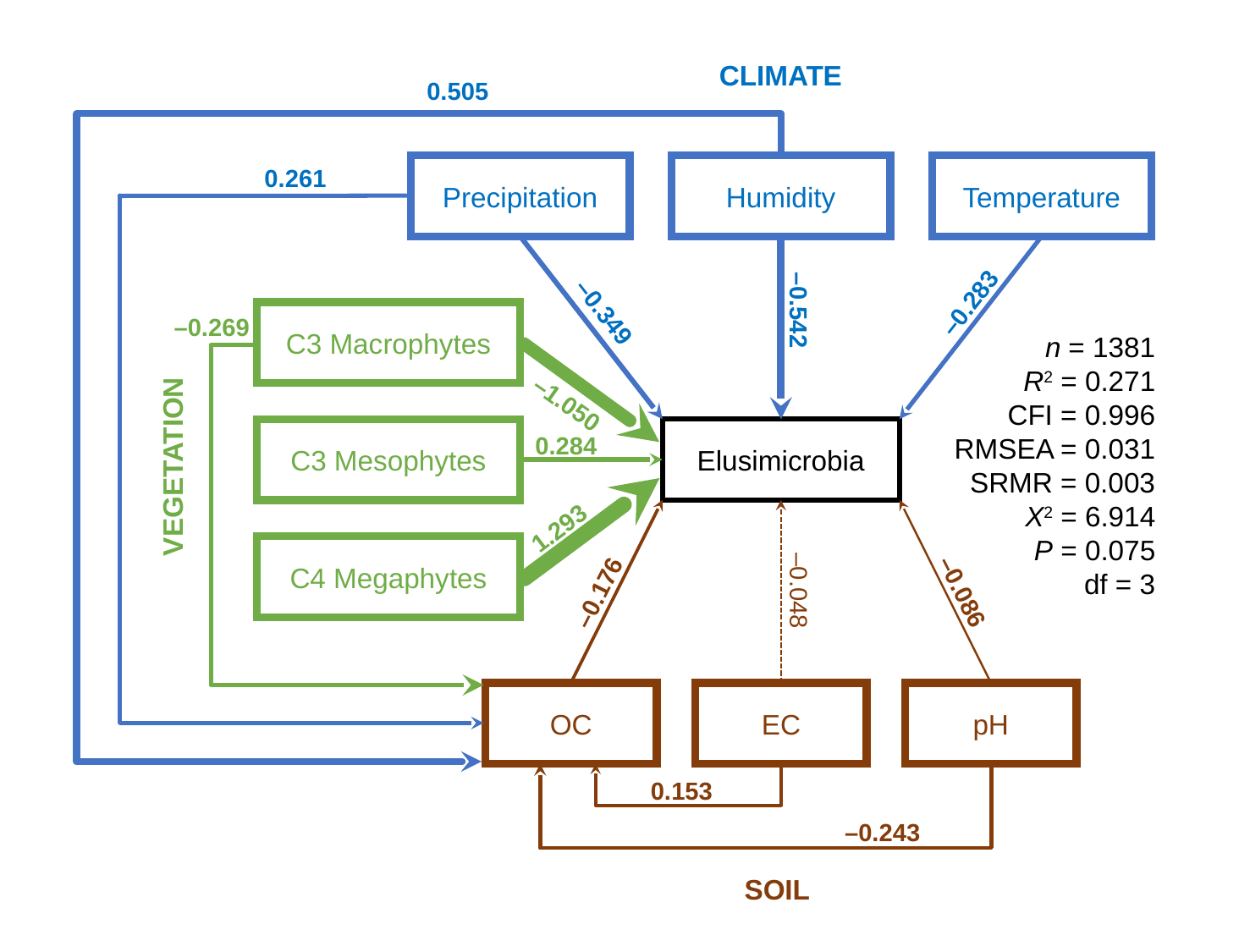

CLIMATE
0.505
Precipitation
Humidity
Temperature
0.261
–0.283
–0.542
–0.349
C3 Macrophytes
–0.269
n = 1381
R2 = 0.271
CFI = 0.996
RMSEA = 0.031
SRMR = 0.003
Χ2 = 6.914
P = 0.075
df = 3
–1.050
C3 Mesophytes
Elusimicrobia
0.284
VEGETATION
1.293
C4 Megaphytes
–0.048
–0.086
–0.176
OC
EC
pH
0.153
–0.243
SOIL

## Slide 16
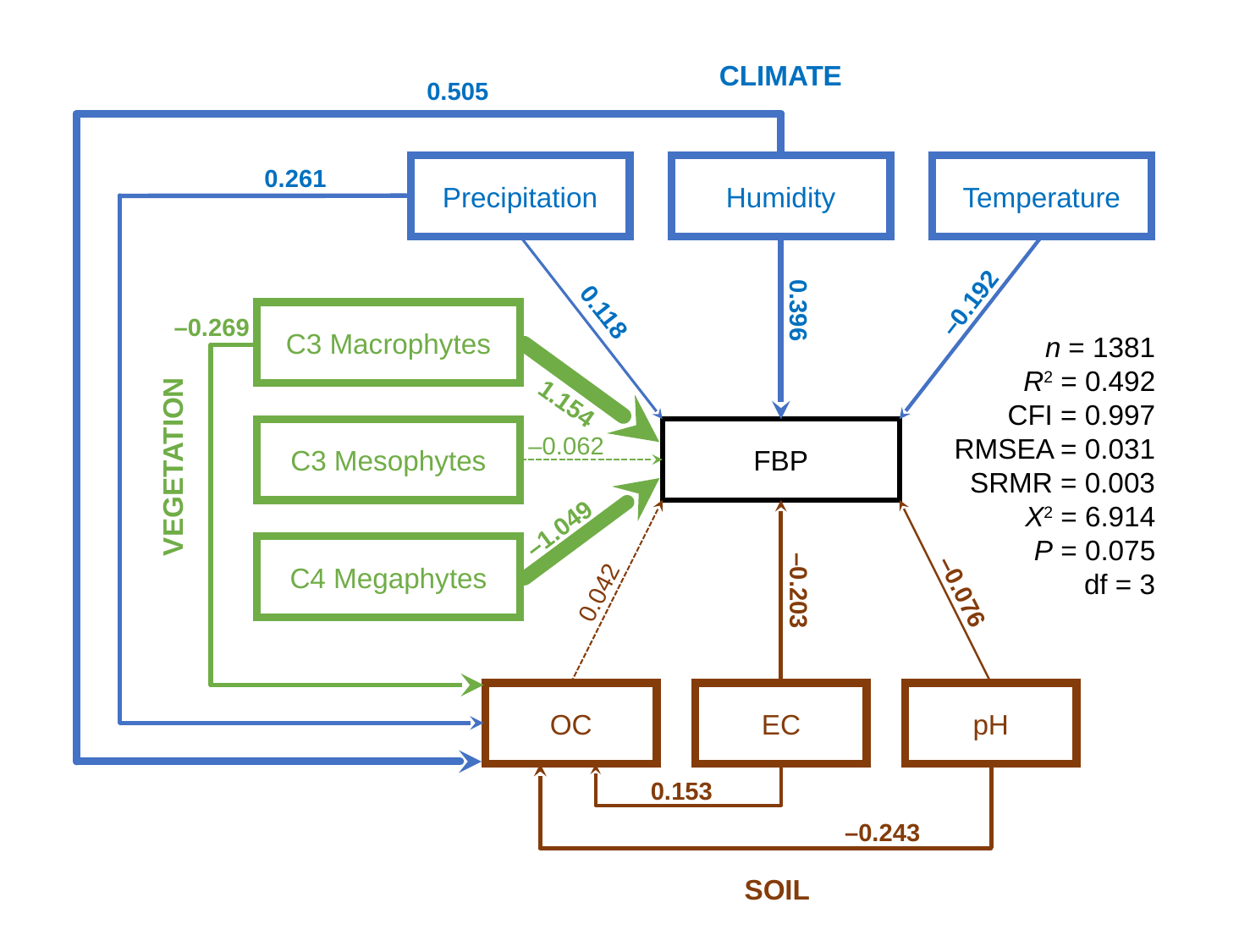

CLIMATE
0.505
Precipitation
Humidity
Temperature
0.261
–0.192
0.396
0.118
C3 Macrophytes
–0.269
n = 1381
R2 = 0.492
CFI = 0.997
RMSEA = 0.031
SRMR = 0.003
Χ2 = 6.914
P = 0.075
df = 3
1.154
C3 Mesophytes
FBP
–0.062
VEGETATION
–1.049
C4 Megaphytes
–0.203
–0.076
0.042
OC
EC
pH
0.153
–0.243
SOIL

## Slide 17
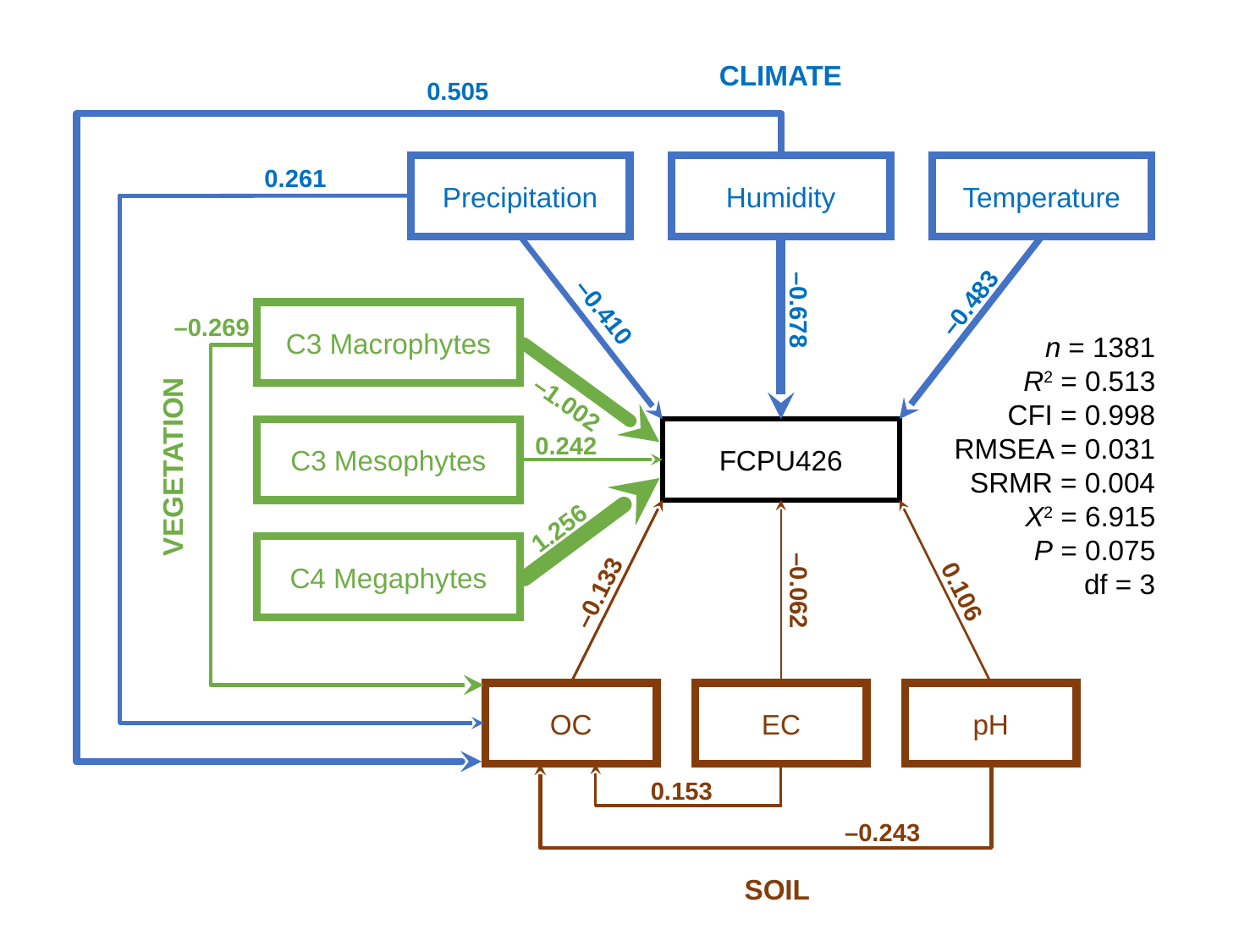

CLIMATE
0.505
Precipitation
Humidity
Temperature
0.261
–0.483
–0.678
–0.410
C3 Macrophytes
–0.269
n = 1381
R2 = 0.513
CFI = 0.998
RMSEA = 0.031
SRMR = 0.004
Χ2 = 6.915
P = 0.075
df = 3
–1.002
C3 Mesophytes
FCPU426
0.242
VEGETATION
1.256
C4 Megaphytes
–0.062
0.106
–0.133
OC
EC
pH
0.153
–0.243
SOIL

## Slide 18
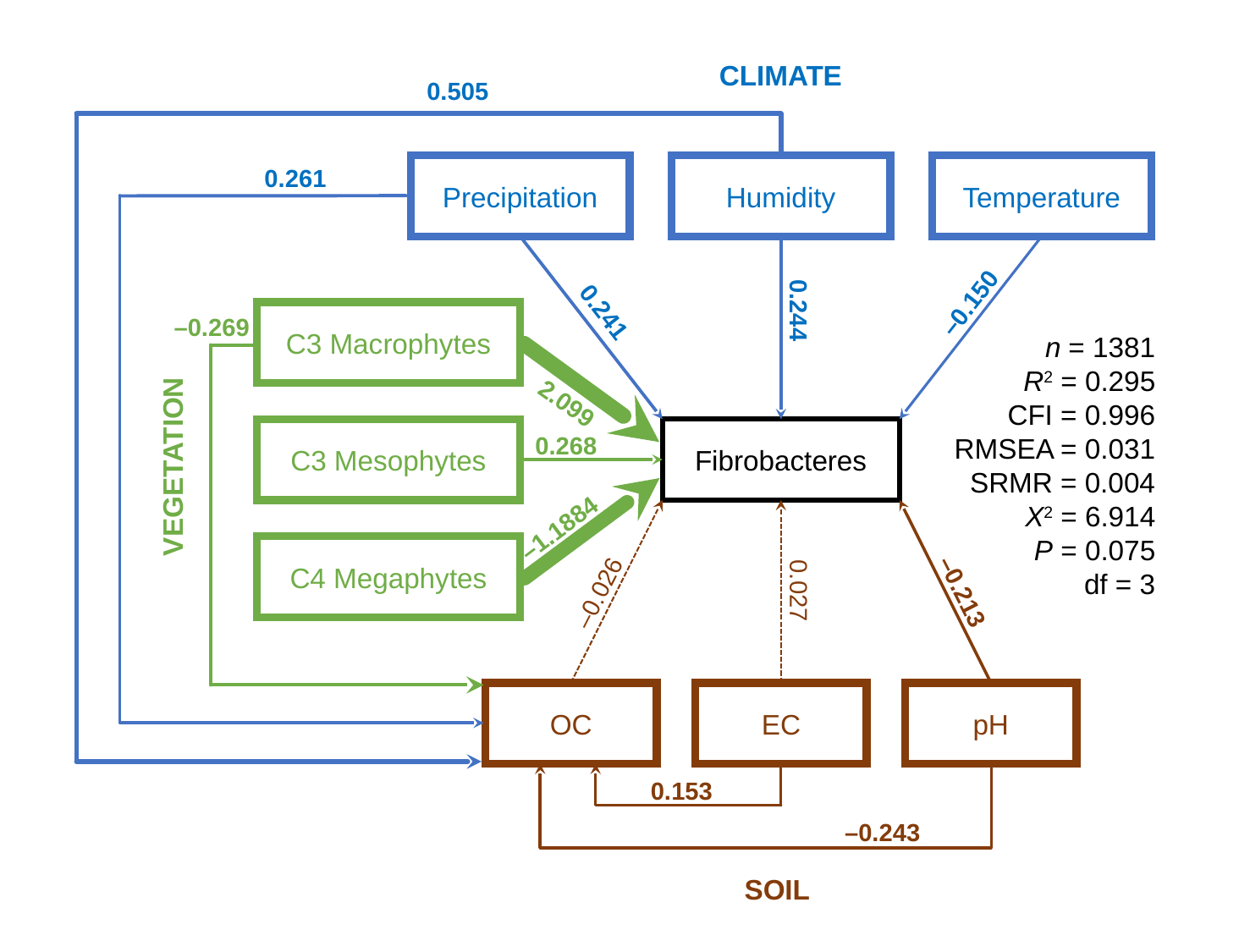

CLIMATE
0.505
Precipitation
Humidity
Temperature
0.261
–0.150
0.244
0.241
C3 Macrophytes
–0.269
n = 1381
R2 = 0.295
CFI = 0.996
RMSEA = 0.031
SRMR = 0.004
Χ2 = 6.914
P = 0.075
df = 3
2.099
C3 Mesophytes
Fibrobacteres
0.268
VEGETATION
–1.1884
C4 Megaphytes
0.027
–0.213
–0.026
OC
EC
pH
0.153
–0.243
SOIL

## Slide 19
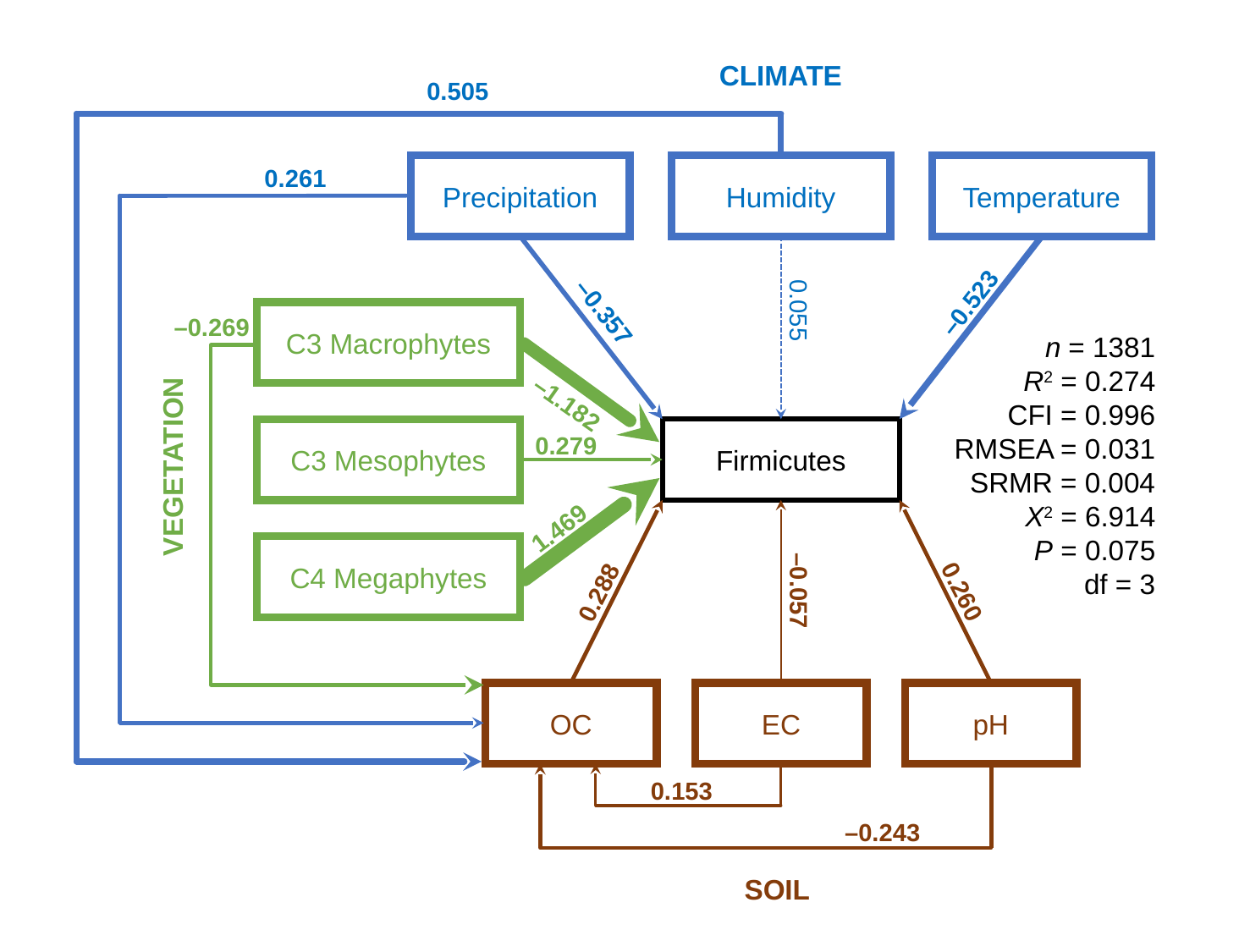

CLIMATE
0.505
Precipitation
Humidity
Temperature
0.261
–0.523
0.055
–0.357
C3 Macrophytes
–0.269
n = 1381
R2 = 0.274
CFI = 0.996
RMSEA = 0.031
SRMR = 0.004
Χ2 = 6.914
P = 0.075
df = 3
–1.182
C3 Mesophytes
Firmicutes
0.279
VEGETATION
1.469
C4 Megaphytes
–0.057
0.260
0.288
OC
EC
pH
0.153
–0.243
SOIL

## Slide 20
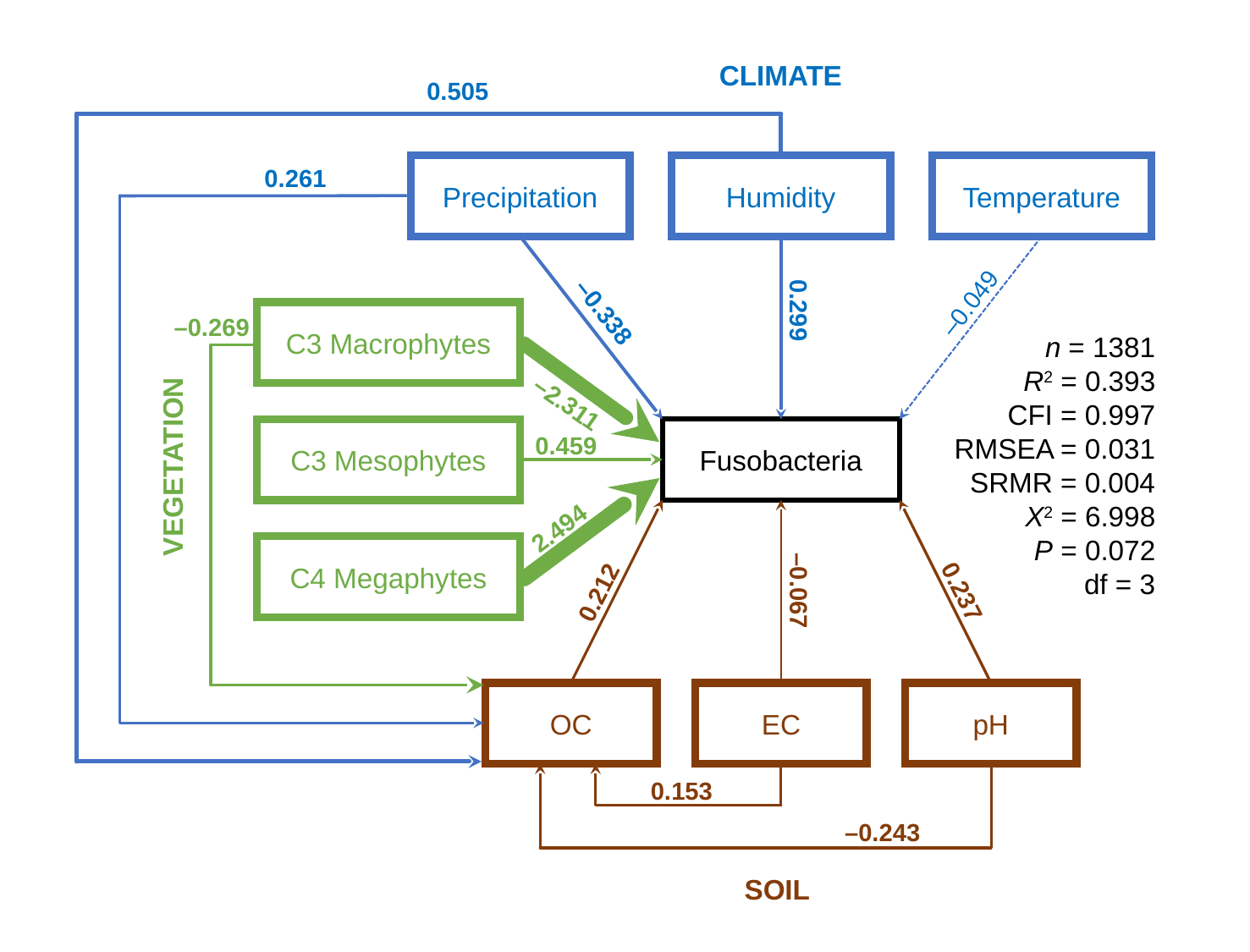

CLIMATE
0.505
Precipitation
Humidity
Temperature
0.261
–0.049
0.299
–0.338
C3 Macrophytes
–0.269
n = 1381
R2 = 0.393
CFI = 0.997
RMSEA = 0.031
SRMR = 0.004
Χ2 = 6.998
P = 0.072
df = 3
–2.311
C3 Mesophytes
Fusobacteria
0.459
VEGETATION
2.494
C4 Megaphytes
–0.067
0.237
0.212
OC
EC
pH
0.153
–0.243
SOIL

## Slide 21
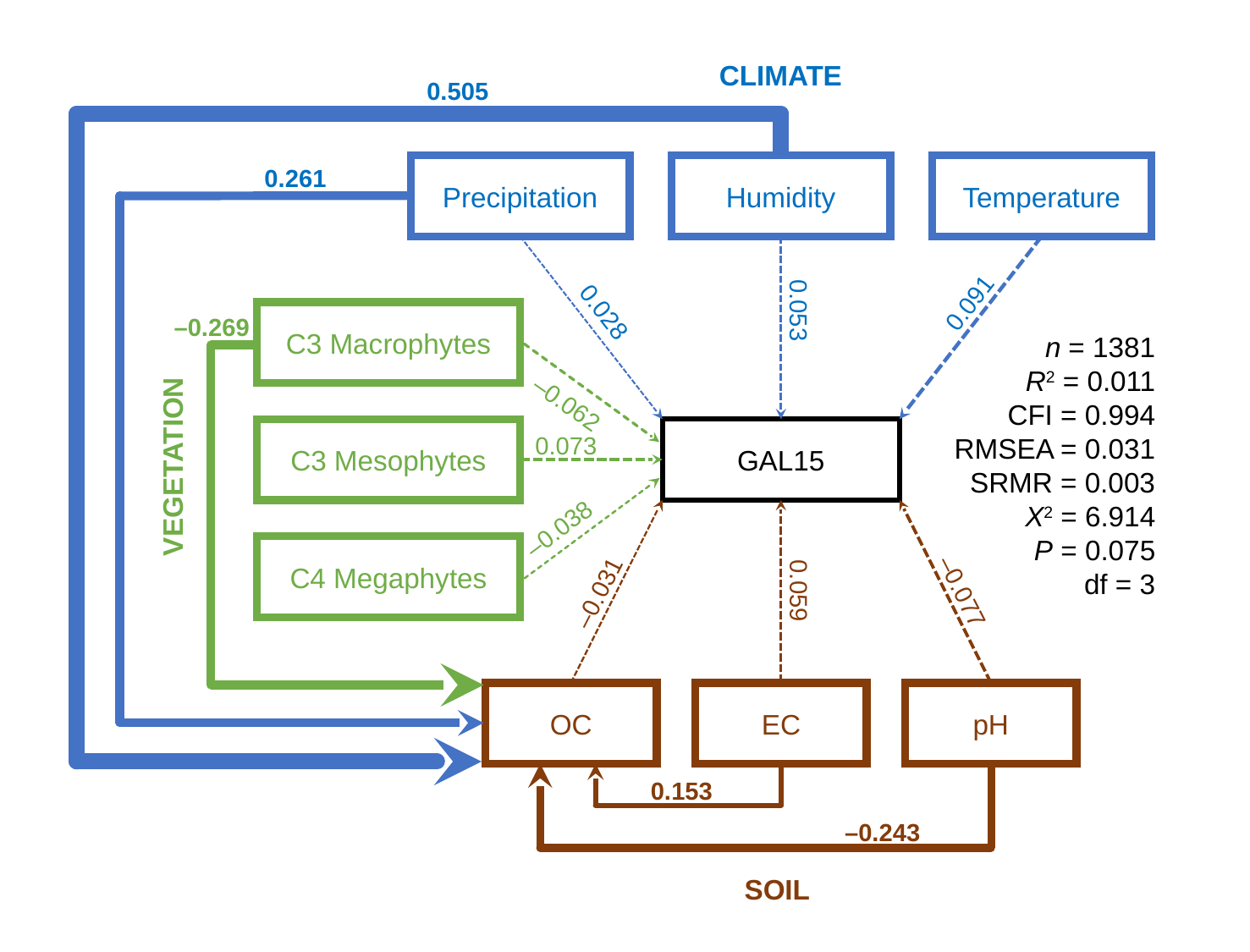

CLIMATE
0.505
Precipitation
Humidity
Temperature
0.261
0.091
0.053
0.028
C3 Macrophytes
–0.269
n = 1381
R2 = 0.011
CFI = 0.994
RMSEA = 0.031
SRMR = 0.003
Χ2 = 6.914
P = 0.075
df = 3
–0.062
C3 Mesophytes
GAL15
0.073
VEGETATION
–0.038
C4 Megaphytes
0.059
–0.077
–0.031
OC
EC
pH
0.153
–0.243
SOIL

## Slide 22
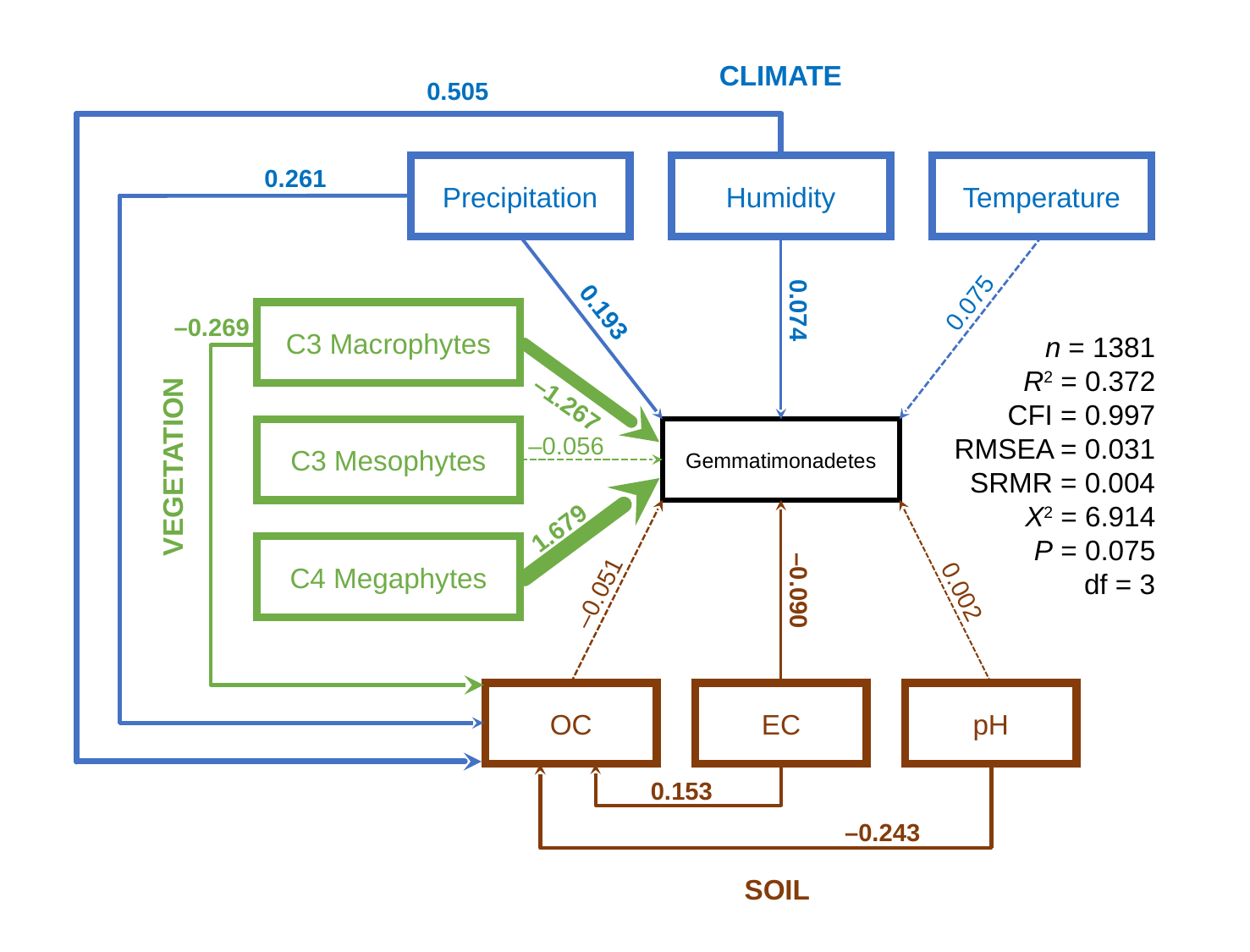

CLIMATE
0.505
Precipitation
Humidity
Temperature
0.261
0.075
0.074
0.193
C3 Macrophytes
–0.269
n = 1381
R2 = 0.372
CFI = 0.997
RMSEA = 0.031
SRMR = 0.004
Χ2 = 6.914
P = 0.075
df = 3
–1.267
C3 Mesophytes
Gemmatimonadetes
–0.056
VEGETATION
1.679
C4 Megaphytes
–0.090
0.002
–0.051
OC
EC
pH
0.153
–0.243
SOIL

## Slide 23
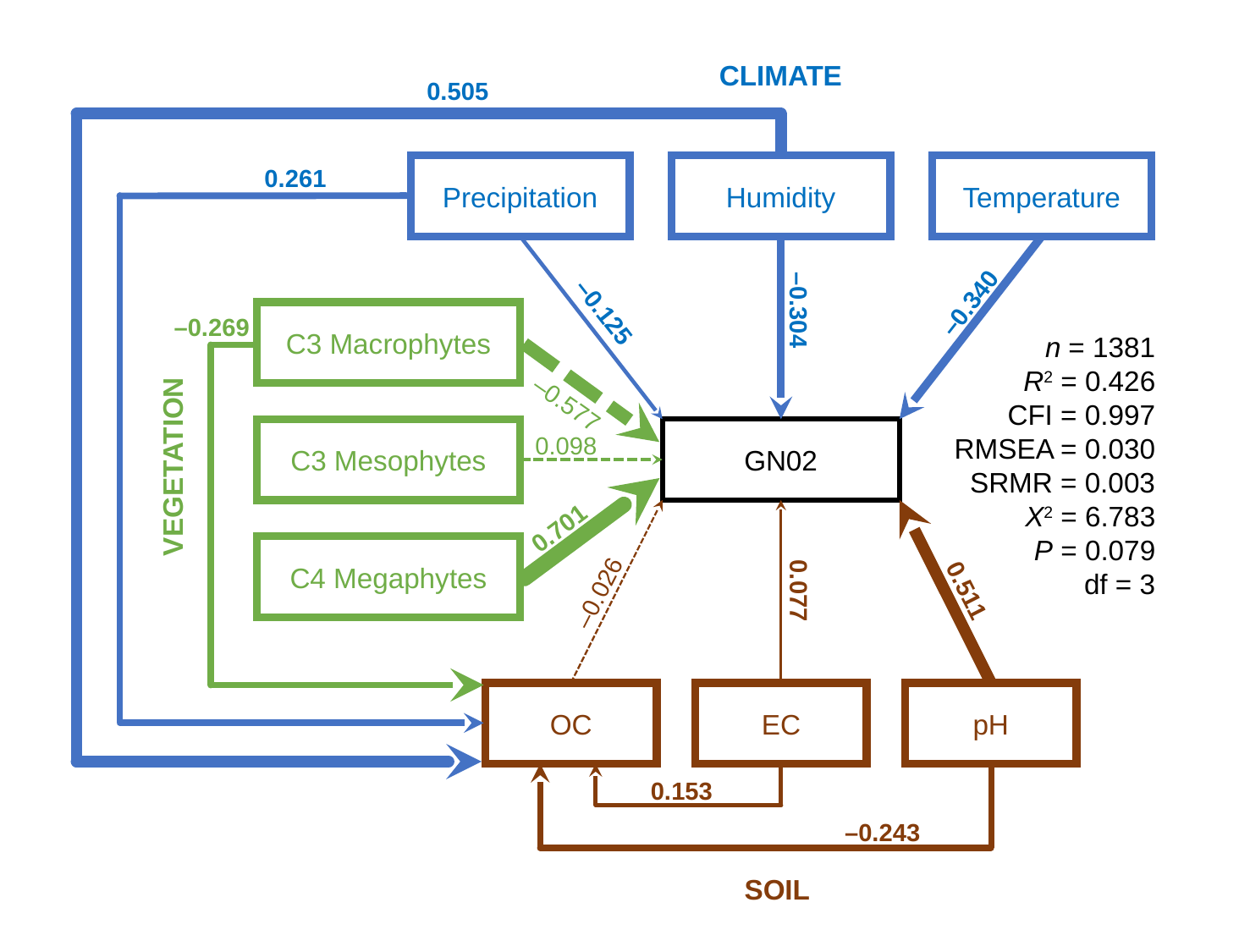

CLIMATE
0.505
Precipitation
Humidity
Temperature
0.261
–0.340
–0.304
–0.125
C3 Macrophytes
–0.269
n = 1381
R2 = 0.426
CFI = 0.997
RMSEA = 0.030
SRMR = 0.003
Χ2 = 6.783
P = 0.079
df = 3
–0.577
C3 Mesophytes
GN02
0.098
VEGETATION
0.701
C4 Megaphytes
0.511
0.077
–0.026
OC
EC
pH
0.153
–0.243
SOIL

## Slide 24
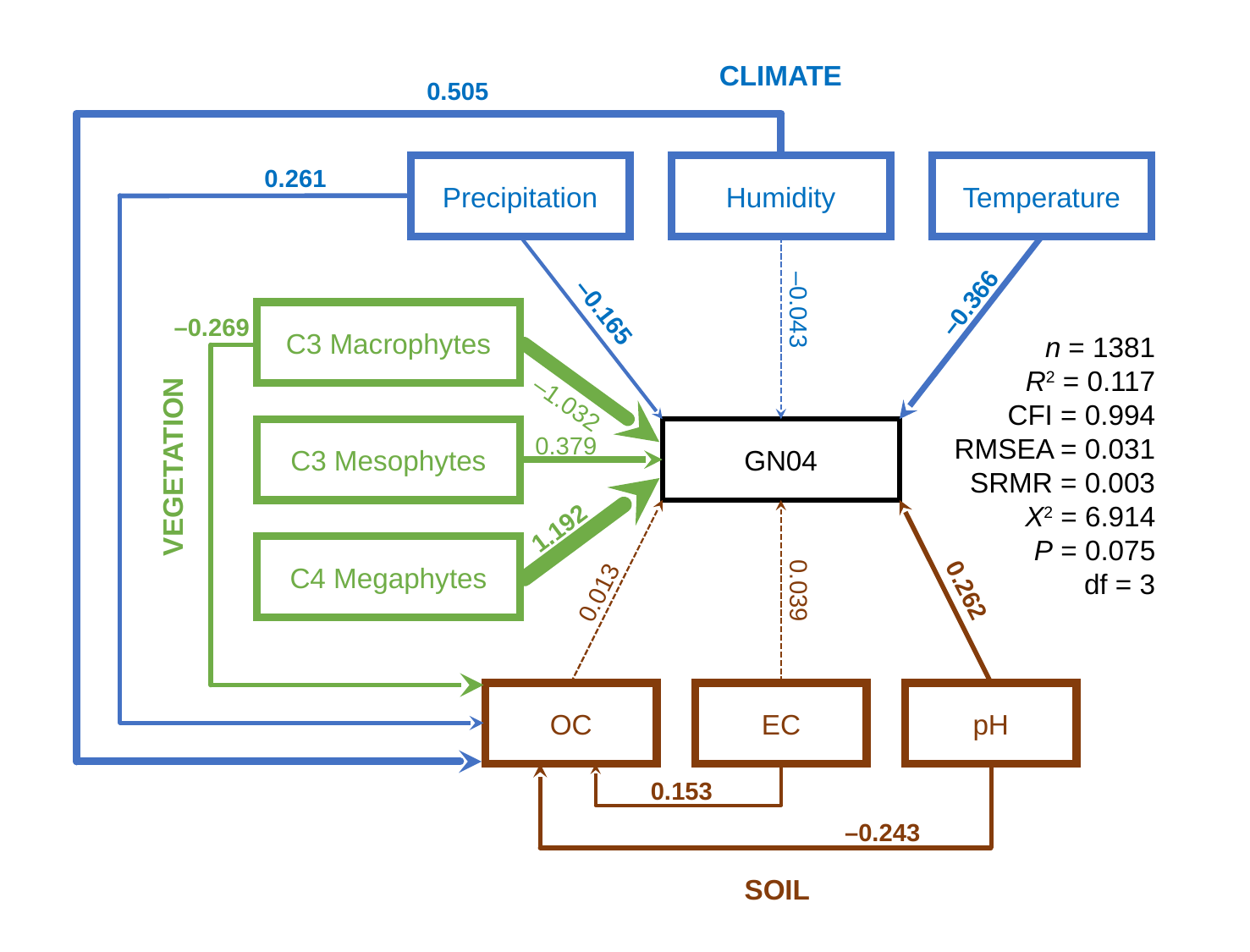

CLIMATE
0.505
Precipitation
Humidity
Temperature
0.261
–0.366
–0.043
–0.165
C3 Macrophytes
–0.269
n = 1381
R2 = 0.117
CFI = 0.994
RMSEA = 0.031
SRMR = 0.003
Χ2 = 6.914
P = 0.075
df = 3
–1.032
C3 Mesophytes
GN04
0.379
VEGETATION
1.192
C4 Megaphytes
0.262
0.039
0.013
OC
EC
pH
0.153
–0.243
SOIL

## Slide 25
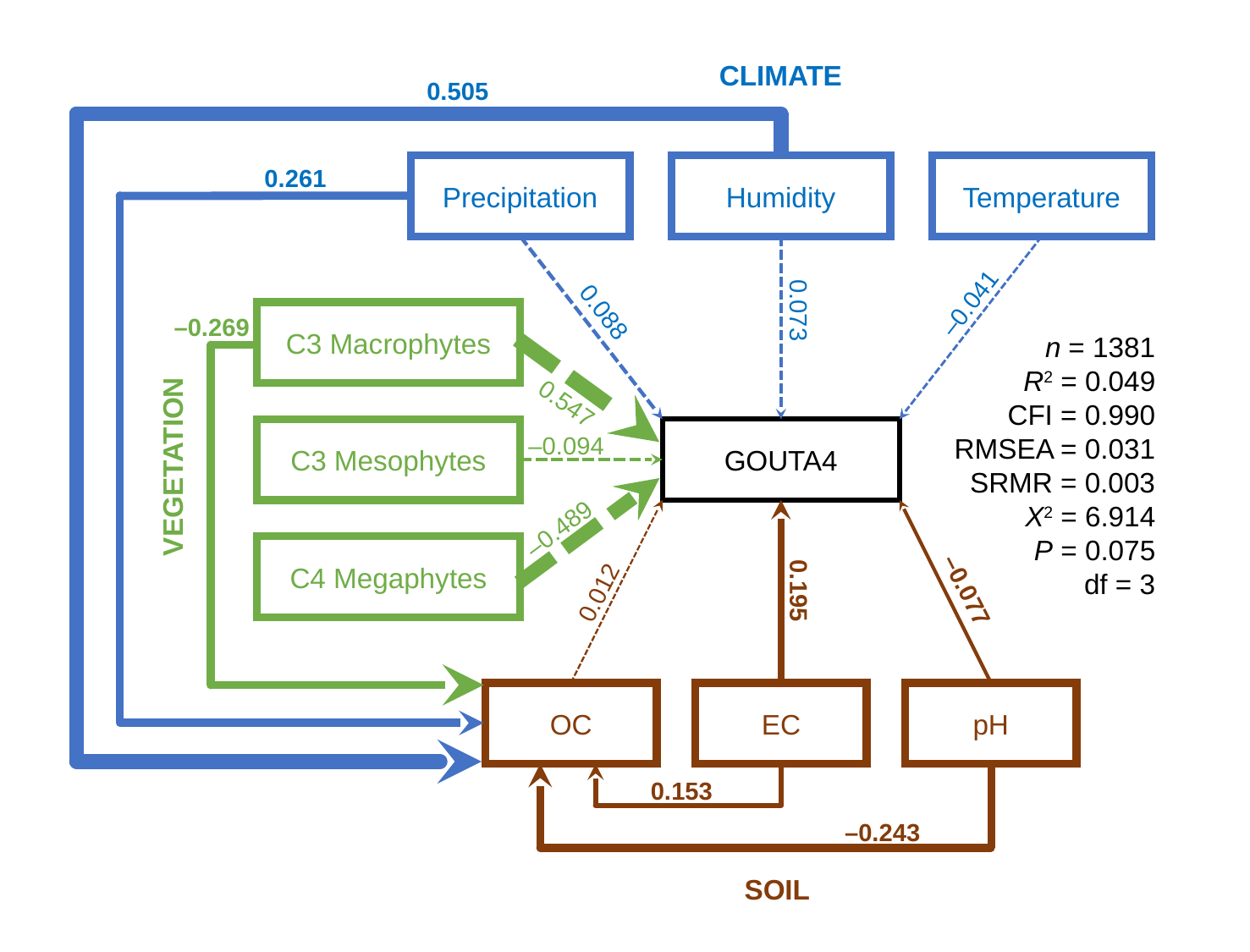

CLIMATE
0.505
Precipitation
Humidity
Temperature
0.261
–0.041
0.073
0.088
C3 Macrophytes
–0.269
n = 1381
R2 = 0.049
CFI = 0.990
RMSEA = 0.031
SRMR = 0.003
Χ2 = 6.914
P = 0.075
df = 3
0.547
C3 Mesophytes
GOUTA4
–0.094
VEGETATION
–0.489
C4 Megaphytes
–0.077
0.195
0.012
OC
EC
pH
0.153
–0.243
SOIL

## Slide 26
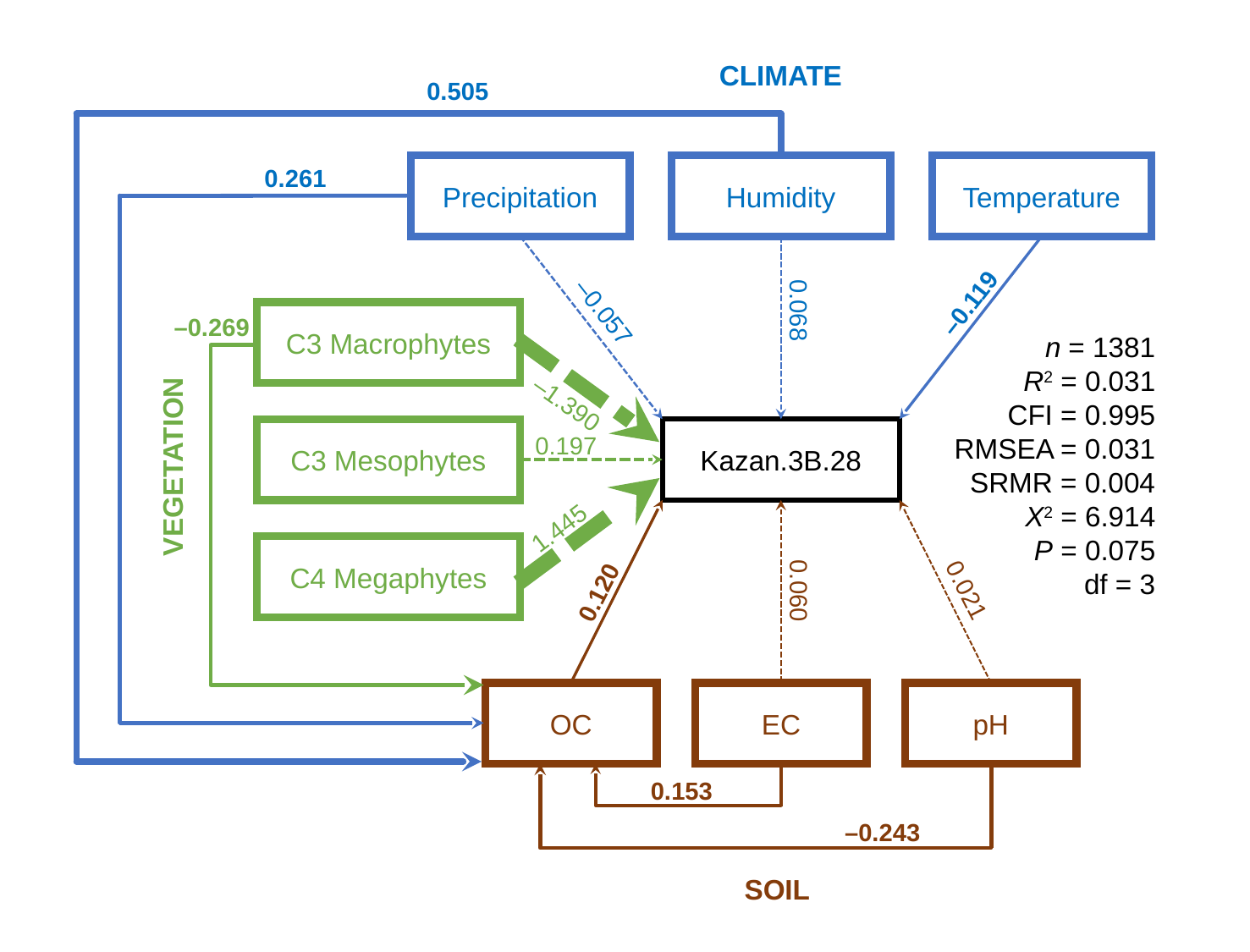

CLIMATE
0.505
Precipitation
Humidity
Temperature
0.261
–0.119
0.068
–0.057
C3 Macrophytes
–0.269
n = 1381
R2 = 0.031
CFI = 0.995
RMSEA = 0.031
SRMR = 0.004
Χ2 = 6.914
P = 0.075
df = 3
–1.390
C3 Mesophytes
Kazan.3B.28
0.197
VEGETATION
1.445
C4 Megaphytes
0.021
0.060
0.120
OC
EC
pH
0.153
–0.243
SOIL

## Slide 27
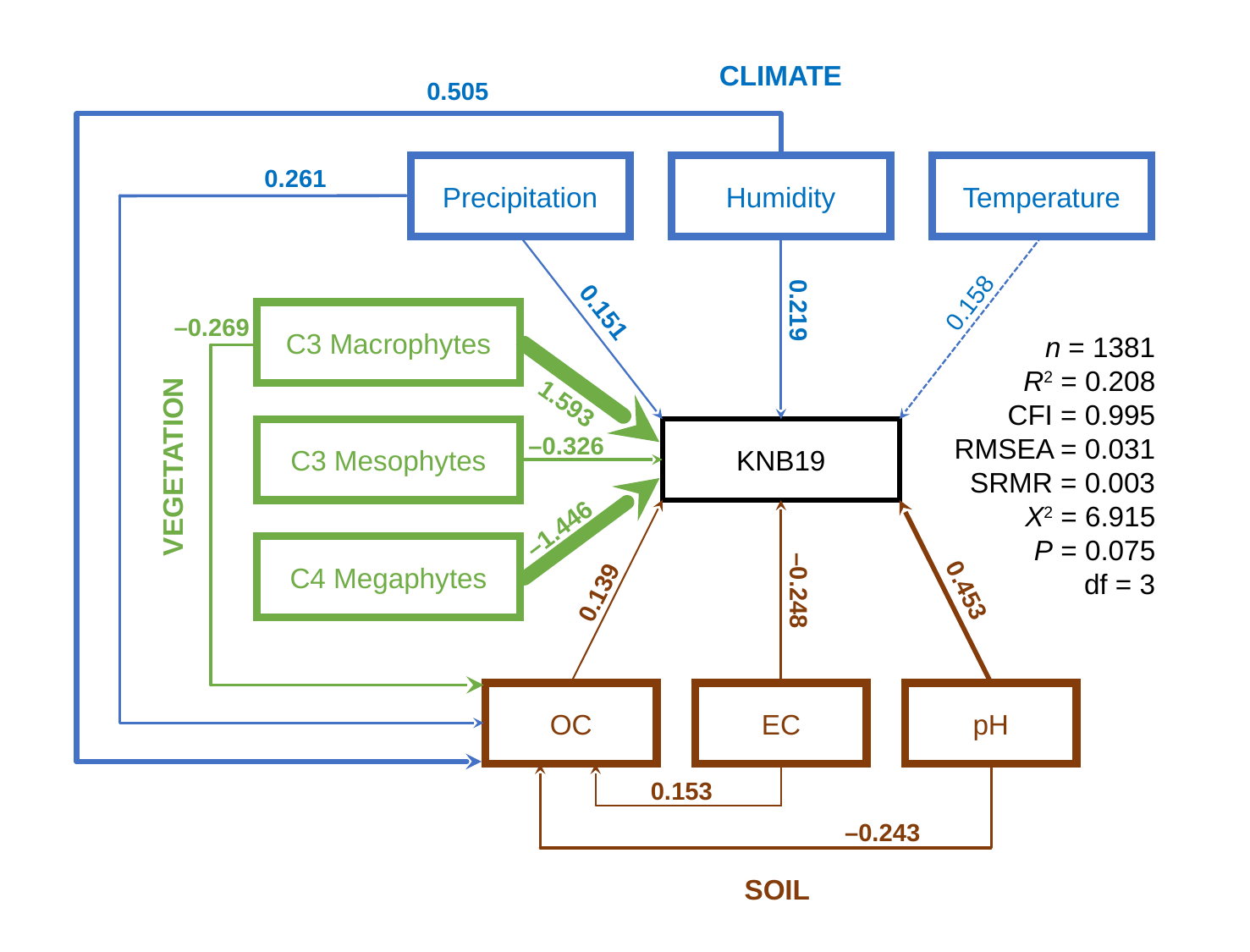

CLIMATE
0.505
Precipitation
Humidity
Temperature
0.261
0.158
0.219
0.151
C3 Macrophytes
–0.269
n = 1381
R2 = 0.208
CFI = 0.995
RMSEA = 0.031
SRMR = 0.003
Χ2 = 6.915
P = 0.075
df = 3
1.593
C3 Mesophytes
KNB19
–0.326
VEGETATION
–1.446
C4 Megaphytes
0.453
–0.248
0.139
OC
EC
pH
0.153
–0.243
SOIL

## Slide 28
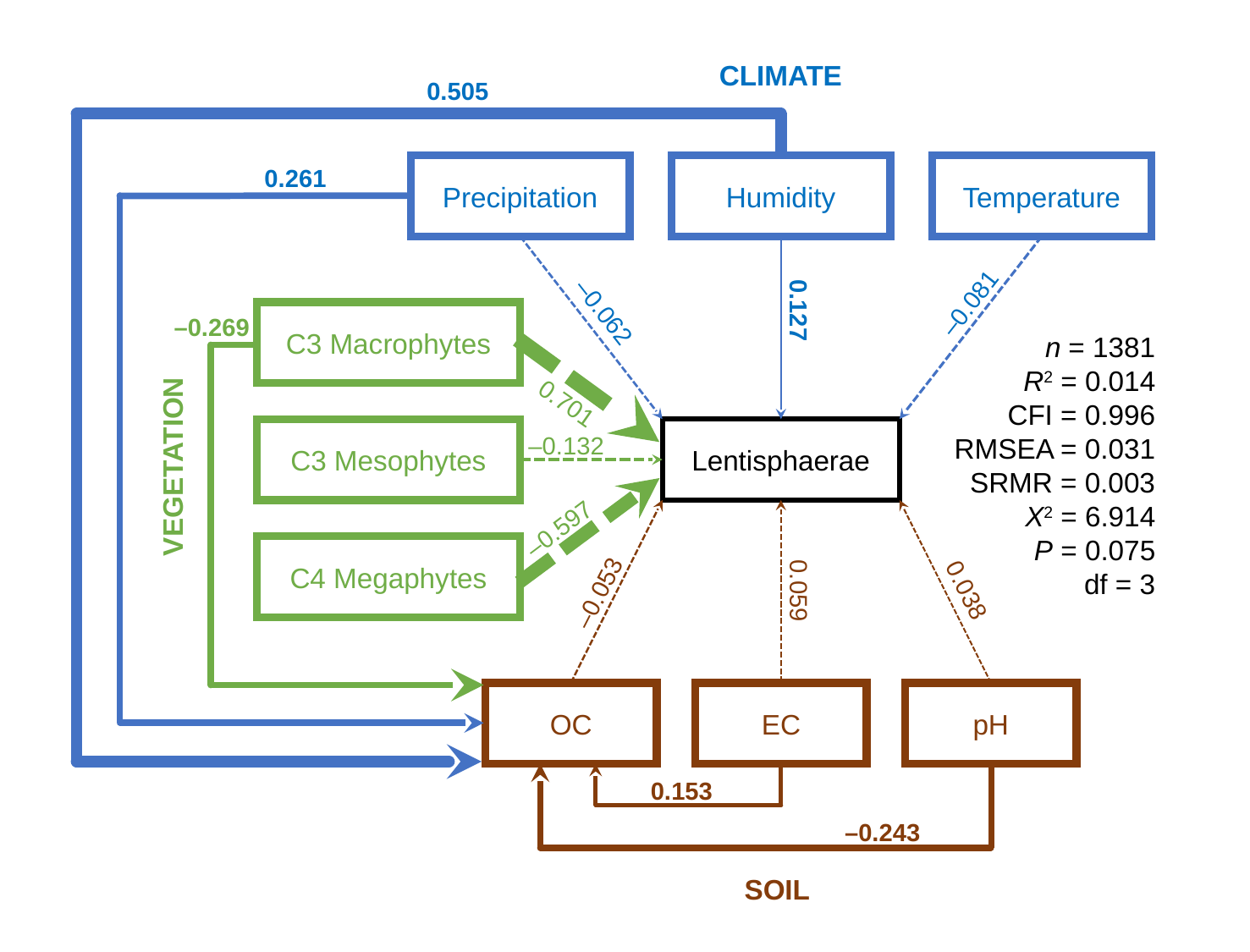

CLIMATE
0.505
Precipitation
Humidity
Temperature
0.261
–0.081
0.127
–0.062
C3 Macrophytes
–0.269
n = 1381
R2 = 0.014
CFI = 0.996
RMSEA = 0.031
SRMR = 0.003
Χ2 = 6.914
P = 0.075
df = 3
0.701
C3 Mesophytes
Lentisphaerae
–0.132
VEGETATION
–0.597
C4 Megaphytes
0.038
0.059
–0.053
OC
EC
pH
0.153
–0.243
SOIL

## Slide 29
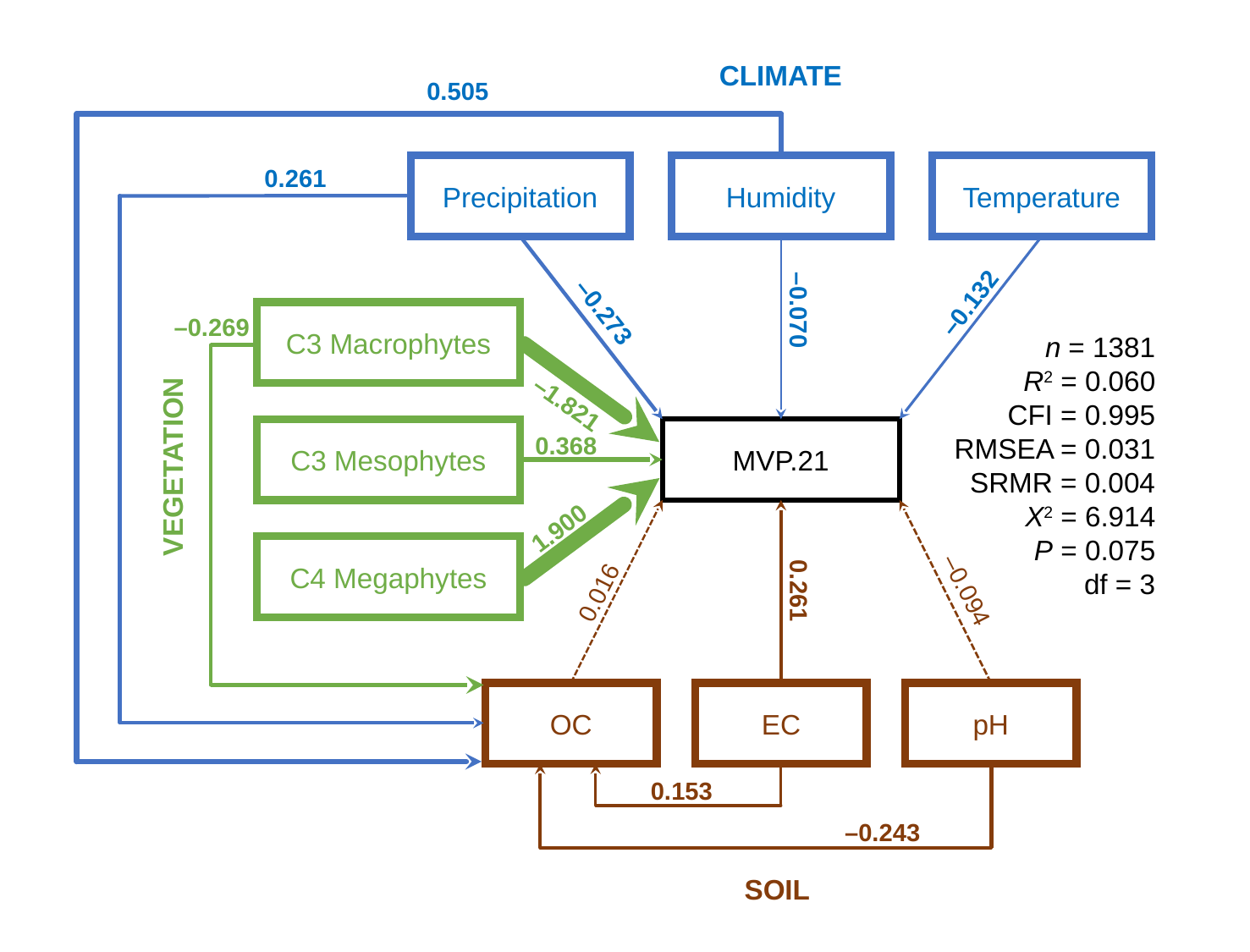

CLIMATE
0.505
Precipitation
Humidity
Temperature
0.261
–0.132
–0.070
–0.273
C3 Macrophytes
–0.269
n = 1381
R2 = 0.060
CFI = 0.995
RMSEA = 0.031
SRMR = 0.004
Χ2 = 6.914
P = 0.075
df = 3
–1.821
C3 Mesophytes
MVP.21
0.368
VEGETATION
1.900
C4 Megaphytes
–0.094
0.261
0.016
OC
EC
pH
0.153
–0.243
SOIL

## Slide 30
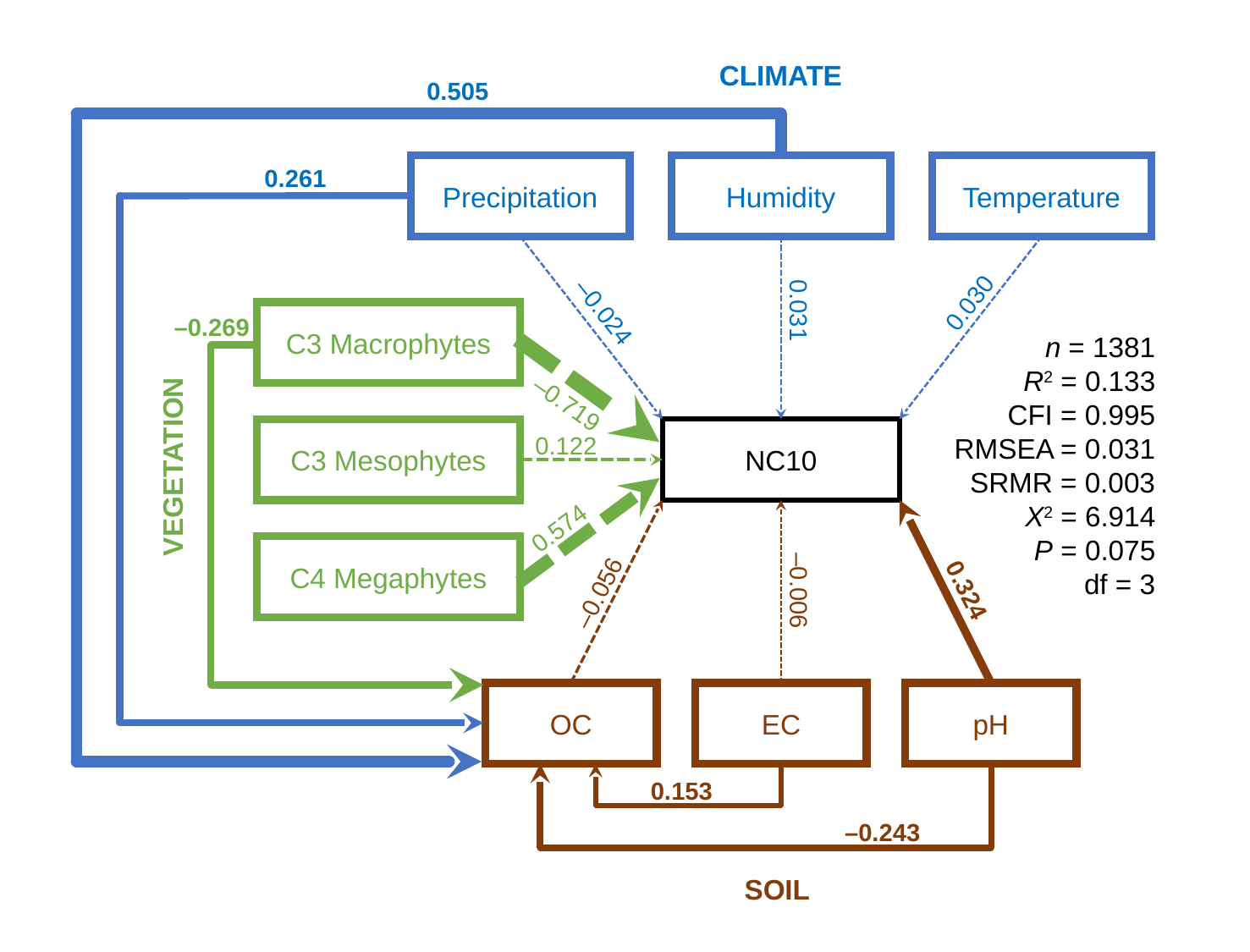

CLIMATE
0.505
Precipitation
Humidity
Temperature
0.261
0.030
0.031
–0.024
C3 Macrophytes
–0.269
n = 1381
R2 = 0.133
CFI = 0.995
RMSEA = 0.031
SRMR = 0.003
Χ2 = 6.914
P = 0.075
df = 3
–0.719
C3 Mesophytes
NC10
0.122
VEGETATION
0.574
C4 Megaphytes
0.324
–0.006
–0.056
OC
EC
pH
0.153
–0.243
SOIL

## Slide 31
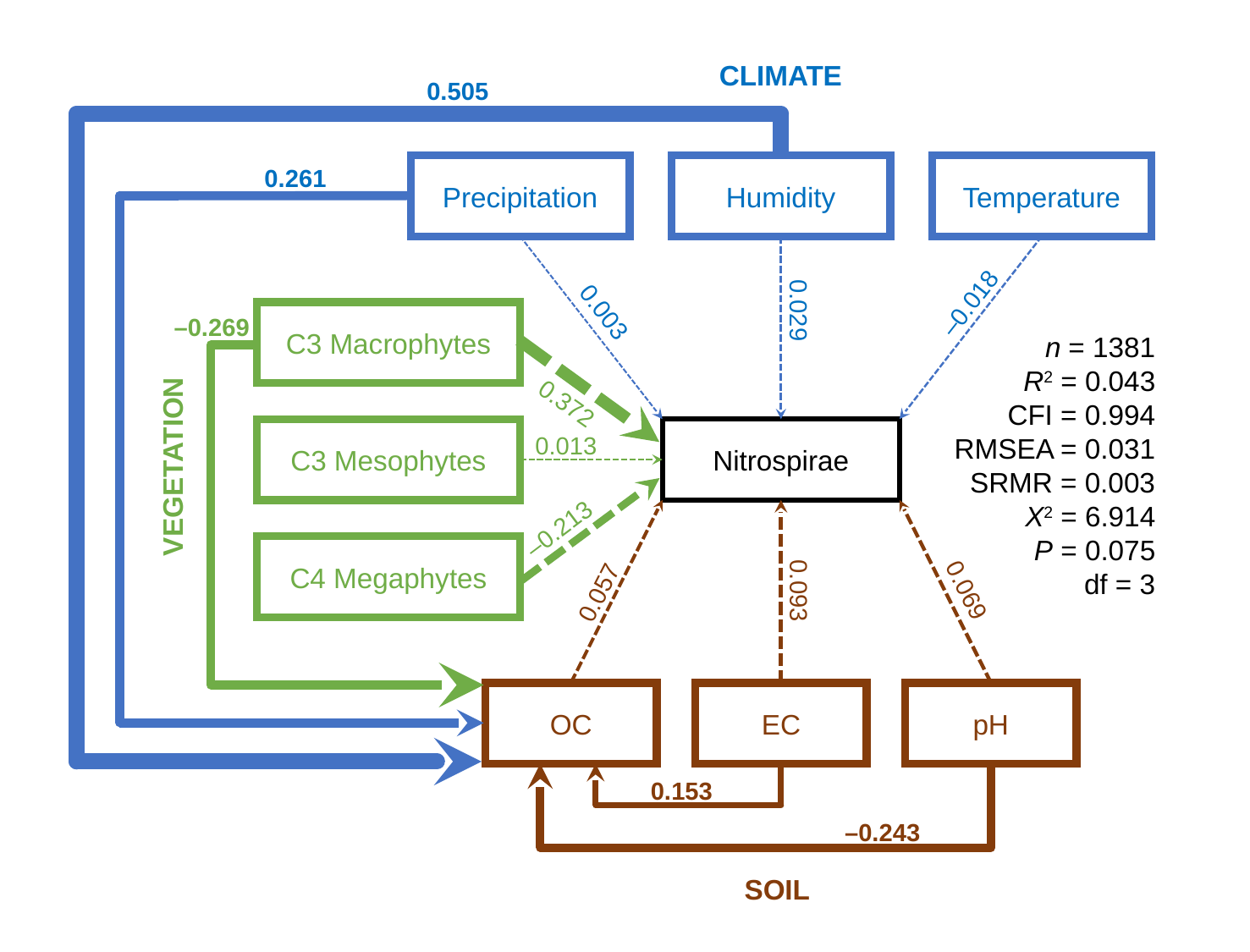

CLIMATE
0.505
Precipitation
Humidity
Temperature
0.261
–0.018
0.029
0.003
C3 Macrophytes
–0.269
n = 1381
R2 = 0.043
CFI = 0.994
RMSEA = 0.031
SRMR = 0.003
Χ2 = 6.914
P = 0.075
df = 3
0.372
C3 Mesophytes
Nitrospirae
0.013
VEGETATION
–0.213
C4 Megaphytes
0.069
0.093
0.057
OC
EC
pH
0.153
–0.243
SOIL

## Slide 32
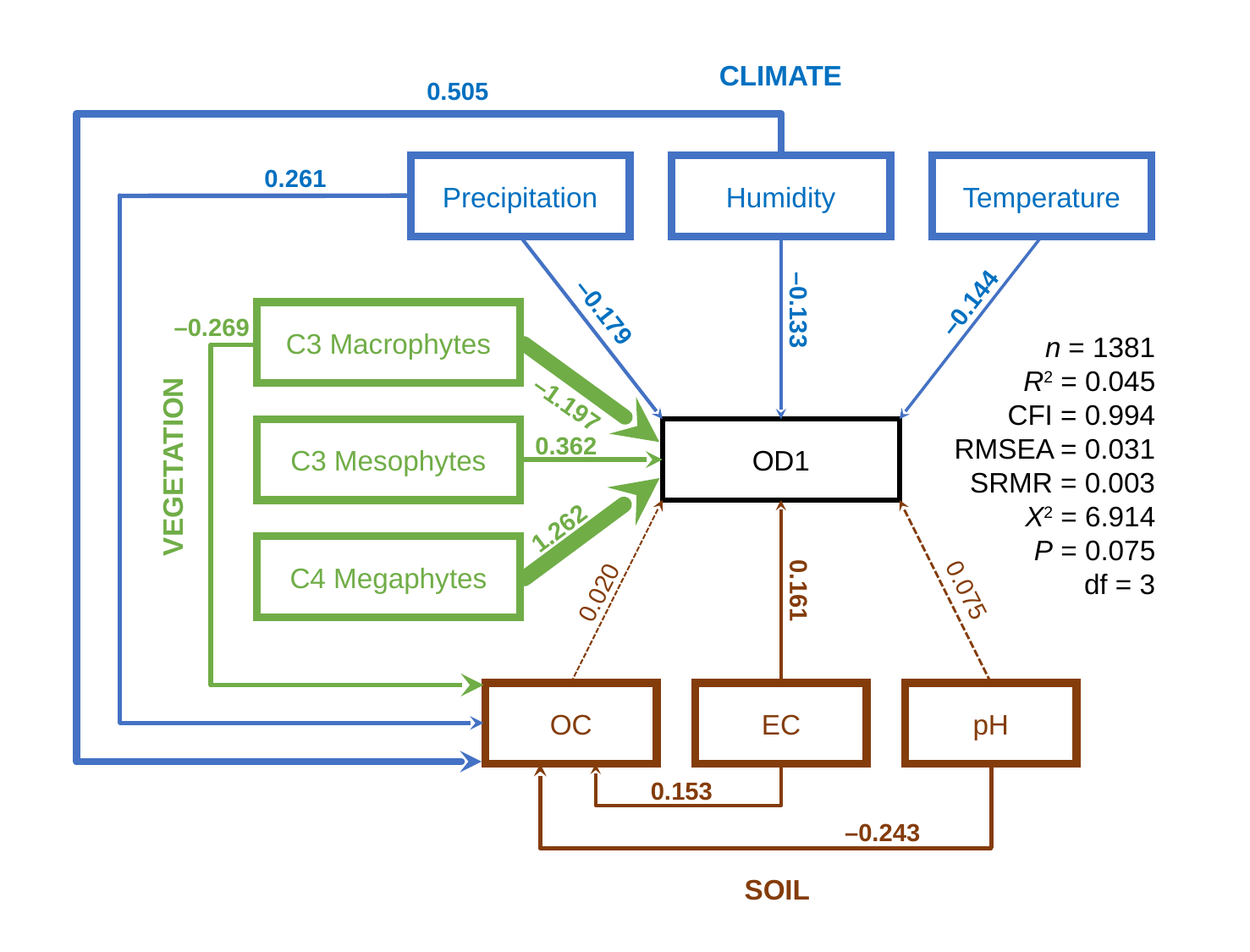

CLIMATE
0.505
Precipitation
Humidity
Temperature
0.261
–0.144
–0.133
–0.179
C3 Macrophytes
–0.269
n = 1381
R2 = 0.045
CFI = 0.994
RMSEA = 0.031
SRMR = 0.003
Χ2 = 6.914
P = 0.075
df = 3
–1.197
C3 Mesophytes
OD1
0.362
VEGETATION
1.262
C4 Megaphytes
0.075
0.161
0.020
OC
EC
pH
0.153
–0.243
SOIL

## Slide 33
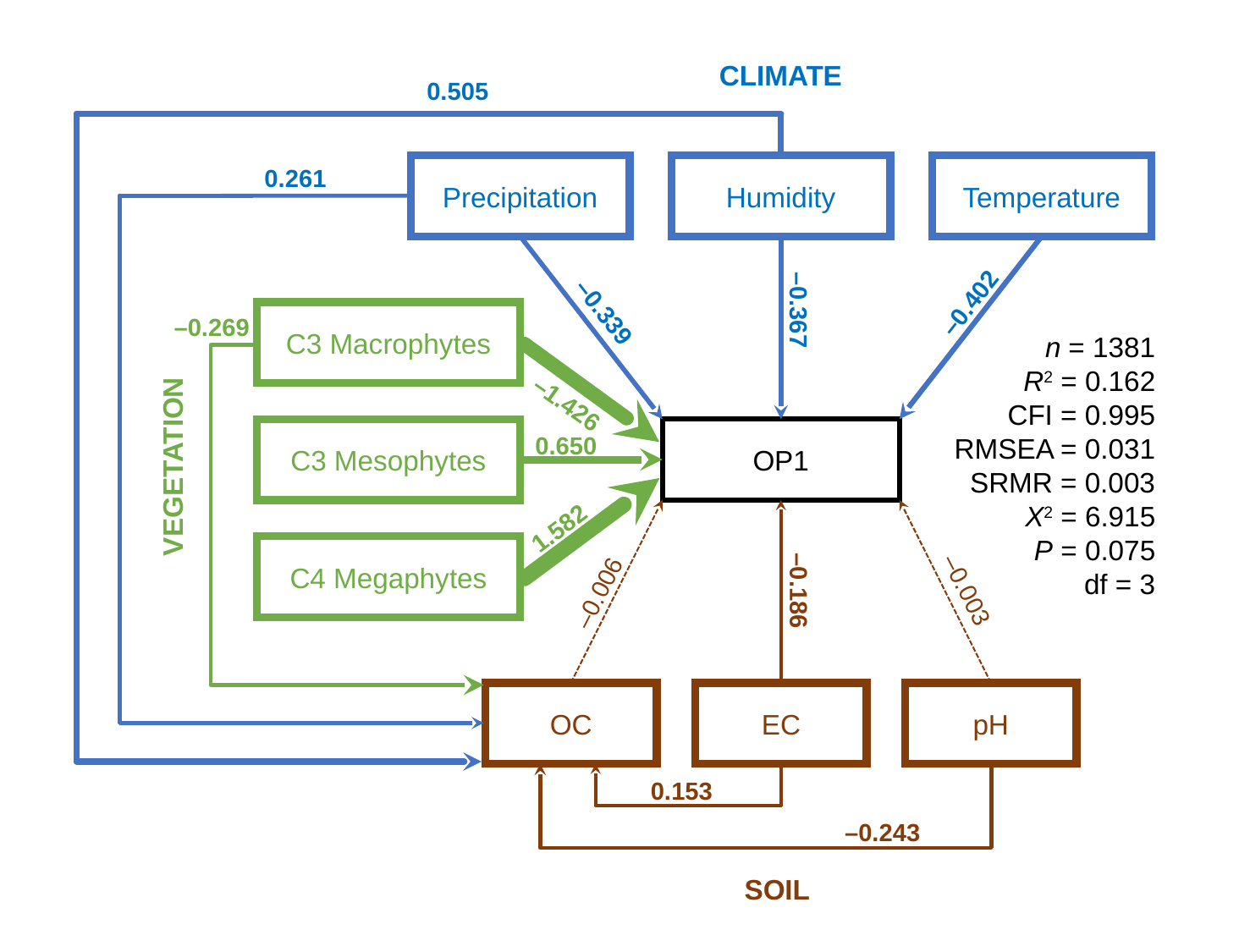

CLIMATE
0.505
Precipitation
Humidity
Temperature
0.261
–0.402
–0.367
–0.339
C3 Macrophytes
–0.269
n = 1381
R2 = 0.162
CFI = 0.995
RMSEA = 0.031
SRMR = 0.003
Χ2 = 6.915
P = 0.075
df = 3
–1.426
C3 Mesophytes
OP1
0.650
VEGETATION
1.582
C4 Megaphytes
–0.003
–0.186
–0.006
OC
EC
pH
0.153
–0.243
SOIL

## Slide 34
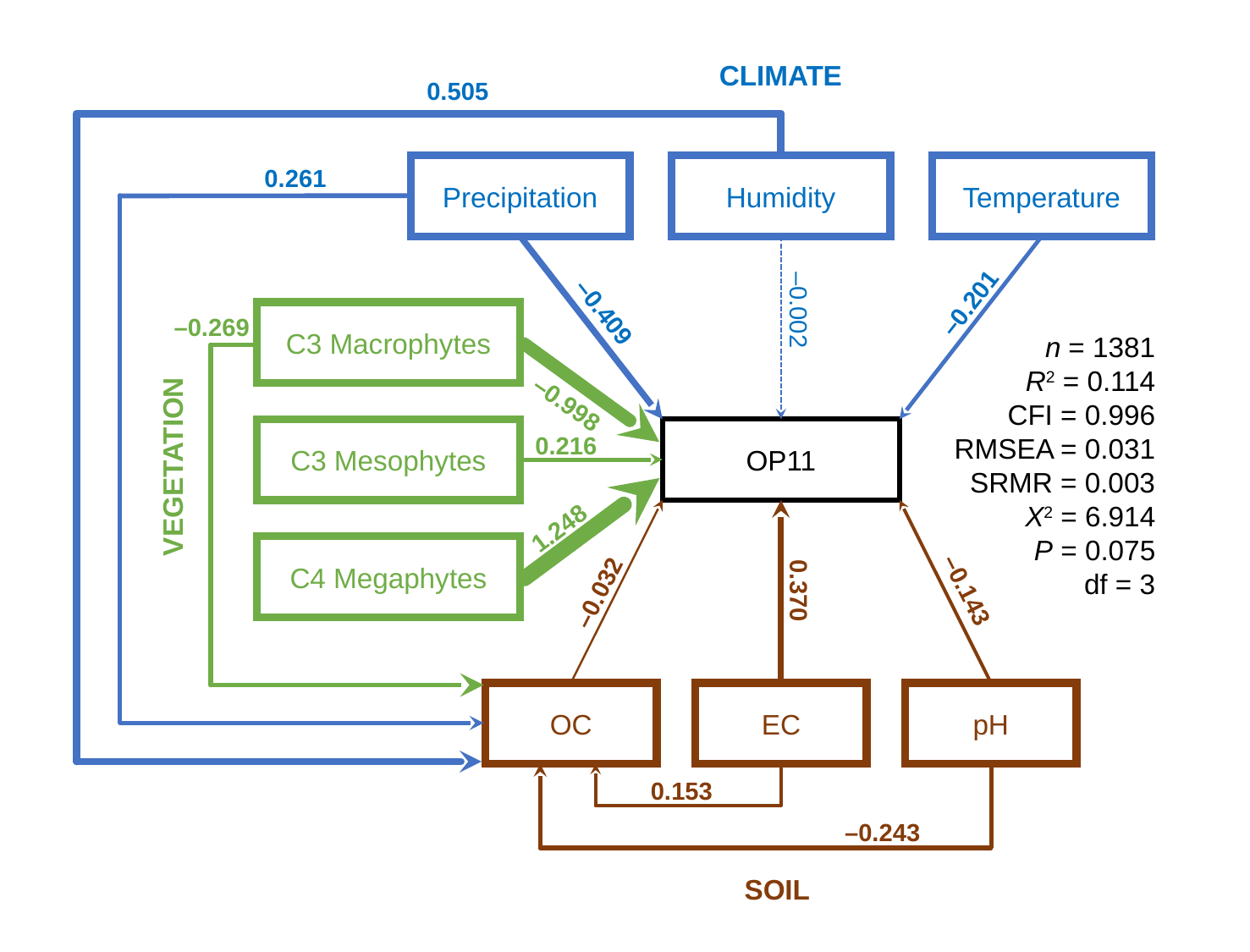

CLIMATE
0.505
Precipitation
Humidity
Temperature
0.261
–0.201
–0.002
–0.409
C3 Macrophytes
–0.269
n = 1381
R2 = 0.114
CFI = 0.996
RMSEA = 0.031
SRMR = 0.003
Χ2 = 6.914
P = 0.075
df = 3
–0.998
C3 Mesophytes
OP11
0.216
VEGETATION
1.248
C4 Megaphytes
–0.143
0.370
–0.032
OC
EC
pH
0.153
–0.243
SOIL

## Slide 35
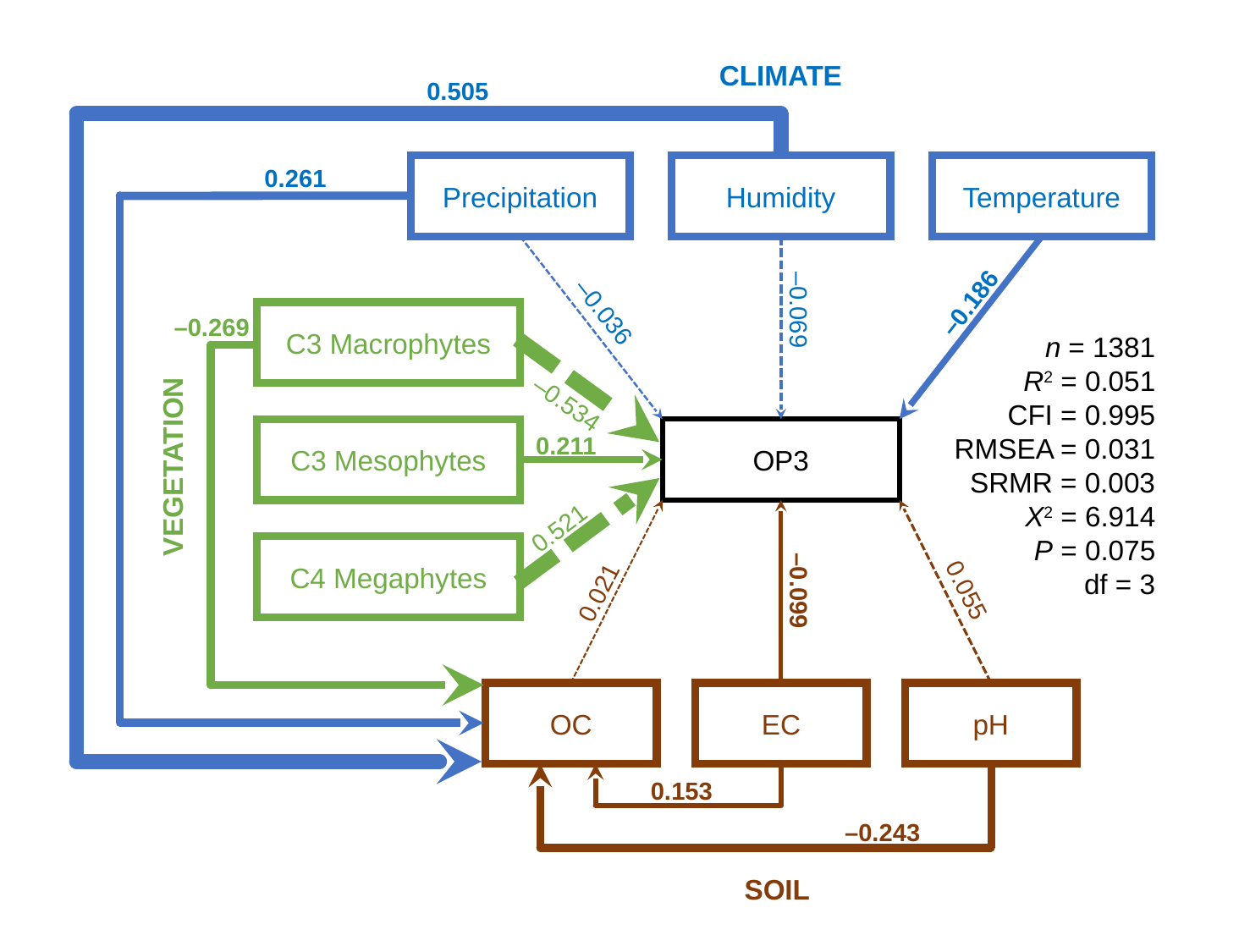

CLIMATE
0.505
Precipitation
Humidity
Temperature
0.261
–0.186
–0.069
–0.036
C3 Macrophytes
–0.269
n = 1381
R2 = 0.051
CFI = 0.995
RMSEA = 0.031
SRMR = 0.003
Χ2 = 6.914
P = 0.075
df = 3
–0.534
C3 Mesophytes
OP3
0.211
VEGETATION
0.521
C4 Megaphytes
0.055
–0.099
0.021
OC
EC
pH
0.153
–0.243
SOIL

## Slide 36
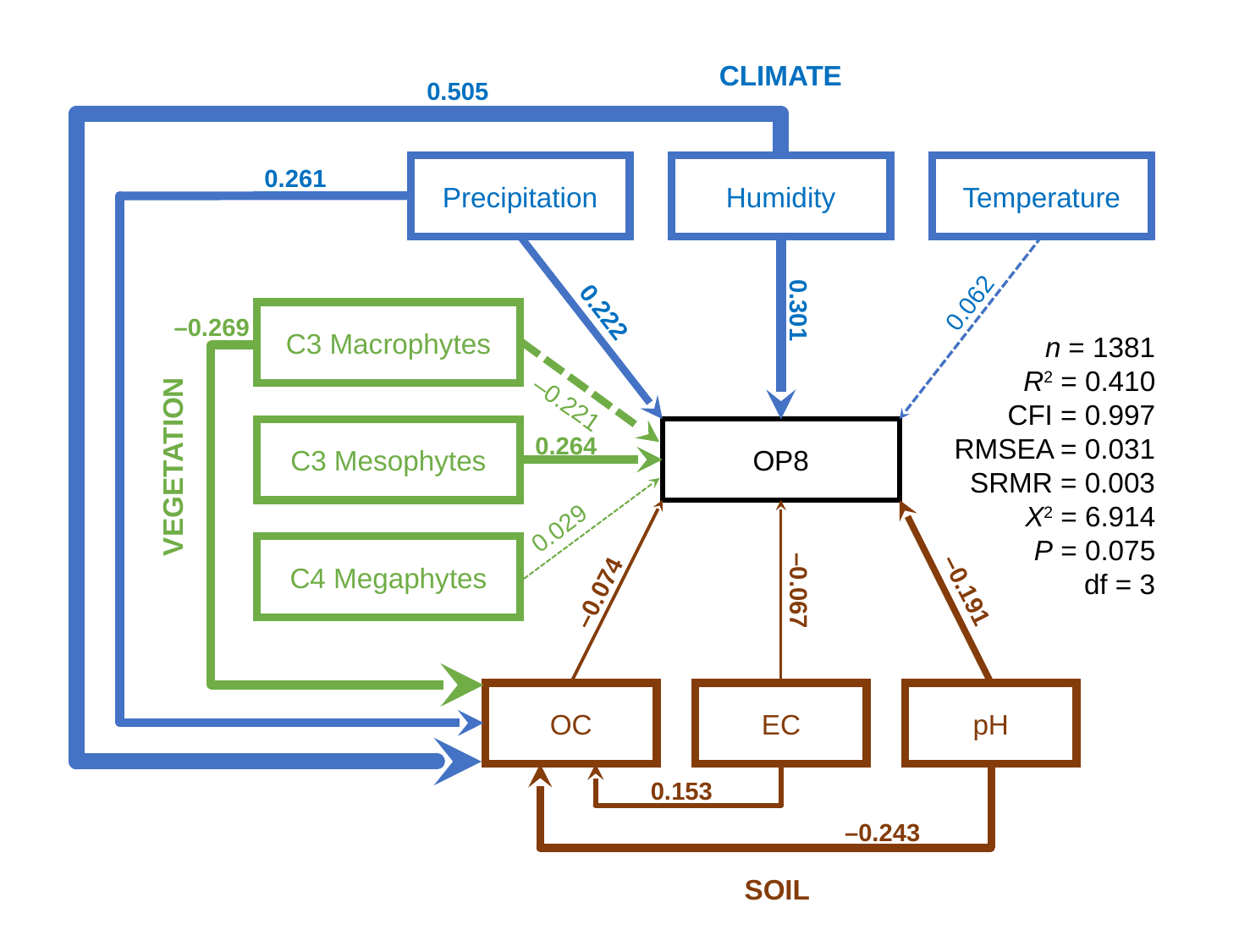

CLIMATE
0.505
Precipitation
Humidity
Temperature
0.261
0.062
0.301
0.222
C3 Macrophytes
–0.269
n = 1381
R2 = 0.410
CFI = 0.997
RMSEA = 0.031
SRMR = 0.003
Χ2 = 6.914
P = 0.075
df = 3
–0.221
C3 Mesophytes
OP8
0.264
VEGETATION
0.029
C4 Megaphytes
–0.191
–0.067
–0.074
OC
EC
pH
0.153
–0.243
SOIL

## Slide 37
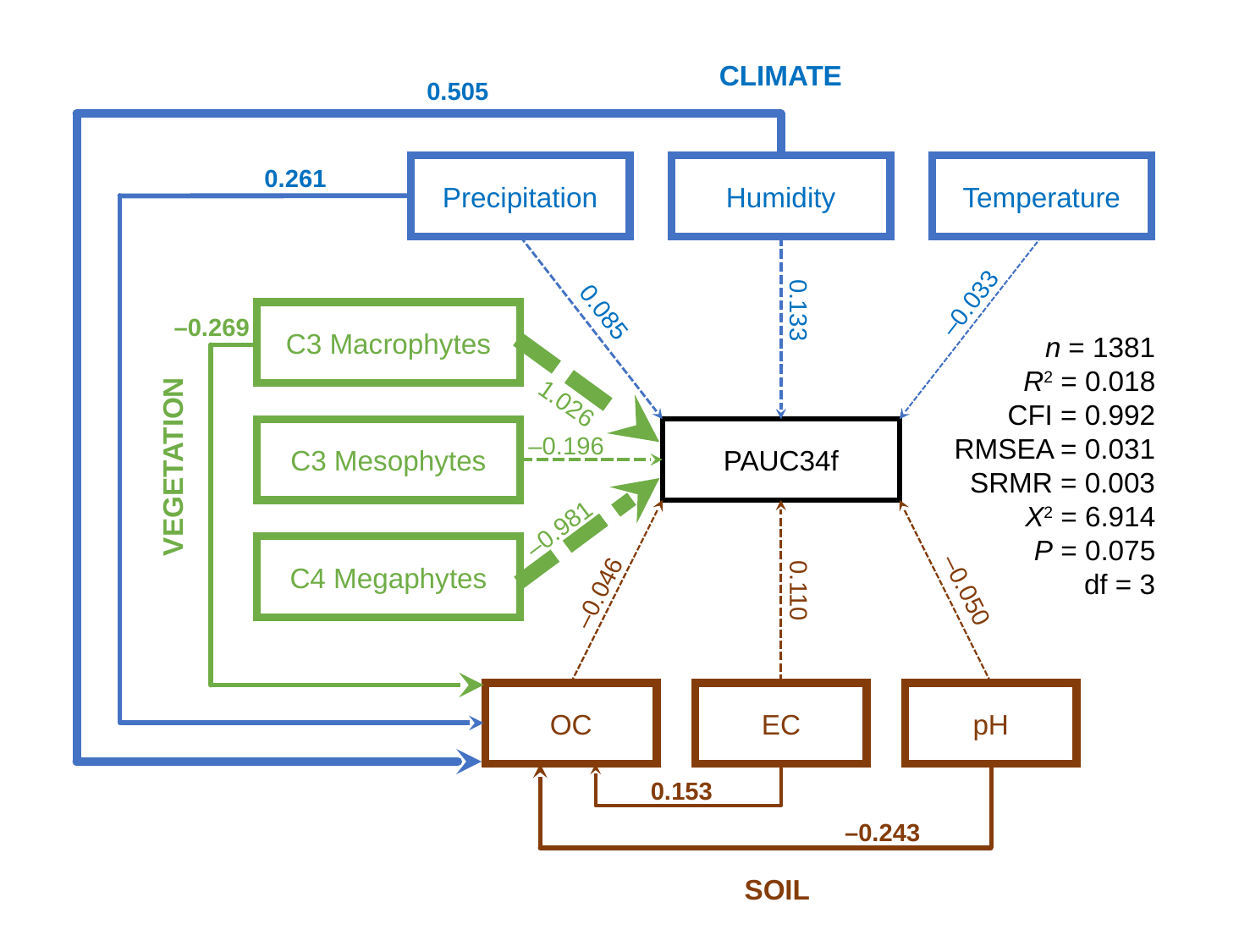

CLIMATE
0.505
Precipitation
Humidity
Temperature
0.261
–0.033
0.133
0.085
C3 Macrophytes
–0.269
n = 1381
R2 = 0.018
CFI = 0.992
RMSEA = 0.031
SRMR = 0.003
Χ2 = 6.914
P = 0.075
df = 3
1.026
C3 Mesophytes
PAUC34f
–0.196
VEGETATION
–0.981
C4 Megaphytes
–0.050
0.110
–0.046
OC
EC
pH
0.153
–0.243
SOIL

## Slide 38
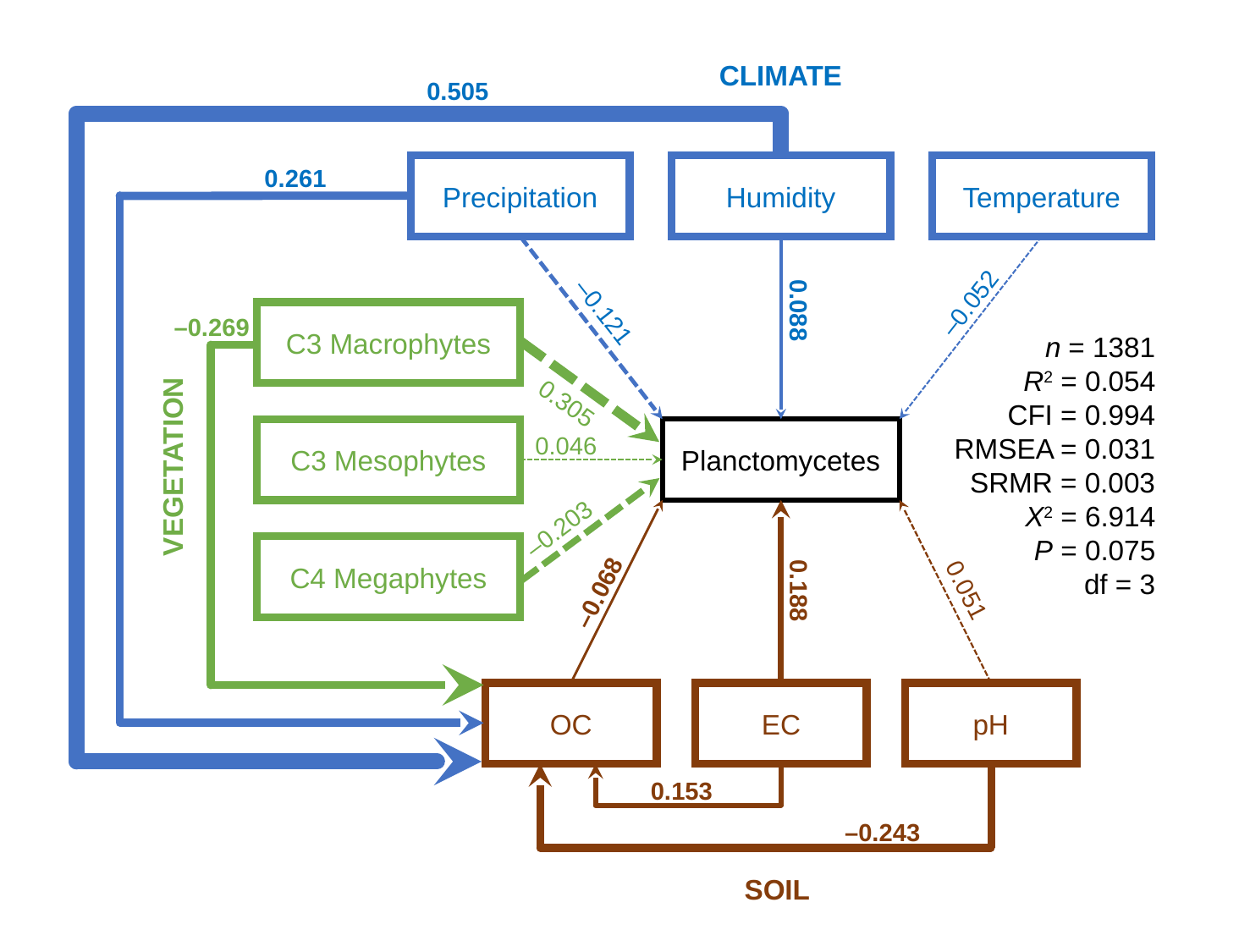

CLIMATE
0.505
Precipitation
Humidity
Temperature
0.261
–0.052
0.088
–0.121
C3 Macrophytes
–0.269
n = 1381
R2 = 0.054
CFI = 0.994
RMSEA = 0.031
SRMR = 0.003
Χ2 = 6.914
P = 0.075
df = 3
0.305
C3 Mesophytes
Planctomycetes
0.046
VEGETATION
–0.203
C4 Megaphytes
0.051
0.188
–0.068
OC
EC
pH
0.153
–0.243
SOIL

## Slide 39
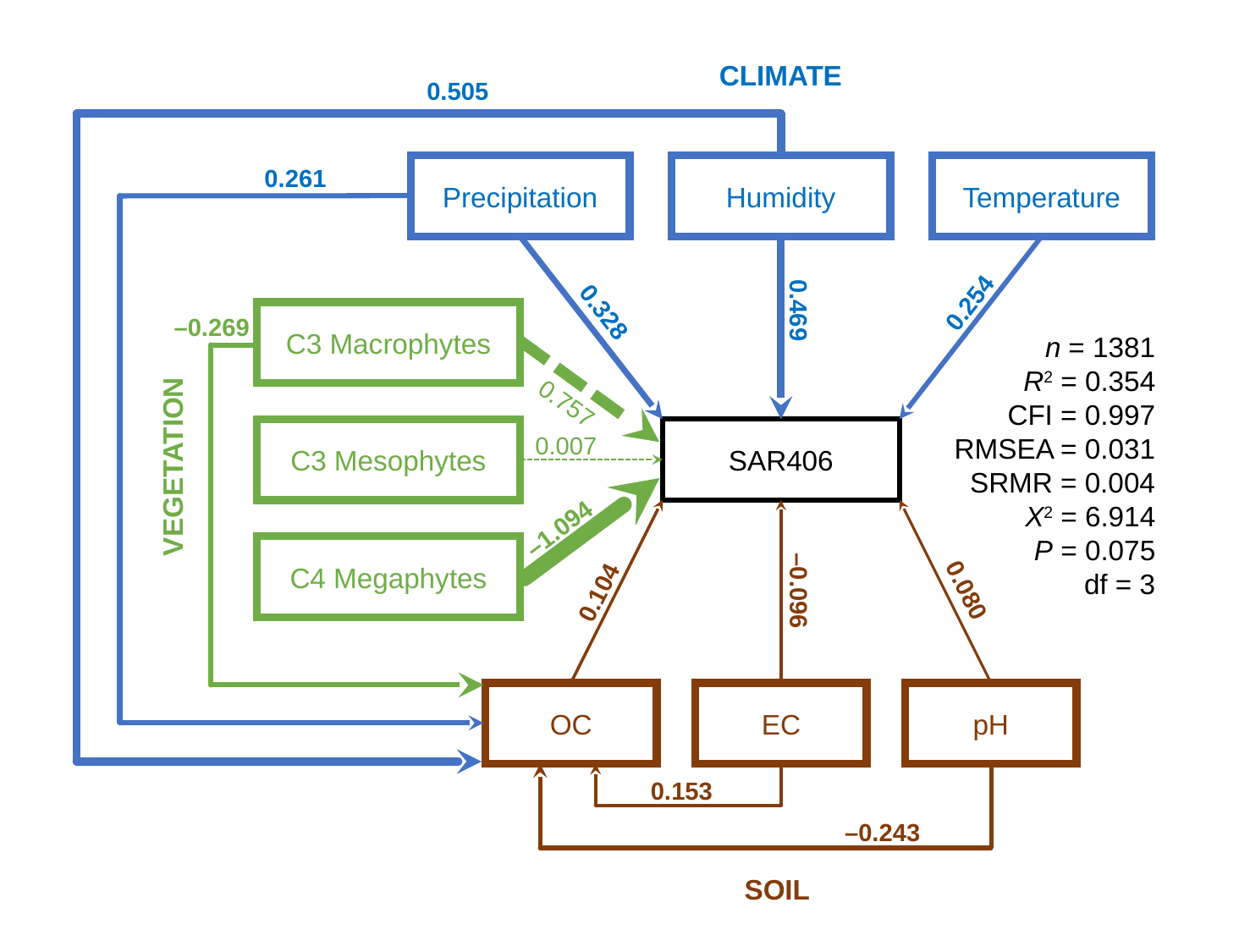

CLIMATE
0.505
Precipitation
Humidity
Temperature
0.261
0.254
0.469
0.328
C3 Macrophytes
–0.269
n = 1381
R2 = 0.354
CFI = 0.997
RMSEA = 0.031
SRMR = 0.004
Χ2 = 6.914
P = 0.075
df = 3
0.757
C3 Mesophytes
SAR406
0.007
VEGETATION
–1.094
C4 Megaphytes
0.080
–0.096
0.104
OC
EC
pH
0.153
–0.243
SOIL

## Slide 40
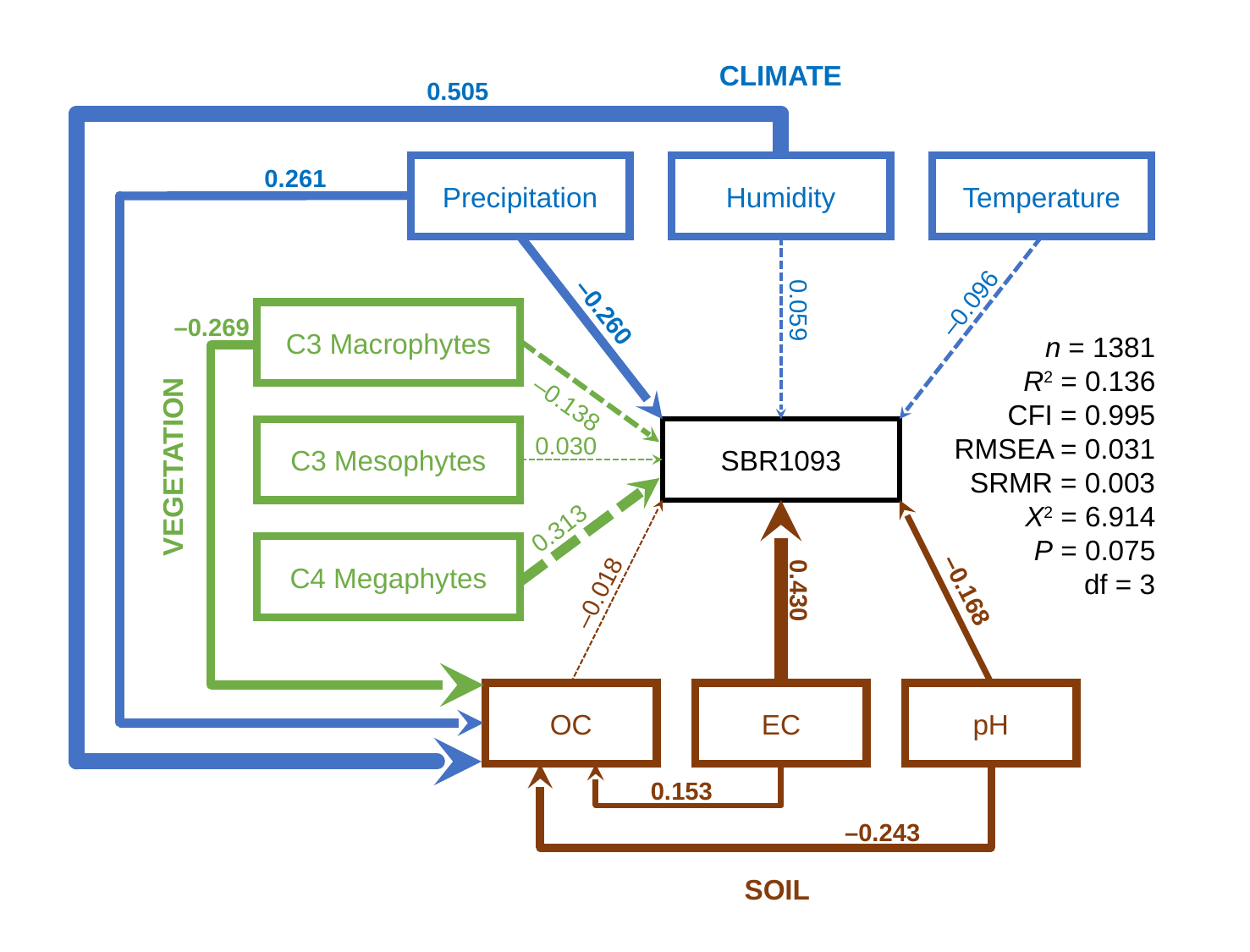

CLIMATE
0.505
Precipitation
Humidity
Temperature
0.261
–0.096
0.059
–0.260
C3 Macrophytes
–0.269
n = 1381
R2 = 0.136
CFI = 0.995
RMSEA = 0.031
SRMR = 0.003
Χ2 = 6.914
P = 0.075
df = 3
–0.138
C3 Mesophytes
SBR1093
0.030
VEGETATION
0.313
C4 Megaphytes
–0.168
0.430
–0.018
OC
EC
pH
0.153
–0.243
SOIL

## Slide 41
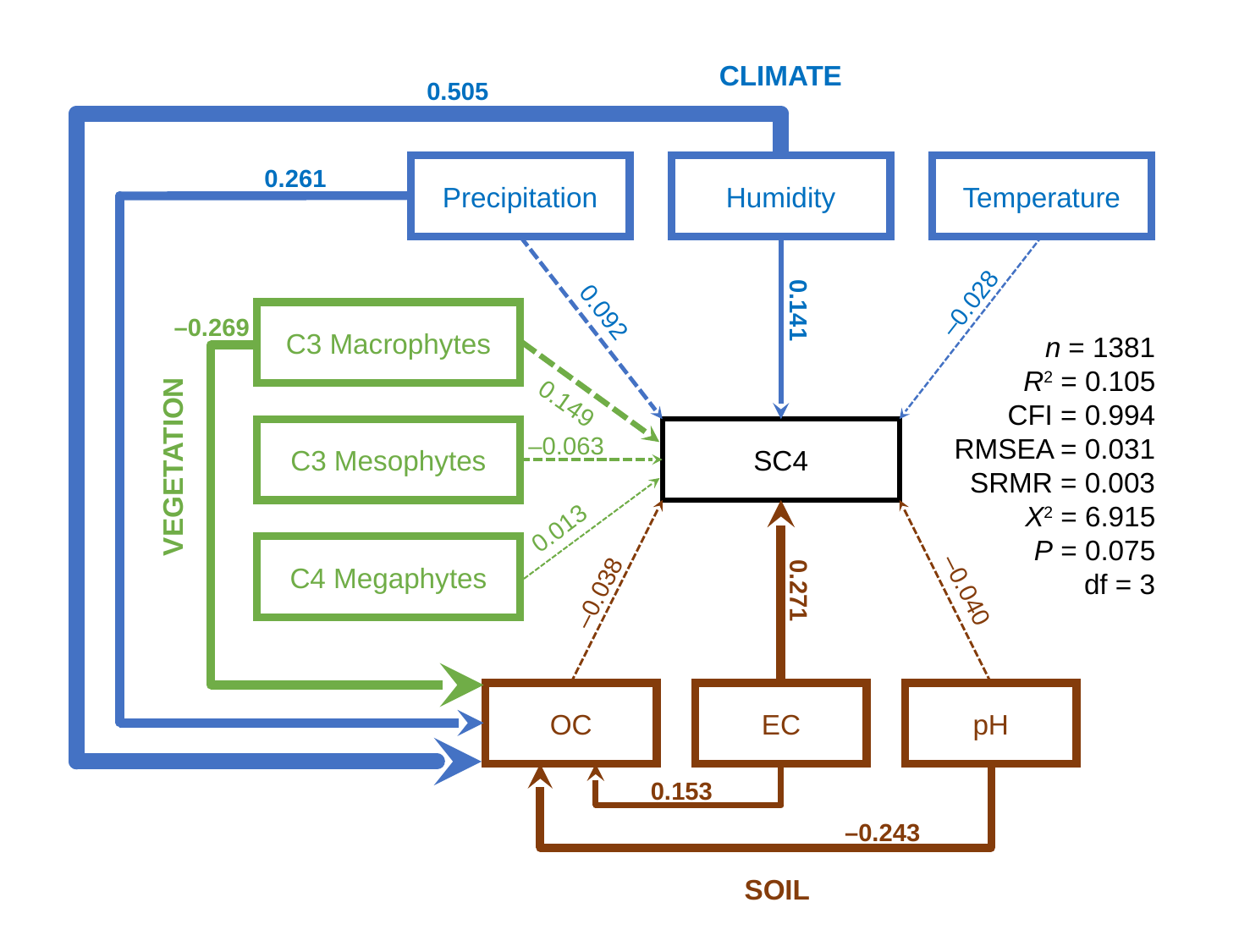

CLIMATE
0.505
Precipitation
Humidity
Temperature
0.261
–0.028
0.141
0.092
C3 Macrophytes
–0.269
n = 1381
R2 = 0.105
CFI = 0.994
RMSEA = 0.031
SRMR = 0.003
Χ2 = 6.915
P = 0.075
df = 3
0.149
C3 Mesophytes
SC4
–0.063
VEGETATION
0.013
C4 Megaphytes
–0.040
0.271
–0.038
OC
EC
pH
0.153
–0.243
SOIL

## Slide 42
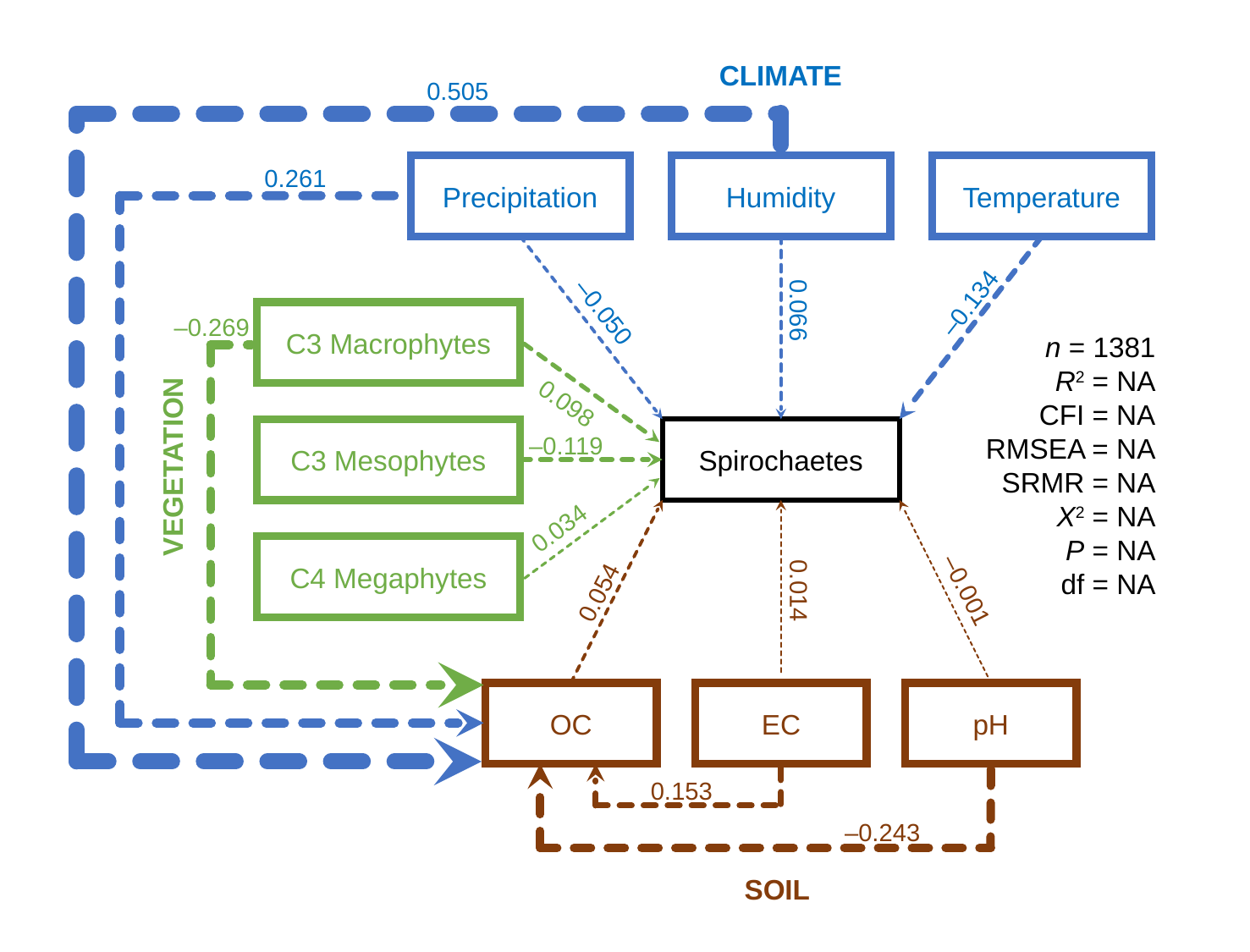

CLIMATE
0.505
Precipitation
Humidity
Temperature
0.261
–0.134
0.066
–0.050
C3 Macrophytes
–0.269
n = 1381
R2 = NA
CFI = NA
RMSEA = NA
SRMR = NA
Χ2 = NA
P = NA
df = NA
0.098
C3 Mesophytes
Spirochaetes
–0.119
VEGETATION
0.034
C4 Megaphytes
–0.001
0.014
0.054
OC
EC
pH
0.153
–0.243
SOIL

## Slide 43
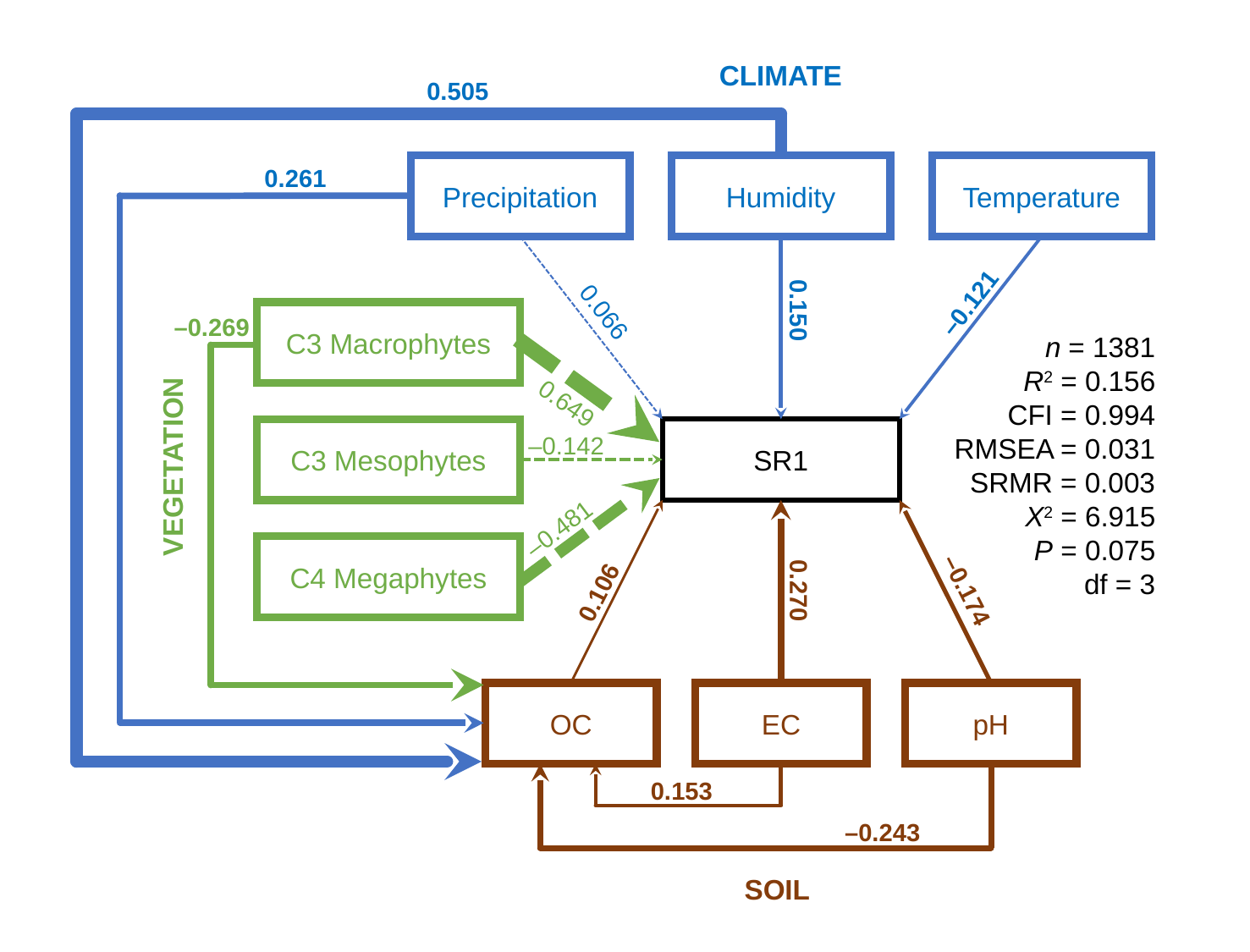

CLIMATE
0.505
Precipitation
Humidity
Temperature
0.261
–0.121
0.150
0.066
C3 Macrophytes
–0.269
n = 1381
R2 = 0.156
CFI = 0.994
RMSEA = 0.031
SRMR = 0.003
Χ2 = 6.915
P = 0.075
df = 3
0.649
C3 Mesophytes
SR1
–0.142
VEGETATION
–0.481
C4 Megaphytes
–0.174
0.270
0.106
OC
EC
pH
0.153
–0.243
SOIL

## Slide 44
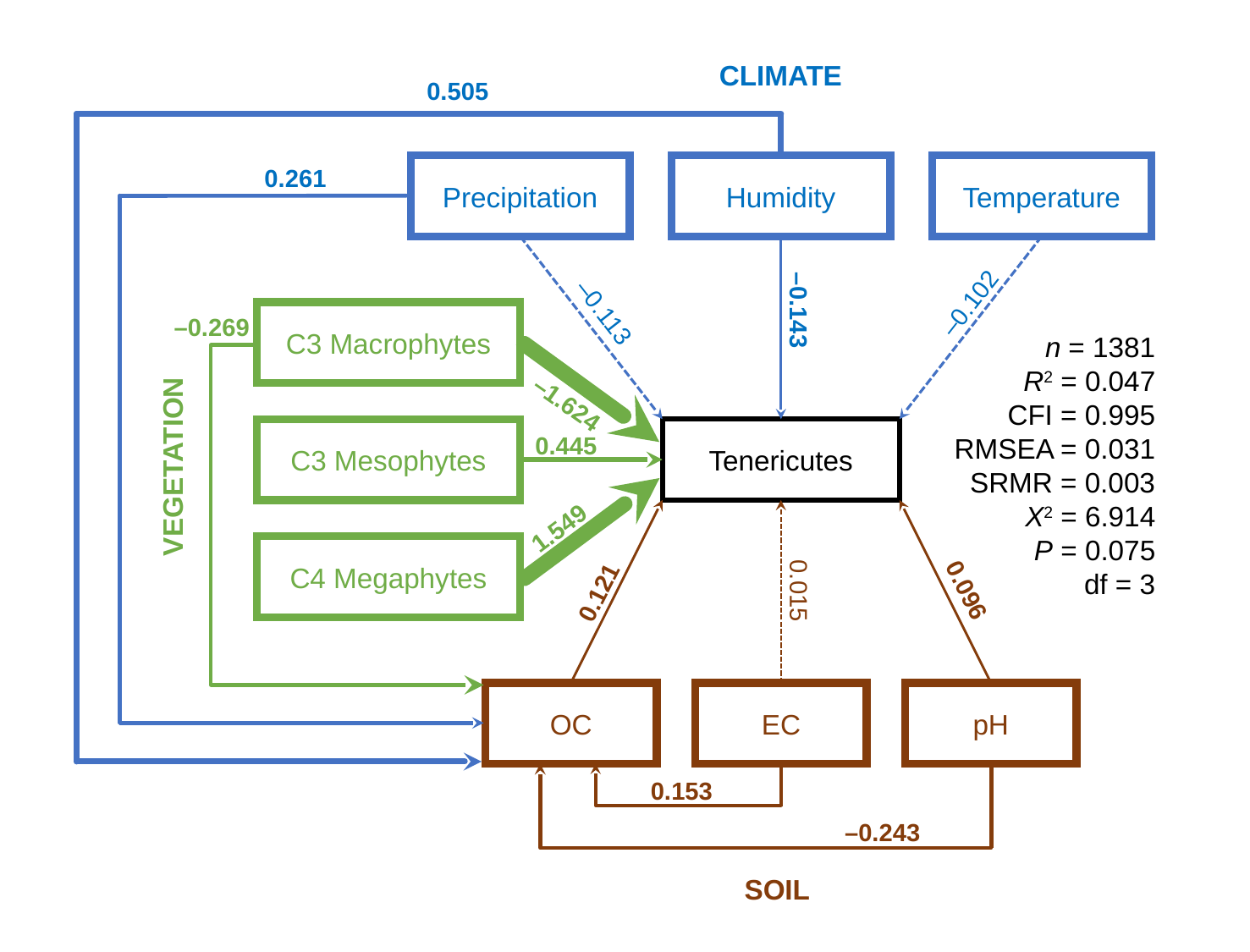

CLIMATE
0.505
Precipitation
Humidity
Temperature
0.261
–0.102
–0.143
–0.113
C3 Macrophytes
–0.269
n = 1381
R2 = 0.047
CFI = 0.995
RMSEA = 0.031
SRMR = 0.003
Χ2 = 6.914
P = 0.075
df = 3
–1.624
C3 Mesophytes
Tenericutes
0.445
VEGETATION
1.549
C4 Megaphytes
0.096
0.015
0.121
OC
EC
pH
0.153
–0.243
SOIL

## Slide 45
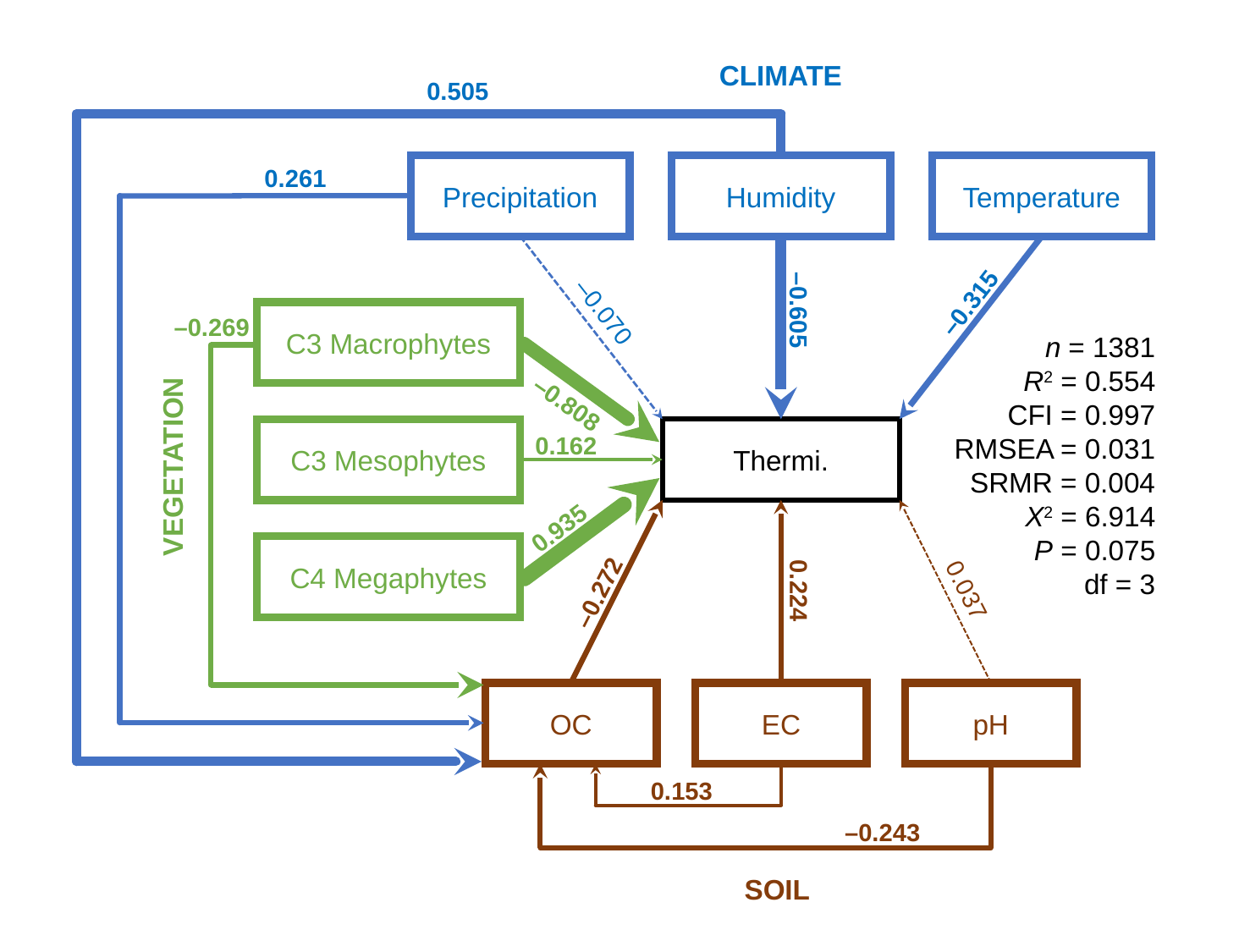

CLIMATE
0.505
Precipitation
Humidity
Temperature
0.261
–0.315
–0.605
–0.070
C3 Macrophytes
–0.269
n = 1381
R2 = 0.554
CFI = 0.997
RMSEA = 0.031
SRMR = 0.004
Χ2 = 6.914
P = 0.075
df = 3
–0.808
C3 Mesophytes
Thermi.
0.162
VEGETATION
0.935
C4 Megaphytes
0.037
0.224
–0.272
OC
EC
pH
0.153
–0.243
SOIL

## Slide 46
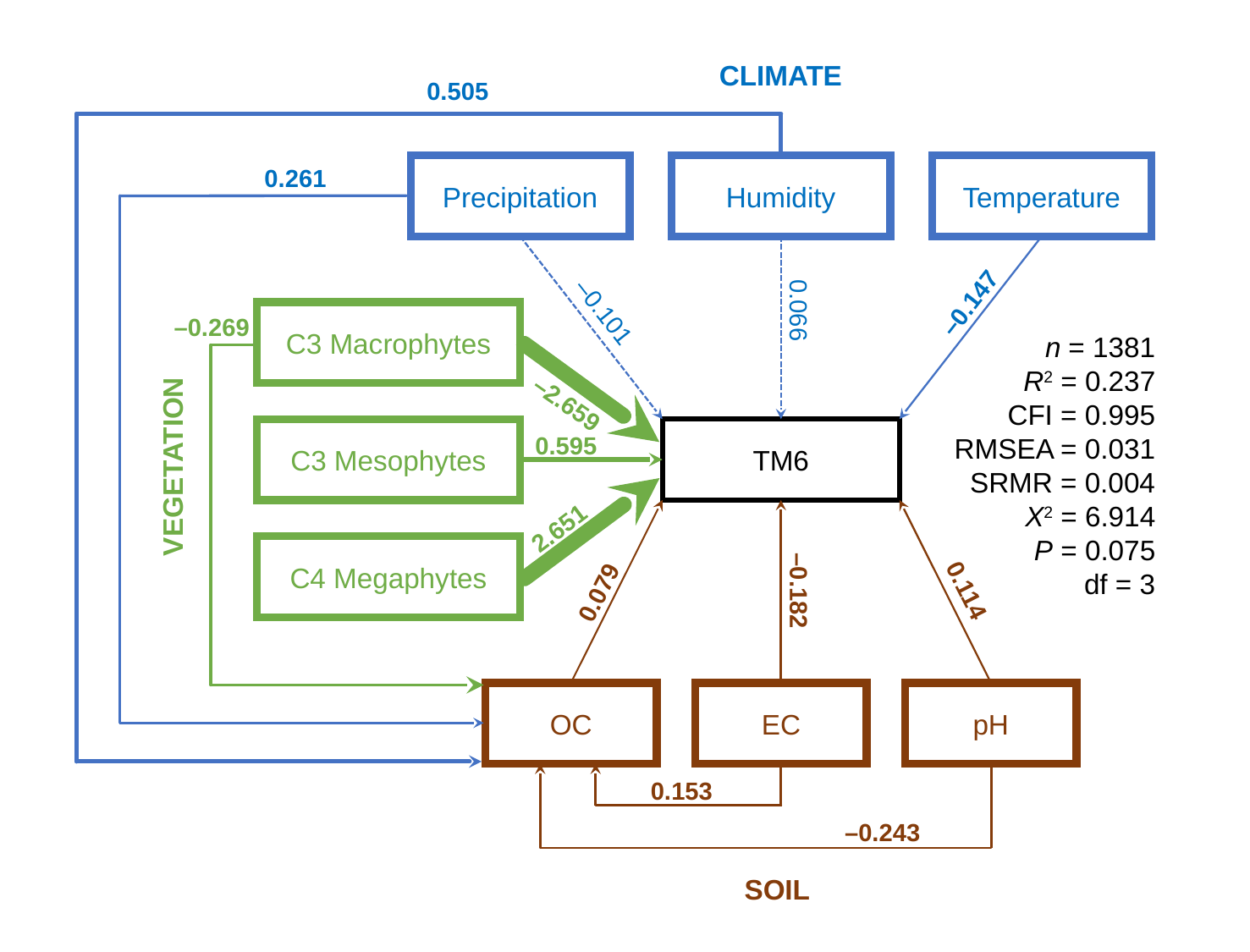

CLIMATE
0.505
Precipitation
Humidity
Temperature
0.261
–0.147
0.066
–0.101
C3 Macrophytes
–0.269
n = 1381
R2 = 0.237
CFI = 0.995
RMSEA = 0.031
SRMR = 0.004
Χ2 = 6.914
P = 0.075
df = 3
–2.659
C3 Mesophytes
TM6
0.595
VEGETATION
2.651
C4 Megaphytes
0.114
–0.182
0.079
OC
EC
pH
0.153
–0.243
SOIL

## Slide 47
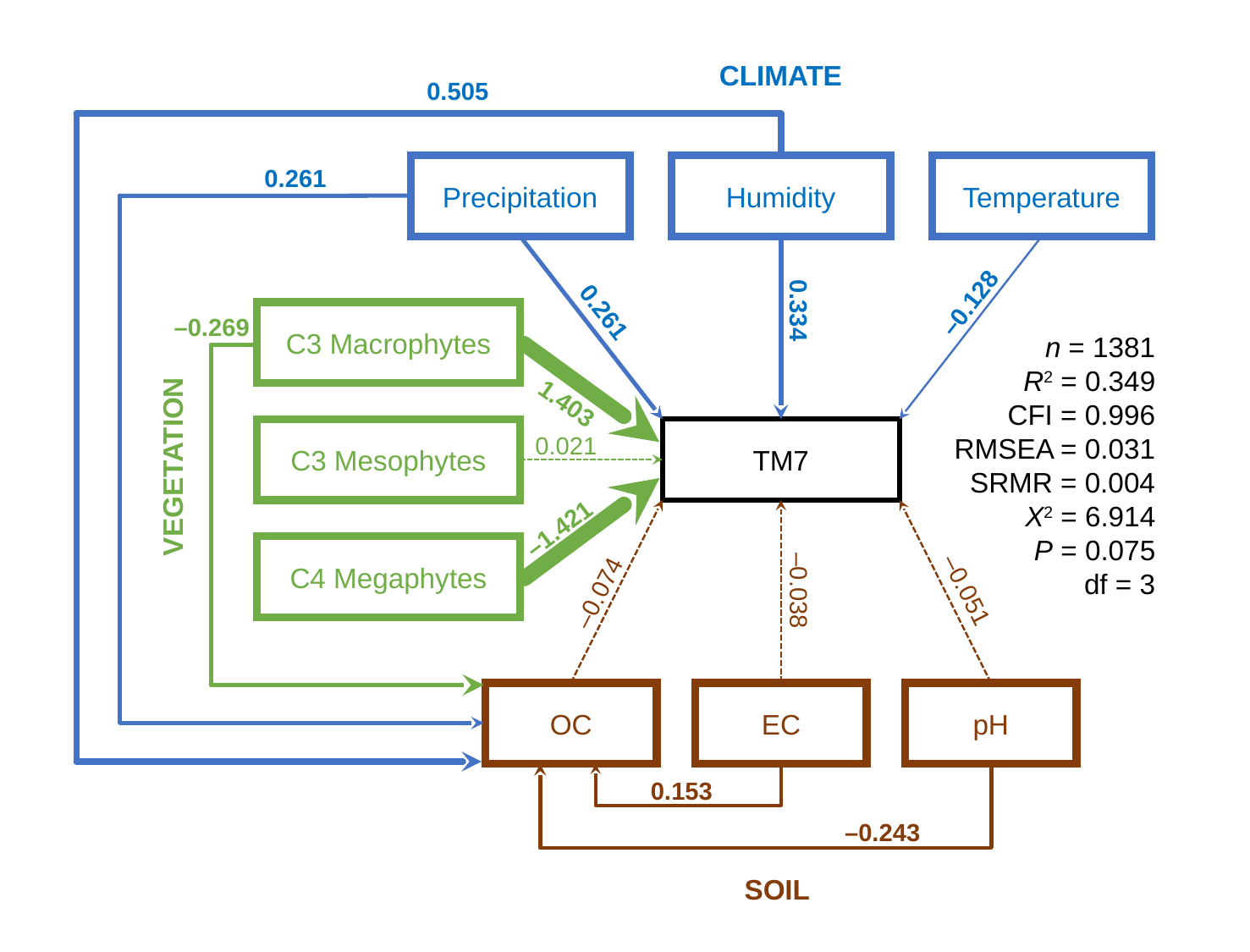

CLIMATE
0.505
Precipitation
Humidity
Temperature
0.261
–0.128
0.334
0.261
C3 Macrophytes
–0.269
n = 1381
R2 = 0.349
CFI = 0.996
RMSEA = 0.031
SRMR = 0.004
Χ2 = 6.914
P = 0.075
df = 3
1.403
C3 Mesophytes
TM7
0.021
VEGETATION
–1.421
C4 Megaphytes
–0.051
–0.038
–0.074
OC
EC
pH
0.153
–0.243
SOIL

## Slide 48
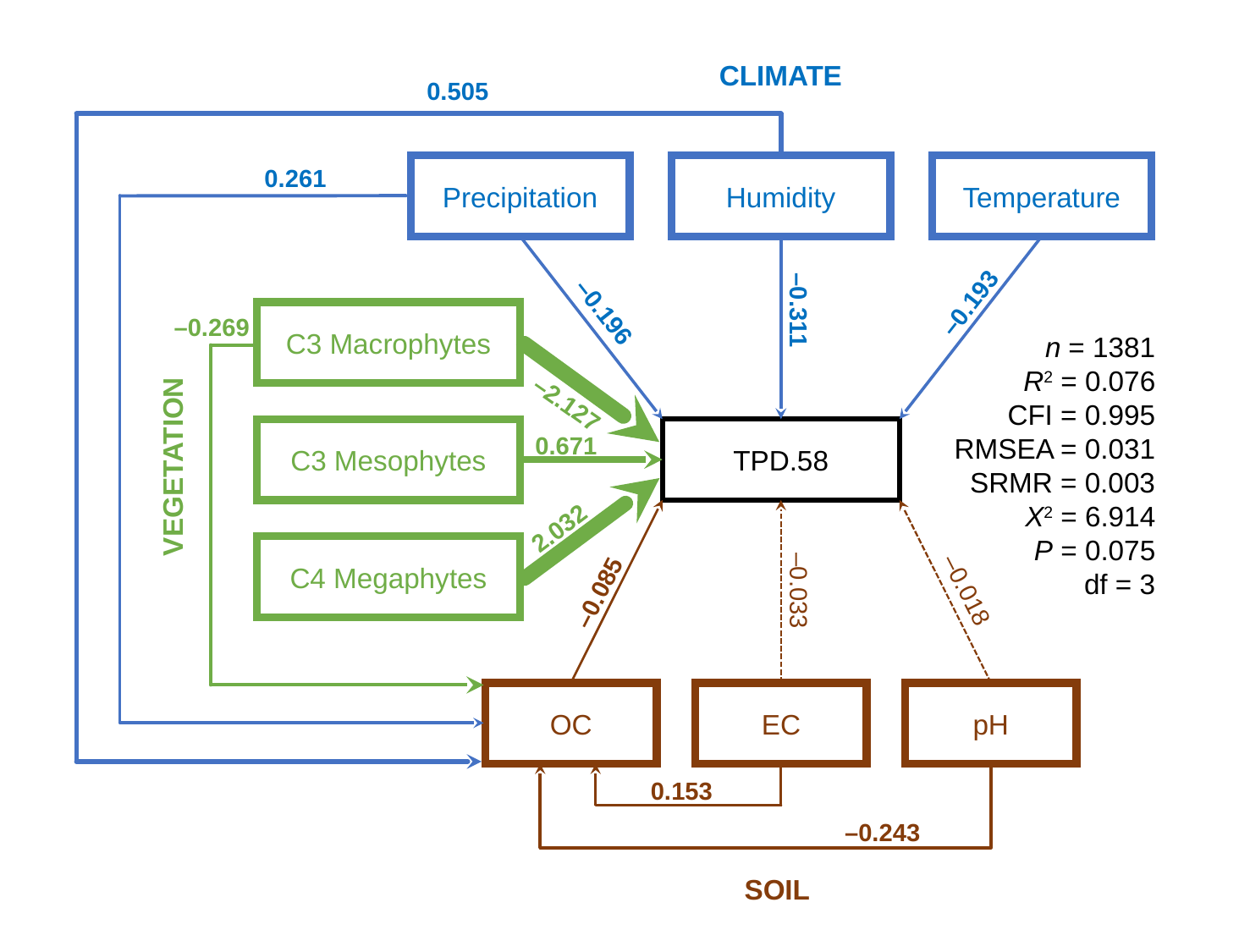

CLIMATE
0.505
Precipitation
Humidity
Temperature
0.261
–0.193
–0.311
–0.196
C3 Macrophytes
–0.269
n = 1381
R2 = 0.076
CFI = 0.995
RMSEA = 0.031
SRMR = 0.003
Χ2 = 6.914
P = 0.075
df = 3
–2.127
C3 Mesophytes
TPD.58
0.671
VEGETATION
2.032
C4 Megaphytes
–0.018
–0.033
–0.085
OC
EC
pH
0.153
–0.243
SOIL

## Slide 49
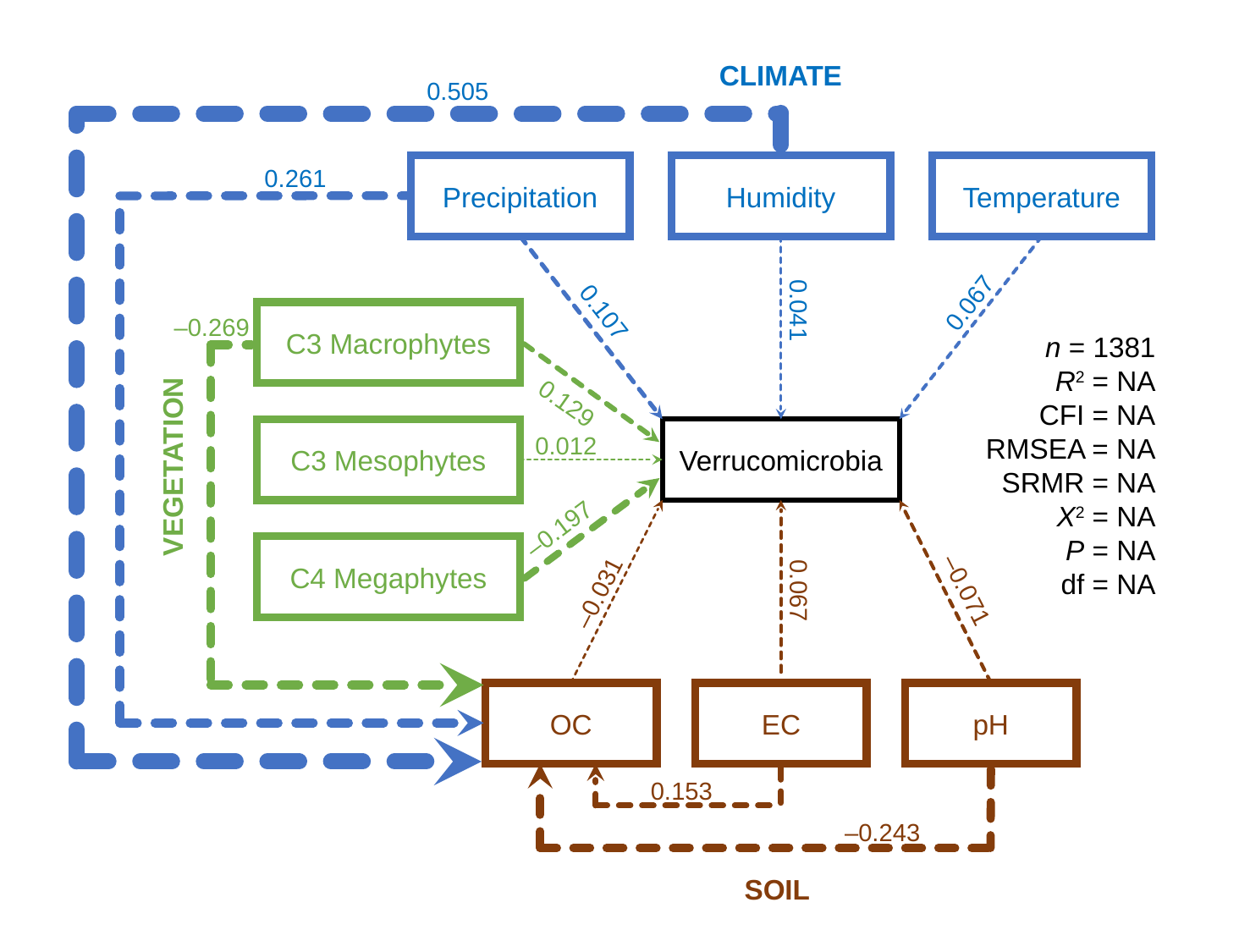

CLIMATE
0.505
Precipitation
Humidity
Temperature
0.261
0.067
0.041
0.107
C3 Macrophytes
–0.269
n = 1381
R2 = NA
CFI = NA
RMSEA = NA
SRMR = NA
Χ2 = NA
P = NA
df = NA
0.129
C3 Mesophytes
Verrucomicrobia
0.012
VEGETATION
–0.197
C4 Megaphytes
–0.071
0.067
–0.031
OC
EC
pH
0.153
–0.243
SOIL

## Slide 50
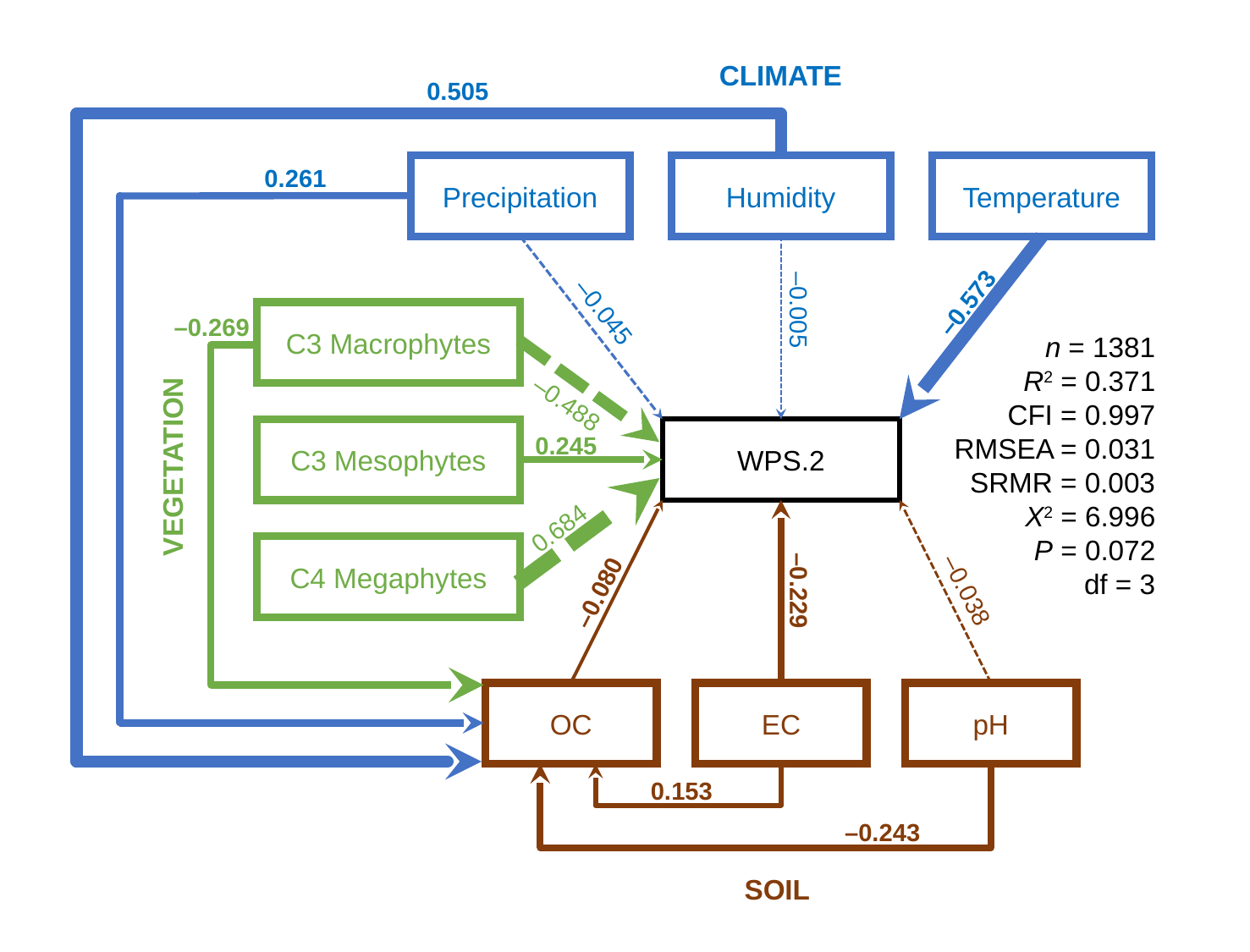

CLIMATE
0.505
Precipitation
Humidity
Temperature
0.261
–0.573
–0.005
–0.045
C3 Macrophytes
–0.269
n = 1381
R2 = 0.371
CFI = 0.997
RMSEA = 0.031
SRMR = 0.003
Χ2 = 6.996
P = 0.072
df = 3
–0.488
C3 Mesophytes
WPS.2
0.245
VEGETATION
0.684
C4 Megaphytes
–0.038
–0.229
–0.080
OC
EC
pH
0.153
–0.243
SOIL

## Slide 51
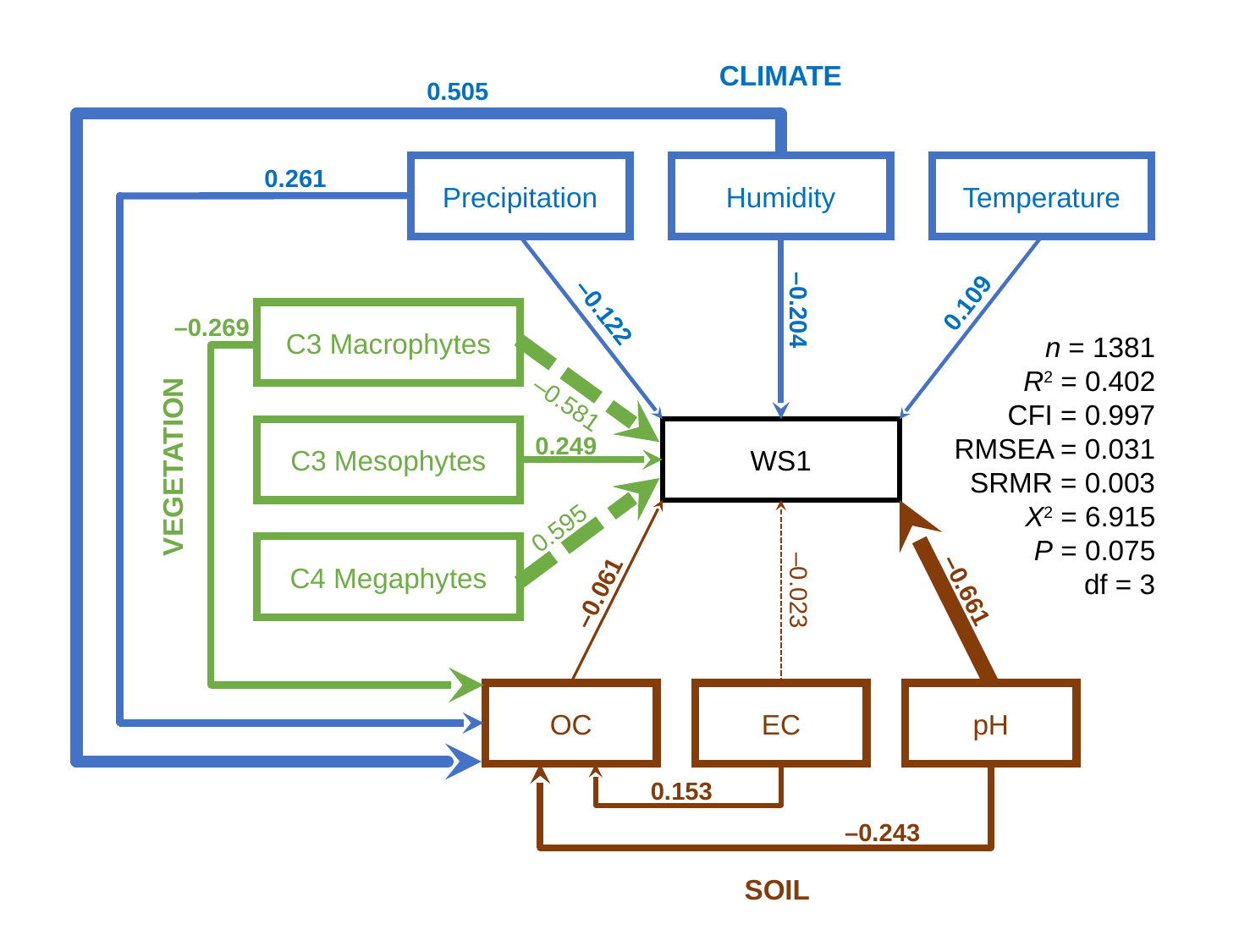

CLIMATE
0.505
Precipitation
Humidity
Temperature
0.261
0.109
–0.204
–0.122
C3 Macrophytes
–0.269
n = 1381
R2 = 0.402
CFI = 0.997
RMSEA = 0.031
SRMR = 0.003
Χ2 = 6.915
P = 0.075
df = 3
–0.581
C3 Mesophytes
WS1
0.249
VEGETATION
0.595
C4 Megaphytes
–0.661
–0.023
–0.061
OC
EC
pH
0.153
–0.243
SOIL

## Slide 52
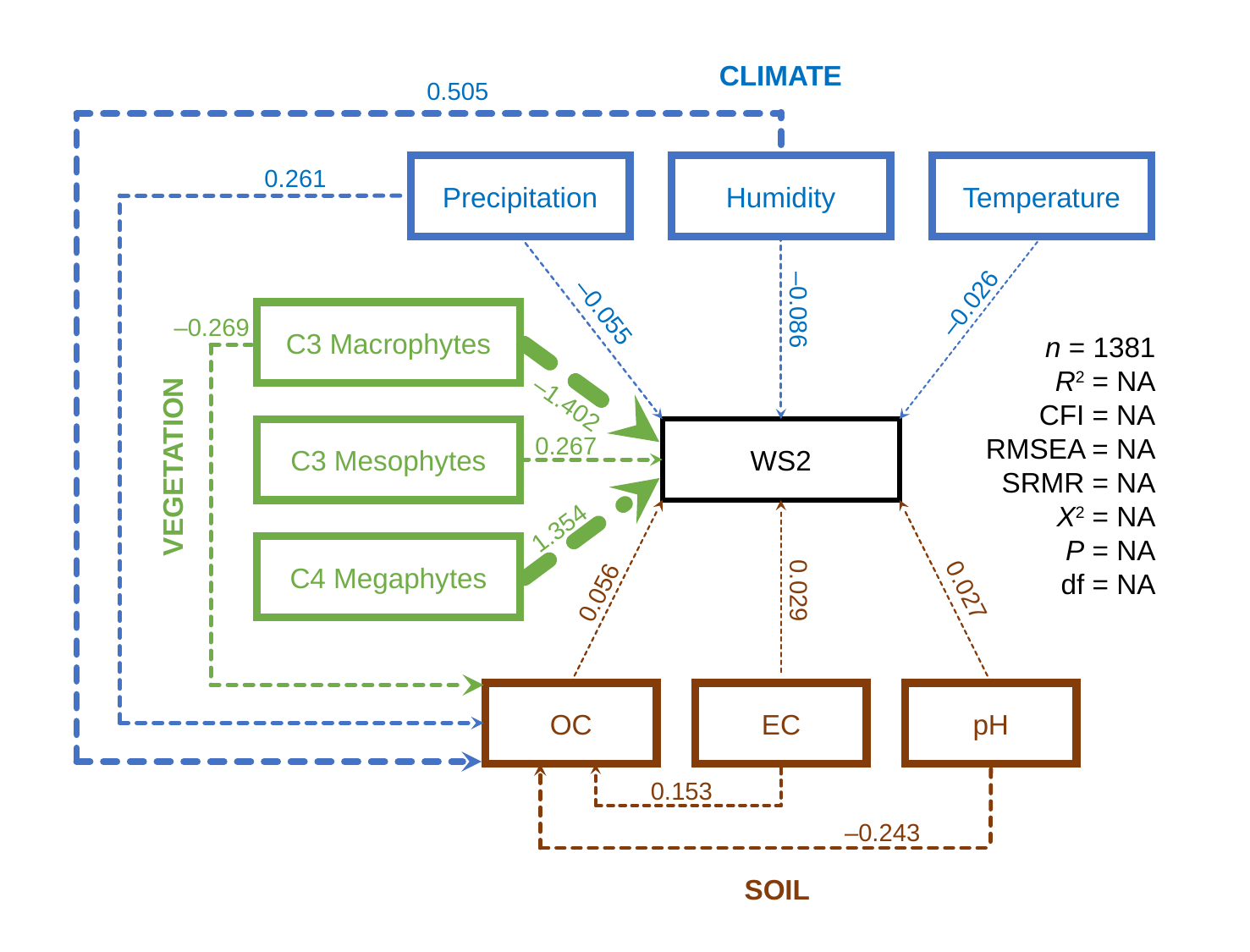

CLIMATE
0.505
Precipitation
Humidity
Temperature
0.261
–0.026
–0.086
–0.055
C3 Macrophytes
–0.269
n = 1381
R2 = NA
CFI = NA
RMSEA = NA
SRMR = NA
Χ2 = NA
P = NA
df = NA
–1.402
C3 Mesophytes
WS2
0.267
VEGETATION
1.354
C4 Megaphytes
0.027
0.029
0.056
OC
EC
pH
0.153
–0.243
SOIL

## Slide 53
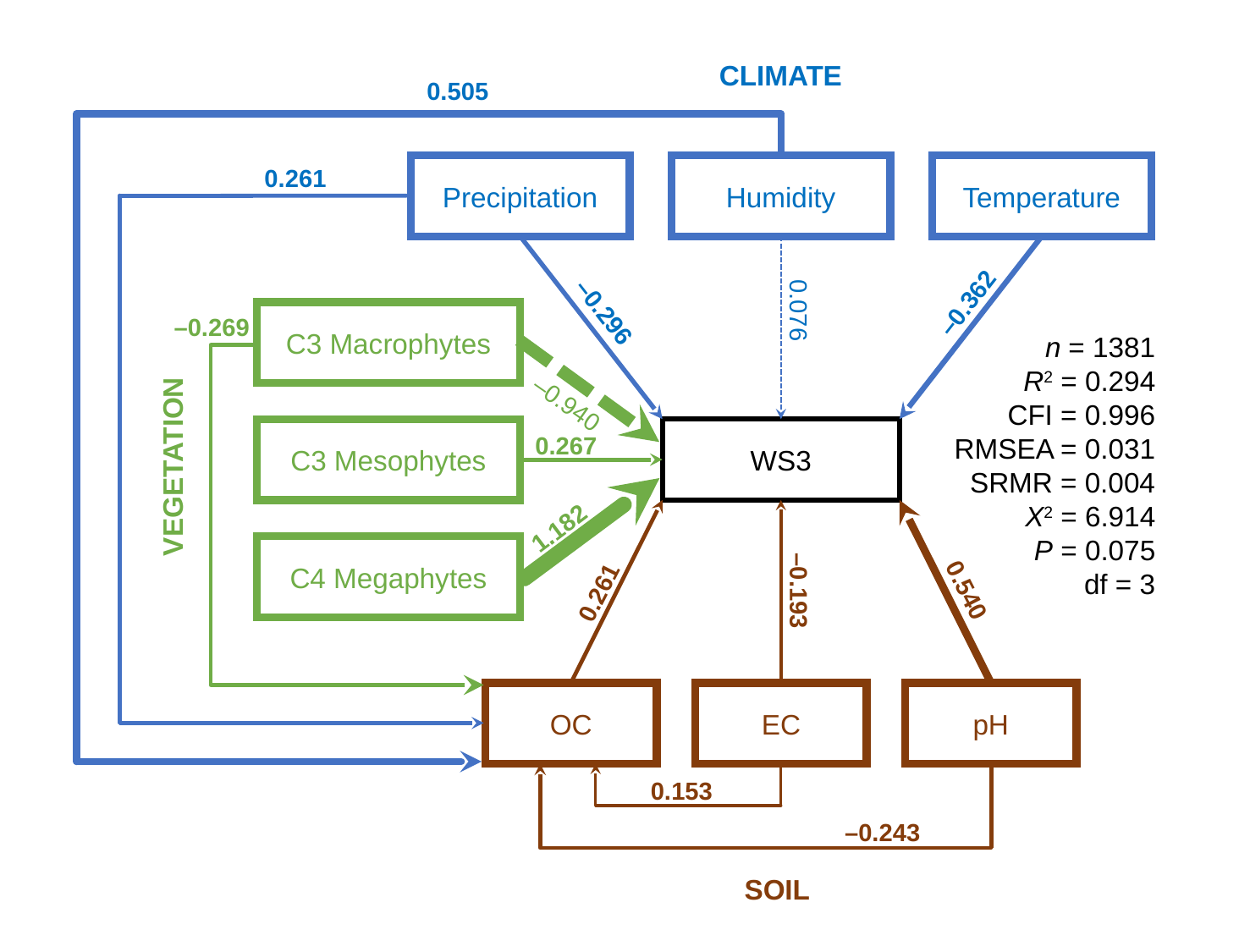

CLIMATE
0.505
Precipitation
Humidity
Temperature
0.261
–0.362
0.076
–0.296
C3 Macrophytes
–0.269
n = 1381
R2 = 0.294
CFI = 0.996
RMSEA = 0.031
SRMR = 0.004
Χ2 = 6.914
P = 0.075
df = 3
–0.940
C3 Mesophytes
WS3
0.267
VEGETATION
1.182
C4 Megaphytes
0.540
–0.193
0.261
OC
EC
pH
0.153
–0.243
SOIL

## Slide 54
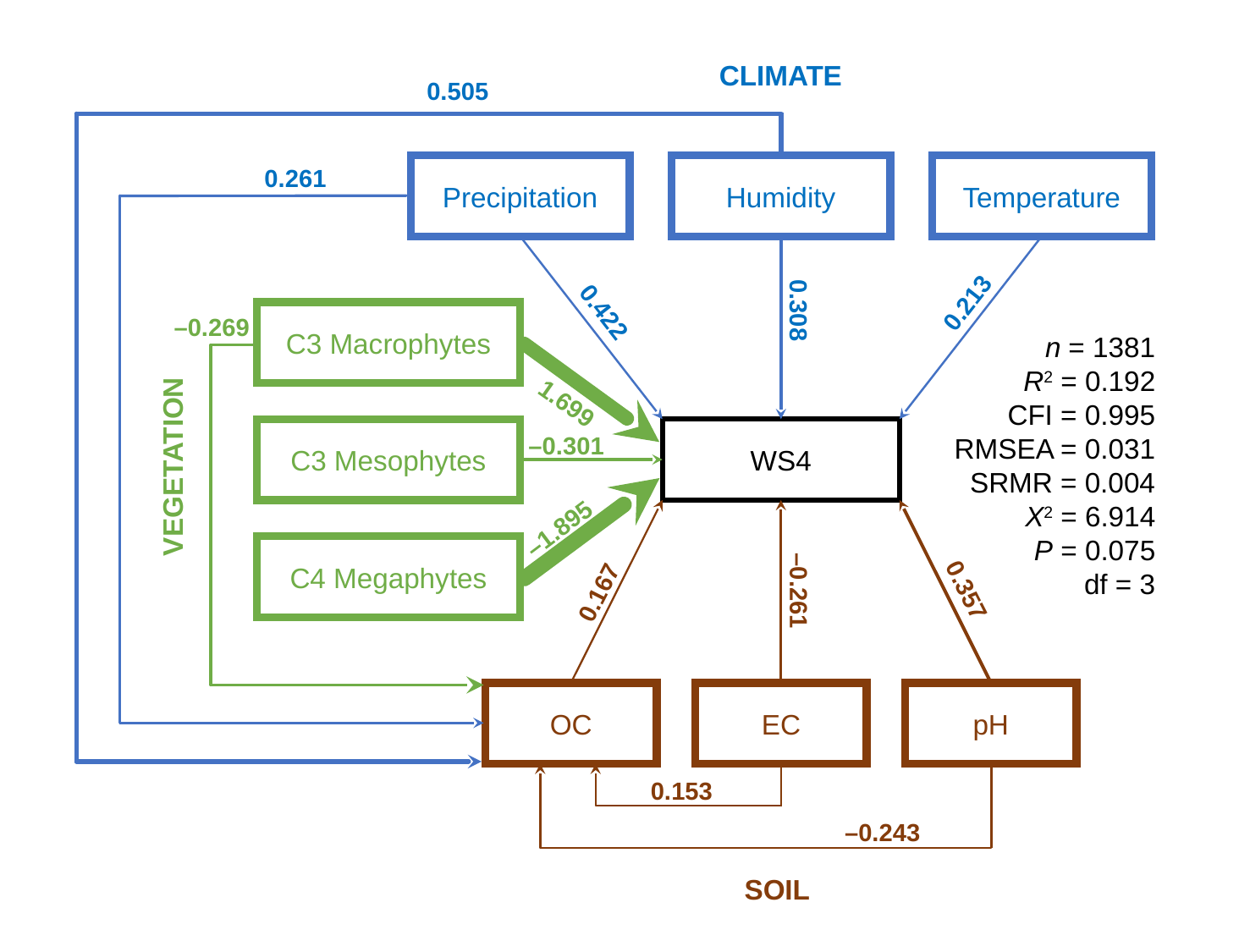

CLIMATE
0.505
Precipitation
Humidity
Temperature
0.261
0.213
0.308
0.422
C3 Macrophytes
–0.269
n = 1381
R2 = 0.192
CFI = 0.995
RMSEA = 0.031
SRMR = 0.004
Χ2 = 6.914
P = 0.075
df = 3
1.699
C3 Mesophytes
WS4
–0.301
VEGETATION
–1.895
C4 Megaphytes
0.357
–0.261
0.167
OC
EC
pH
0.153
–0.243
SOIL

## Slide 55
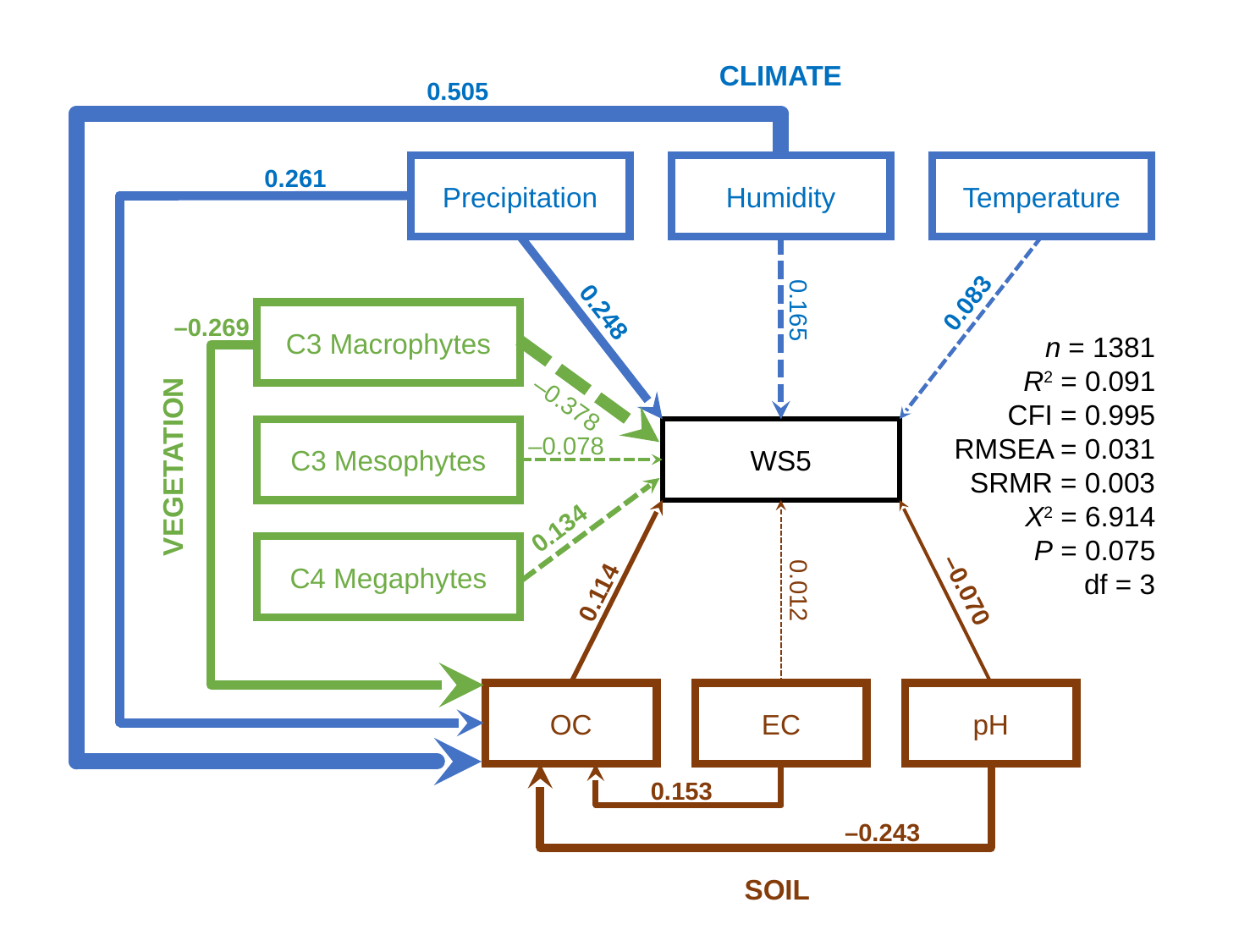

CLIMATE
0.505
Precipitation
Humidity
Temperature
0.261
0.083
0.165
0.248
C3 Macrophytes
–0.269
n = 1381
R2 = 0.091
CFI = 0.995
RMSEA = 0.031
SRMR = 0.003
Χ2 = 6.914
P = 0.075
df = 3
–0.378
C3 Mesophytes
WS5
–0.078
VEGETATION
0.134
C4 Megaphytes
–0.070
0.012
0.114
OC
EC
pH
0.153
–0.243
SOIL

## Slide 56
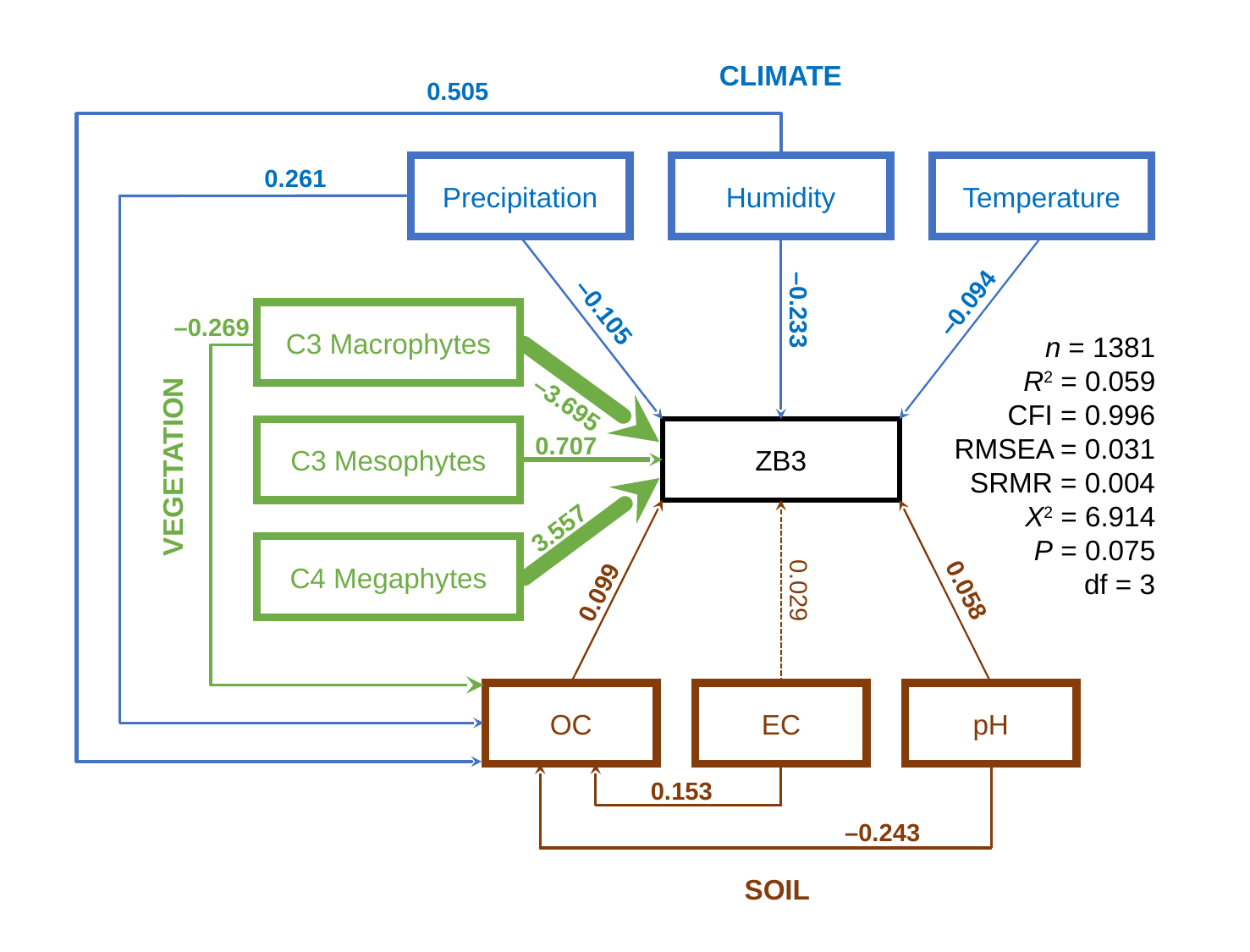

CLIMATE
0.505
Precipitation
Humidity
Temperature
0.261
–0.094
–0.233
–0.105
C3 Macrophytes
–0.269
n = 1381
R2 = 0.059
CFI = 0.996
RMSEA = 0.031
SRMR = 0.004
Χ2 = 6.914
P = 0.075
df = 3
–3.695
C3 Mesophytes
ZB3
0.707
VEGETATION
3.557
C4 Megaphytes
0.058
0.029
0.099
OC
EC
pH
0.153
–0.243
SOIL
