# Supplementary material for: Linking niche size and phylogenetic signals to predict future soil microbial relative abundances
Source: Front Microbiol. 2023 Aug 14;14:1097909. doi: 10.3389/fmicb.2023.1097909 (PMC10461061; doi:10.3389/fmicb.2023.1097909)
Supplement: Supplementary file 1 [file Data_Sheet_1.zip › Sup_figures_captions.R1.docx]

**Supplemental Information**

Table of Contents

**Figure S1:** Map of Sample Locations across Australia.

**Figure S2**: Example of relationships between the nine endogenous variables and the distribution of Acidobacteria.

**Figure S3**: Example of relationships between the nine endogenous variables and the distribution of Actinobacteria.

**Figure S4**: Link between SEM clusters and climate niches of select phyla.

**Figure S5**: Example eHOF model analysis of Verrucomicrobia and V. Opitutae.

**Figures S6-S61.** Structural Equation Models of Phylum Abundance across 1381 samples.

1. AC1
2. Acidobacteria
3. Actinobacteria
4. AD3
5. Aquificae
6. Armatimonadetes
7. Bacteroidetes
8. BRC1
9. Caldiserica
10. Caldithrix
11. Caldithrix.
12. Chlorobi
13. Chloroflexi
14. Cyanobacteria
15. Elusimicrobia
16. FBP
17. FCPU426
18. Fibrobacteres
19. Firmicutes
20. Fusobacteria
21. GAL15
22. Gemmatimonadetes
23. GN02
24. GN04
25. GOUTA4
26. Kazan.3B.28
27. KNB19
28. Lentisphaerae
29. MVP.21
30. NC10
31. Nitrospirae
32. OD1
33. OP1
34. OP11
35. OP3
36. OP8
37. PAUC34f
38. Planctomycetes
39. SAR406
40. SBR1093
41. SC4
42. Spirochaetes
43. SR1
44. Tenericutes
45. Thermi.
46. TM6
47. TM7
48. TPD.58
49. Verrucomicrobia
50. WPS.2
51. WS1
52. WS2
53. WS3
54. WS4
55. WS5
56. ZB3

**Figure S63**: Relationship between 16S rRNA amplicon PICRUST predicted and shotgun metagenomic derived methane metabolism genes in 370 of the 1281 samples used in this study (OLS regression R-squared = 0.9, P < 0.05). Metagenomic reads analysed using superfocus *(Silva, G. G. Z., Green K., B. E. Dutilh, and R. A. Edwards: SUPER-FOCUS: A tool for agile functional analysis of shotgun metagenomic data. Bioinformatics. 2015 Oct 9. pii: btv584. Website:* [*https://edwards.sdsu.edu/SUPERFOCUS*](https://edwards.sdsu.edu/SUPERFOCUS)*)*. R^2^ = 0.9.

**Figure S64: Gridded climate and plant related data used in models.**

**Table S1.** Sample numbers and geolocation of samples used in this study.

**Table S2:** Summary fit statistics for SEMs at the Phyla level.

**Table S3:** Phylogenetic signal of SEM traits with traits significantly different from zero, as determined by a permutation test (n=99).
