# Supplementary material for: Linking niche size and phylogenetic signals to predict future soil microbial relative abundances
Source: Front Microbiol. 2023 Aug 14;14:1097909. doi: 10.3389/fmicb.2023.1097909 (PMC10461061; doi:10.3389/fmicb.2023.1097909)
Supplement: Supplementary file 1 [file Data_Sheet_1.zip › Table S1.docx]

**Table S1.** Sample numbers and geolocation of samples used in this study. Data for all soil samples were obtained from the Biomes of Australian Soil Environments project (<https://data.bioplatforms.com/organization/about/bpa-base>).

| **Sample ID** | **latitude** | **longitude** |
| --- | --- | --- |
| 102.100.100/10603 | -10.4929 | 105.5699 |
| 102.100.100/10605 | -10.489 | 105.5708 |
| 102.100.100/10606 | -10.489 | 105.5708 |
| 102.100.100/10609 | -10.4835 | 105.6443 |
| 102.100.100/10610 | -10.4835 | 105.6443 |
| 102.100.100/10611 | -10.477 | 105.6455 |
| 102.100.100/10612 | -10.477 | 105.6455 |
| 102.100.100/10613 | -10.4163 | 105.6549 |
| 102.100.100/10617 | -10.4488 | 105.7024 |
| 102.100.100/10618 | -10.4488 | 105.7024 |
| 102.100.100/10619 | -10.4813 | 105.5739 |
| 102.100.100/10620 | -10.4813 | 105.5739 |
| 102.100.100/10621 | -10.4788 | 105.5572 |
| 102.100.100/10622 | -10.4788 | 105.5572 |
| 102.100.100/10623 | -10.4825 | 105.5559 |
| 102.100.100/10624 | -10.4825 | 105.5559 |
| 102.100.100/10714 | -34.0597 | 140.6958 |
| 102.100.100/10715 | -34.0597 | 140.6958 |
| 102.100.100/10716 | -34.0562 | 140.7354 |
| 102.100.100/10717 | -34.0562 | 140.7354 |
| 102.100.100/10718 | -34.0372 | 140.7064 |
| 102.100.100/10719 | -34.0372 | 140.7064 |
| 102.100.100/10720 | -33.9556 | 140.719 |
| 102.100.100/10721 | -33.9556 | 140.719 |
| 102.100.100/12424 | -42.1074 | 148.3395 |
| 102.100.100/12425 | -42.1074 | 148.3395 |
| 102.100.100/12426 | -42.1062 | 148.3408 |
| 102.100.100/12427 | -42.1062 | 148.3408 |
| 102.100.100/12428 | -42.1058 | 148.3367 |
| 102.100.100/12429 | -42.1058 | 148.3367 |
| 102.100.100/12430 | -42.1685 | 148.2641 |
| 102.100.100/12431 | -42.1685 | 148.2641 |
| 102.100.100/12432 | -42.1738 | 148.2791 |
| 102.100.100/12433 | -42.1738 | 148.2791 |
| 102.100.100/12434 | -42.173 | 148.2851 |
| 102.100.100/12435 | -42.173 | 148.2851 |
| 102.100.100/12436 | -42.0585 | 148.3002 |
| 102.100.100/12437 | -42.0585 | 148.3002 |
| 102.100.100/12438 | -42.0578 | 148.3021 |
| 102.100.100/12439 | -42.0578 | 148.3021 |
| 102.100.100/12440 | -42.0233 | 148.2828 |
| 102.100.100/12441 | -42.0233 | 148.2828 |
| 102.100.100/12442 | -42.0228 | 148.2833 |
| 102.100.100/12443 | -42.0228 | 148.2833 |
| 102.100.100/12444 | -42.6831 | 146.6099 |
| 102.100.100/12445 | -42.6831 | 146.6099 |
| 102.100.100/12446 | -42.6735 | 146.625 |
| 102.100.100/12447 | -42.6793 | 146.6711 |
| 102.100.100/12448 | -42.6793 | 146.6711 |
| 102.100.100/12449 | -30.201 | 149.5974 |
| 102.100.100/12450 | -30.2012 | 149.5974 |
| 102.100.100/12451 | -30.2012 | 149.5974 |
| 102.100.100/12459 | -35.4236 | 148.7989 |
| 102.100.100/12460 | -35.4236 | 148.7989 |
| 102.100.100/12461 | -35.4237 | 148.7976 |
| 102.100.100/12462 | -35.4237 | 148.7976 |
| 102.100.100/12463 | -35.4239 | 148.7979 |
| 102.100.100/12464 | -35.4239 | 148.7979 |
| 102.100.100/12465 | -35.4214 | 148.7986 |
| 102.100.100/12466 | -35.4214 | 148.7986 |
| 102.100.100/12467 | -35.4238 | 148.796 |
| 102.100.100/12468 | -35.4238 | 148.796 |
| 102.100.100/12469 | -35.4235 | 148.7968 |
| 102.100.100/12470 | -35.4235 | 148.7968 |
| 102.100.100/12471 | -35.4239 | 148.7972 |
| 102.100.100/12472 | -35.4239 | 148.7972 |
| 102.100.100/12473 | -35.4233 | 148.7968 |
| 102.100.100/12474 | -35.4233 | 148.7968 |
| 102.100.100/12475 | -35.4239 | 148.7966 |
| 102.100.100/12476 | -35.4239 | 148.7966 |
| 102.100.100/12477 | -35.4226 | 148.7961 |
| 102.100.100/12478 | -35.4226 | 148.7961 |
| 102.100.100/12479 | -35.4232 | 148.7964 |
| 102.100.100/12480 | -35.4232 | 148.7964 |
| 102.100.100/12481 | -32.526 | 142.2193 |
| 102.100.100/12482 | -32.526 | 142.2193 |
| 102.100.100/12483 | -29.0557 | 141.8981 |
| 102.100.100/12484 | -29.0557 | 141.8981 |
| 102.100.100/12485 | -29.0359 | 141.3528 |
| 102.100.100/12486 | -29.0359 | 141.3528 |
| 102.100.100/12487 | -29.9456 | 144.1117 |
| 102.100.100/12488 | -29.9456 | 144.1117 |
| 102.100.100/12489 | -30.395 | 142.7178 |
| 102.100.100/12490 | -30.395 | 142.7178 |
| 102.100.100/12491 | -35.881 | 149.0076 |
| 102.100.100/12492 | -35.881 | 149.0076 |
| 102.100.100/12493 | -35.8811 | 149.0083 |
| 102.100.100/12494 | -35.8811 | 149.0083 |
| 102.100.100/12495 | -35.8767 | 149.0097 |
| 102.100.100/12496 | -35.8767 | 149.0097 |
| 102.100.100/12497 | -35.8769 | 149.0103 |
| 102.100.100/12498 | -35.8769 | 149.0103 |
| 102.100.100/12499 | -35.8749 | 149.0109 |
| 102.100.100/12500 | -35.8749 | 149.0109 |
| 102.100.100/12501 | -30.2005 | 149.5969 |
| 102.100.100/12502 | -30.2005 | 149.5969 |
| 102.100.100/12503 | -30.2009 | 149.5969 |
| 102.100.100/12504 | -30.2009 | 149.5969 |
| 102.100.100/12505 | -30.2018 | 149.5968 |
| 102.100.100/12506 | -30.2018 | 149.5968 |
| 102.100.100/12507 | -30.2013 | 149.5969 |
| 102.100.100/12508 | -30.2013 | 149.5969 |
| 102.100.100/12509 | -30.2012 | 149.5969 |
| 102.100.100/12510 | -30.2012 | 149.5969 |
| 102.100.100/12511 | -30.2009 | 149.597 |
| 102.100.100/12512 | -30.2009 | 149.597 |
| 102.100.100/12513 | -30.2018 | 149.597 |
| 102.100.100/12514 | -30.2018 | 149.597 |
| 102.100.100/12515 | -30.2015 | 149.597 |
| 102.100.100/12516 | -30.2015 | 149.597 |
| 102.100.100/12517 | -30.2017 | 149.597 |
| 102.100.100/12518 | -30.2017 | 149.597 |
| 102.100.100/12519 | -30.2005 | 149.5973 |
| 102.100.100/12520 | -30.2005 | 149.5973 |
| 102.100.100/12521 | -30.2007 | 149.5972 |
| 102.100.100/12522 | -30.2007 | 149.5972 |
| 102.100.100/12523 | -30.2009 | 149.5972 |
| 102.100.100/12524 | -30.2009 | 149.5972 |
| 102.100.100/12525 | -30.2017 | 149.5972 |
| 102.100.100/12526 | -30.2017 | 149.5972 |
| 102.100.100/12527 | -30.2014 | 149.5973 |
| 102.100.100/12528 | -30.2014 | 149.5973 |
| 102.100.100/12529 | -30.201 | 149.5974 |
| 102.100.100/12560 | -35.881 | 149.0076 |
| 102.100.100/12561 | -35.881 | 149.0076 |
| 102.100.100/12562 | -35.8811 | 149.0083 |
| 102.100.100/12563 | -35.8811 | 149.0083 |
| 102.100.100/12564 | -35.8767 | 149.0097 |
| 102.100.100/12565 | -35.8767 | 149.0097 |
| 102.100.100/12566 | -35.8769 | 149.0103 |
| 102.100.100/12567 | -35.8769 | 149.0103 |
| 102.100.100/12568 | -35.8749 | 149.0109 |
| 102.100.100/12569 | -35.8749 | 149.0109 |
| 102.100.100/12570 | -35.8748 | 149.0117 |
| 102.100.100/12571 | -35.8748 | 149.0117 |
| 102.100.100/12572 | -35.881 | 149.0076 |
| 102.100.100/12573 | -35.881 | 149.0076 |
| 102.100.100/12574 | -35.8811 | 149.0083 |
| 102.100.100/12575 | -35.8811 | 149.0083 |
| 102.100.100/12576 | -35.8767 | 149.0097 |
| 102.100.100/12577 | -35.8767 | 149.0097 |
| 102.100.100/12578 | -35.8769 | 149.0103 |
| 102.100.100/12579 | -35.8769 | 149.0103 |
| 102.100.100/12580 | -35.8749 | 149.0109 |
| 102.100.100/12581 | -35.8749 | 149.0109 |
| 102.100.100/12582 | -35.8748 | 149.0117 |
| 102.100.100/12583 | -35.8748 | 149.0117 |
| 102.100.100/12584 | -34.428 | 142.2752 |
| 102.100.100/12585 | -34.428 | 142.2752 |
| 102.100.100/12586 | -34.428 | 142.2752 |
| 102.100.100/12587 | -34.428 | 142.2752 |
| 102.100.100/12588 | -35.2892 | 139.0228 |
| 102.100.100/12589 | -35.2892 | 139.0228 |
| 102.100.100/12590 | -35.2892 | 139.0228 |
| 102.100.100/12591 | -35.2892 | 139.0228 |
| 102.100.100/12592 | -34.0993 | 139.8489 |
| 102.100.100/12593 | -34.0993 | 139.8489 |
| 102.100.100/12594 | -34.0993 | 139.8489 |
| 102.100.100/12595 | -34.0993 | 139.8489 |
| 102.100.100/12614 | -33.6598 | 150.2654 |
| 102.100.100/12615 | -33.6598 | 150.2654 |
| 102.100.100/12616 | -33.6598 | 150.2654 |
| 102.100.100/12617 | -33.6598 | 150.2654 |
| 102.100.100/12618 | -33.6528 | 150.2682 |
| 102.100.100/12619 | -33.6528 | 150.2682 |
| 102.100.100/12620 | -33.6528 | 150.2682 |
| 102.100.100/12621 | -33.6528 | 150.2682 |
| 102.100.100/12622 | -33.653 | 150.2678 |
| 102.100.100/12623 | -33.653 | 150.2678 |
| 102.100.100/12624 | -33.6526 | 150.2678 |
| 102.100.100/12625 | -33.6526 | 150.2678 |
| 102.100.100/12816 | -16.1034 | 145.4479 |
| 102.100.100/12818 | -16.103 | 145.4472 |
| 102.100.100/12819 | -16.1034 | 145.4479 |
| 102.100.100/12824 | -17.1211 | 145.6309 |
| 102.100.100/12825 | -17.1211 | 145.6309 |
| 102.100.100/12828 | -17.1036 | 145.612 |
| 102.100.100/12829 | -17.1036 | 145.612 |
| 102.100.100/12830 | -17.0921 | 145.6202 |
| 102.100.100/12831 | -17.0921 | 145.6202 |
| 102.100.100/12832 | -27.3876 | 152.8783 |
| 102.100.100/12833 | -27.3876 | 152.8783 |
| 102.100.100/12834 | -27.3871 | 152.8791 |
| 102.100.100/12835 | -27.3871 | 152.8791 |
| 102.100.100/12836 | -27.3861 | 152.8789 |
| 102.100.100/12837 | -27.3861 | 152.8789 |
| 102.100.100/12838 | -27.6327 | 153.083 |
| 102.100.100/12839 | -27.6327 | 153.083 |
| 102.100.100/12858 | -27.3861 | 152.8789 |
| 102.100.100/12859 | -27.3861 | 152.8789 |
| 102.100.100/12860 | -22.2828 | 133.2493 |
| 102.100.100/12861 | -22.2828 | 133.2493 |
| 102.100.100/12862 | -22.2888 | 133.6136 |
| 102.100.100/12863 | -22.2888 | 133.6136 |
| 102.100.100/12876 | -14.1952 | 135.7192 |
| 102.100.100/12877 | -14.1952 | 135.7192 |
| 102.100.100/12878 | -12.9326 | 135.2019 |
| 102.100.100/12879 | -12.9326 | 135.2019 |
| 102.100.100/12880 | -12.5092 | 135.8103 |
| 102.100.100/12881 | -12.5092 | 135.8103 |
| 102.100.100/12882 | -13.5212 | 134.5062 |
| 102.100.100/12883 | -13.5212 | 134.5062 |
| 102.100.100/12884 | -36.6732 | 145.0292 |
| 102.100.100/12885 | -36.6732 | 145.0292 |
| 102.100.100/12886 | -36.6504 | 145.5755 |
| 102.100.100/12887 | -36.6504 | 145.5755 |
| 102.100.100/12891 | -37.2521 | 144.0539 |
| 102.100.100/12892 | -37.2521 | 144.0539 |
| 102.100.100/12897 | -34.0036 | 140.5876 |
| 102.100.100/12898 | -34.0036 | 140.5876 |
| 102.100.100/12899 | -34.098 | 140.3515 |
| 102.100.100/12900 | -34.098 | 140.3515 |
| 102.100.100/12901 | -33.9716 | 140.7281 |
| 102.100.100/12902 | -33.9716 | 140.7281 |
| 102.100.100/12903 | -43.0951 | 146.6543 |
| 102.100.100/12904 | -43.0951 | 146.6543 |
| 102.100.100/12939 | -13.4082 | 135.7763 |
| 102.100.100/12940 | -13.4082 | 135.7763 |
| 102.100.100/13260 | -39.9214 | 143.8515 |
| 102.100.100/13261 | -39.9214 | 143.8515 |
| 102.100.100/13262 | -40.1087 | 143.9054 |
| 102.100.100/13263 | -40.1087 | 143.9054 |
| 102.100.100/13264 | -40.1073 | 143.7058 |
| 102.100.100/13265 | -40.1073 | 143.7058 |
| 102.100.100/13266 | -39.9358 | 144.1195 |
| 102.100.100/13267 | -39.9358 | 144.1195 |
| 102.100.100/13268 | -39.935 | 144.1197 |
| 102.100.100/13269 | -39.935 | 144.1197 |
| 102.100.100/13270 | -39.8927 | 144.0821 |
| 102.100.100/13271 | -39.8927 | 144.0821 |
| 102.100.100/13272 | -39.8913 | 144.0732 |
| 102.100.100/13273 | -39.8913 | 144.0732 |
| 102.100.100/13274 | -39.7293 | 143.929 |
| 102.100.100/13275 | -39.7293 | 143.929 |
| 102.100.100/13276 | -39.6652 | 143.9776 |
| 102.100.100/13277 | -39.6652 | 143.9776 |
| 102.100.100/13278 | -39.6509 | 143.9728 |
| 102.100.100/13279 | -39.6509 | 143.9728 |
| 102.100.100/13280 | -39.6266 | 143.9715 |
| 102.100.100/13281 | -39.6266 | 143.9715 |
| 102.100.100/13282 | -39.8567 | 143.8935 |
| 102.100.100/13283 | -39.8567 | 143.8935 |
| 102.100.100/13284 | -39.8571 | 143.8985 |
| 102.100.100/13286 | -39.8644 | 143.8913 |
| 102.100.100/13287 | -39.8644 | 143.8913 |
| 102.100.100/13363 | -66.3677 | 110.5853 |
| 102.100.100/13364 | -66.3677 | 110.5853 |
| 102.100.100/13365 | -66.3677 | 110.5853 |
| 102.100.100/13366 | -66.3677 | 110.5853 |
| 102.100.100/13367 | -66.3677 | 110.5853 |
| 102.100.100/13368 | -66.3677 | 110.5853 |
| 102.100.100/13369 | -66.3677 | 110.5853 |
| 102.100.100/13370 | -66.3677 | 110.5853 |
| 102.100.100/13371 | -66.3677 | 110.5853 |
| 102.100.100/13372 | -66.3677 | 110.5853 |
| 102.100.100/13373 | -66.3677 | 110.5853 |
| 102.100.100/13374 | -66.3678 | 110.5854 |
| 102.100.100/13375 | -66.3678 | 110.5853 |
| 102.100.100/13376 | -66.3678 | 110.5853 |
| 102.100.100/13377 | -66.3678 | 110.5854 |
| 102.100.100/13378 | -66.3678 | 110.5853 |
| 102.100.100/13379 | -66.3678 | 110.5853 |
| 102.100.100/13380 | -66.3679 | 110.5854 |
| 102.100.100/13381 | -66.3679 | 110.5854 |
| 102.100.100/13382 | -66.3679 | 110.5853 |
| 102.100.100/13383 | -66.3681 | 110.5856 |
| 102.100.100/13385 | -66.3681 | 110.5855 |
| 102.100.100/13386 | -66.3686 | 110.5857 |
| 102.100.100/13387 | -66.3686 | 110.5857 |
| 102.100.100/13388 | -66.3686 | 110.5857 |
| 102.100.100/13389 | -66.3686 | 110.5857 |
| 102.100.100/13390 | -66.3686 | 110.5856 |
| 102.100.100/13391 | -66.3686 | 110.5856 |
| 102.100.100/13392 | -66.3686 | 110.5856 |
| 102.100.100/13393 | -66.3686 | 110.5856 |
| 102.100.100/13394 | -66.3686 | 110.5856 |
| 102.100.100/13395 | -66.3686 | 110.5856 |
| 102.100.100/13396 | -66.3686 | 110.5856 |
| 102.100.100/13397 | -66.3686 | 110.5856 |
| 102.100.100/13398 | -66.3686 | 110.5857 |
| 102.100.100/13399 | -66.3686 | 110.5856 |
| 102.100.100/13400 | -66.3686 | 110.5856 |
| 102.100.100/13401 | -66.3687 | 110.5856 |
| 102.100.100/13402 | -66.3687 | 110.5856 |
| 102.100.100/13403 | -66.3686 | 110.5856 |
| 102.100.100/13404 | -66.3687 | 110.5856 |
| 102.100.100/13405 | -66.3687 | 110.5856 |
| 102.100.100/13406 | -66.369 | 110.5856 |
| 102.100.100/13407 | -66.369 | 110.5856 |
| 102.100.100/13408 | -66.3688 | 110.5856 |
| 102.100.100/13409 | -66.369 | 110.5855 |
| 102.100.100/13410 | -66.3695 | 110.5856 |
| 102.100.100/13411 | -66.3695 | 110.5856 |
| 102.100.100/13412 | -66.3695 | 110.5856 |
| 102.100.100/13413 | -66.3695 | 110.5856 |
| 102.100.100/13414 | -66.3695 | 110.5856 |
| 102.100.100/13415 | -66.3695 | 110.5856 |
| 102.100.100/13416 | -66.3695 | 110.5856 |
| 102.100.100/13417 | -66.3695 | 110.5856 |
| 102.100.100/13418 | -66.3694 | 110.5855 |
| 102.100.100/13419 | -66.3695 | 110.5855 |
| 102.100.100/13420 | -66.3695 | 110.5855 |
| 102.100.100/13421 | -66.3695 | 110.5855 |
| 102.100.100/13422 | -66.3695 | 110.5856 |
| 102.100.100/13423 | -66.3695 | 110.5856 |
| 102.100.100/13424 | -66.3695 | 110.5855 |
| 102.100.100/13425 | -66.3695 | 110.5856 |
| 102.100.100/13426 | -66.3695 | 110.5856 |
| 102.100.100/13427 | -66.3695 | 110.5855 |
| 102.100.100/13428 | -66.3696 | 110.5856 |
| 102.100.100/13429 | -66.3696 | 110.5856 |
| 102.100.100/13430 | -66.3696 | 110.5855 |
| 102.100.100/13431 | -66.3699 | 110.5856 |
| 102.100.100/13432 | -66.3699 | 110.5856 |
| 102.100.100/13433 | -66.3699 | 110.5855 |
| 102.100.100/13434 | -66.3703 | 110.5856 |
| 102.100.100/13435 | -66.3703 | 110.5856 |
| 102.100.100/13436 | -66.3703 | 110.5855 |
| 102.100.100/13437 | -66.2822 | 110.5265 |
| 102.100.100/13438 | -66.2822 | 110.5265 |
| 102.100.100/13439 | -66.2822 | 110.5265 |
| 102.100.100/13440 | -66.2822 | 110.5265 |
| 102.100.100/13441 | -66.2822 | 110.5264 |
| 102.100.100/13442 | -66.2822 | 110.5263 |
| 102.100.100/13443 | -66.2821 | 110.5262 |
| 102.100.100/13444 | -66.2818 | 110.5252 |
| 102.100.100/13445 | -66.2818 | 110.5252 |
| 102.100.100/13446 | -66.2818 | 110.5252 |
| 102.100.100/13447 | -66.2818 | 110.5252 |
| 102.100.100/13448 | -66.2817 | 110.5249 |
| 102.100.100/13449 | -66.2815 | 110.5245 |
| 102.100.100/13450 | -66.2822 | 110.5265 |
| 102.100.100/13451 | -66.2822 | 110.5265 |
| 102.100.100/13452 | -66.2822 | 110.5265 |
| 102.100.100/13453 | -66.2822 | 110.5264 |
| 102.100.100/13454 | -66.2822 | 110.5263 |
| 102.100.100/13455 | -66.2822 | 110.5261 |
| 102.100.100/13456 | -66.282 | 110.5256 |
| 102.100.100/13457 | -66.2819 | 110.5252 |
| 102.100.100/13458 | -66.2819 | 110.5252 |
| 102.100.100/13459 | -66.2819 | 110.5252 |
| 102.100.100/13460 | -66.2815 | 110.5245 |
| 102.100.100/13461 | -66.2823 | 110.5265 |
| 102.100.100/13462 | -66.2823 | 110.5265 |
| 102.100.100/13463 | -66.2823 | 110.5265 |
| 102.100.100/13464 | -66.2822 | 110.5264 |
| 102.100.100/13465 | -66.2822 | 110.5263 |
| 102.100.100/13466 | -66.2822 | 110.5261 |
| 102.100.100/13467 | -66.282 | 110.5255 |
| 102.100.100/13468 | -66.2819 | 110.5252 |
| 102.100.100/13469 | -66.2819 | 110.5252 |
| 102.100.100/13470 | -66.2819 | 110.5252 |
| 102.100.100/13471 | -66.2819 | 110.5252 |
| 102.100.100/13472 | -66.2815 | 110.5245 |
| 102.100.100/13473 | -66.4725 | 110.5496 |
| 102.100.100/13474 | -66.4725 | 110.5496 |
| 102.100.100/13475 | -66.4725 | 110.5496 |
| 102.100.100/13476 | -66.4725 | 110.5495 |
| 102.100.100/13477 | -66.4724 | 110.5495 |
| 102.100.100/13478 | -66.4724 | 110.5494 |
| 102.100.100/13479 | -66.4724 | 110.5492 |
| 102.100.100/13480 | -66.4723 | 110.5485 |
| 102.100.100/13481 | -66.4721 | 110.5476 |
| 102.100.100/13482 | -66.4721 | 110.5476 |
| 102.100.100/13483 | -66.4721 | 110.5476 |
| 102.100.100/13484 | -66.4721 | 110.5475 |
| 102.100.100/13485 | -66.4721 | 110.5475 |
| 102.100.100/13486 | -66.4721 | 110.5474 |
| 102.100.100/13487 | -66.472 | 110.5472 |
| 102.100.100/13488 | -66.4719 | 110.5466 |
| 102.100.100/13489 | -66.4717 | 110.5455 |
| 102.100.100/13490 | -66.4717 | 110.5455 |
| 102.100.100/13491 | -66.4717 | 110.5455 |
| 102.100.100/13492 | -66.4717 | 110.5455 |
| 102.100.100/13493 | -66.4717 | 110.5454 |
| 102.100.100/13494 | -66.4717 | 110.5453 |
| 102.100.100/13495 | -66.4717 | 110.5451 |
| 102.100.100/13496 | -66.4715 | 110.5445 |
| 102.100.100/13497 | -66.4714 | 110.5435 |
| 102.100.100/13498 | -66.4724 | 110.5496 |
| 102.100.100/13499 | -66.4724 | 110.5496 |
| 102.100.100/13500 | -66.4724 | 110.5496 |
| 102.100.100/13501 | -66.4724 | 110.5496 |
| 102.100.100/13502 | -66.4724 | 110.5495 |
| 102.100.100/13503 | -66.4724 | 110.5494 |
| 102.100.100/13504 | -66.4724 | 110.5492 |
| 102.100.100/13505 | -66.4723 | 110.5486 |
| 102.100.100/13506 | -66.4721 | 110.5476 |
| 102.100.100/13507 | -66.4721 | 110.5476 |
| 102.100.100/13508 | -66.4721 | 110.5476 |
| 102.100.100/13509 | -66.4721 | 110.5476 |
| 102.100.100/13510 | -66.4721 | 110.5475 |
| 102.100.100/13511 | -66.472 | 110.5474 |
| 102.100.100/13512 | -66.472 | 110.5472 |
| 102.100.100/13513 | -66.4719 | 110.5466 |
| 102.100.100/13514 | -66.4717 | 110.5455 |
| 102.100.100/13515 | -66.4717 | 110.5455 |
| 102.100.100/13516 | -66.4717 | 110.5455 |
| 102.100.100/13517 | -66.4717 | 110.5455 |
| 102.100.100/13518 | -66.4717 | 110.5454 |
| 102.100.100/13519 | -66.4717 | 110.5453 |
| 102.100.100/13520 | -66.4716 | 110.5451 |
| 102.100.100/13521 | -66.4715 | 110.5445 |
| 102.100.100/13522 | -66.4714 | 110.5435 |
| 102.100.100/13523 | -66.4724 | 110.5496 |
| 102.100.100/13524 | -66.4724 | 110.5496 |
| 102.100.100/13525 | -66.4724 | 110.5496 |
| 102.100.100/13526 | -66.4724 | 110.5496 |
| 102.100.100/13527 | -66.4724 | 110.5495 |
| 102.100.100/13528 | -66.4724 | 110.5494 |
| 102.100.100/13529 | -66.4724 | 110.5492 |
| 102.100.100/13530 | -66.4723 | 110.5486 |
| 102.100.100/13531 | -66.4721 | 110.5476 |
| 102.100.100/13532 | -66.4721 | 110.5476 |
| 102.100.100/13533 | -66.4721 | 110.5476 |
| 102.100.100/13534 | -66.4721 | 110.5476 |
| 102.100.100/13535 | -66.472 | 110.5475 |
| 102.100.100/13536 | -66.472 | 110.5474 |
| 102.100.100/13537 | -66.472 | 110.5472 |
| 102.100.100/13538 | -66.4719 | 110.5466 |
| 102.100.100/13539 | -66.4717 | 110.5455 |
| 102.100.100/13540 | -66.4717 | 110.5455 |
| 102.100.100/13541 | -66.4717 | 110.5455 |
| 102.100.100/13542 | -66.4717 | 110.5455 |
| 102.100.100/13543 | -66.4717 | 110.5454 |
| 102.100.100/13544 | -66.4717 | 110.5453 |
| 102.100.100/13545 | -66.4716 | 110.5451 |
| 102.100.100/13546 | -66.4715 | 110.5445 |
| 102.100.100/13547 | -66.4713 | 110.5435 |
| 102.100.100/13548 | -66.4102 | 110.6549 |
| 102.100.100/13549 | -66.4102 | 110.6549 |
| 102.100.100/13550 | -66.4102 | 110.6549 |
| 102.100.100/13551 | -66.4102 | 110.6549 |
| 102.100.100/13552 | -66.4102 | 110.6548 |
| 102.100.100/13553 | -66.4102 | 110.6548 |
| 102.100.100/13555 | -66.4102 | 110.6548 |
| 102.100.100/13556 | -66.4102 | 110.6548 |
| 102.100.100/13557 | -66.4102 | 110.6548 |
| 102.100.100/13558 | -66.4102 | 110.6548 |
| 102.100.100/13559 | -66.4102 | 110.6548 |
| 102.100.100/13560 | -66.4102 | 110.655 |
| 102.100.100/13561 | -66.4102 | 110.6549 |
| 102.100.100/13562 | -66.4103 | 110.6549 |
| 102.100.100/13563 | -66.4103 | 110.655 |
| 102.100.100/13564 | -66.4103 | 110.655 |
| 102.100.100/13565 | -66.4103 | 110.655 |
| 102.100.100/13567 | -66.4103 | 110.6552 |
| 102.100.100/13568 | -66.4103 | 110.6551 |
| 102.100.100/13569 | -66.4105 | 110.6557 |
| 102.100.100/13570 | -66.4105 | 110.6557 |
| 102.100.100/13571 | -66.4105 | 110.6557 |
| 102.100.100/13572 | -66.4108 | 110.6566 |
| 102.100.100/13573 | -66.4108 | 110.6566 |
| 102.100.100/13574 | -66.4108 | 110.6566 |
| 102.100.100/13575 | -66.4108 | 110.6566 |
| 102.100.100/13576 | -66.4108 | 110.6565 |
| 102.100.100/13577 | -66.4108 | 110.6565 |
| 102.100.100/13578 | -66.4108 | 110.6565 |
| 102.100.100/13579 | -66.4108 | 110.6565 |
| 102.100.100/13580 | -66.4108 | 110.6565 |
| 102.100.100/13581 | -66.4108 | 110.6565 |
| 102.100.100/13582 | -66.4108 | 110.6565 |
| 102.100.100/13583 | -66.4108 | 110.6565 |
| 102.100.100/13584 | -66.4108 | 110.6566 |
| 102.100.100/13585 | -66.4108 | 110.6566 |
| 102.100.100/13586 | -66.4108 | 110.6566 |
| 102.100.100/13587 | -66.4108 | 110.6567 |
| 102.100.100/13588 | -66.4109 | 110.6567 |
| 102.100.100/13589 | -66.4109 | 110.6567 |
| 102.100.100/13590 | -66.4109 | 110.6569 |
| 102.100.100/13591 | -66.4109 | 110.6569 |
| 102.100.100/13592 | -66.4109 | 110.6568 |
| 102.100.100/13593 | -66.4111 | 110.6574 |
| 102.100.100/13594 | -66.4111 | 110.6574 |
| 102.100.100/13595 | -66.4111 | 110.6574 |
| 102.100.100/13596 | -66.4113 | 110.6583 |
| 102.100.100/13597 | -66.4113 | 110.6583 |
| 102.100.100/13598 | -66.4114 | 110.6583 |
| 102.100.100/13599 | -66.4114 | 110.6583 |
| 102.100.100/13600 | -66.4114 | 110.6582 |
| 102.100.100/13601 | -66.4114 | 110.6582 |
| 102.100.100/13602 | -66.4114 | 110.6582 |
| 102.100.100/13603 | -66.4114 | 110.6583 |
| 102.100.100/13604 | -66.4114 | 110.6582 |
| 102.100.100/13605 | -66.4114 | 110.6582 |
| 102.100.100/13606 | -66.4114 | 110.6582 |
| 102.100.100/13607 | -66.4114 | 110.6582 |
| 102.100.100/13608 | -66.4114 | 110.6584 |
| 102.100.100/13609 | -66.4114 | 110.6583 |
| 102.100.100/13610 | -66.4114 | 110.6583 |
| 102.100.100/13611 | -66.4114 | 110.6584 |
| 102.100.100/13612 | -66.4114 | 110.6584 |
| 102.100.100/13613 | -66.4114 | 110.6584 |
| 102.100.100/13614 | -66.4115 | 110.6586 |
| 102.100.100/13615 | -66.4115 | 110.6586 |
| 102.100.100/13616 | -66.4115 | 110.6586 |
| 102.100.100/13617 | -66.4116 | 110.6591 |
| 102.100.100/13618 | -66.4116 | 110.6591 |
| 102.100.100/13619 | -66.4117 | 110.6591 |
| 102.100.100/13620 | -66.4119 | 110.66 |
| 102.100.100/13621 | -66.4119 | 110.66 |
| 102.100.100/13622 | -66.4119 | 110.66 |
| 102.100.100/13729 | -30.1919 | 120.6551 |
| 102.100.100/13730 | -30.1919 | 120.6551 |
| 102.100.100/13731 | -30.1916 | 120.6517 |
| 102.100.100/13732 | -30.1916 | 120.6517 |
| 102.100.100/13733 | -30.1919 | 120.6425 |
| 102.100.100/13735 | -30.1944 | 120.6337 |
| 102.100.100/13736 | -30.1944 | 120.6337 |
| 102.100.100/13737 | -15.2586 | 132.3705 |
| 102.100.100/13738 | -15.2586 | 132.3705 |
| 102.100.100/13739 | -13.1791 | 130.7944 |
| 102.100.100/13740 | -13.1791 | 130.7944 |
| 102.100.100/13892 | -35.6083 | 138.2613 |
| 102.100.100/13893 | -35.2721 | 138.6902 |
| 102.100.100/13894 | -32.8285 | 138.0327 |
| 102.100.100/13895 | -32.7484 | 138.1363 |
| 102.100.100/13896 | -33.9142 | 138.6038 |
| 102.100.100/13897 | -34.609 | 138.8613 |
| 102.100.100/13898 | -34.5769 | 139.0061 |
| 102.100.100/13899 | -34.6831 | 138.9086 |
| 102.100.100/13900 | -34.8808 | 138.7083 |
| 102.100.100/13901 | -34.9334 | 138.727 |
| 102.100.100/13902 | -32.321 | 137.9544 |
| 102.100.100/13903 | -30.7757 | 138.7976 |
| 102.100.100/13904 | -31.3277 | 138.5673 |
| 102.100.100/13905 | -31.5439 | 138.5946 |
| 102.100.100/13906 | -35.8748 | 149.0117 |
| 102.100.100/13907 | -35.8748 | 149.0117 |
| 102.100.100/14181 | -24.8384 | 147.7814 |
| 102.100.100/14182 | -24.8384 | 147.7814 |
| 102.100.100/14183 | -24.8538 | 147.5212 |
| 102.100.100/14184 | -24.8538 | 147.5212 |
| 102.100.100/14185 | -41.6556 | 145.0819 |
| 102.100.100/14186 | -41.6556 | 145.0819 |
| 102.100.100/14187 | -41.5837 | 145.0931 |
| 102.100.100/14188 | -41.5837 | 145.0931 |
| 102.100.100/15823 | -28.2298 | 152.4244 |
| 102.100.100/15824 | -28.2298 | 152.4244 |
| 102.100.100/15825 | -28.2887 | 152.4247 |
| 102.100.100/15826 | -28.2887 | 152.4247 |
| 102.100.100/15827 | -28.2276 | 152.4269 |
| 102.100.100/15828 | -28.2276 | 152.4269 |
| 102.100.100/15829 | -28.2276 | 152.4272 |
| 102.100.100/15830 | -28.2276 | 152.4272 |
| 102.100.100/15861 | -13.6788 | 143.141 |
| 102.100.100/15862 | -13.6788 | 143.141 |
| 102.100.100/15863 | -13.6812 | 143.14 |
| 102.100.100/15864 | -13.6812 | 143.14 |
| 102.100.100/15865 | -13.8347 | 143.0981 |
| 102.100.100/15866 | -13.8347 | 143.0981 |
| 102.100.100/15867 | -13.8299 | 143.0986 |
| 102.100.100/15868 | -13.8299 | 143.0986 |
| 102.100.100/15869 | -13.534 | 143.0477 |
| 102.100.100/15870 | -13.534 | 143.0477 |
| 102.100.100/15871 | -13.5324 | 143.0475 |
| 102.100.100/15872 | -13.5324 | 143.0475 |
| 102.100.100/15873 | -13.5153 | 143.0416 |
| 102.100.100/15874 | -13.5153 | 143.0416 |
| 102.100.100/15877 | -13.7723 | 141.8882 |
| 102.100.100/15878 | -13.7723 | 141.8882 |
| 102.100.100/15879 | -13.7701 | 141.8905 |
| 102.100.100/15880 | -13.7701 | 141.8905 |
| 102.100.100/15881 | -13.6025 | 141.6811 |
| 102.100.100/15882 | -13.6025 | 141.6811 |
| 102.100.100/15883 | -13.6032 | 141.6841 |
| 102.100.100/15884 | -13.6032 | 141.6841 |
| 102.100.100/15885 | -13.7418 | 141.5068 |
| 102.100.100/15886 | -13.7418 | 141.5068 |
| 102.100.100/15887 | -13.7477 | 141.5062 |
| 102.100.100/15888 | -13.7477 | 141.5062 |
| 102.100.100/15891 | -13.8439 | 141.6164 |
| 102.100.100/15892 | -13.8439 | 141.6164 |
| 102.100.100/15895 | -13.9747 | 141.6526 |
| 102.100.100/15896 | -13.9747 | 141.6526 |
| 102.100.100/15943 | -42.3846 | 147.3106 |
| 102.100.100/15944 | -42.3846 | 147.3106 |
| 102.100.100/15945 | -42.3843 | 147.3109 |
| 102.100.100/15946 | -42.3843 | 147.3109 |
| 102.100.100/15947 | -42.4042 | 147.4077 |
| 102.100.100/15948 | -42.4042 | 147.4077 |
| 102.100.100/15949 | -42.3285 | 147.3976 |
| 102.100.100/15950 | -42.3285 | 147.3976 |
| 102.100.100/15951 | -42.2844 | 147.4015 |
| 102.100.100/15952 | -42.2844 | 147.4015 |
| 102.100.100/15953 | -42.2423 | 147.4597 |
| 102.100.100/15954 | -42.2423 | 147.4597 |
| 102.100.100/15955 | -42.8223 | 147.5057 |
| 102.100.100/15956 | -42.8223 | 147.5057 |
| 102.100.100/15957 | -42.279 | 147.6145 |
| 102.100.100/15958 | -42.279 | 147.6145 |
| 102.100.100/15959 | -42.8072 | 147.4398 |
| 102.100.100/15960 | -42.8072 | 147.4398 |
| 102.100.100/15961 | -42.6174 | 147.4226 |
| 102.100.100/15962 | -42.6174 | 147.4226 |
| 102.100.100/15963 | -42.6178 | 147.4223 |
| 102.100.100/15964 | -42.6178 | 147.4223 |
| 102.100.100/15965 | -42.5452 | 147.4561 |
| 102.100.100/15966 | -42.5452 | 147.4561 |
| 102.100.100/15967 | -42.8074 | 147.4391 |
| 102.100.100/15968 | -42.8074 | 147.4391 |
| 102.100.100/15969 | -42.3878 | 147.0474 |
| 102.100.100/15970 | -42.3878 | 147.0474 |
| 102.100.100/15971 | -42.3985 | 147.0791 |
| 102.100.100/15972 | -42.3985 | 147.0791 |
| 102.100.100/15973 | -42.3984 | 147.0799 |
| 102.100.100/15974 | -42.3984 | 147.0799 |
| 102.100.100/15975 | -42.2734 | 146.8948 |
| 102.100.100/15976 | -42.2734 | 146.8948 |
| 102.100.100/15977 | -42.2715 | 146.8927 |
| 102.100.100/15978 | -42.2715 | 146.8927 |
| 102.100.100/15979 | -42.2703 | 146.8964 |
| 102.100.100/15980 | -42.2703 | 146.8964 |
| 102.100.100/15981 | -42.556 | 147.2005 |
| 102.100.100/15982 | -42.556 | 147.2005 |
| 102.100.100/15983 | -42.5591 | 147.2065 |
| 102.100.100/15984 | -42.5591 | 147.2065 |
| 102.100.100/15985 | -42.3868 | 147.016 |
| 102.100.100/15986 | -42.3868 | 147.016 |
| 102.100.100/15987 | -42.4018 | 147.0196 |
| 102.100.100/15988 | -42.4018 | 147.0196 |
| 102.100.100/15989 | -42.4032 | 147.0124 |
| 102.100.100/15990 | -42.4032 | 147.0124 |
| 102.100.100/15991 | -42.4269 | 146.6994 |
| 102.100.100/15992 | -42.4269 | 146.6994 |
| 102.100.100/15993 | -42.5465 | 146.8494 |
| 102.100.100/15994 | -42.5465 | 146.8494 |
| 102.100.100/15995 | -42.5469 | 146.8485 |
| 102.100.100/15996 | -42.5469 | 146.8485 |
| 102.100.100/15997 | -42.5726 | 146.7729 |
| 102.100.100/15998 | -42.5726 | 146.7729 |
| 102.100.100/15999 | -42.5705 | 146.771 |
| 102.100.100/16000 | -42.5705 | 146.771 |
| 102.100.100/16001 | -41.7775 | 147.3189 |
| 102.100.100/16002 | -41.7775 | 147.3189 |
| 102.100.100/16003 | -41.758 | 147.1713 |
| 102.100.100/16004 | -41.758 | 147.1713 |
| 102.100.100/16005 | -41.7582 | 147.1716 |
| 102.100.100/16006 | -41.7582 | 147.1716 |
| 102.100.100/16007 | -42.2796 | 147.4684 |
| 102.100.100/16008 | -42.2796 | 147.4684 |
| 102.100.100/16009 | -42.2766 | 147.466 |
| 102.100.100/16010 | -42.2766 | 147.466 |
| 102.100.100/16011 | -42.2794 | 147.6138 |
| 102.100.100/16012 | -42.2794 | 147.6138 |
| 102.100.100/16013 | -42.0154 | 147.491 |
| 102.100.100/16014 | -42.0154 | 147.491 |
| 102.100.100/19231 | -35.6407 | 138.1301 |
| 102.100.100/19232 | -35.6407 | 138.1301 |
| 102.100.100/19233 | -35.6401 | 138.1301 |
| 102.100.100/19234 | -35.6401 | 138.1301 |
| 102.100.100/19235 | -35.6407 | 138.1292 |
| 102.100.100/19236 | -35.6407 | 138.1292 |
| 102.100.100/19237 | -35.6387 | 138.1254 |
| 102.100.100/19238 | -35.6387 | 138.1254 |
| 102.100.100/19239 | -35.6387 | 138.1258 |
| 102.100.100/19240 | -35.6387 | 138.1258 |
| 102.100.100/19241 | -35.6382 | 138.1264 |
| 102.100.100/19242 | -35.6382 | 138.1264 |
| 102.100.100/19243 | -35.6373 | 138.1221 |
| 102.100.100/19244 | -35.6373 | 138.1221 |
| 102.100.100/19245 | -35.637 | 138.1221 |
| 102.100.100/19246 | -35.637 | 138.1221 |
| 102.100.100/19247 | -35.6363 | 138.1219 |
| 102.100.100/19248 | -35.6363 | 138.1219 |
| 102.100.100/19249 | -35.6347 | 138.1198 |
| 102.100.100/19250 | -35.6347 | 138.1198 |
| 102.100.100/19251 | -35.6348 | 138.1202 |
| 102.100.100/19252 | -35.6348 | 138.1202 |
| 102.100.100/19253 | -35.6346 | 138.1202 |
| 102.100.100/19254 | -35.6346 | 138.1202 |
| 102.100.100/19255 | -35.6352 | 138.119 |
| 102.100.100/19256 | -35.6352 | 138.119 |
| 102.100.100/19257 | -35.6348 | 138.1188 |
| 102.100.100/19258 | -35.6348 | 138.1188 |
| 102.100.100/19259 | -35.6352 | 138.1184 |
| 102.100.100/19260 | -35.6352 | 138.1184 |
| 102.100.100/19261 | -35.6356 | 138.1207 |
| 102.100.100/19262 | -35.6356 | 138.1207 |
| 102.100.100/19263 | -35.6353 | 138.1209 |
| 102.100.100/19264 | -35.6353 | 138.1209 |
| 102.100.100/19265 | -35.6364 | 138.1206 |
| 102.100.100/19266 | -35.6364 | 138.1206 |
| 102.100.100/19267 | -35.6314 | 138.1167 |
| 102.100.100/19268 | -35.6314 | 138.1167 |
| 102.100.100/19269 | -35.6327 | 138.1164 |
| 102.100.100/19270 | -35.6327 | 138.1164 |
| 102.100.100/19271 | -35.6304 | 138.1162 |
| 102.100.100/19272 | -35.6304 | 138.1162 |
| 102.100.100/19273 | -35.655 | 138.1623 |
| 102.100.100/19274 | -35.655 | 138.1623 |
| 102.100.100/19275 | -35.655 | 138.1629 |
| 102.100.100/19276 | -35.655 | 138.1629 |
| 102.100.100/19277 | -35.6555 | 138.1628 |
| 102.100.100/19278 | -35.6555 | 138.1628 |
| 102.100.100/19279 | -35.6554 | 138.1626 |
| 102.100.100/19280 | -35.6554 | 138.1626 |
| 102.100.100/19281 | -35.0917 | 138.6546 |
| 102.100.100/19282 | -35.0917 | 138.6546 |
| 102.100.100/19283 | -35.091 | 138.6548 |
| 102.100.100/19284 | -35.091 | 138.6548 |
| 102.100.100/19285 | -35.0907 | 138.6549 |
| 102.100.100/19286 | -35.0907 | 138.6549 |
| 102.100.100/19287 | -35.0854 | 138.6589 |
| 102.100.100/19288 | -35.0854 | 138.6589 |
| 102.100.100/19289 | -35.0854 | 138.6591 |
| 102.100.100/19290 | -35.0854 | 138.6591 |
| 102.100.100/19291 | -35.0857 | 138.6594 |
| 102.100.100/19292 | -35.0857 | 138.6594 |
| 102.100.100/19293 | -35.0973 | 138.6711 |
| 102.100.100/19294 | -35.0973 | 138.6711 |
| 102.100.100/19295 | -35.0974 | 138.6707 |
| 102.100.100/19296 | -35.0974 | 138.6707 |
| 102.100.100/19297 | -35.097 | 138.671 |
| 102.100.100/19298 | -35.097 | 138.671 |
| 102.100.100/19299 | -35.0982 | 138.6777 |
| 102.100.100/19300 | -35.0982 | 138.6777 |
| 102.100.100/19301 | -35.098 | 138.6777 |
| 102.100.100/19302 | -35.0984 | 138.6777 |
| 102.100.100/19303 | -35.098 | 138.6776 |
| 102.100.100/19304 | -35.098 | 138.6776 |
| 102.100.100/19305 | -35.103 | 138.6844 |
| 102.100.100/19306 | -35.103 | 138.6844 |
| 102.100.100/19307 | -35.1031 | 138.6849 |
| 102.100.100/19308 | -35.1031 | 138.6849 |
| 102.100.100/19309 | -35.103 | 138.6851 |
| 102.100.100/19310 | -35.103 | 138.6851 |
| 102.100.100/19311 | -35.0917 | 138.6762 |
| 102.100.100/19312 | -35.0917 | 138.6762 |
| 102.100.100/19313 | -35.0916 | 138.6762 |
| 102.100.100/19314 | -35.0916 | 138.6762 |
| 102.100.100/19315 | -35.0913 | 138.6761 |
| 102.100.100/19316 | -35.0913 | 138.6761 |
| 102.100.100/19317 | -35.1021 | 138.6507 |
| 102.100.100/19318 | -35.1021 | 138.6507 |
| 102.100.100/19319 | -35.1019 | 138.6507 |
| 102.100.100/19320 | -35.1019 | 138.6507 |
| 102.100.100/19321 | -35.102 | 138.6503 |
| 102.100.100/19322 | -35.102 | 138.6503 |
| 102.100.100/19323 | -35.0814 | 138.6586 |
| 102.100.100/19324 | -35.0814 | 138.6586 |
| 102.100.100/19325 | -35.0814 | 138.6582 |
| 102.100.100/19326 | -35.0814 | 138.6582 |
| 102.100.100/19327 | -35.0816 | 138.6576 |
| 102.100.100/19328 | -35.0816 | 138.6576 |
| 102.100.100/19329 | -35.0815 | 138.6572 |
| 102.100.100/19330 | -35.0815 | 138.6572 |
| 102.100.100/19381 | -68.5516 | 78.01818 |
| 102.100.100/19382 | -68.5516 | 78.01821 |
| 102.100.100/19383 | -68.5516 | 78.01825 |
| 102.100.100/19384 | -68.5516 | 78.01822 |
| 102.100.100/19385 | -68.5516 | 78.01824 |
| 102.100.100/19386 | -68.5516 | 78.01829 |
| 102.100.100/19387 | -68.5509 | 78.01969 |
| 102.100.100/19388 | -68.5509 | 78.01974 |
| 102.100.100/19389 | -68.5509 | 78.01978 |
| 102.100.100/19390 | -68.5509 | 78.01972 |
| 102.100.100/19391 | -68.5509 | 78.01976 |
| 102.100.100/19392 | -68.5509 | 78.0198 |
| 102.100.100/19393 | -68.5502 | 78.02103 |
| 102.100.100/19394 | -68.5502 | 78.02108 |
| 102.100.100/19395 | -68.5502 | 78.02111 |
| 102.100.100/19396 | -68.5502 | 78.02106 |
| 102.100.100/19397 | -68.5502 | 78.02111 |
| 102.100.100/19398 | -68.5502 | 78.02114 |
| 102.100.100/19399 | -68.6001 | 77.95221 |
| 102.100.100/19400 | -68.6001 | 77.95223 |
| 102.100.100/19401 | -68.6001 | 77.95225 |
| 102.100.100/19402 | -68.6001 | 77.95225 |
| 102.100.100/19403 | -68.6001 | 77.95227 |
| 102.100.100/19404 | -68.6001 | 77.95229 |
| 102.100.100/19405 | -68.5997 | 77.95439 |
| 102.100.100/19406 | -68.5997 | 77.95441 |
| 102.100.100/19407 | -68.5997 | 77.95444 |
| 102.100.100/19408 | -68.5997 | 77.95444 |
| 102.100.100/19409 | -68.5997 | 77.95446 |
| 102.100.100/19410 | -68.5997 | 77.95448 |
| 102.100.100/19411 | -68.5993 | 77.95659 |
| 102.100.100/19412 | -68.5993 | 77.95662 |
| 102.100.100/19413 | -68.5993 | 77.95664 |
| 102.100.100/19414 | -68.5993 | 77.95664 |
| 102.100.100/19415 | -68.5993 | 77.95666 |
| 102.100.100/19416 | -68.5993 | 77.95669 |
| 102.100.100/19417 | -68.4938 | 78.10926 |
| 102.100.100/19418 | -68.4938 | 78.1093 |
| 102.100.100/19419 | -68.4938 | 78.10934 |
| 102.100.100/19420 | -68.4938 | 78.1093 |
| 102.100.100/19421 | -68.4938 | 78.10933 |
| 102.100.100/19422 | -68.4938 | 78.10938 |
| 102.100.100/19423 | -68.4932 | 78.11098 |
| 102.100.100/19424 | -68.4932 | 78.111 |
| 102.100.100/19425 | -68.4932 | 78.11106 |
| 102.100.100/19426 | -68.4932 | 78.11102 |
| 102.100.100/19427 | -68.4932 | 78.11104 |
| 102.100.100/19428 | -68.4932 | 78.11109 |
| 102.100.100/19429 | -68.4927 | 78.11303 |
| 102.100.100/19430 | -68.4927 | 78.11306 |
| 102.100.100/19431 | -68.4927 | 78.11308 |
| 102.100.100/19432 | -68.4927 | 78.11307 |
| 102.100.100/19433 | -68.4927 | 78.1131 |
| 102.100.100/19434 | -68.4927 | 78.11312 |
| 102.100.100/19435 | -68.5778 | 78.00607 |
| 102.100.100/19436 | -68.5778 | 78.00603 |
| 102.100.100/19437 | -68.5778 | 78.00596 |
| 102.100.100/19438 | -68.5779 | 78.00606 |
| 102.100.100/19439 | -68.5778 | 78.00601 |
| 102.100.100/19440 | -68.5778 | 78.00595 |
| 102.100.100/19441 | -68.5787 | 78.00531 |
| 102.100.100/19442 | -68.5787 | 78.00527 |
| 102.100.100/19443 | -68.5787 | 78.00523 |
| 102.100.100/19444 | -68.5787 | 78.0053 |
| 102.100.100/19445 | -68.5787 | 78.00525 |
| 102.100.100/19446 | -68.5787 | 78.00521 |
| 102.100.100/19447 | -68.5795 | 78.00456 |
| 102.100.100/19448 | -68.5795 | 78.0045 |
| 102.100.100/19449 | -68.5795 | 78.00446 |
| 102.100.100/19450 | -68.5795 | 78.00453 |
| 102.100.100/19451 | -68.5795 | 78.00448 |
| 102.100.100/19452 | -68.5795 | 78.00444 |
| 102.100.100/19453 | -33.6096 | 150.7373 |
| 102.100.100/19454 | -33.6096 | 150.7373 |
| 102.100.100/19455 | -33.6101 | 150.7369 |
| 102.100.100/19456 | -33.6101 | 150.7369 |
| 102.100.100/19457 | -33.6123 | 150.739 |
| 102.100.100/19458 | -33.6123 | 150.739 |
| 102.100.100/19459 | -33.6129 | 150.738 |
| 102.100.100/19460 | -33.6129 | 150.738 |
| 102.100.100/19461 | -33.6126 | 150.7377 |
| 102.100.100/19462 | -33.6126 | 150.7377 |
| 102.100.100/19463 | -33.6157 | 150.7362 |
| 102.100.100/19464 | -33.6157 | 150.7362 |
| 102.100.100/19465 | -33.6162 | 150.736 |
| 102.100.100/19466 | -33.6162 | 150.736 |
| 102.100.100/19467 | -33.6175 | 150.7361 |
| 102.100.100/19468 | -33.6175 | 150.7361 |
| 102.100.100/19469 | -33.621 | 150.7339 |
| 102.100.100/19470 | -33.621 | 150.7339 |
| 102.100.100/19471 | -33.6207 | 150.7338 |
| 102.100.100/19472 | -33.6207 | 150.7338 |
| 102.100.100/19473 | -33.6208 | 150.7342 |
| 102.100.100/19474 | -33.6208 | 150.7342 |
| 102.100.100/19475 | -33.6213 | 150.7354 |
| 102.100.100/19476 | -33.6213 | 150.7354 |
| 102.100.100/19477 | -33.6208 | 150.7358 |
| 102.100.100/19478 | -33.6208 | 150.7358 |
| 102.100.100/19479 | -33.6152 | 150.7227 |
| 102.100.100/19480 | -33.6152 | 150.7227 |
| 102.100.100/19481 | -33.6158 | 150.7248 |
| 102.100.100/19482 | -33.6158 | 150.7248 |
| 102.100.100/19483 | -33.6135 | 150.7249 |
| 102.100.100/19484 | -33.6135 | 150.7249 |
| 102.100.100/19485 | -33.6129 | 150.7298 |
| 102.100.100/19486 | -33.6129 | 150.7298 |
| 102.100.100/19487 | -33.6047 | 150.7304 |
| 102.100.100/19488 | -33.6047 | 150.7304 |
| 102.100.100/19489 | -33.6052 | 150.7298 |
| 102.100.100/19490 | -33.6052 | 150.7298 |
| 102.100.100/19491 | -33.6062 | 150.7304 |
| 102.100.100/19492 | -33.6062 | 150.7304 |
| 102.100.100/19493 | -33.6094 | 150.7314 |
| 102.100.100/19494 | -33.6094 | 150.7314 |
| 102.100.100/19495 | -33.6096 | 150.7324 |
| 102.100.100/19496 | -33.6096 | 150.7324 |
| 102.100.100/19497 | -33.609 | 150.7254 |
| 102.100.100/19498 | -33.609 | 150.7254 |
| 102.100.100/19499 | -33.6113 | 150.7284 |
| 102.100.100/19500 | -33.6113 | 150.7284 |
| 102.100.100/19501 | -33.6116 | 150.7258 |
| 102.100.100/19502 | -33.6116 | 150.7258 |
| 102.100.100/19503 | -33.6089 | 150.7226 |
| 102.100.100/19504 | -33.6089 | 150.7226 |
| 102.100.100/19505 | -33.6145 | 150.7288 |
| 102.100.100/19506 | -33.6145 | 150.7288 |
| 102.100.100/19507 | -33.6101 | 150.7198 |
| 102.100.100/19508 | -33.6101 | 150.7198 |
| 102.100.100/19509 | -33.6107 | 150.7204 |
| 102.100.100/19510 | -33.6107 | 150.7204 |
| 102.100.100/19511 | -33.6126 | 150.721 |
| 102.100.100/19512 | -33.6126 | 150.721 |
| 102.100.100/19513 | -33.611 | 150.72 |
| 102.100.100/19514 | -33.611 | 150.72 |
| 102.100.100/19515 | -33.6157 | 150.7217 |
| 102.100.100/19516 | -33.6157 | 150.7217 |
| 102.100.100/19517 | -33.6174 | 150.7328 |
| 102.100.100/19518 | -33.6174 | 150.7328 |
| 102.100.100/7031 | -31.2428 | 136.7404 |
| 102.100.100/7032 | -31.2428 | 136.7404 |
| 102.100.100/7033 | -31.2274 | 136.4046 |
| 102.100.100/7034 | -31.2274 | 136.4046 |
| 102.100.100/7035 | -29.002 | 134.6857 |
| 102.100.100/7036 | -29.002 | 134.6857 |
| 102.100.100/7037 | -27.2399 | 133.5572 |
| 102.100.100/7038 | -27.2399 | 133.5572 |
| 102.100.100/7039 | -25.7096 | 133.2486 |
| 102.100.100/7040 | -25.7096 | 133.2486 |
| 102.100.100/7041 | -22.9102 | 132.6421 |
| 102.100.100/7042 | -22.9102 | 132.6421 |
| 102.100.100/7043 | -23.2524 | 132.6404 |
| 102.100.100/7044 | -23.2524 | 132.6404 |
| 102.100.100/7045 | -22.914 | 132.6242 |
| 102.100.100/7047 | -22.435 | 132.0354 |
| 102.100.100/7048 | -22.435 | 132.0354 |
| 102.100.100/7049 | -21.7076 | 131.1041 |
| 102.100.100/7050 | -21.7076 | 131.1041 |
| 102.100.100/7051 | -20.2924 | 130.0358 |
| 102.100.100/7052 | -20.2924 | 130.0358 |
| 102.100.100/7053 | -20.2715 | 129.9691 |
| 102.100.100/7054 | -20.2715 | 129.9691 |
| 102.100.100/7055 | -20.2881 | 129.9873 |
| 102.100.100/7056 | -20.2881 | 129.9873 |
| 102.100.100/7057 | -20.1877 | 129.1592 |
| 102.100.100/7058 | -20.1877 | 129.1592 |
| 102.100.100/7059 | -17.8736 | 127.873 |
| 102.100.100/7060 | -17.8736 | 127.873 |
| 102.100.100/7061 | -17.6741 | 125.2178 |
| 102.100.100/7062 | -17.6741 | 125.2178 |
| 102.100.100/7063 | -17.507 | 123.7349 |
| 102.100.100/7064 | -17.507 | 123.7349 |
| 102.100.100/7065 | -19.7687 | 120.6808 |
| 102.100.100/7066 | -19.7687 | 120.6808 |
| 102.100.100/7067 | -21.1165 | 116.8286 |
| 102.100.100/7068 | -21.1165 | 116.8286 |
| 102.100.100/7069 | -22.5707 | 118.0807 |
| 102.100.100/7070 | -22.5707 | 118.0807 |
| 102.100.100/7075 | -28.0505 | 120.5462 |
| 102.100.100/7076 | -28.0505 | 120.5462 |
| 102.100.100/7077 | -28.0531 | 120.5453 |
| 102.100.100/7078 | -28.0531 | 120.5453 |
| 102.100.100/7079 | -28.1722 | 121.2343 |
| 102.100.100/7080 | -28.1722 | 121.2343 |
| 102.100.100/7081 | -30.4051 | 121.0623 |
| 102.100.100/7082 | -30.4051 | 121.0623 |
| 102.100.100/7083 | -32.82 | 116.4136 |
| 102.100.100/7084 | -32.82 | 116.4136 |
| 102.100.100/7085 | -32.5889 | 116.4591 |
| 102.100.100/7086 | -32.5889 | 116.4591 |
| 102.100.100/7087 | -32.1587 | 121.7603 |
| 102.100.100/7088 | -32.1587 | 121.7603 |
| 102.100.100/7089 | -32.1586 | 121.7609 |
| 102.100.100/7090 | -32.1586 | 121.7609 |
| 102.100.100/7091 | -31.8633 | 126.9831 |
| 102.100.100/7093 | -31.4092 | 130.9937 |
| 102.100.100/7094 | -31.4092 | 130.9937 |
| 102.100.100/7095 | -32.9101 | 135.34 |
| 102.100.100/7096 | -32.9101 | 135.34 |
| 102.100.100/7823 | -35.1643 | 150.6594 |
| 102.100.100/7824 | -35.1643 | 150.6594 |
| 102.100.100/7825 | -35.1419 | 150.7596 |
| 102.100.100/7826 | -35.1419 | 150.7596 |
| 102.100.100/7827 | -35.1281 | 150.7569 |
| 102.100.100/7828 | -35.1281 | 150.7569 |
| 102.100.100/7829 | -35.1537 | 150.7537 |
| 102.100.100/7830 | -35.1537 | 150.7537 |
| 102.100.100/7831 | -35.153 | 150.7353 |
| 102.100.100/7832 | -35.153 | 150.7353 |
| 102.100.100/7833 | -35.138 | 150.7076 |
| 102.100.100/7834 | -35.138 | 150.7076 |
| 102.100.100/7835 | -35.1984 | 150.6945 |
| 102.100.100/7836 | -35.1984 | 150.6945 |
| 102.100.100/7837 | -35.1626 | 150.7251 |
| 102.100.100/7838 | -35.1626 | 150.7251 |
| 102.100.100/7839 | -35.128 | 150.6584 |
| 102.100.100/7840 | -35.128 | 150.6584 |
| 102.100.100/7841 | -35.1393 | 150.6831 |
| 102.100.100/7842 | -35.1393 | 150.6831 |
| 102.100.100/7843 | -35.1512 | 150.6487 |
| 102.100.100/7844 | -35.1512 | 150.6487 |
| 102.100.100/7845 | -35.1591 | 150.637 |
| 102.100.100/7846 | -35.1591 | 150.637 |
| 102.100.100/7847 | -35.1573 | 150.6136 |
| 102.100.100/7848 | -35.1573 | 150.6136 |
| 102.100.100/7849 | -35.1413 | 150.738 |
| 102.100.100/7850 | -35.1413 | 150.738 |
| 102.100.100/7851 | -35.1497 | 150.6863 |
| 102.100.100/7852 | -35.1497 | 150.6863 |
| 102.100.100/7853 | -35.163 | 150.6731 |
| 102.100.100/7854 | -35.163 | 150.6731 |
| 102.100.100/7855 | -35.1367 | 150.6667 |
| 102.100.100/7856 | -35.1367 | 150.6667 |
| 102.100.100/7857 | -35.1618 | 150.6327 |
| 102.100.100/7858 | -35.1618 | 150.6327 |
| 102.100.100/7859 | -35.1737 | 150.5941 |
| 102.100.100/7860 | -35.1737 | 150.5941 |
| 102.100.100/7861 | -35.1736 | 150.5943 |
| 102.100.100/7862 | -35.1736 | 150.5943 |
| 102.100.100/7863 | -35.1771 | 150.5937 |
| 102.100.100/7864 | -35.1771 | 150.5937 |
| 102.100.100/7882 | -34.1178 | 118.7606 |
| 102.100.100/7883 | -34.1178 | 118.7606 |
| 102.100.100/7884 | -34.1172 | 118.7605 |
| 102.100.100/7885 | -34.1172 | 118.7605 |
| 102.100.100/7886 | -34.1172 | 118.7615 |
| 102.100.100/7887 | -34.1172 | 118.7615 |
| 102.100.100/7888 | -35.431 | 148.8023 |
| 102.100.100/7889 | -35.431 | 148.8023 |
| 102.100.100/7890 | -32.2414 | 118.72 |
| 102.100.100/7891 | -32.2414 | 118.72 |
| 102.100.100/7892 | -34.2401 | 118.7205 |
| 102.100.100/7893 | -34.2401 | 118.7205 |
| 102.100.100/7894 | -34.2416 | 118.721 |
| 102.100.100/7895 | -34.2416 | 118.721 |
| 102.100.100/7896 | -34.0863 | 118.8528 |
| 102.100.100/7897 | -34.0863 | 118.8528 |
| 102.100.100/7898 | -34.0849 | 118.8512 |
| 102.100.100/7899 | -34.0849 | 118.8512 |
| 102.100.100/7900 | -34.0845 | 118.8502 |
| 102.100.100/7901 | -34.0845 | 118.8502 |
| 102.100.100/7902 | -34.0761 | 118.8709 |
| 102.100.100/7903 | -34.0761 | 118.8709 |
| 102.100.100/7904 | -34.0772 | 118.8687 |
| 102.100.100/7905 | -34.0772 | 118.8687 |
| 102.100.100/7906 | -34.0746 | 118.8695 |
| 102.100.100/7907 | -34.0746 | 118.8695 |
| 102.100.100/7908 | -34.0944 | 118.8603 |
| 102.100.100/7909 | -34.0944 | 118.8603 |
| 102.100.100/7910 | -34.0939 | 118.8601 |
| 102.100.100/7911 | -34.0939 | 118.8601 |
| 102.100.100/7912 | -34.0945 | 118.8615 |
| 102.100.100/7913 | -34.0945 | 118.8615 |
| 102.100.100/7914 | -34.3032 | 118.7278 |
| 102.100.100/7915 | -34.3032 | 118.7278 |
| 102.100.100/7916 | -34.3037 | 118.7303 |
| 102.100.100/7917 | -34.3037 | 118.7303 |
| 102.100.100/7918 | -34.3033 | 118.7313 |
| 102.100.100/7919 | -34.3033 | 118.7313 |
| 102.100.100/7920 | -30.0912 | 115.1531 |
| 102.100.100/7921 | -30.0912 | 115.1531 |
| 102.100.100/7922 | -30.103 | 115.1639 |
| 102.100.100/7923 | -30.103 | 115.1639 |
| 102.100.100/8076 | -30.1816 | 115.2116 |
| 102.100.100/8077 | -30.1816 | 115.2116 |
| 102.100.100/8078 | -30.1711 | 115.2134 |
| 102.100.100/8079 | -30.1711 | 115.2134 |
| 102.100.100/8080 | -30.1691 | 115.2526 |
| 102.100.100/8081 | -30.1691 | 115.2526 |
| 102.100.100/8082 | -30.1586 | 115.2499 |
| 102.100.100/8083 | -30.1586 | 115.2499 |
| 102.100.100/8084 | -30.1119 | 115.2585 |
| 102.100.100/8085 | -30.1119 | 115.2585 |
| 102.100.100/8086 | -30.1363 | 115.1575 |
| 102.100.100/8087 | -30.1363 | 115.1575 |
| 102.100.100/8088 | -30.0411 | 115.0204 |
| 102.100.100/8089 | -30.0411 | 115.0204 |
| 102.100.100/8090 | -30.0489 | 115.0423 |
| 102.100.100/8091 | -30.0489 | 115.0423 |
| 102.100.100/8092 | -30.0716 | 115.0761 |
| 102.100.100/8093 | -30.0716 | 115.0761 |
| 102.100.100/8094 | -30.1348 | 115.0195 |
| 102.100.100/8095 | -30.1348 | 115.0195 |
| 102.100.100/8096 | -30.1831 | 115.1268 |
| 102.100.100/8097 | -30.1831 | 115.1268 |
| 102.100.100/8098 | -30.1411 | 115.0896 |
| 102.100.100/8099 | -30.1411 | 115.0896 |
| 102.100.100/8100 | -30.1067 | 115.1161 |
| 102.100.100/8101 | -30.1067 | 115.1161 |
| 102.100.100/8102 | -30.1611 | 115.1746 |
| 102.100.100/8103 | -30.1611 | 115.1746 |
| 102.100.100/8104 | -30.1095 | 115.15 |
| 102.100.100/8105 | -30.1095 | 115.15 |
| 102.100.100/8106 | -30.1066 | 115.1502 |
| 102.100.100/8107 | -30.1066 | 115.1502 |
| 102.100.100/8108 | -33.8568 | 119.1616 |
| 102.100.100/8109 | -33.8568 | 119.1616 |
| 102.100.100/8110 | -34.0003 | 119.2123 |
| 102.100.100/8111 | -34.0003 | 119.2123 |
| 102.100.100/8112 | -34.0237 | 119.2884 |
| 102.100.100/8113 | -34.0237 | 119.2884 |
| 102.100.100/8114 | -34.0877 | 119.4063 |
| 102.100.100/8115 | -34.0877 | 119.4063 |
| 102.100.100/8116 | -34.1677 | 119.5757 |
| 102.100.100/8117 | -34.1677 | 119.5757 |
| 102.100.100/8118 | -34.3398 | 119.3947 |
| 102.100.100/8119 | -34.3398 | 119.3947 |
| 102.100.100/8120 | -34.2165 | 119.304 |
| 102.100.100/8121 | -34.2165 | 119.304 |
| 102.100.100/8122 | -34.1922 | 119.4254 |
| 102.100.100/8123 | -34.1922 | 119.4254 |
| 102.100.100/8124 | -34.3811 | 119.3413 |
| 102.100.100/8125 | -34.3811 | 119.3413 |
| 102.100.100/8126 | -33.8095 | 119.4345 |
| 102.100.100/8127 | -33.8095 | 119.4345 |
| 102.100.100/8128 | -33.7284 | 119.7221 |
| 102.100.100/8129 | -33.7284 | 119.7221 |
| 102.100.100/8130 | -33.6884 | 119.7617 |
| 102.100.100/8131 | -33.6884 | 119.7617 |
| 102.100.100/8132 | -33.9313 | 119.9997 |
| 102.100.100/8133 | -33.9313 | 119.9997 |
| 102.100.100/8134 | -33.8771 | 119.9184 |
| 102.100.100/8135 | -33.8771 | 119.9184 |
| 102.100.100/8136 | -33.8471 | 119.9151 |
| 102.100.100/8137 | -33.8471 | 119.9151 |
| 102.100.100/8138 | -30.2007 | 149.5975 |
| 102.100.100/8139 | -30.2007 | 149.5975 |
| 102.100.100/8140 | -30.2006 | 149.5976 |
| 102.100.100/8141 | -30.2006 | 149.5976 |
| 102.100.100/8142 | -35.8748 | 149.0117 |
| 102.100.100/8143 | -35.8748 | 149.0117 |
| 102.100.100/8144 | -35.881 | 149.0076 |
| 102.100.100/8145 | -35.881 | 149.0076 |
| 102.100.100/8146 | -35.8811 | 149.0083 |
| 102.100.100/8147 | -35.8811 | 149.0083 |
| 102.100.100/8148 | -35.8767 | 149.0097 |
| 102.100.100/8149 | -35.8767 | 149.0097 |
| 102.100.100/8150 | -35.8769 | 149.0103 |
| 102.100.100/8151 | -35.8769 | 149.0103 |
| 102.100.100/8152 | -35.8749 | 149.0109 |
| 102.100.100/8153 | -35.8749 | 149.0109 |
| 102.100.100/8154 | -25.351 | 131.0423 |
| 102.100.100/8155 | -25.351 | 131.0423 |
| 102.100.100/8156 | -25.3564 | 131.0368 |
| 102.100.100/8157 | -25.3564 | 131.0368 |
| 102.100.100/8158 | -25.3504 | 131.0516 |
| 102.100.100/8159 | -25.3504 | 131.0516 |
| 102.100.100/8160 | -25.351 | 131.041 |
| 102.100.100/8161 | -25.351 | 131.041 |
| 102.100.100/8162 | -25.3523 | 131.0343 |
| 102.100.100/8163 | -25.3523 | 131.0343 |
| 102.100.100/8164 | -25.3457 | 131.0213 |
| 102.100.100/8165 | -25.3457 | 131.0213 |
| 102.100.100/8166 | -25.3457 | 131.0213 |
| 102.100.100/8167 | -25.3457 | 131.0213 |
| 102.100.100/8168 | -25.3374 | 131.0404 |
| 102.100.100/8169 | -25.3374 | 131.0404 |
| 102.100.100/8170 | -25.3374 | 131.0403 |
| 102.100.100/8171 | -25.3374 | 131.0403 |
| 102.100.100/8172 | -25.3629 | 131.0138 |
| 102.100.100/8173 | -25.3629 | 131.0138 |
| 102.100.100/8174 | -25.3629 | 131.0138 |
| 102.100.100/8175 | -25.3629 | 131.0138 |
| 102.100.100/8180 | -35.1164 | 141.9764 |
| 102.100.100/8181 | -35.1164 | 141.9764 |
| 102.100.100/8182 | -35.1164 | 141.9766 |
| 102.100.100/8183 | -35.1164 | 141.9766 |
| 102.100.100/8184 | -35.1164 | 141.9766 |
| 102.100.100/8185 | -35.1164 | 141.9766 |
| 102.100.100/8190 | -19.7333 | 147.1787 |
| 102.100.100/8191 | -19.7333 | 147.1787 |
| 102.100.100/8192 | -21.1596 | 149.1128 |
| 102.100.100/8193 | -21.1596 | 149.1128 |
| 102.100.100/8194 | -19.7333 | 147.1787 |
| 102.100.100/8196 | -19.7333 | 147.1787 |
| 102.100.100/8198 | -21.1596 | 149.1128 |
| 102.100.100/8199 | -21.1596 | 149.1128 |
| 102.100.100/8200 | -34.2327 | 138.3102 |
| 102.100.100/8201 | -34.2394 | 138.3135 |
| 102.100.100/8202 | -34.2406 | 138.3146 |
| 102.100.100/8203 | -32.7224 | 134.2871 |
| 102.100.100/8204 | -30.2012 | 149.5957 |
| 102.100.100/8205 | -30.2012 | 149.5957 |
| 102.100.100/8206 | -30.2003 | 149.5939 |
| 102.100.100/8207 | -30.2012 | 149.5957 |
| 102.100.100/8208 | -30.2012 | 149.5957 |
| 102.100.100/8209 | -30.2043 | 149.5944 |
| 102.100.100/8210 | -35.0822 | 140.0879 |
| 102.100.100/8211 | -35.0822 | 140.0879 |
| 102.100.100/8212 | -35.0822 | 140.0879 |
| 102.100.100/8213 | -35.0822 | 140.0879 |
| 102.100.100/8214 | -35.0857 | 140.086 |
| 102.100.100/8215 | -35.0857 | 140.086 |
| 102.100.100/8216 | -32.8314 | 135.1638 |
| 102.100.100/8217 | -32.8346 | 135.1557 |
| 102.100.100/8218 | -32.8346 | 135.1557 |
| 102.100.100/8220 | -21.1596 | 149.1128 |
| 102.100.100/8221 | -21.1596 | 149.1128 |
| 102.100.100/8226 | -28.2138 | 152.1001 |
| 102.100.100/8227 | -28.2138 | 152.1001 |
| 102.100.100/8228 | -28.2138 | 152.1001 |
| 102.100.100/8229 | -28.2138 | 152.1001 |
| 102.100.100/8230 | -28.2075 | 152.105 |
| 102.100.100/8231 | -28.2075 | 152.105 |
| 102.100.100/8232 | -26.8744 | 151.629 |
| 102.100.100/8233 | -26.8744 | 151.629 |
| 102.100.100/8234 | -26.8747 | 151.0748 |
| 102.100.100/8235 | -27.4737 | 151.3966 |
| 102.100.100/8236 | -27.4737 | 151.3966 |
| 102.100.100/8237 | -27.4737 | 151.3966 |
| 102.100.100/8262 | -35.1164 | 141.9763 |
| 102.100.100/8263 | -35.1164 | 141.9763 |
| 102.100.100/8264 | -35.1164 | 141.9765 |
| 102.100.100/8265 | -35.1164 | 141.9765 |
| 102.100.100/8266 | -35.1164 | 141.9766 |
| 102.100.100/8267 | -35.1164 | 141.9766 |
| 102.100.100/8268 | -35.1228 | 141.9968 |
| 102.100.100/8269 | -35.1228 | 141.9968 |
| 102.100.100/8270 | -36.6715 | 142.2893 |
| 102.100.100/8271 | -36.6715 | 142.2893 |
| 102.100.100/8272 | -36.6717 | 142.2889 |
| 102.100.100/8273 | -36.6717 | 142.2889 |
| 102.100.100/8274 | -36.6711 | 142.2893 |
| 102.100.100/8275 | -36.6711 | 142.2893 |
| 102.100.100/8276 | -36.6684 | 142.3018 |
| 102.100.100/8277 | -36.6684 | 142.3018 |
| 102.100.100/8278 | -37.8412 | 142.0881 |
| 102.100.100/8279 | -37.8412 | 142.0881 |
| 102.100.100/8280 | -37.8416 | 142.089 |
| 102.100.100/8281 | -37.8416 | 142.089 |
| 102.100.100/8282 | -37.8392 | 142.0872 |
| 102.100.100/8283 | -37.8392 | 142.0872 |
| 102.100.100/8284 | -37.8409 | 142.088 |
| 102.100.100/8285 | -37.8409 | 142.088 |
| 102.100.100/8286 | -37.8413 | 142.0882 |
| 102.100.100/8287 | -37.8413 | 142.0882 |
| 102.100.100/8288 | -37.8407 | 142.0833 |
| 102.100.100/8291 | -36.1103 | 146.5083 |
| 102.100.100/8292 | -36.1096 | 146.5083 |
| 102.100.100/8293 | -36.1096 | 146.5083 |
| 102.100.100/8294 | -36.1111 | 146.5083 |
| 102.100.100/8295 | -36.1111 | 146.5083 |
| 102.100.100/8296 | -36.1138 | 146.4875 |
| 102.100.100/8297 | -36.1138 | 146.4875 |
| 102.100.100/8453 | -25.3982 | 130.9972 |
| 102.100.100/8455 | -25.3119 | 131.085 |
| 102.100.100/8456 | -25.3119 | 131.085 |
| 102.100.100/8457 | -25.3118 | 131.084 |
| 102.100.100/8458 | -25.3118 | 131.084 |
| 102.100.100/8459 | -25.2875 | 130.9286 |
| 102.100.100/8460 | -25.2875 | 130.9286 |
| 102.100.100/8461 | -25.2713 | 130.756 |
| 102.100.100/8462 | -25.2713 | 130.756 |
| 102.100.100/8463 | -25.3565 | 130.8933 |
| 102.100.100/8464 | -25.3565 | 130.8933 |
| 102.100.100/8465 | -25.3311 | 131.0738 |
| 102.100.100/8466 | -25.3311 | 131.0738 |
| 102.100.100/8467 | -25.2686 | 130.936 |
| 102.100.100/8468 | -25.2686 | 130.936 |
| 102.100.100/8469 | -25.2882 | 130.9074 |
| 102.100.100/8470 | -25.2882 | 130.9074 |
| 102.100.100/8471 | -25.287 | 130.9296 |
| 102.100.100/8472 | -25.287 | 130.9296 |
| 102.100.100/8487 | -12.6489 | 132.3738 |
| 102.100.100/8488 | -12.6489 | 132.3738 |
| 102.100.100/8489 | -12.6207 | 132.6334 |
| 102.100.100/8490 | -12.6207 | 132.6334 |
| 102.100.100/8491 | -12.6208 | 132.6337 |
| 102.100.100/8492 | -12.6208 | 132.6337 |
| 102.100.100/8493 | -12.9293 | 132.5948 |
| 102.100.100/8494 | -12.9293 | 132.5948 |
| 102.100.100/8495 | -12.9028 | 132.6307 |
| 102.100.100/8496 | -12.9028 | 132.6307 |
| 102.100.100/8497 | -13.5539 | 132.2711 |
| 102.100.100/8498 | -13.5539 | 132.2711 |
| 102.100.100/8499 | -12.8575 | 132.6867 |
| 102.100.100/8500 | -12.8575 | 132.6867 |
| 102.100.100/8501 | -35.4227 | 148.7953 |
| 102.100.100/8502 | -35.4227 | 148.7953 |
| 102.100.100/8504 | -35.4037 | 148.7973 |
| 102.100.100/8505 | -35.3965 | 148.8211 |
| 102.100.100/8506 | -35.3965 | 148.8211 |
| 102.100.100/8507 | -13.5165 | 132.4582 |
| 102.100.100/8508 | -13.5165 | 132.4582 |
| 102.100.100/8509 | -13.5216 | 132.4531 |
| 102.100.100/8510 | -13.5216 | 132.4531 |
| 102.100.100/8511 | -13.5183 | 132.4452 |
| 102.100.100/8512 | -13.5183 | 132.4452 |
| 102.100.100/8513 | -13.2964 | 132.3365 |
| 102.100.100/8514 | -13.2964 | 132.3365 |
| 102.100.100/8515 | -12.8233 | 132.7981 |
| 102.100.100/8516 | -12.8233 | 132.7981 |
| 102.100.100/8517 | -12.8649 | 132.79 |
| 102.100.100/8518 | -12.8649 | 132.79 |
| 102.100.100/8519 | -12.8654 | 132.8191 |
| 102.100.100/8520 | -12.8654 | 132.8191 |
| 102.100.100/8521 | -12.6763 | 132.8138 |
| 102.100.100/8522 | -12.6763 | 132.8138 |
| 102.100.100/8523 | -12.7061 | 132.8432 |
| 102.100.100/8524 | -12.7061 | 132.8432 |
| 102.100.100/8525 | -12.4305 | 132.9485 |
| 102.100.100/8526 | -12.4305 | 132.9485 |
| 102.100.100/8527 | -12.4338 | 132.942 |
| 102.100.100/8528 | -12.4338 | 132.942 |
| 102.100.100/8529 | -12.6733 | 132.4819 |
| 102.100.100/8530 | -12.6733 | 132.4819 |
| 102.100.100/8531 | -12.6786 | 132.4792 |
| 102.100.100/8532 | -12.6786 | 132.4792 |
| 102.100.100/9430 | -35.6016 | 148.9509 |
| 102.100.100/9431 | -35.6016 | 148.9509 |
| 102.100.100/9432 | -35.5199 | 148.7724 |
| 102.100.100/9433 | -35.5199 | 148.7724 |
| 102.100.100/9434 | -35.6055 | 148.9321 |
| 102.100.100/9435 | -35.6055 | 148.9321 |
| 102.100.100/9436 | -35.6073 | 148.9297 |
| 102.100.100/9437 | -35.6073 | 148.9297 |
| 102.100.100/9438 | -35.4454 | 148.7851 |
| 102.100.100/9439 | -35.4454 | 148.7851 |
| 102.100.100/9440 | -35.4502 | 148.7826 |
| 102.100.100/9441 | -35.4502 | 148.7826 |
| 102.100.100/9442 | -35.4463 | 148.7846 |
| 102.100.100/9443 | -35.4463 | 148.7846 |
| 102.100.100/9444 | -35.3365 | 148.8305 |
| 102.100.100/9445 | -35.3365 | 148.8305 |
| 102.100.100/9446 | -35.3556 | 148.8001 |
| 102.100.100/9447 | -35.3556 | 148.8001 |
| 102.100.100/9448 | -35.3952 | 148.8234 |
| 102.100.100/9449 | -35.3952 | 148.8234 |
| 102.100.100/9450 | -35.3941 | 148.8271 |
| 102.100.100/9451 | -35.3941 | 148.8271 |
| 102.100.100/9452 | -35.3929 | 148.8149 |
| 102.100.100/9453 | -35.3929 | 148.8149 |
| 102.100.100/9454 | -35.5655 | 148.7831 |
| 102.100.100/9455 | -35.5655 | 148.7831 |
| 102.100.100/9456 | -35.5252 | 148.7763 |
| 102.100.100/9457 | -35.5252 | 148.7763 |
| 102.100.100/9458 | -35.3912 | 148.8111 |
| 102.100.100/9459 | -35.3912 | 148.8111 |
| 102.100.100/9460 | -35.3719 | 148.8447 |
| 102.100.100/9461 | -35.3719 | 148.8447 |
| 102.100.100/9462 | -35.4346 | 148.7989 |
| 102.100.100/9463 | -35.4346 | 148.7989 |
| 102.100.100/9464 | -35.586 | 148.9125 |
| 102.100.100/9465 | -35.586 | 148.9125 |
| 102.100.100/9466 | -35.5907 | 148.9085 |
| 102.100.100/9467 | -35.5907 | 148.9085 |
| 102.100.100/9468 | -35.4238 | 148.7832 |
| 102.100.100/9469 | -35.4238 | 148.7832 |
| 102.100.100/9484 | -35.6083 | 138.2613 |
| 102.100.100/9485 | -35.6083 | 138.2613 |
| 102.100.100/9486 | -35.2721 | 138.6902 |
| 102.100.100/9487 | -35.2721 | 138.6902 |
| 102.100.100/9488 | -32.8285 | 138.0327 |
| 102.100.100/9489 | -32.8285 | 138.0327 |
| 102.100.100/9490 | -32.3034 | 137.9574 |
| 102.100.100/9491 | -32.3034 | 137.9574 |
| 102.100.100/9492 | -32.7484 | 138.1363 |
| 102.100.100/9493 | -32.7484 | 138.1363 |
| 102.100.100/9494 | -33.9142 | 138.6038 |
| 102.100.100/9495 | -33.9142 | 138.6038 |
| 102.100.100/9496 | -34.609 | 138.8613 |
| 102.100.100/9497 | -34.609 | 138.8613 |
| 102.100.100/9498 | -34.5769 | 139.0061 |
| 102.100.100/9499 | -34.5769 | 139.0061 |
| 102.100.100/9500 | -34.6831 | 138.9086 |
| 102.100.100/9501 | -34.6831 | 138.9086 |
| 102.100.100/9502 | -34.8808 | 138.7083 |
| 102.100.100/9503 | -34.8808 | 138.7083 |
| 102.100.100/9504 | -34.9334 | 138.727 |
| 102.100.100/9505 | -34.9334 | 138.727 |
| 102.100.100/9506 | -32.321 | 137.9544 |
| 102.100.100/9507 | -32.321 | 137.9544 |
| 102.100.100/9508 | -30.7757 | 138.7976 |
| 102.100.100/9509 | -30.7757 | 138.7976 |
| 102.100.100/9510 | -31.3277 | 138.5673 |
| 102.100.100/9511 | -31.3277 | 138.5673 |
| 102.100.100/9512 | -31.5439 | 138.5946 |
| 102.100.100/9513 | -31.5439 | 138.5946 |
| 102.100.100/9514 | -33.9743 | 140.7258 |
| 102.100.100/9515 | -33.9743 | 140.7258 |
| 102.100.100/9516 | -34.0189 | 140.7095 |
| 102.100.100/9517 | -33.9569 | 140.7186 |
| 102.100.100/9518 | -33.9556 | 140.719 |
| 102.100.100/9519 | -33.9743 | 140.7258 |
| 102.100.100/9520 | -33.9727 | 140.7273 |
| 102.100.100/9521 | -34.0372 | 140.7064 |
| 102.100.100/9522 | -33.9727 | 140.7273 |
| 102.100.100/9523 | -33.9727 | 140.7273 |
| 102.100.100/9524 | -34.0189 | 140.7095 |
| 102.100.100/9525 | -34.0189 | 140.7095 |
| 102.100.100/9527 | -34.0446 | 140.7615 |
| 102.100.100/9528 | -34.0432 | 140.7596 |
| 102.100.100/9529 | -34.0432 | 140.7596 |
| 102.100.100/9530 | -33.9569 | 140.7186 |
| 102.100.100/9531 | -33.9569 | 140.7186 |
| 102.100.100/9567 | -41.5324 | 145.8955 |
| 102.100.100/9568 | -41.5324 | 145.8955 |
| 102.100.100/9570 | -41.5334 | 145.8955 |
| 102.100.100/9571 | -41.5554 | 145.8814 |
| 102.100.100/9572 | -41.5554 | 145.8814 |
| 102.100.100/9573 | -42.0416 | 146.299 |
| 102.100.100/9574 | -42.0416 | 146.299 |
| 102.100.100/9576 | -42.0436 | 146.3034 |
| 102.100.100/9577 | -34.8585 | 148.5994 |
| 102.100.100/9578 | -34.8585 | 148.5994 |
| 102.100.100/9579 | -34.8581 | 148.5673 |
| 102.100.100/9580 | -34.8581 | 148.5673 |
| 102.100.100/9581 | -34.8585 | 148.5994 |
| 102.100.100/9582 | -34.8585 | 148.5994 |
| 102.100.100/9583 | -34.8581 | 148.5673 |
| 102.100.100/9584 | -34.8581 | 148.5673 |
| 102.100.100/9585 | -41.183 | 148.0291 |
| 102.100.100/9586 | -41.183 | 148.0291 |
| 102.100.100/9587 | -41.1835 | 148.032 |
| 102.100.100/9588 | -41.1835 | 148.032 |
