# Supplementary material for: Linking niche size and phylogenetic signals to predict future soil microbial relative abundances
Source: Front Microbiol. 2023 Aug 14;14:1097909. doi: 10.3389/fmicb.2023.1097909 (PMC10461061; doi:10.3389/fmicb.2023.1097909)
Supplement: Supplementary file 1 [file Data_Sheet_1.zip › Table S2a.docx]

**Table S2: Summary Fit Statistics for SEMs at the Phyla Level.**

|  | Observations | ChiSqM_Value | ChiSqM_DF | ChiSqM_PValue | CFI | RMSEA_Estimate | WRMR | SRMR |
| --- | --- | --- | --- | --- | --- | --- | --- | --- |
| 1 | 1381 | 13.287 | 3 | 0.0041 | 0.986 | 0.05 | 0.107 | 0.002 |
| 2 | 1381 | 13.287 | 3 | 0.0041 | 0.996 | 0.05 | 0.110 | 0.002 |
| 3 | 1381 | 13.287 | 3 | 0.0041 | 0.995 | 0.05 | 0.108 | 0.002 |
| 4 | 1381 | 13.287 | 3 | 0.0041 | 0.994 | 0.05 | 0.109 | 0.002 |
| 5 | 1381 | 13.287 | 3 | 0.0041 | 0.988 | 0.05 | 0.107 | 0.002 |
| 6 | 1381 | 13.287 | 3 | 0.0041 | 0.995 | 0.05 | 0.107 | 0.002 |
| 7 | 1381 | 13.287 | 3 | 0.0041 | 0.995 | 0.05 | 0.108 | 0.002 |
| 8 | 1381 | 13.287 | 3 | 0.0041 | 0.994 | 0.05 | 0.111 | 0.002 |
| 10 | 1381 | 13.287 | 3 | 0.0041 | 0.988 | 0.05 | 0.108 | 0.002 |
| 11 | 1381 | 13.287 | 3 | 0.0041 | 0.989 | 0.05 | 0.106 | 0.002 |
| 12 | 1381 | 13.287 | 3 | 0.0041 | 0.991 | 0.05 | 0.108 | 0.002 |
| 13 | 1380 | 13.028 | 3 | 0.0046 | 0.994 | 0.049 | 0.103 | 0.002 |
| 14 | 1381 | 13.287 | 3 | 0.0041 | 0.994 | 0.05 | 0.111 | 0.002 |
| 15 | 1381 | 13.287 | 3 | 0.0041 | 0.996 | 0.05 | 0.110 | 0.002 |
| 16 | 1381 | 13.287 | 3 | 0.0041 | 0.996 | 0.05 | 0.111 | 0.002 |
| 17 | 1381 | 13.287 | 3 | 0.0041 | 0.993 | 0.05 | 0.111 | 0.002 |
| 18 | 1381 | 13.287 | 3 | 0.0041 | 0.994 | 0.05 | 0.115 | 0.002 |
| 19 | 1380 | 13.437 | 3 | 0.0038 | 0.995 | 0.05 | 0.115 | 0.002 |
| 20 | 1381 | 13.287 | 3 | 0.0041 | 0.989 | 0.05 | 0.107 | 0.002 |
| 21 | 1381 | 13.287 | 3 | 0.0041 | 0.992 | 0.05 | 0.131 | 0.002 |
| 22 | 1380 | 13.034 | 3 | 0.0046 | 0.995 | 0.049 | 0.109 | 0.002 |
| 23 | 1381 | 13.287 | 3 | 0.0041 | 0.990 | 0.05 | 0.107 | 0.002 |
| 24 | 1381 | 13.287 | 3 | 0.0041 | 0.984 | 0.05 | 0.106 | 0.002 |
| 25 | 1381 | 13.287 | 3 | 0.0041 | 0.988 | 0.05 | 0.108 | 0.002 |
| 26 | 1381 | 13.287 | 3 | 0.0041 | 0.993 | 0.05 | 0.109 | 0.002 |
| 27 | 1381 | 13.287 | 3 | 0.0041 | 0.986 | 0.05 | 0.107 | 0.002 |
| 28 | 1381 | 13.287 | 3 | 0.0041 | 0.972 | 0.05 | 0.119 | 0.002 |
| 29 | 1381 | 13.287 | 3 | 0.0041 | 0.992 | 0.05 | 0.107 | 0.002 |
| 30 | 1381 | 13.287 | 3 | 0.0041 | 0.988 | 0.05 | 0.107 | 0.002 |
| 31 | 1381 | 13.287 | 3 | 0.0041 | 0.989 | 0.05 | 0.107 | 0.002 |
| 32 | 1381 | 13.287 | 3 | 0.0041 | 0.992 | 0.05 | 0.106 | 0.002 |
| 33 | 1381 | 13.287 | 3 | 0.0041 | 0.982 | 0.05 | 0.137 | 0.002 |
| 34 | 1381 | 13.287 | 3 | 0.0041 | 0.991 | 0.05 | 0.106 | 0.002 |
| 35 | 1381 | 13.287 | 3 | 0.0041 | 0.995 | 0.05 | 0.109 | 0.002 |
| 36 | 1381 | 13.287 | 3 | 0.0041 | 0.982 | 0.05 | 0.107 | 0.002 |
| 37 | 1381 | 13.287 | 3 | 0.0041 | 0.986 | 0.05 | 0.107 | 0.002 |
| 38 | 1381 | 13.287 | 3 | 0.0041 | 0.995 | 0.05 | 0.114 | 0.002 |
| 39 | 1381 | 13.287 | 3 | 0.0041 | 0.995 | 0.05 | 0.115 | 0.002 |
| 40 | 1381 | 13.287 | 3 | 0.0041 | 0.983 | 0.05 | 0.107 | 0.002 |
| 41 | 1381 | 13.287 | 3 | 0.0041 | 0.988 | 0.05 | 0.106 | 0.002 |
| 43 | 1381 | 13.287 | 3 | 0.0041 | 0.989 | 0.05 | 0.107 | 0.002 |
| 44 | 1381 | 13.287 | 3 | 0.0041 | 0.990 | 0.05 | 0.108 | 0.002 |
| 45 | 1381 | 13.287 | 3 | 0.0041 | 0.996 | 0.05 | 0.113 | 0.002 |
| 46 | 1381 | 13.287 | 3 | 0.0041 | 0.993 | 0.05 | 0.114 | 0.002 |
| 47 | 1381 | 13.287 | 3 | 0.0041 | 0.994 | 0.05 | 0.115 | 0.002 |
| 48 | 1381 | 13.287 | 3 | 0.0041 | 0.992 | 0.05 | 0.107 | 0.002 |
| 50 | 1380 | 13.444 | 3 | 0.0038 | 0.995 | 0.05 | 0.105 | 0.002 |
| 51 | 1381 | 13.287 | 3 | 0.0041 | 0.996 | 0.05 | 0.106 | 0.002 |
| 53 | 1381 | 13.287 | 3 | 0.0041 | 0.994 | 0.05 | 0.112 | 0.002 |
| 54 | 1381 | 13.287 | 3 | 0.0041 | 0.994 | 0.05 | 0.116 | 0.002 |
| 55 | 1381 | 13.287 | 3 | 0.0041 | 0.989 | 0.05 | 0.107 | 0.002 |
| 56 | 1381 | 13.287 | 3 | 0.0041 | 0.991 | 0.05 | 0.114 | 0.002 |
