# Supplementary material for: Linking niche size and phylogenetic signals to predict future soil microbial relative abundances
Source: Front Microbiol. 2023 Aug 14;14:1097909. doi: 10.3389/fmicb.2023.1097909 (PMC10461061; doi:10.3389/fmicb.2023.1097909)
Supplement: Supplementary file 1 [file Data_Sheet_1.zip › Table S3.docx]

**Table S3:** Phylogenetic signal of SEM traits with traits significantly different from zero, as determined by a permutation test (n=99), **bolded.**

|  | SEM Driver (Trait) | Abouheif’s C_mean_ | Probability |
| --- | --- | --- | --- |
| Soil | Conductivity | 0.18 | **0.01** |
|  | pH | 0.11 | **0.01** |
|  | Organic Carbon | -0.04 | 1.00 |
| Vegetation | C3 Macrothermal plants | 0.04 | 0.02 |
|  | C3 Mesothermal plants | 0.02 | 0.08 |
|  | C4 Macrothermal plants | 0.05 | 0.02 |
| Climate | Temperature | 0.06 | **0.01** |
|  | Humidity | 0.03 | 0.03 |
|  | Precipitation | 0.06 | 0.02 |
