# Supplementary figures and images for: Linking niche size and phylogenetic signals to predict future soil microbial relative abundances
Source: Front Microbiol. 2023 Aug 14;14:1097909. doi: 10.3389/fmicb.2023.1097909 (PMC10461061; doi:10.3389/fmicb.2023.1097909)

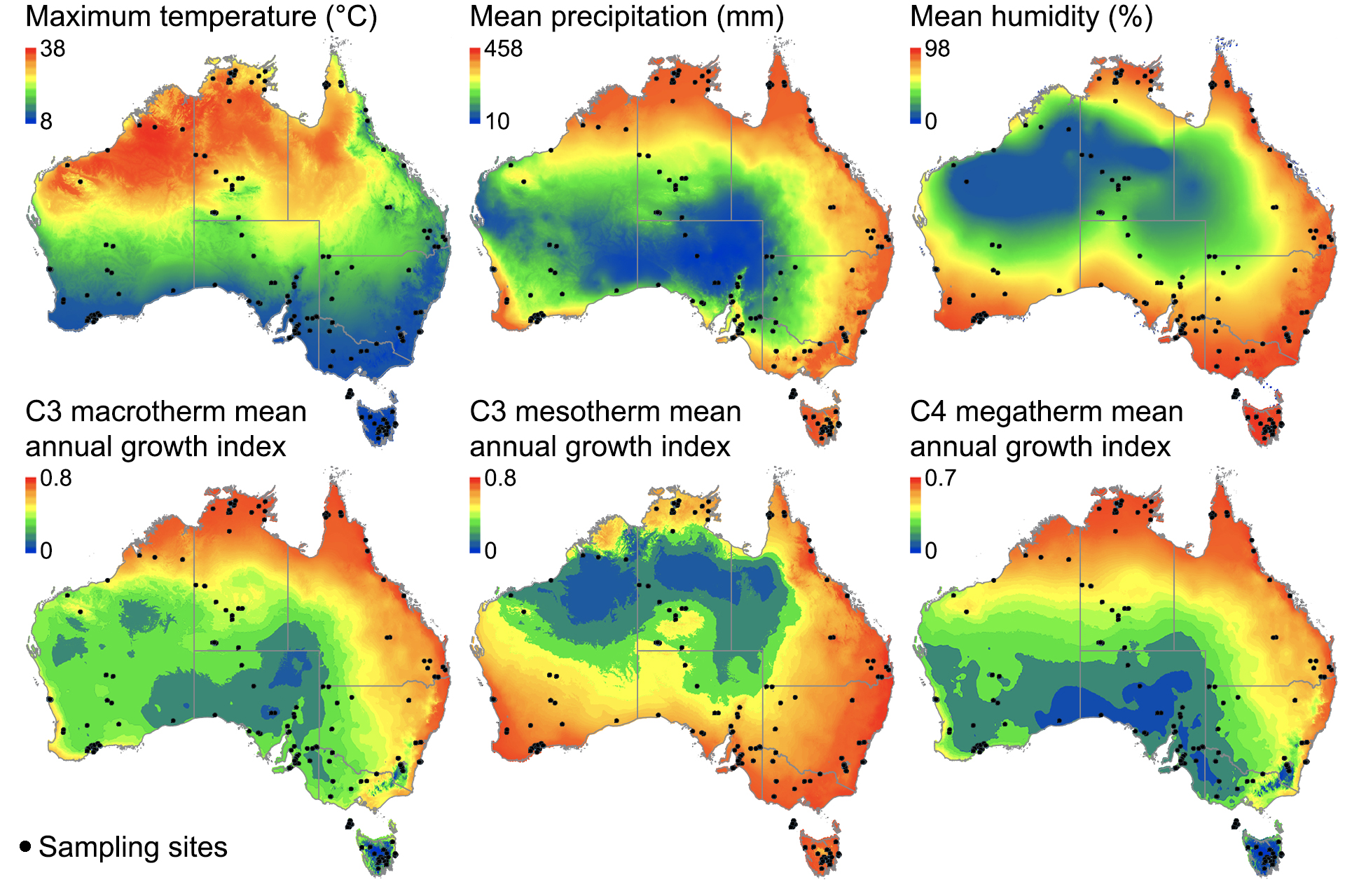

Supplement: Supplementary file 1 [file Data_Sheet_1.zip › ALA_variables_map.jpg]

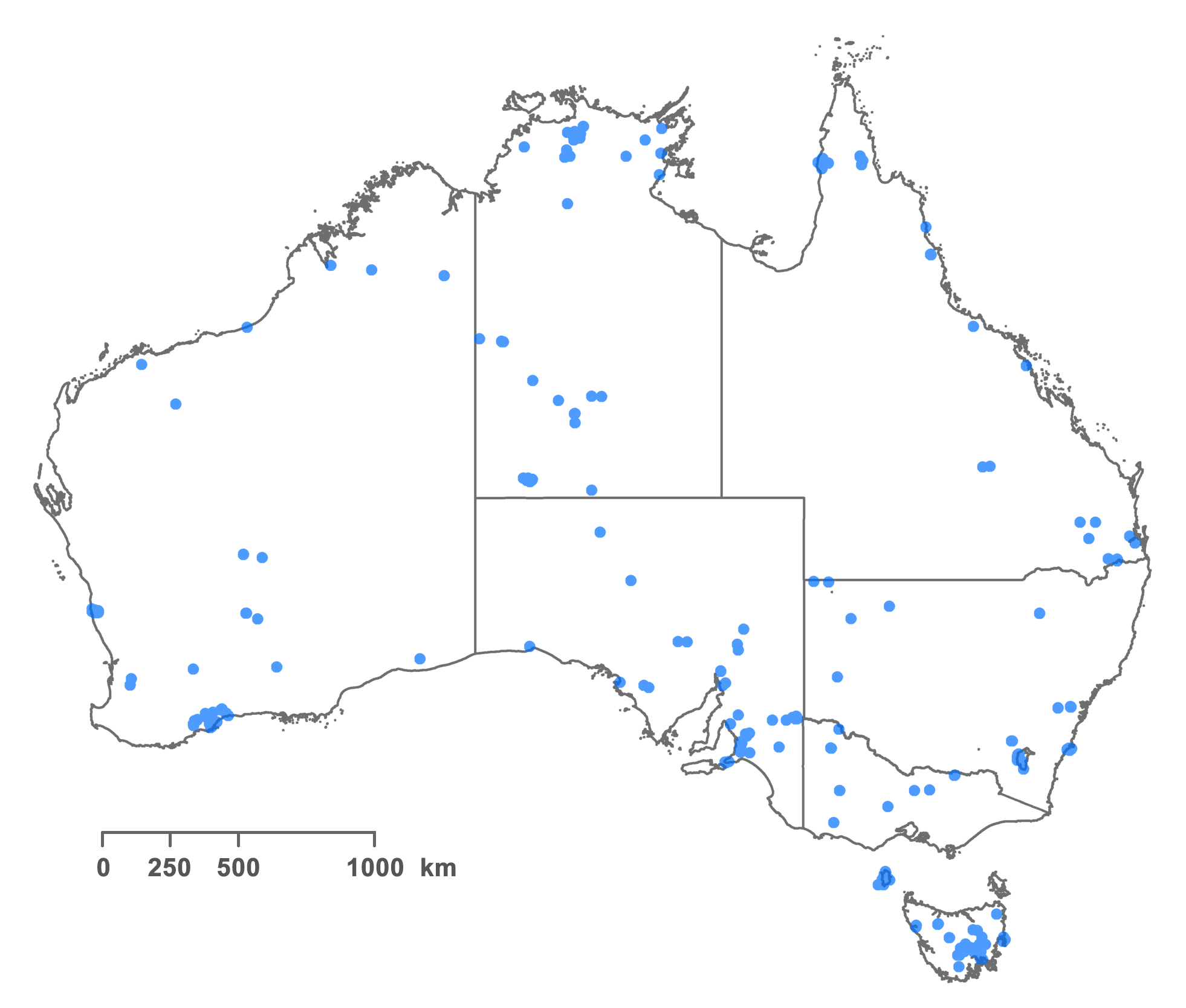

Supplement: Supplementary file 1 [file Data_Sheet_1.zip › Figure S1 Supplementary_Aus_Map.jpg]

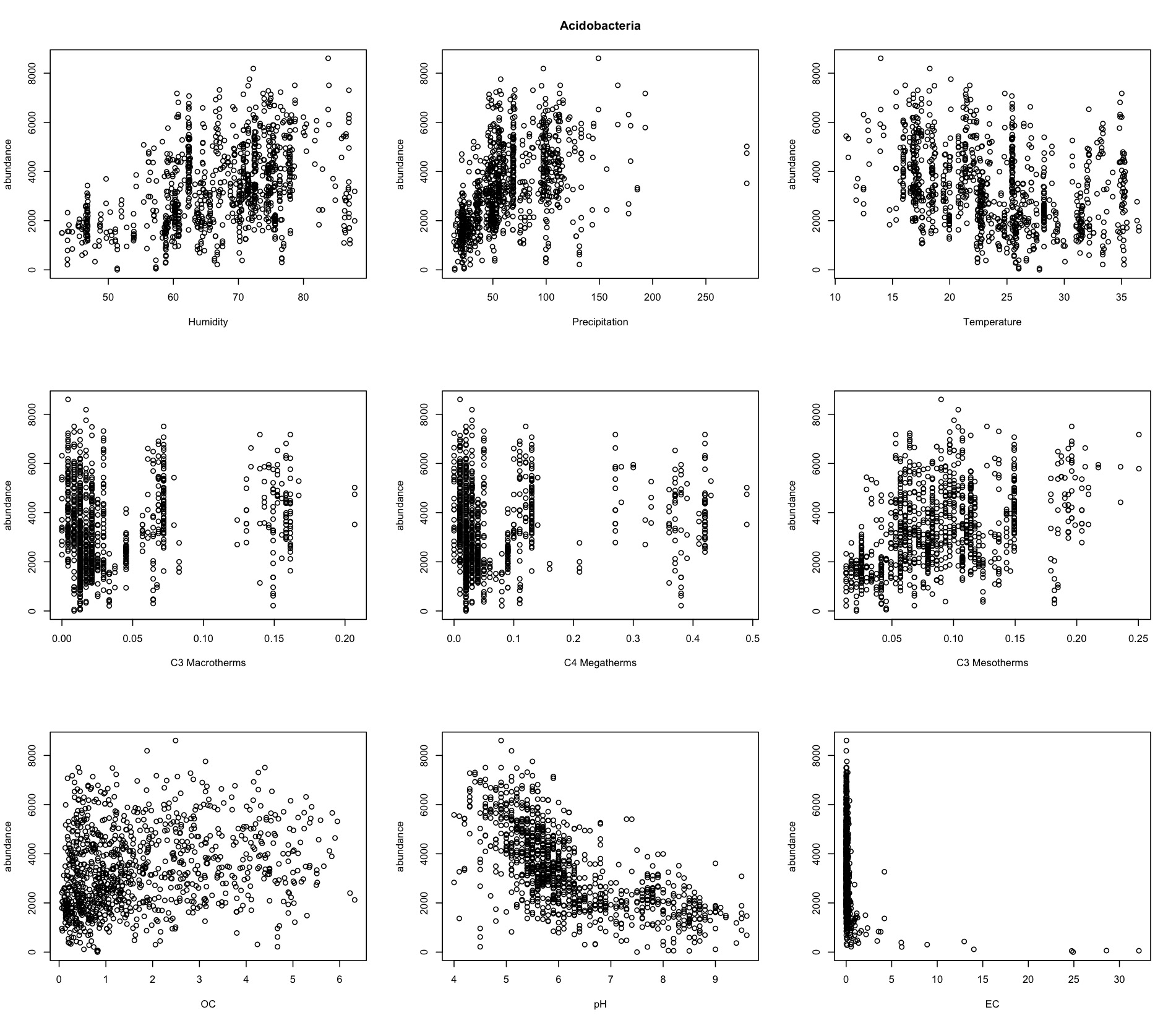

Supplement: Supplementary file 1 [file Data_Sheet_1.zip › Figure S2 Acidobacteria links_R1.png]

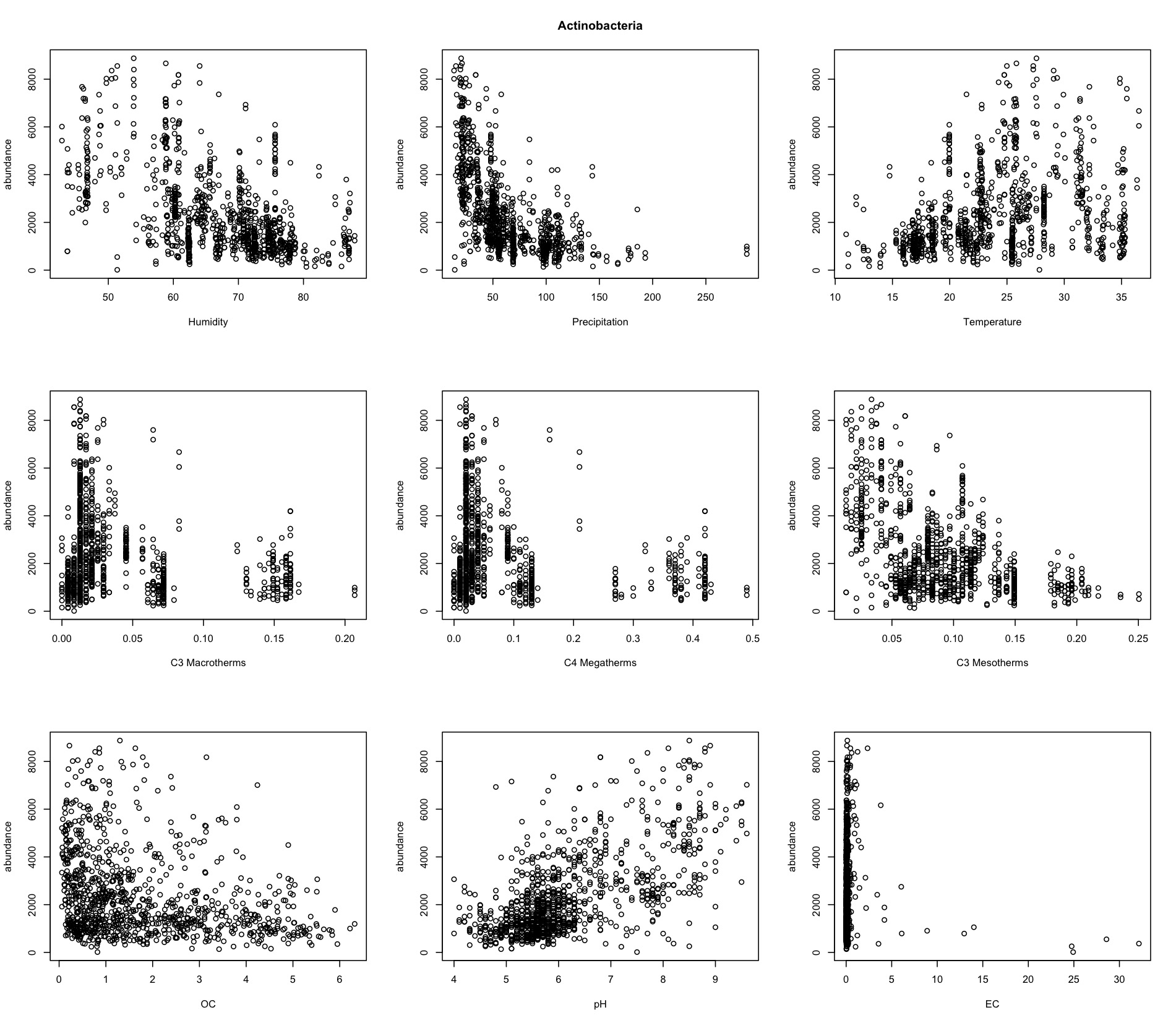

Supplement: Supplementary file 1 [file Data_Sheet_1.zip › Figure S3 Actinobacteria links_R1.png]

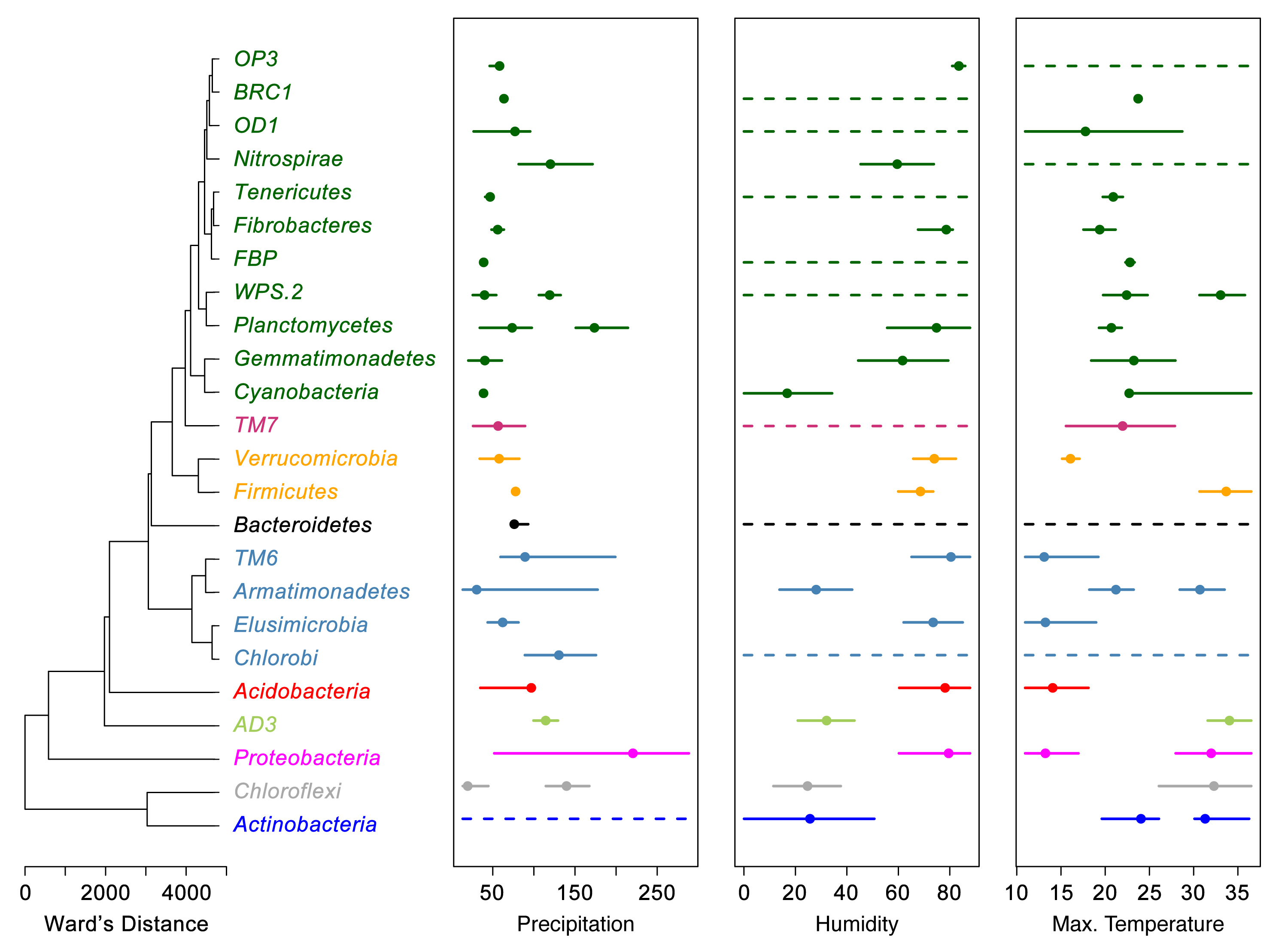

Supplement: Supplementary file 1 [file Data_Sheet_1.zip › Figure S4 phyla sem EHOF_climate.jpg]

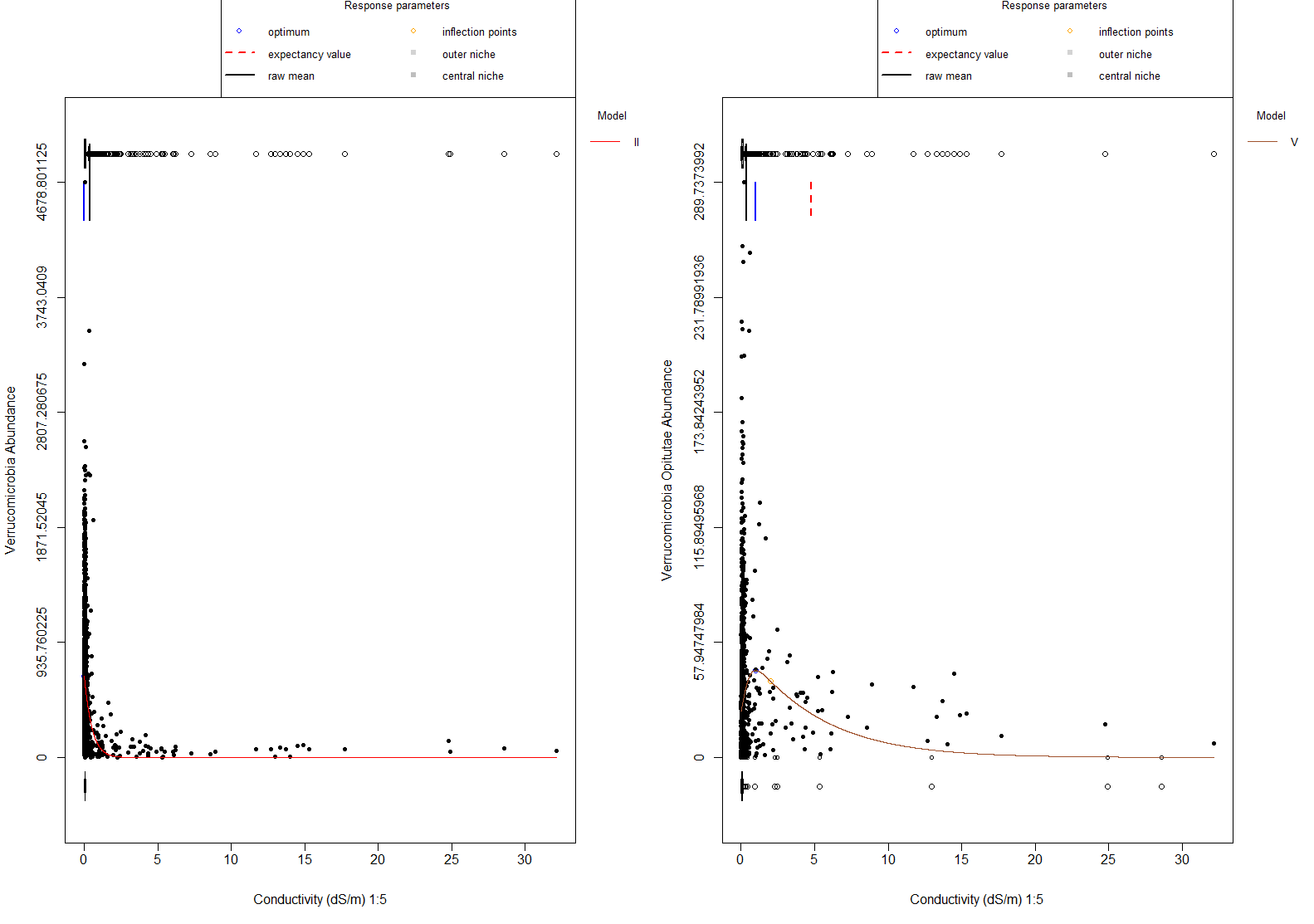

Supplement: Supplementary file 1 [file Data_Sheet_1.zip › Figure S5 verrucomicrobia ec niche.png]

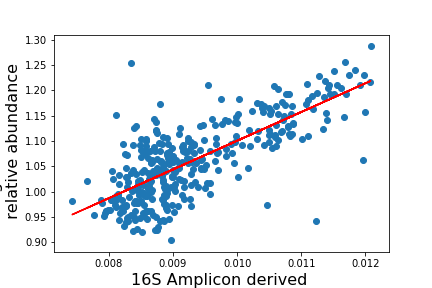

Supplement: Supplementary file 1 [file Data_Sheet_1.zip › figure_S63_methane_PC_SC_comparison.png]

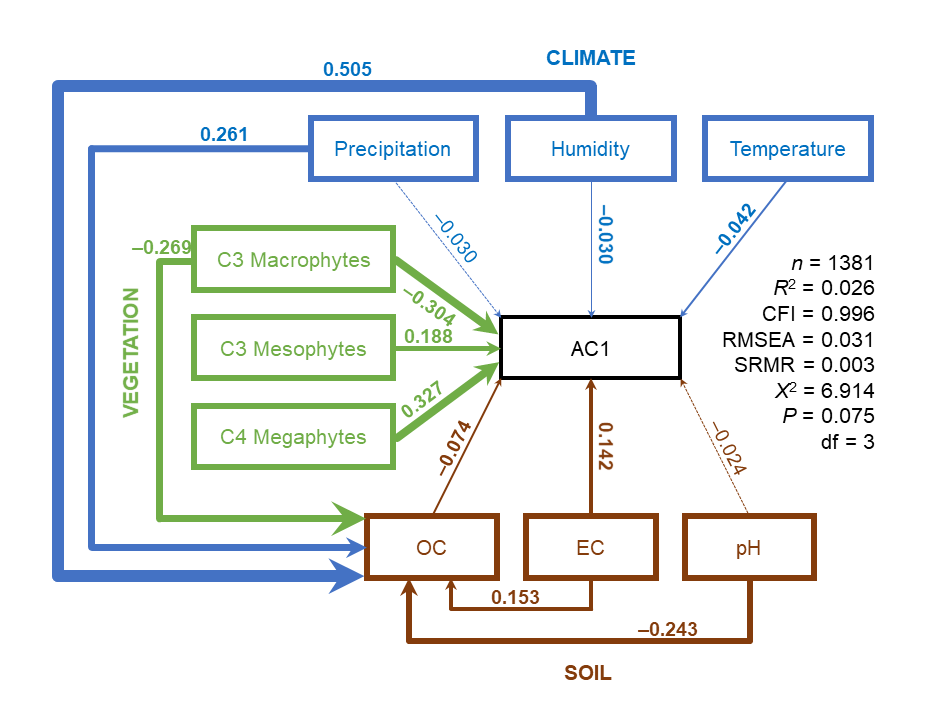

Supplement: Supplementary file 1 [file Data_Sheet_1.zip › Slide1.PNG]

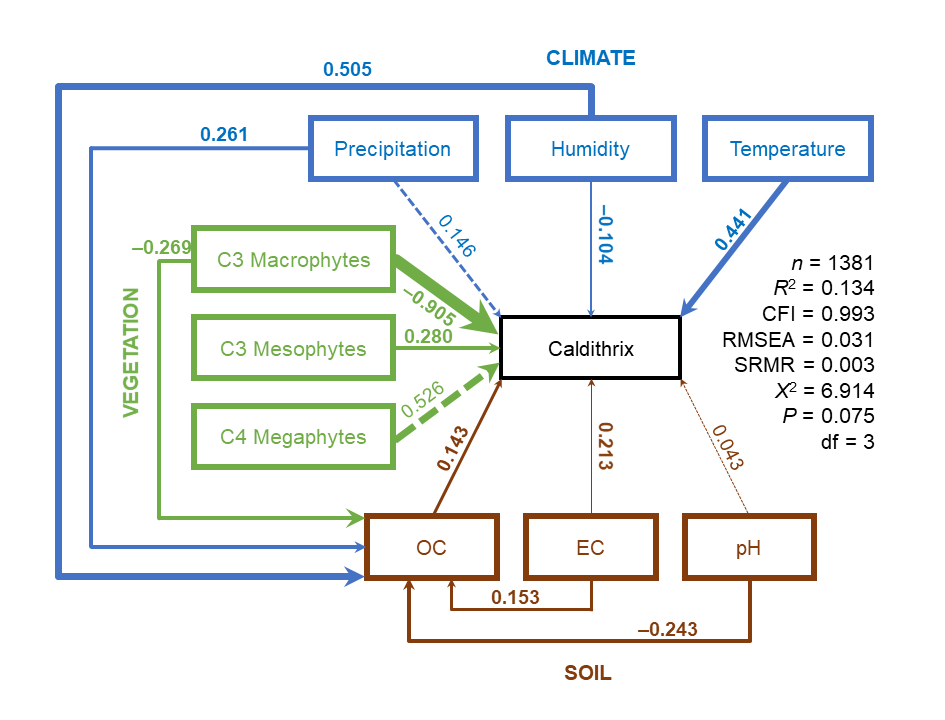

Supplement: Supplementary file 1 [file Data_Sheet_1.zip › Slide10.PNG]

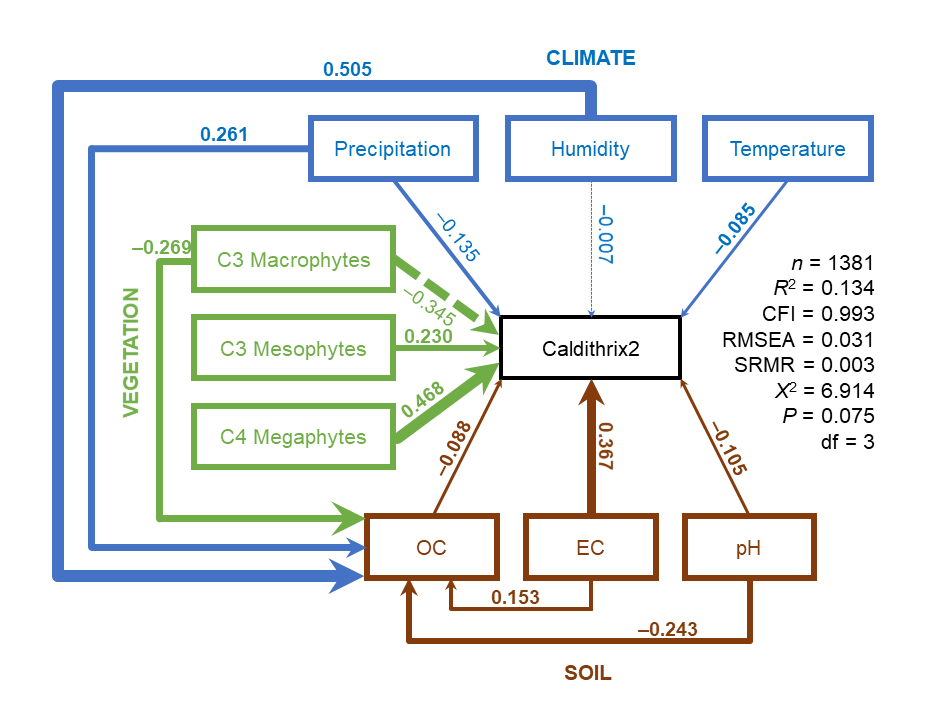

Supplement: Supplementary file 1 [file Data_Sheet_1.zip › Slide11.PNG]

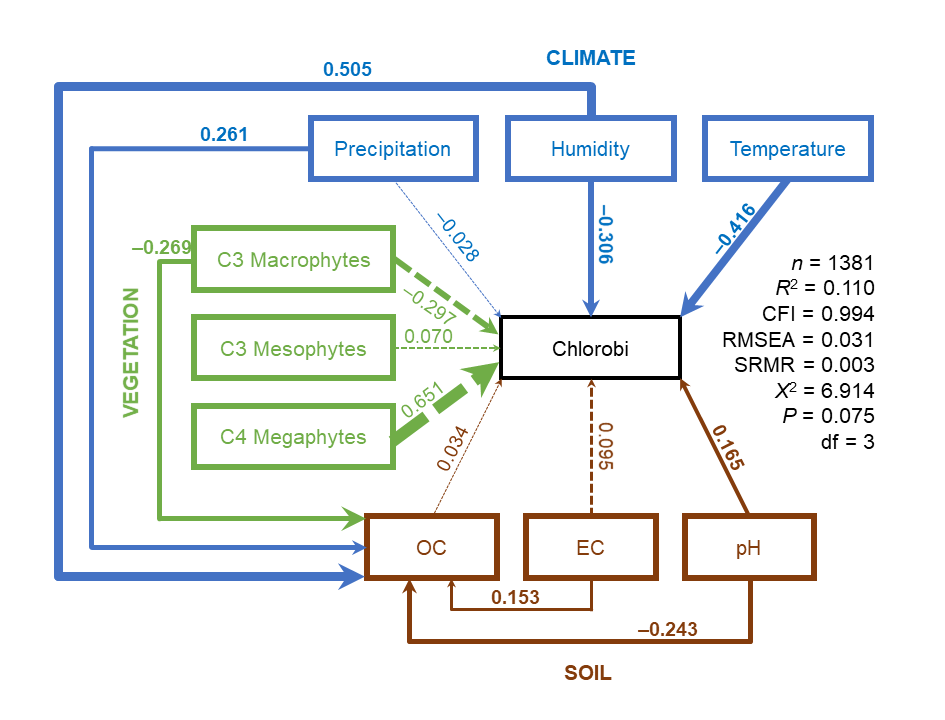

Supplement: Supplementary file 1 [file Data_Sheet_1.zip › Slide12.PNG]

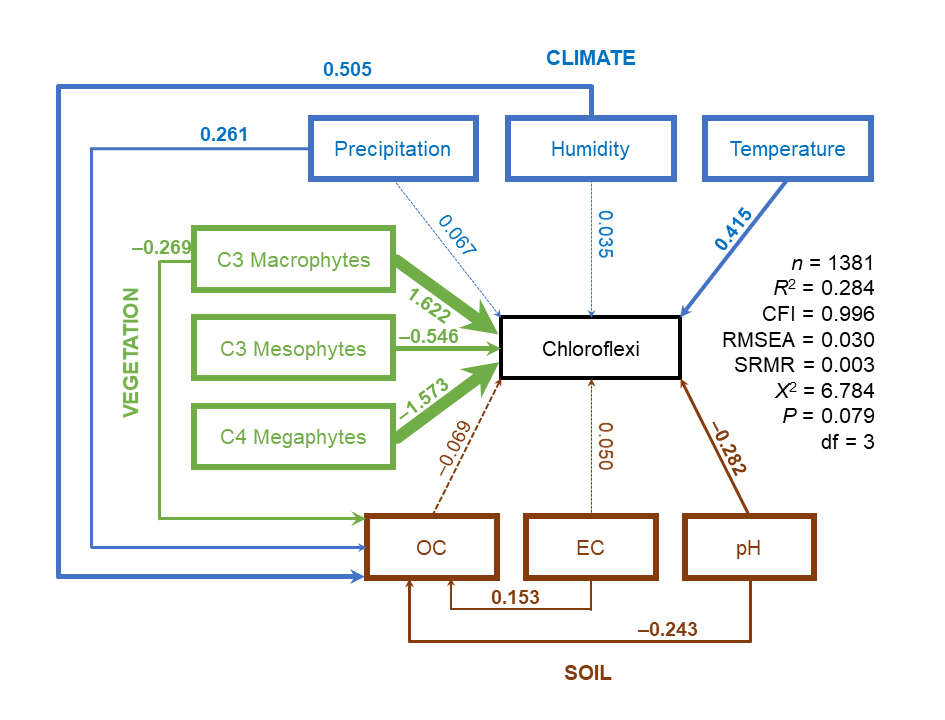

Supplement: Supplementary file 1 [file Data_Sheet_1.zip › Slide13.PNG]

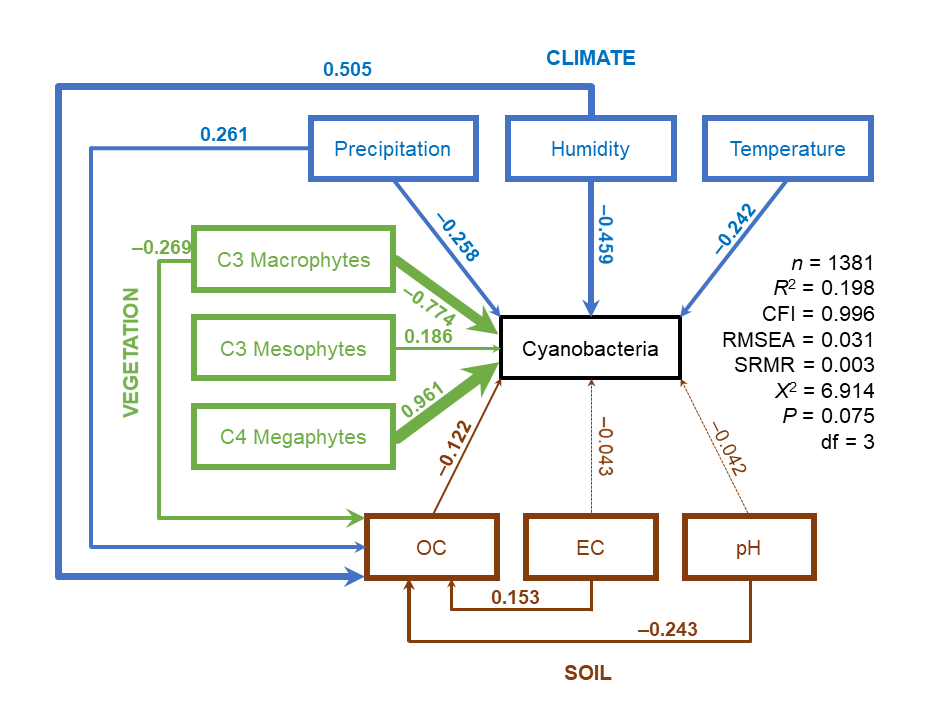

Supplement: Supplementary file 1 [file Data_Sheet_1.zip › Slide14.PNG]

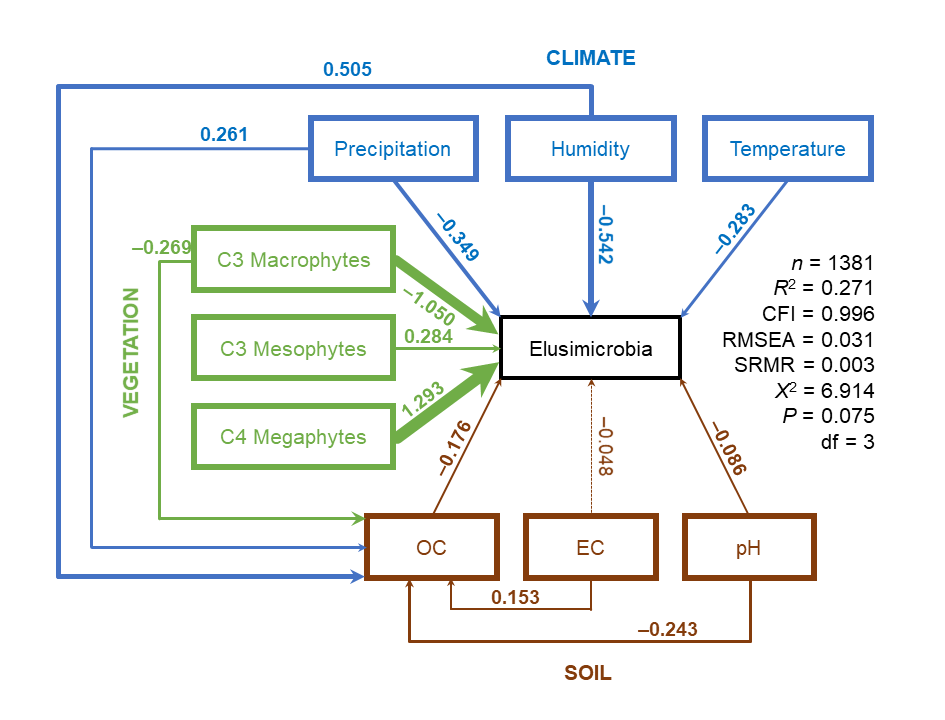

Supplement: Supplementary file 1 [file Data_Sheet_1.zip › Slide15.PNG]

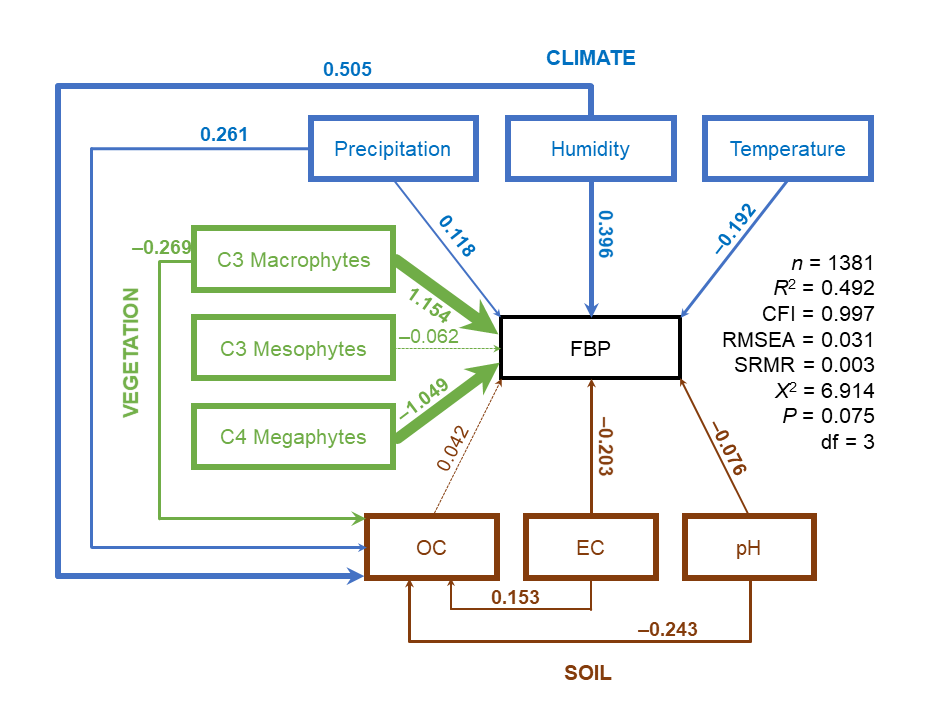

Supplement: Supplementary file 1 [file Data_Sheet_1.zip › Slide16.PNG]

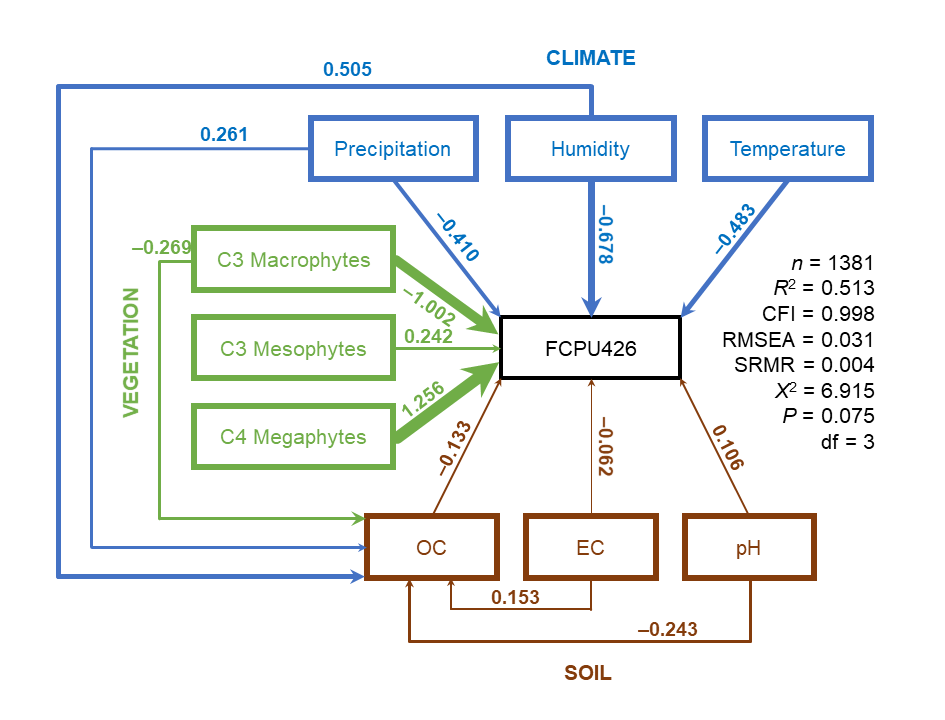

Supplement: Supplementary file 1 [file Data_Sheet_1.zip › Slide17.PNG]

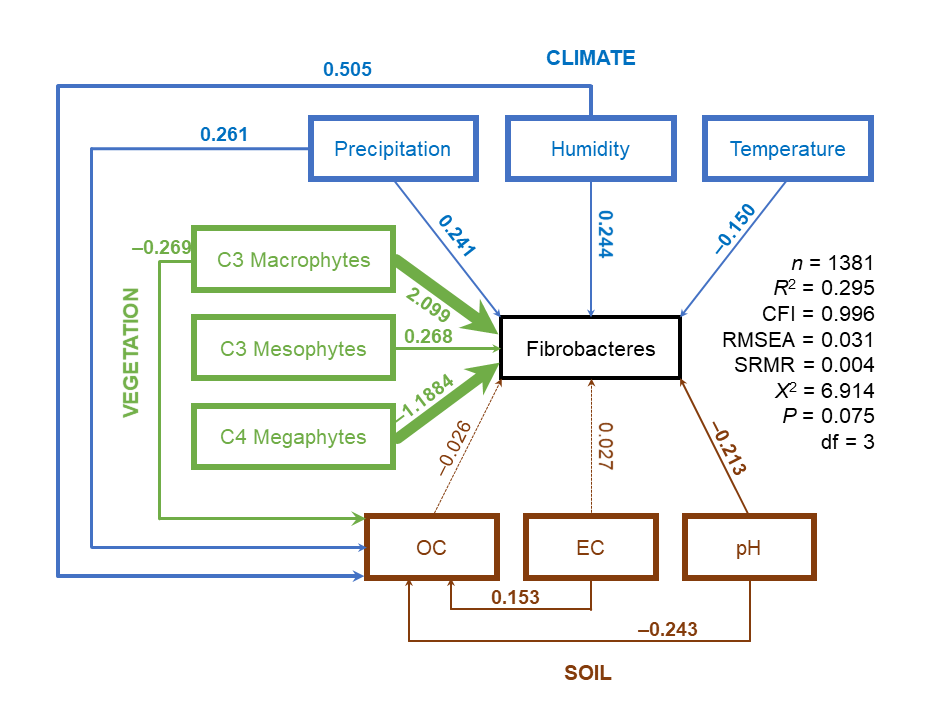

Supplement: Supplementary file 1 [file Data_Sheet_1.zip › Slide18.PNG]

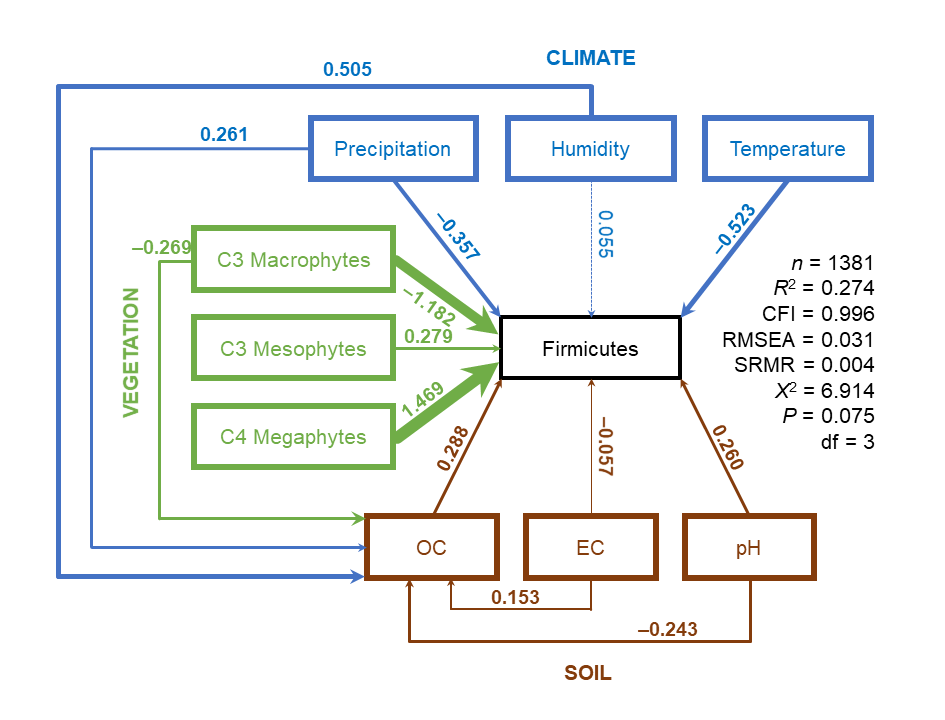

Supplement: Supplementary file 1 [file Data_Sheet_1.zip › Slide19.PNG]

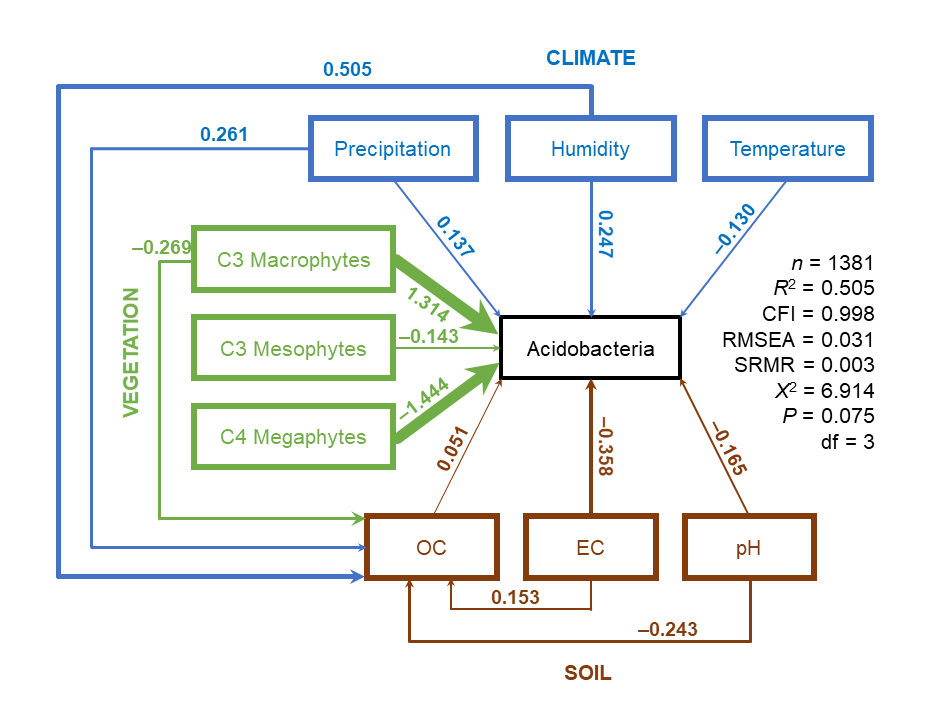

Supplement: Supplementary file 1 [file Data_Sheet_1.zip › Slide2.PNG]

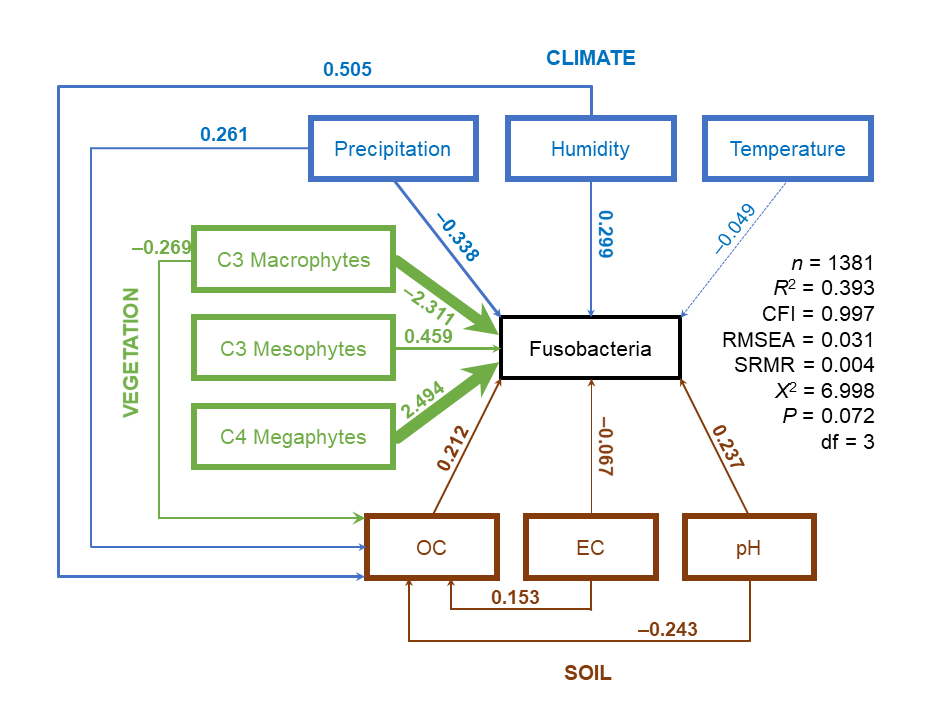

Supplement: Supplementary file 1 [file Data_Sheet_1.zip › Slide20.PNG]

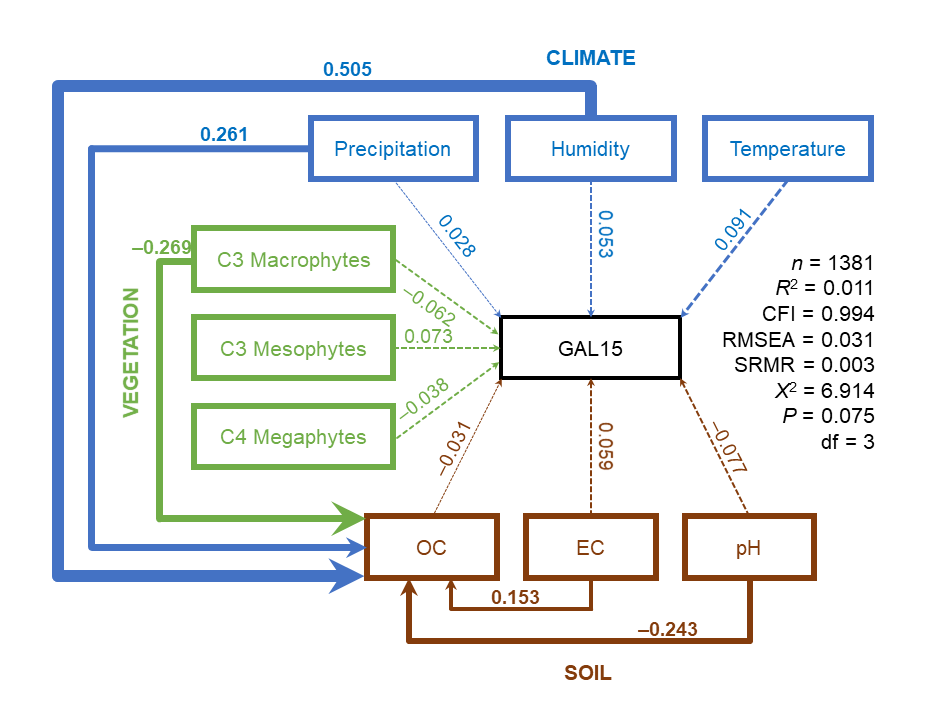

Supplement: Supplementary file 1 [file Data_Sheet_1.zip › Slide21.PNG]

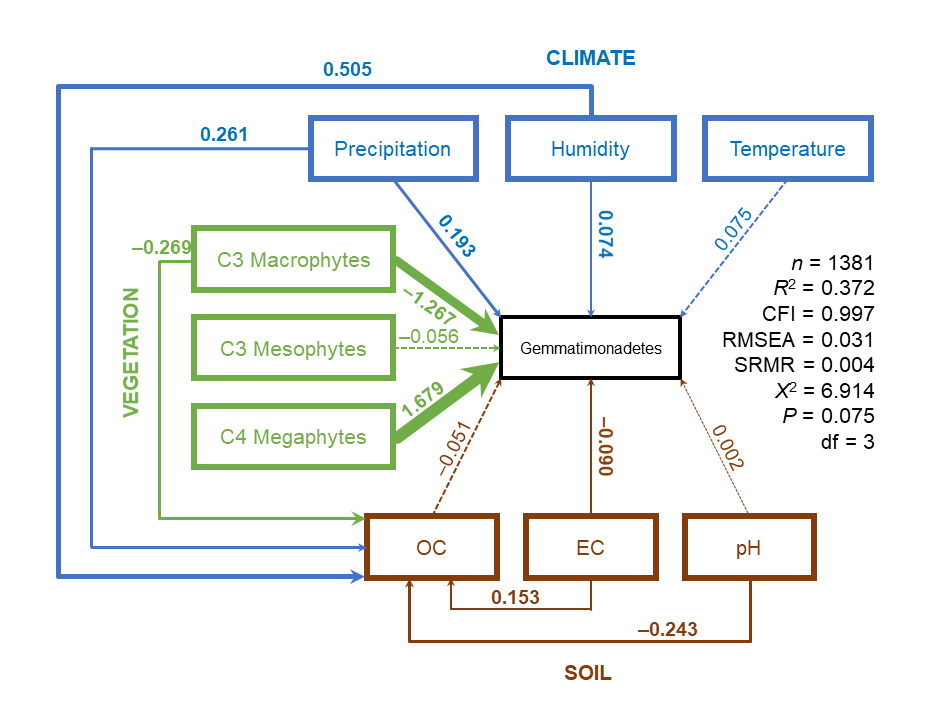

Supplement: Supplementary file 1 [file Data_Sheet_1.zip › Slide22.PNG]

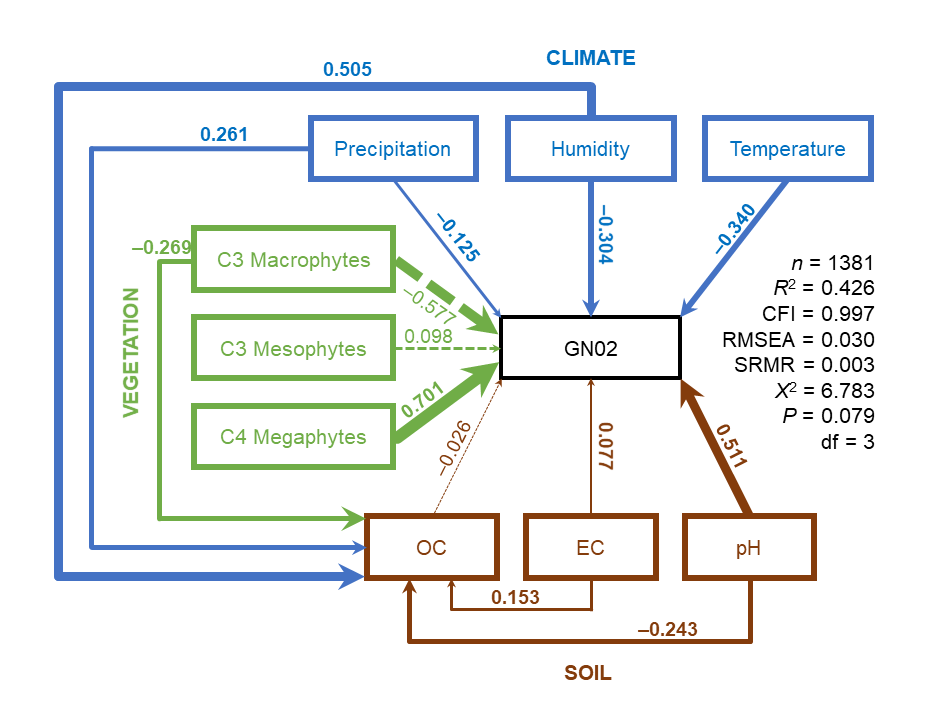

Supplement: Supplementary file 1 [file Data_Sheet_1.zip › Slide23.PNG]

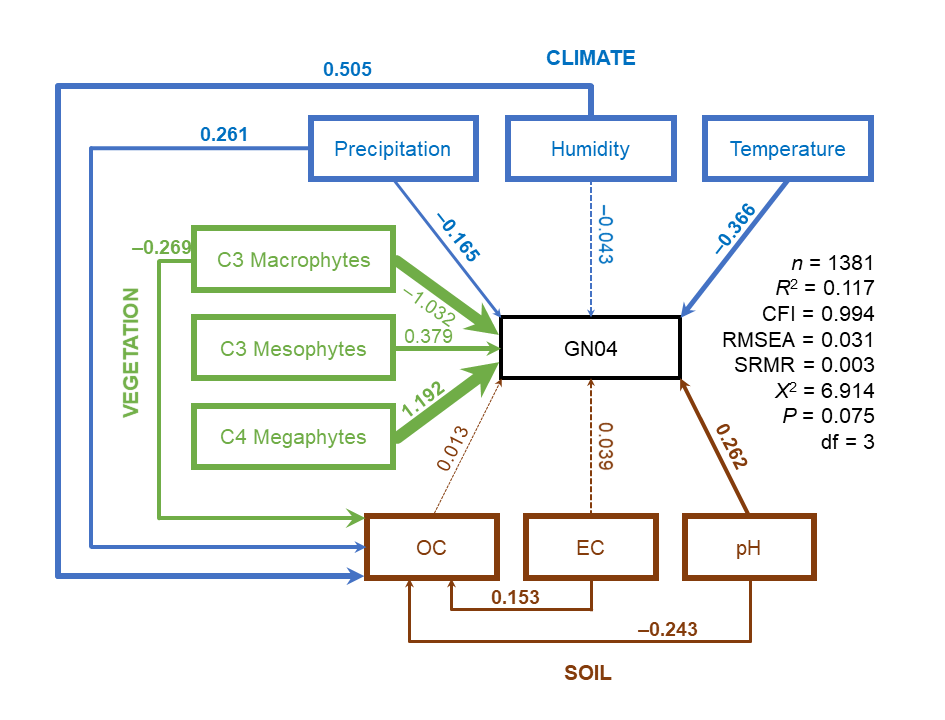

Supplement: Supplementary file 1 [file Data_Sheet_1.zip › Slide24.PNG]

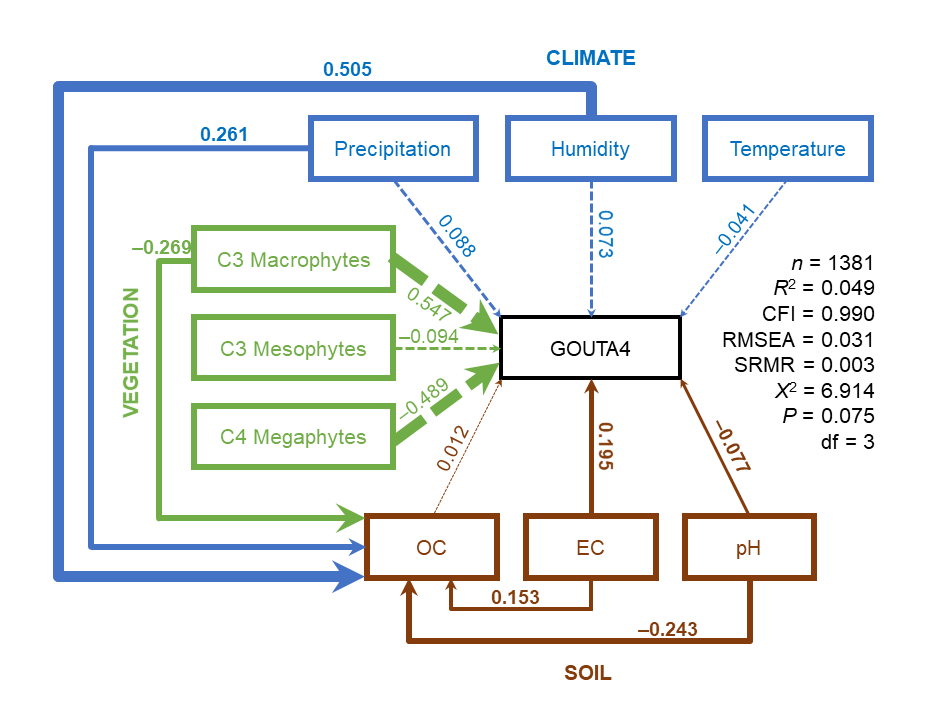

Supplement: Supplementary file 1 [file Data_Sheet_1.zip › Slide25.PNG]

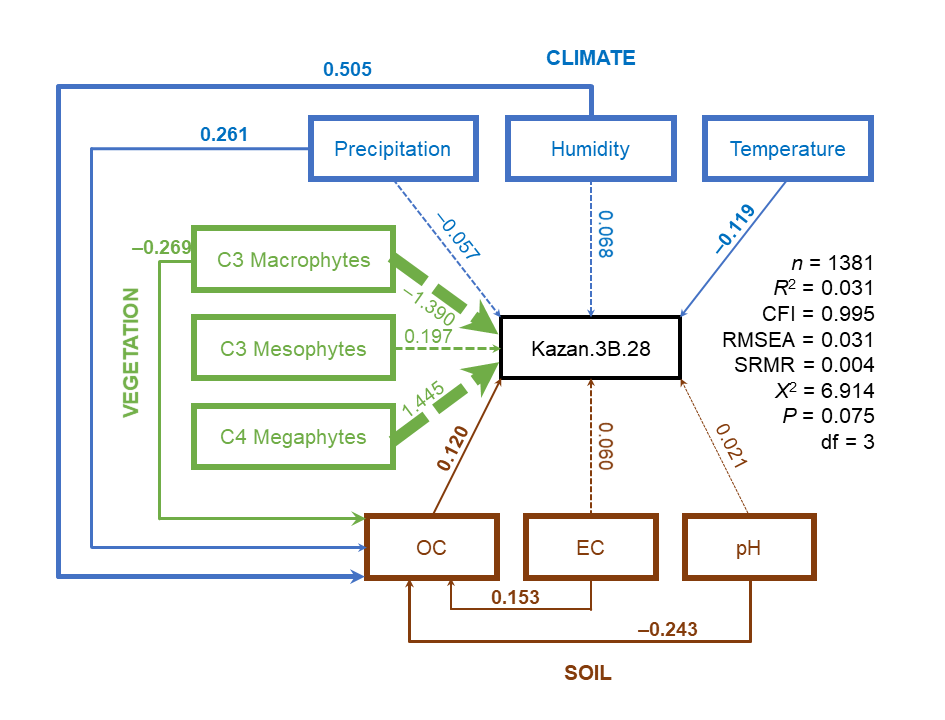

Supplement: Supplementary file 1 [file Data_Sheet_1.zip › Slide26.PNG]

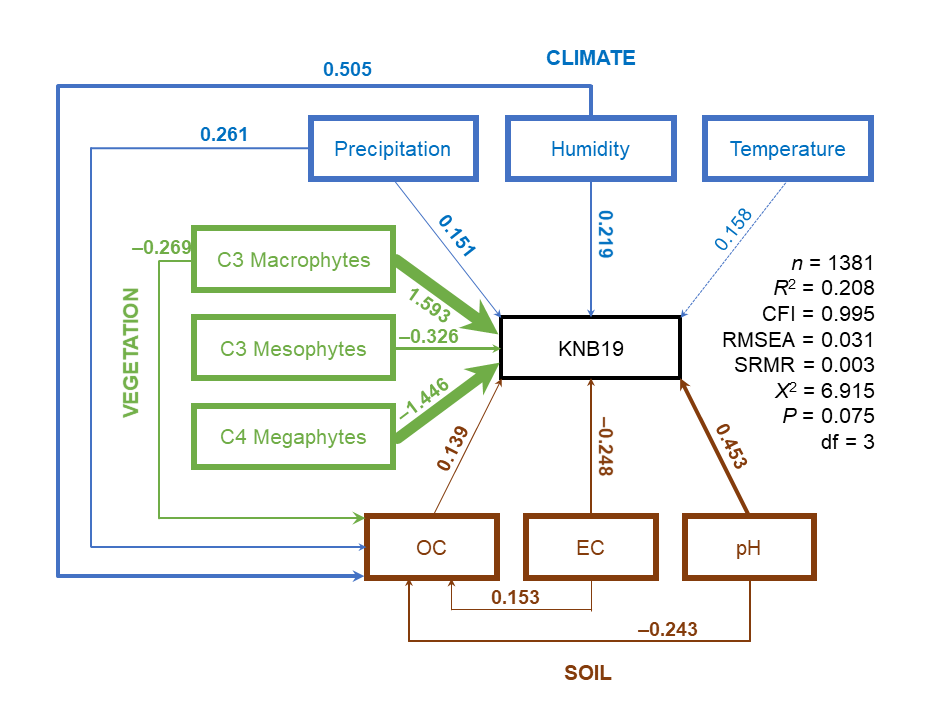

Supplement: Supplementary file 1 [file Data_Sheet_1.zip › Slide27.PNG]

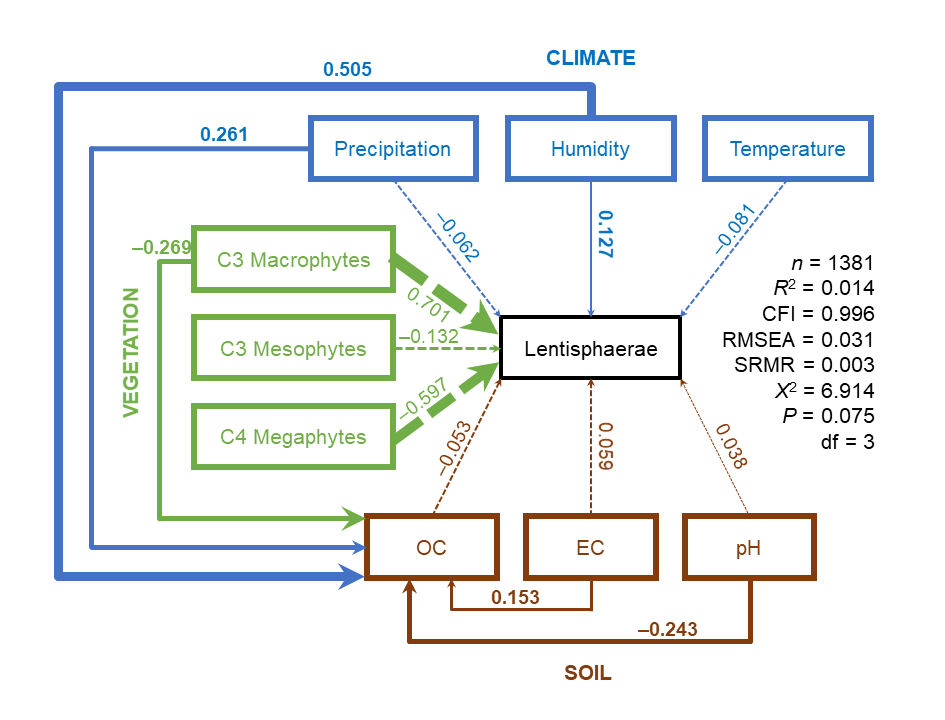

Supplement: Supplementary file 1 [file Data_Sheet_1.zip › Slide28.PNG]

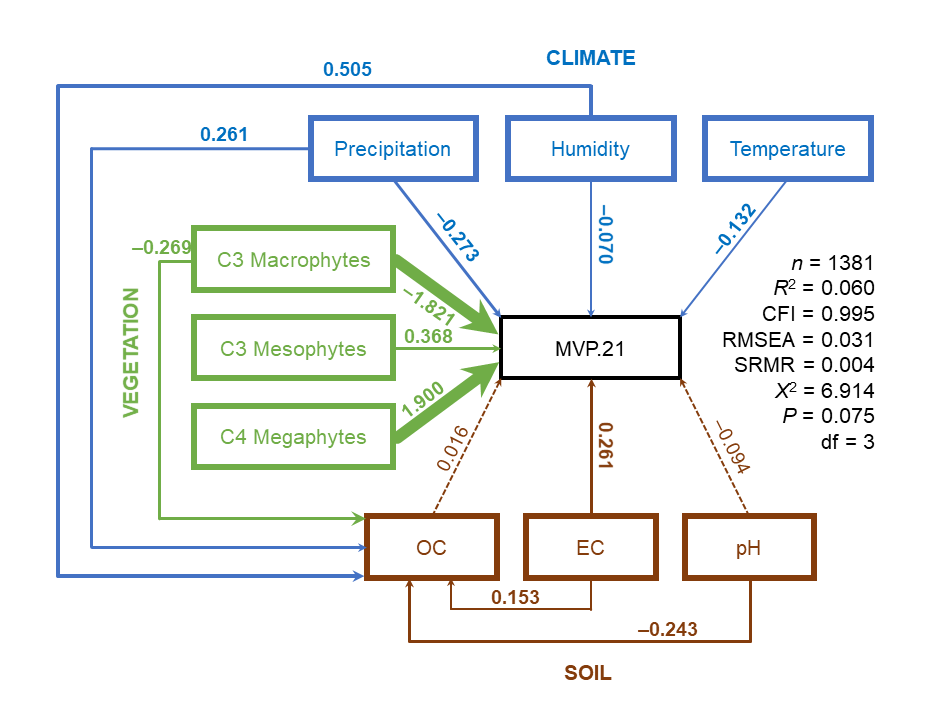

Supplement: Supplementary file 1 [file Data_Sheet_1.zip › Slide29.PNG]

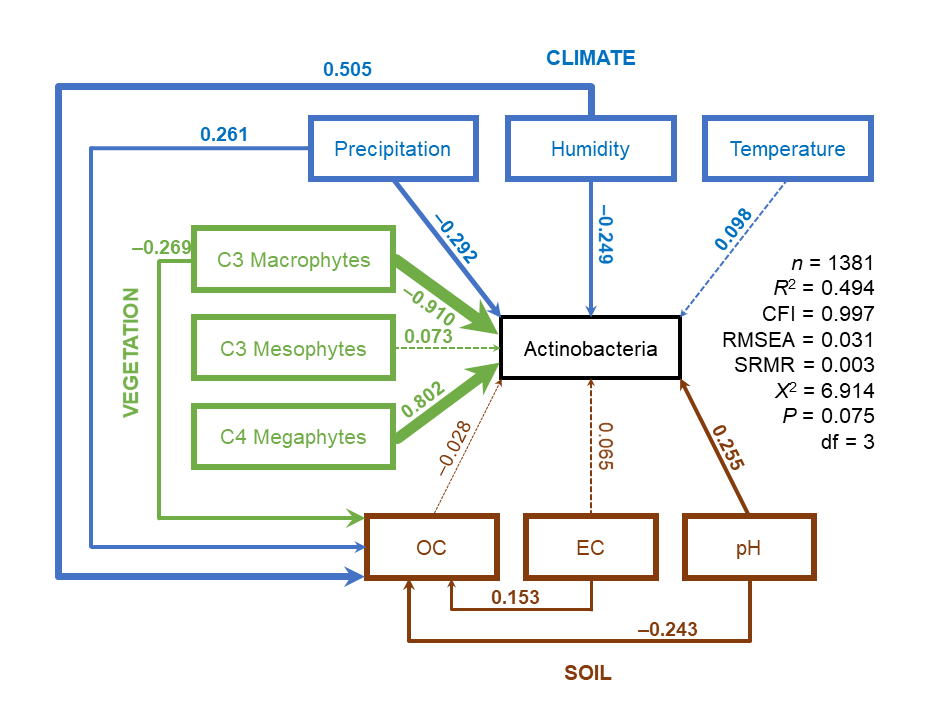

Supplement: Supplementary file 1 [file Data_Sheet_1.zip › Slide3.PNG]

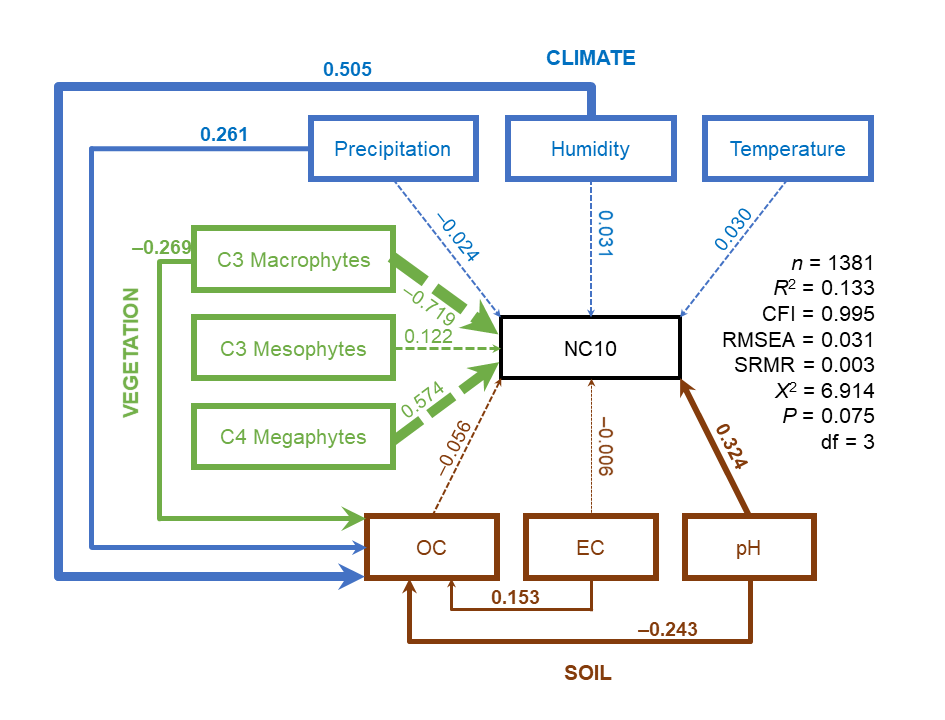

Supplement: Supplementary file 1 [file Data_Sheet_1.zip › Slide30.PNG]

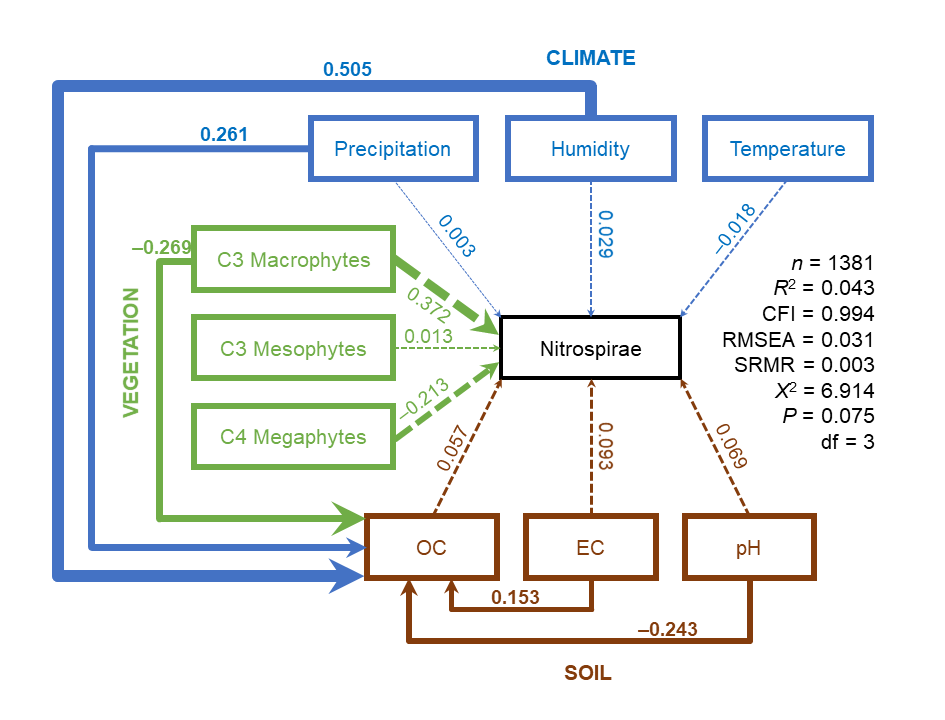

Supplement: Supplementary file 1 [file Data_Sheet_1.zip › Slide31.PNG]

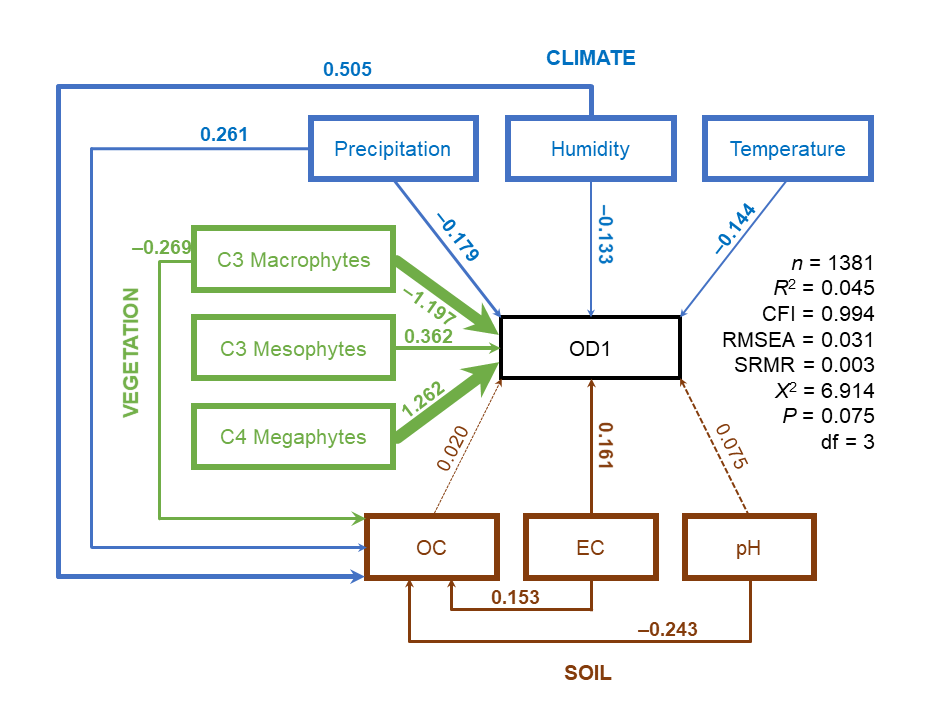

Supplement: Supplementary file 1 [file Data_Sheet_1.zip › Slide32.PNG]

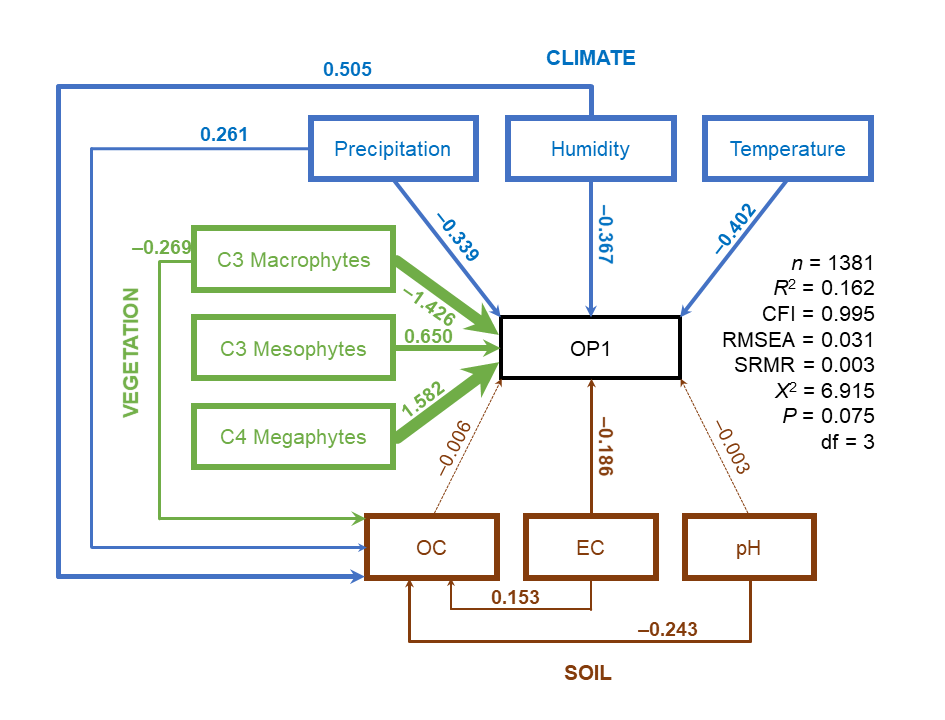

Supplement: Supplementary file 1 [file Data_Sheet_1.zip › Slide33.PNG]

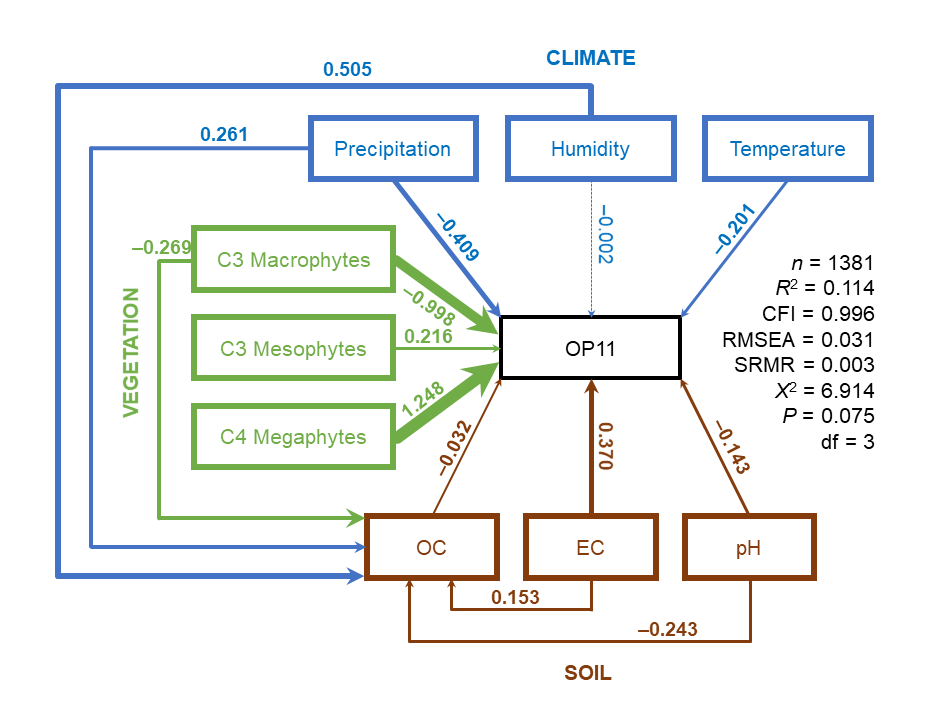

Supplement: Supplementary file 1 [file Data_Sheet_1.zip › Slide34.PNG]

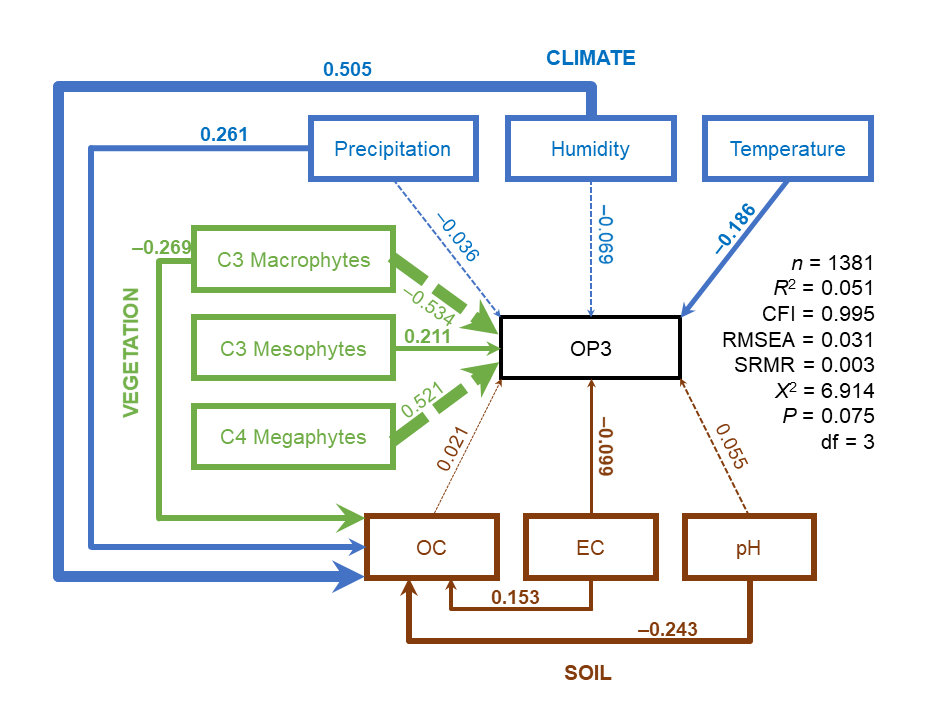

Supplement: Supplementary file 1 [file Data_Sheet_1.zip › Slide35.PNG]

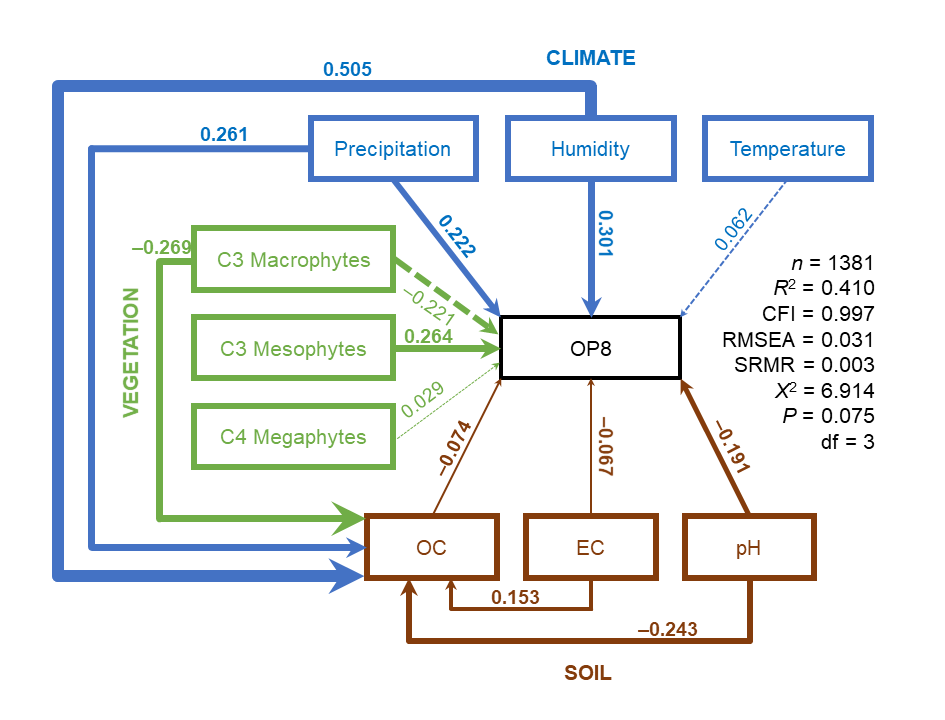

Supplement: Supplementary file 1 [file Data_Sheet_1.zip › Slide36.PNG]

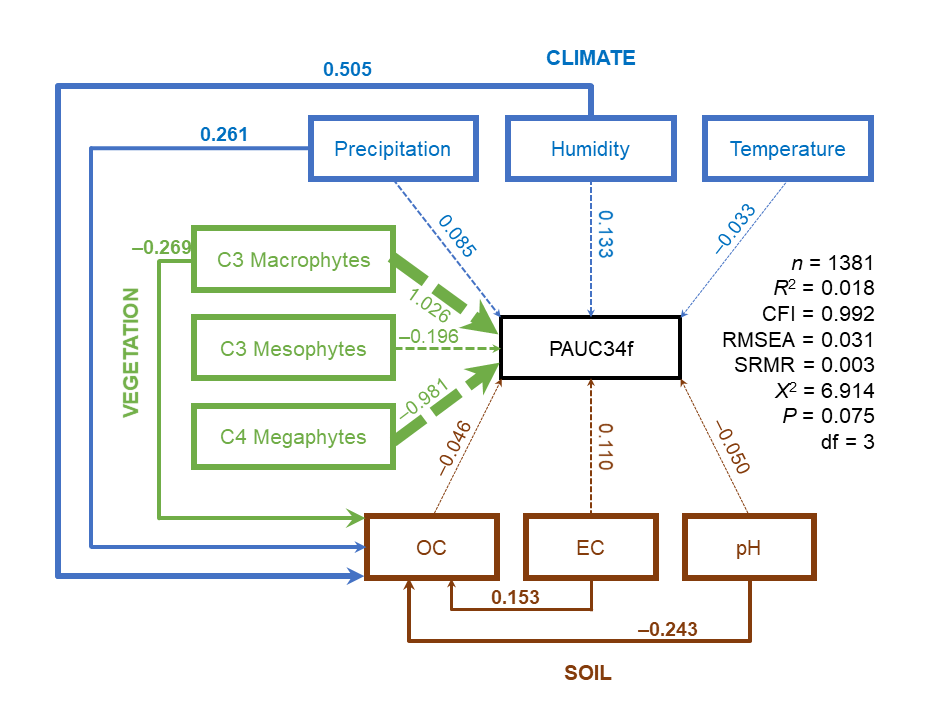

Supplement: Supplementary file 1 [file Data_Sheet_1.zip › Slide37.PNG]

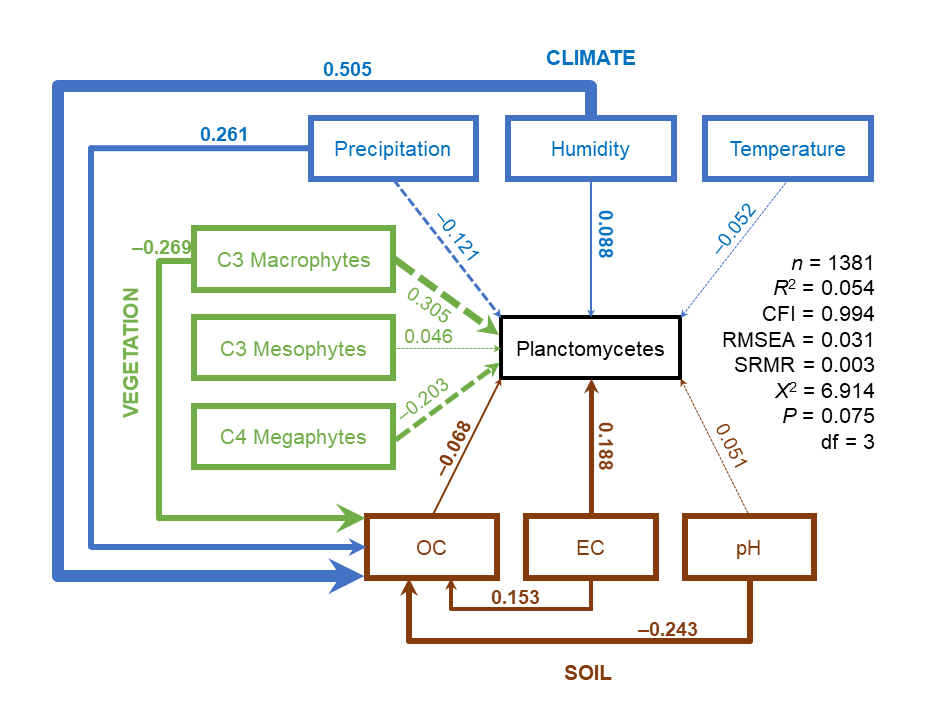

Supplement: Supplementary file 1 [file Data_Sheet_1.zip › Slide38.PNG]

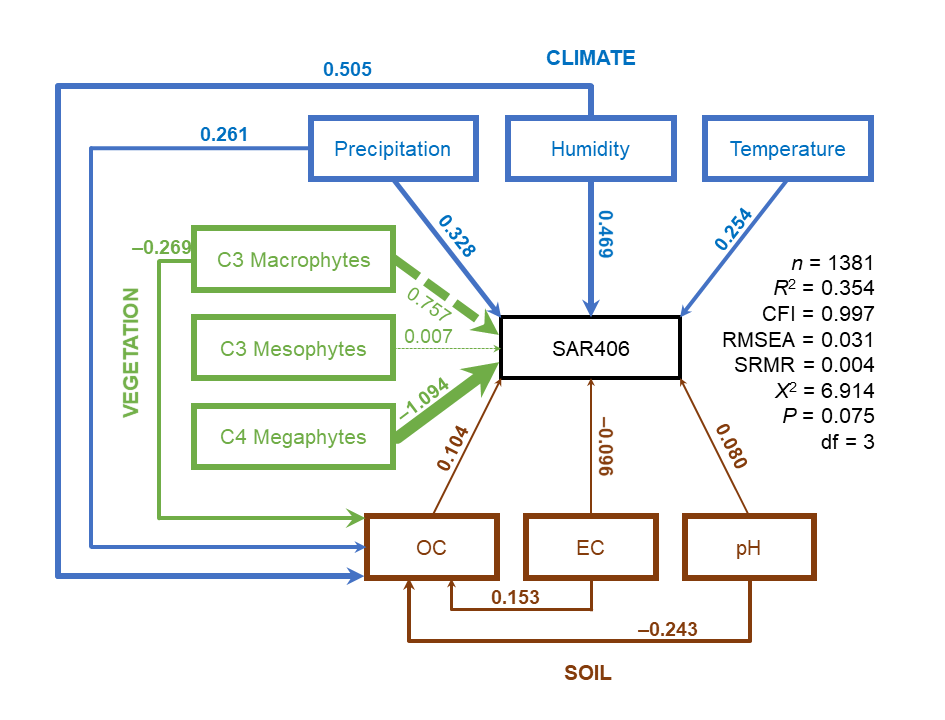

Supplement: Supplementary file 1 [file Data_Sheet_1.zip › Slide39.PNG]

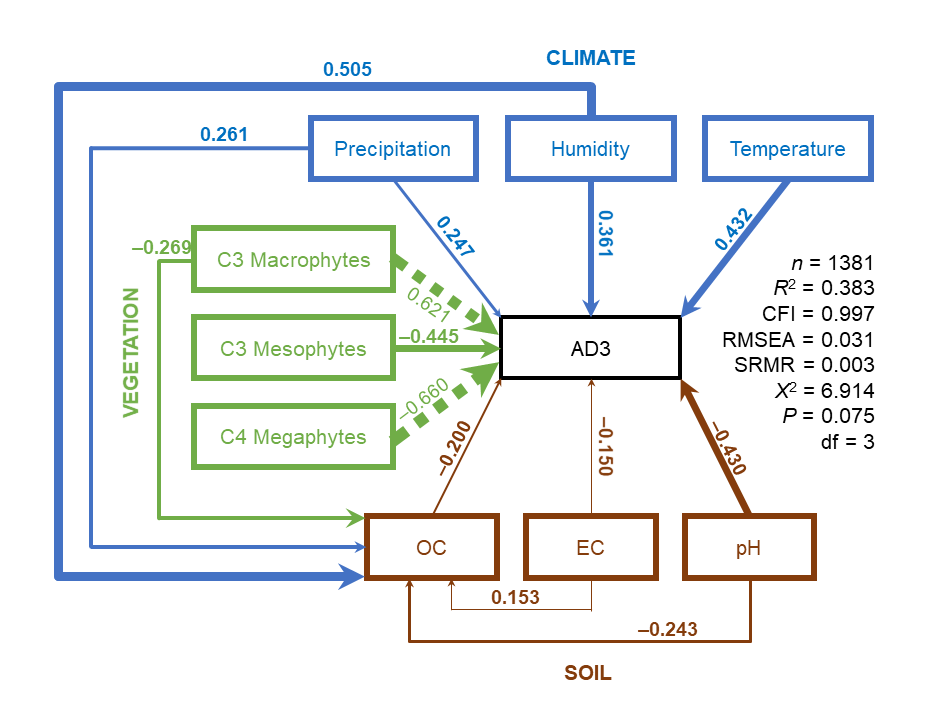

Supplement: Supplementary file 1 [file Data_Sheet_1.zip › Slide4.PNG]

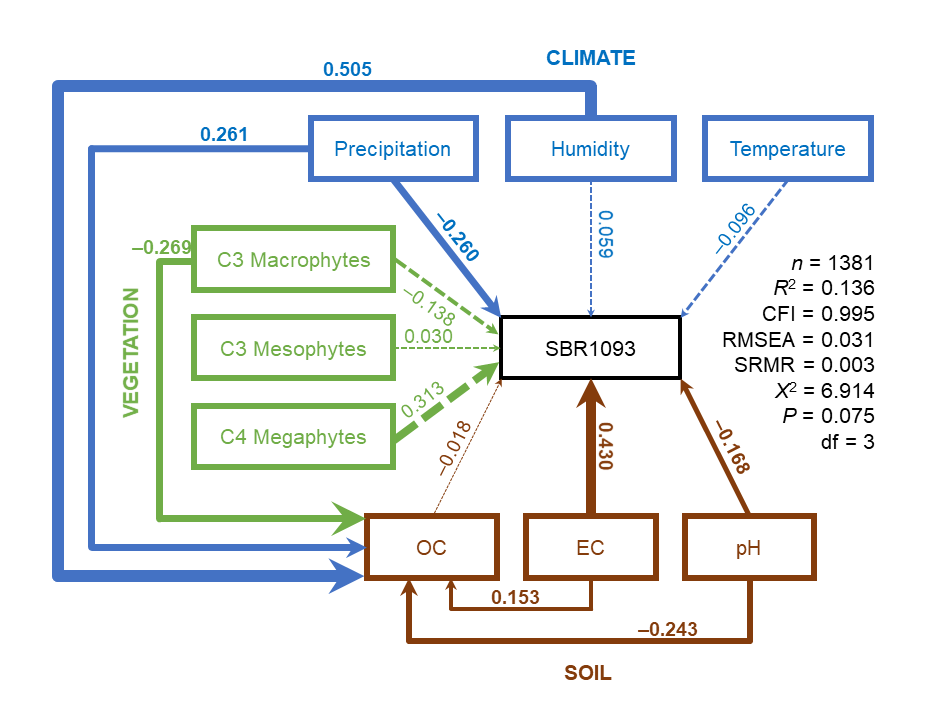

Supplement: Supplementary file 1 [file Data_Sheet_1.zip › Slide40.PNG]

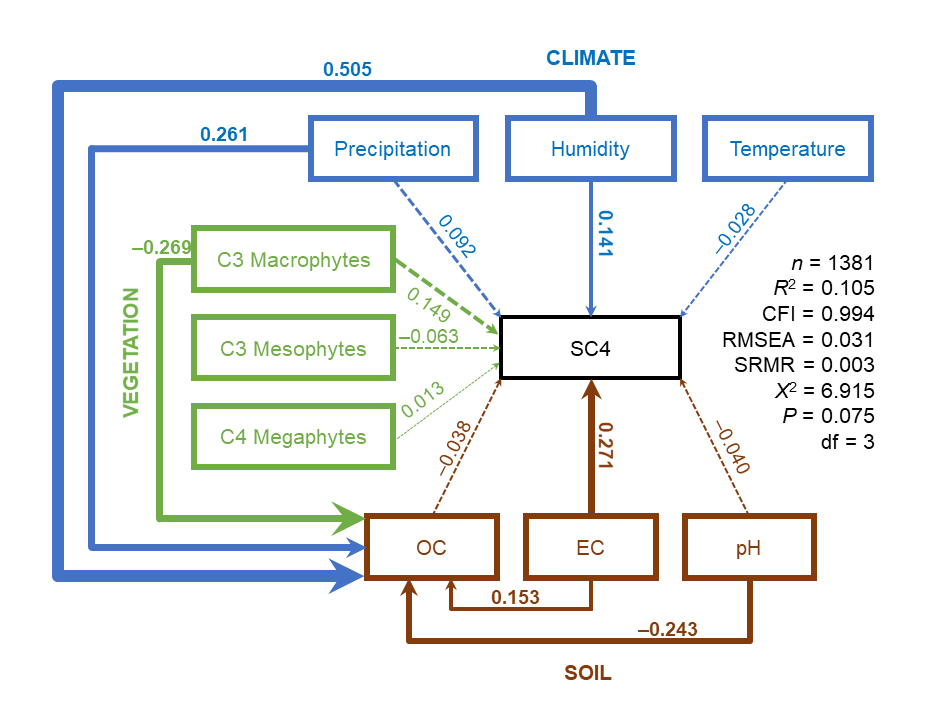

Supplement: Supplementary file 1 [file Data_Sheet_1.zip › Slide41.PNG]

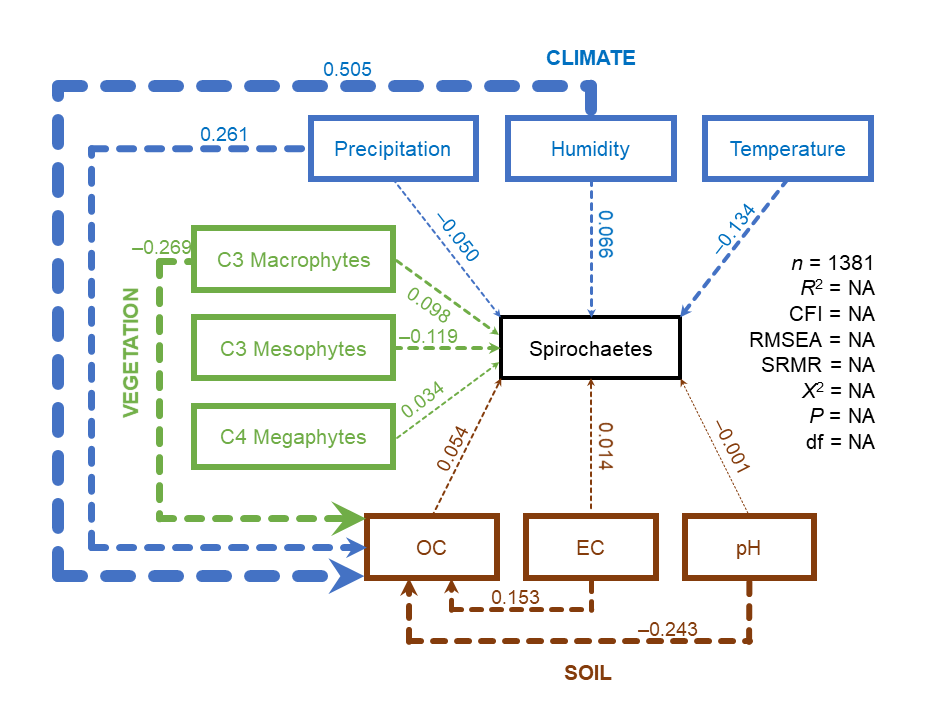

Supplement: Supplementary file 1 [file Data_Sheet_1.zip › Slide42.PNG]

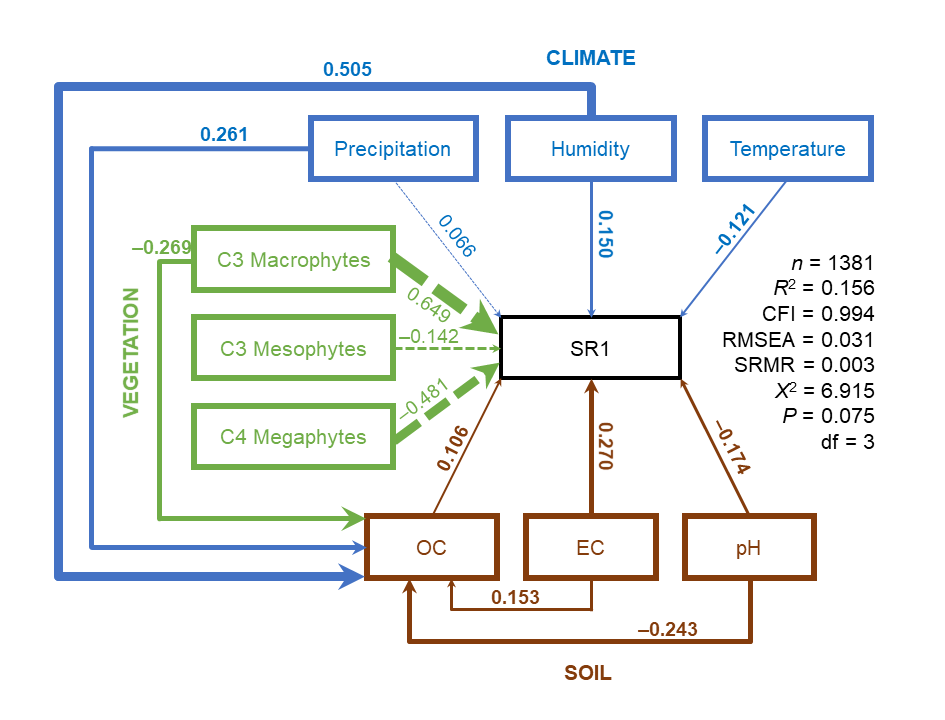

Supplement: Supplementary file 1 [file Data_Sheet_1.zip › Slide43.PNG]

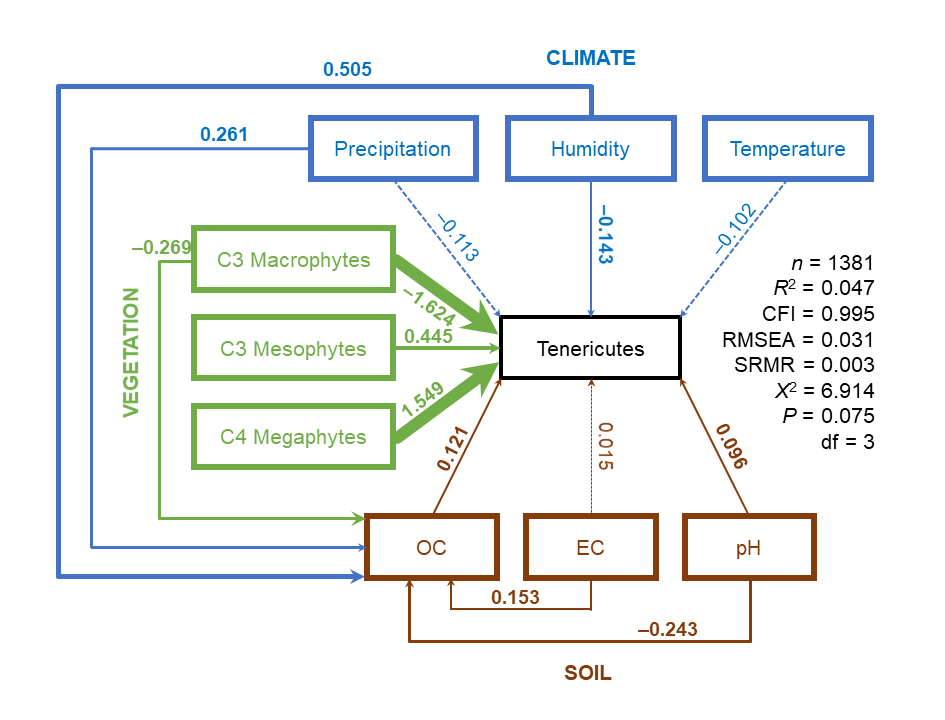

Supplement: Supplementary file 1 [file Data_Sheet_1.zip › Slide44.PNG]

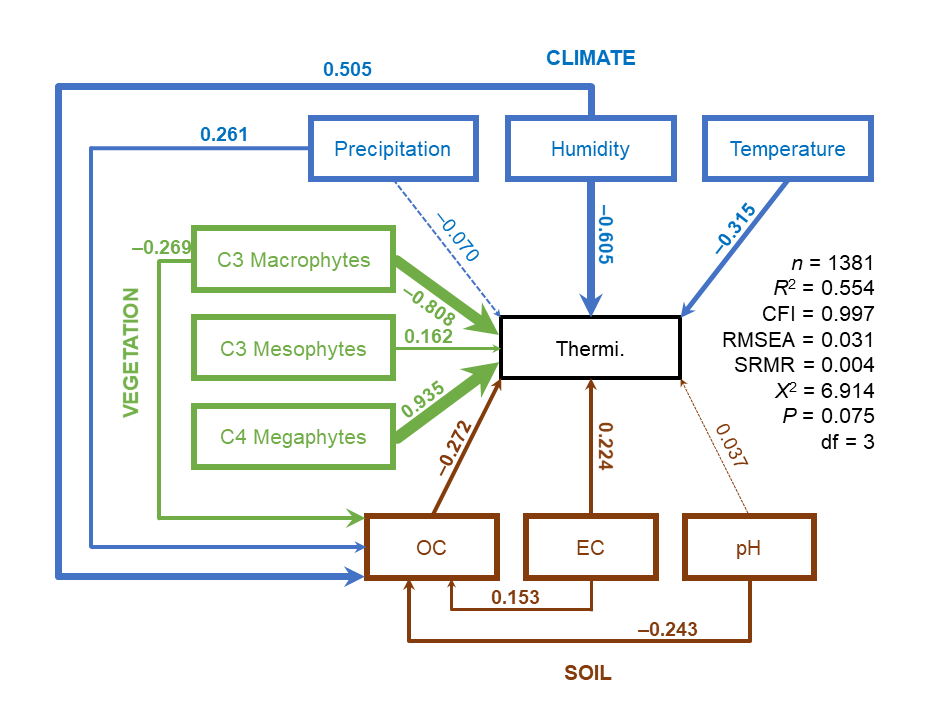

Supplement: Supplementary file 1 [file Data_Sheet_1.zip › Slide45.PNG]

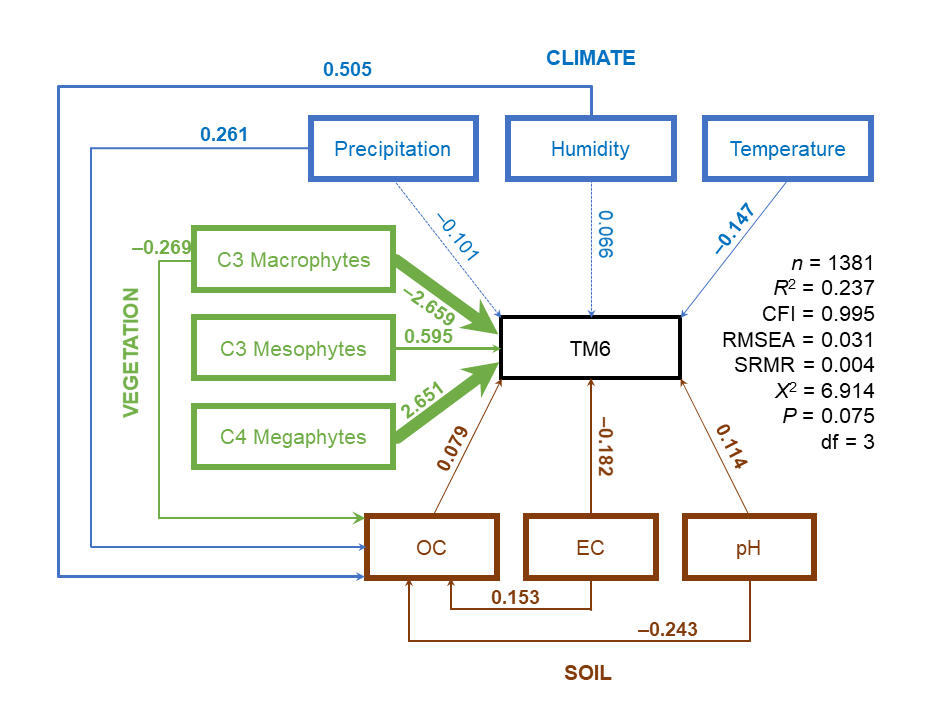

Supplement: Supplementary file 1 [file Data_Sheet_1.zip › Slide46.PNG]

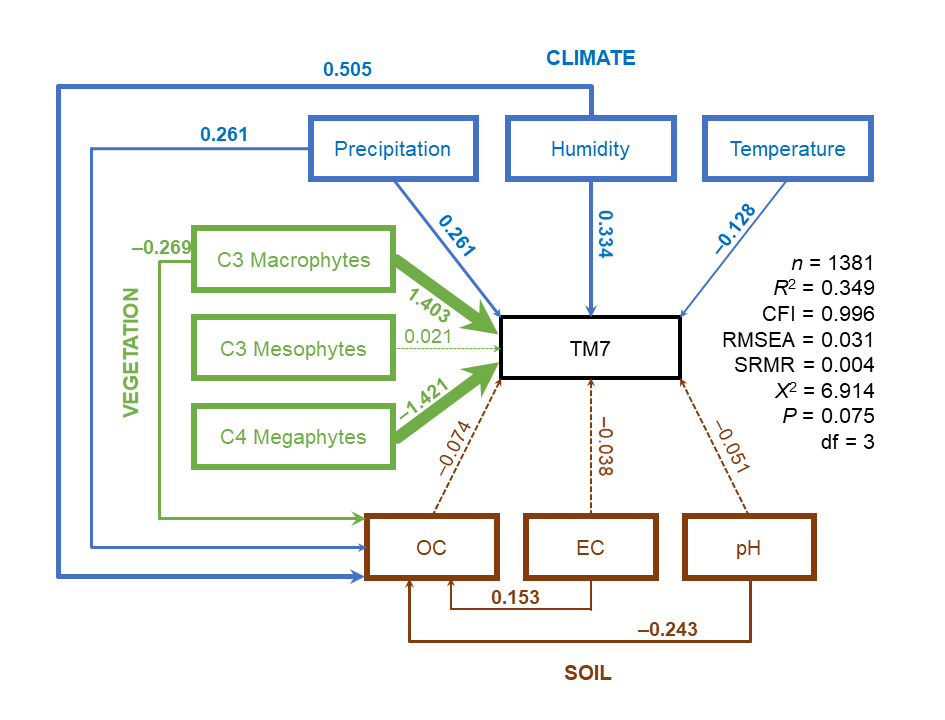

Supplement: Supplementary file 1 [file Data_Sheet_1.zip › Slide47.PNG]

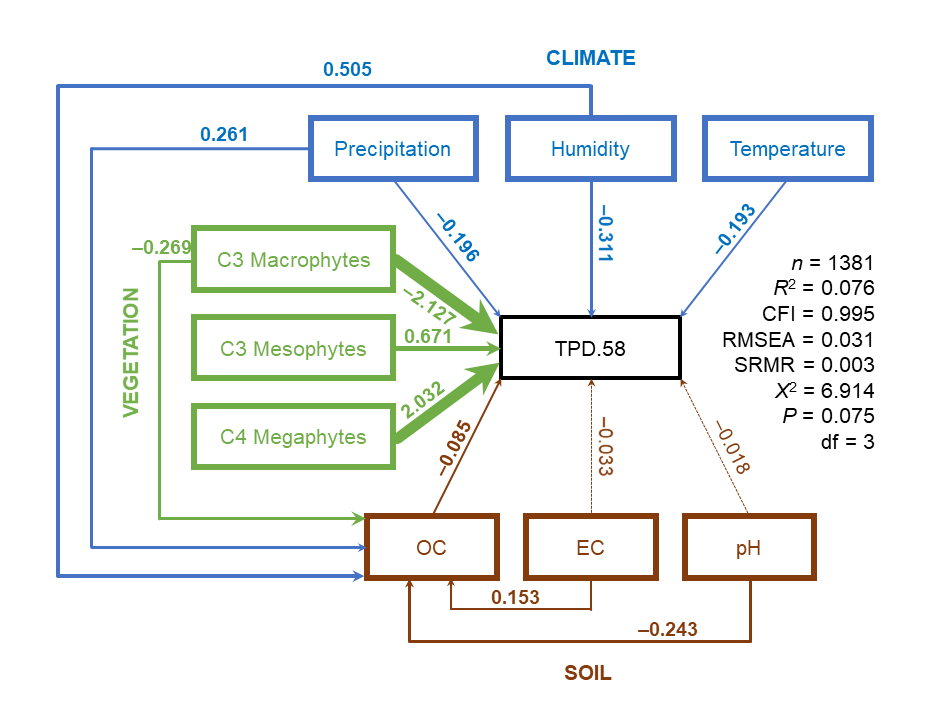

Supplement: Supplementary file 1 [file Data_Sheet_1.zip › Slide48.PNG]

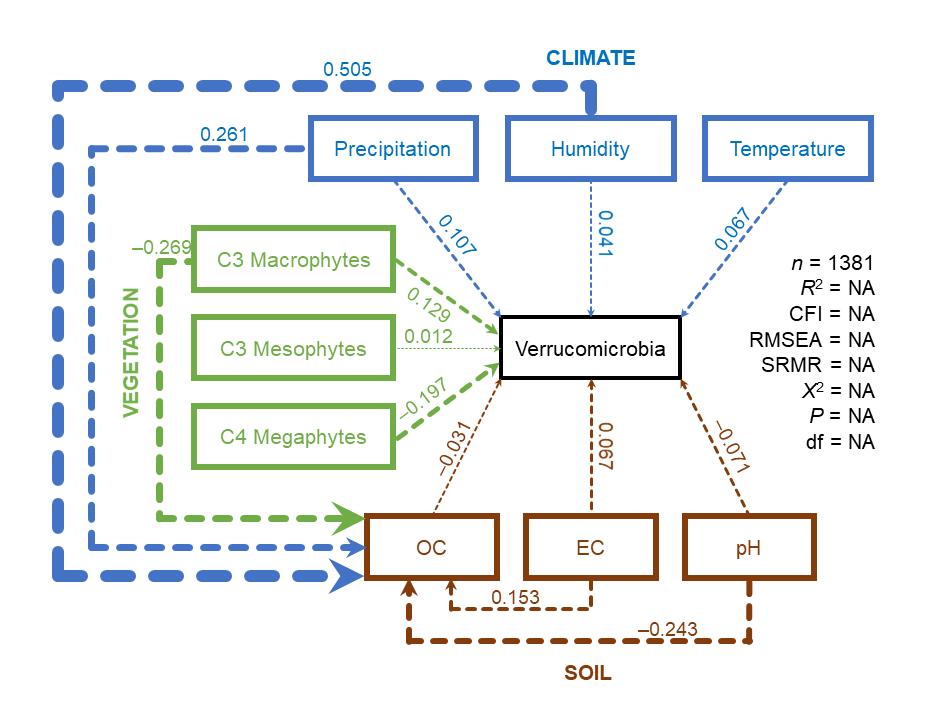

Supplement: Supplementary file 1 [file Data_Sheet_1.zip › Slide49.PNG]

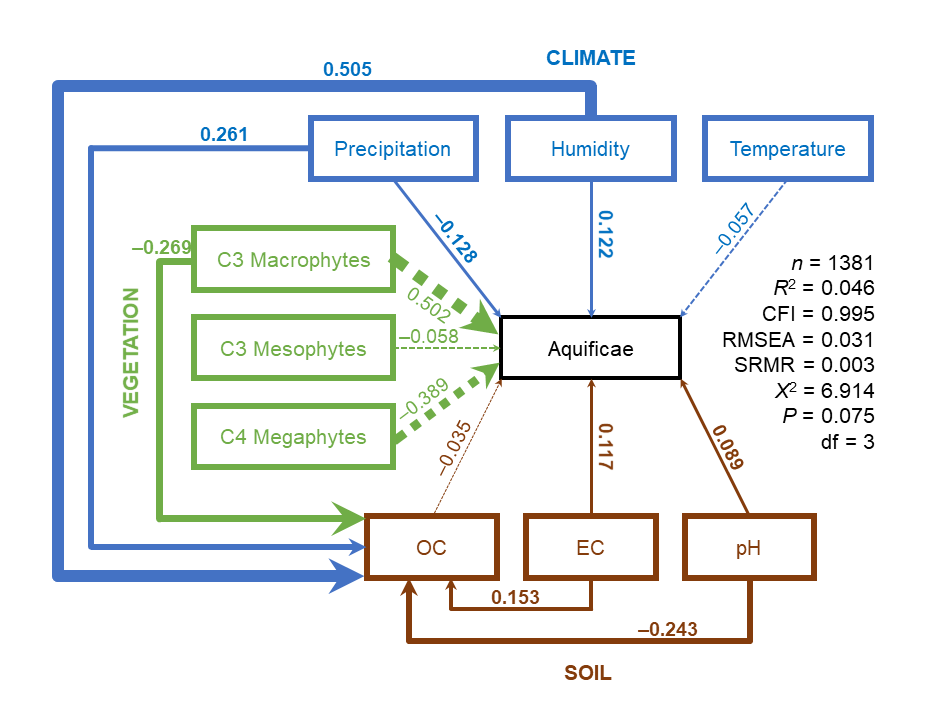

Supplement: Supplementary file 1 [file Data_Sheet_1.zip › Slide5.PNG]

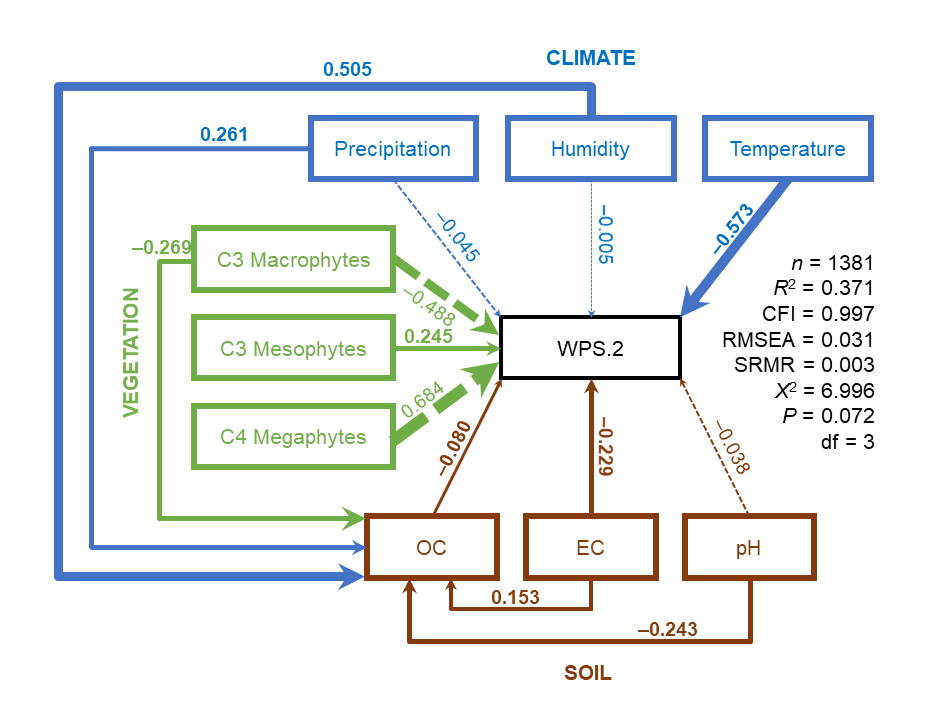

Supplement: Supplementary file 1 [file Data_Sheet_1.zip › Slide50.PNG]

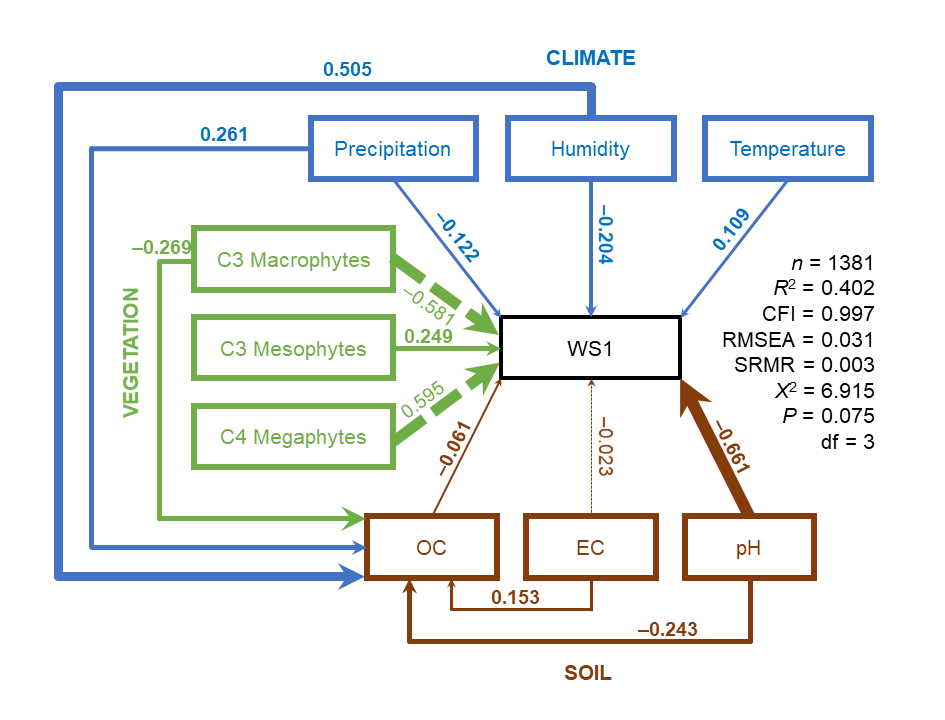

Supplement: Supplementary file 1 [file Data_Sheet_1.zip › Slide51.PNG]

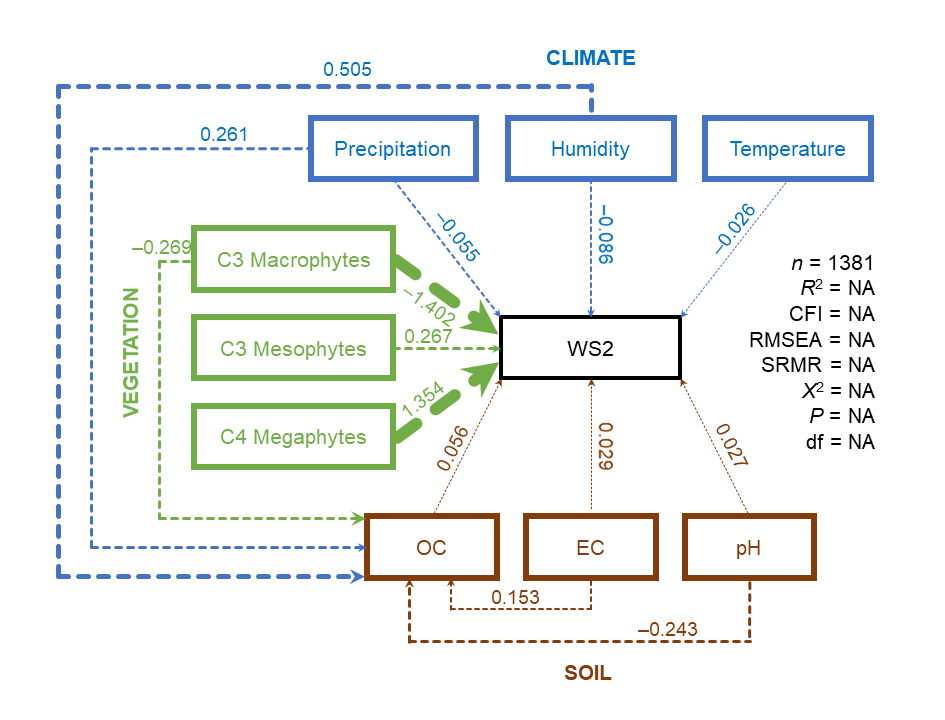

Supplement: Supplementary file 1 [file Data_Sheet_1.zip › Slide52.PNG]

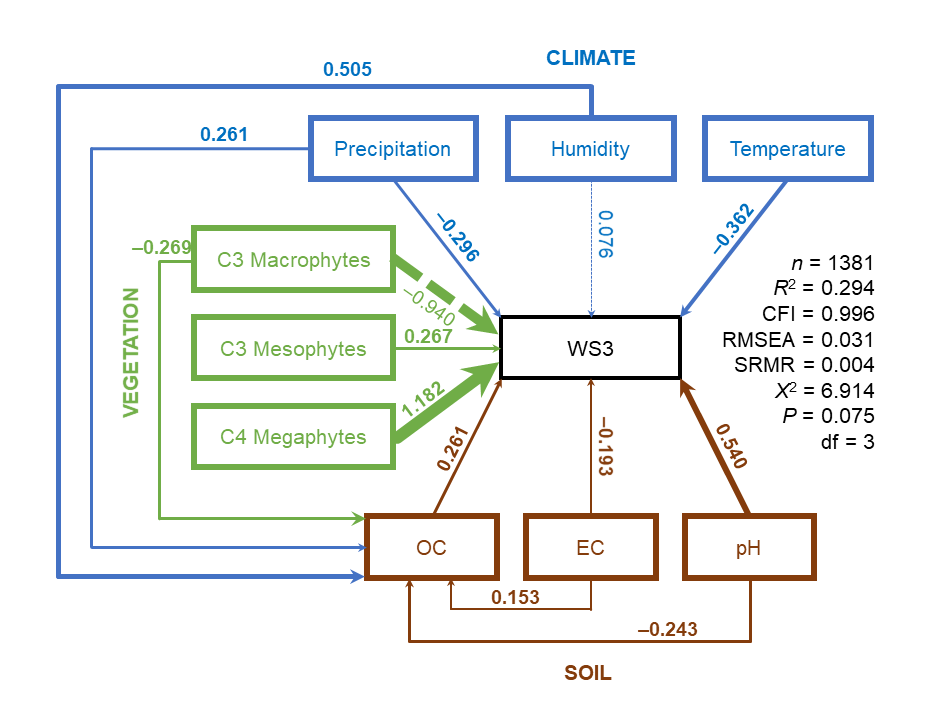

Supplement: Supplementary file 1 [file Data_Sheet_1.zip › Slide53.PNG]

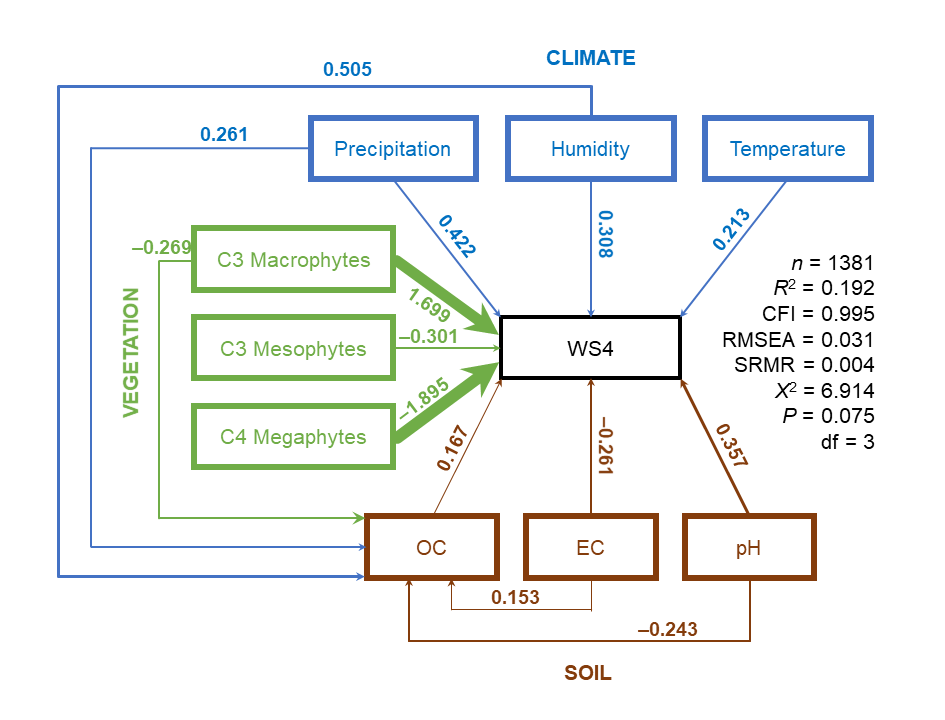

Supplement: Supplementary file 1 [file Data_Sheet_1.zip › Slide54.PNG]

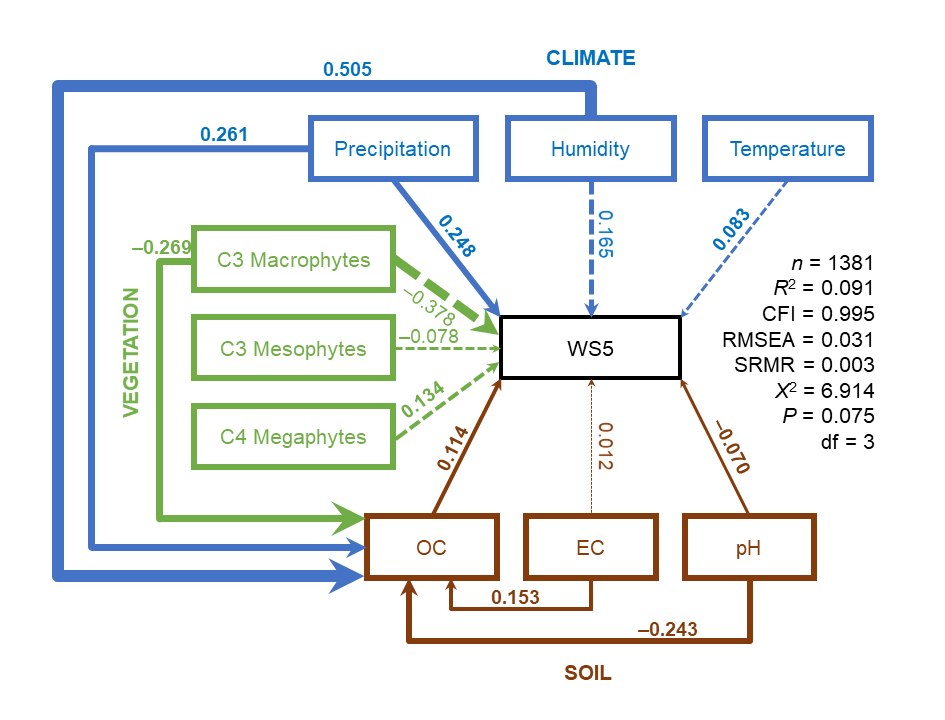

Supplement: Supplementary file 1 [file Data_Sheet_1.zip › Slide55.PNG]

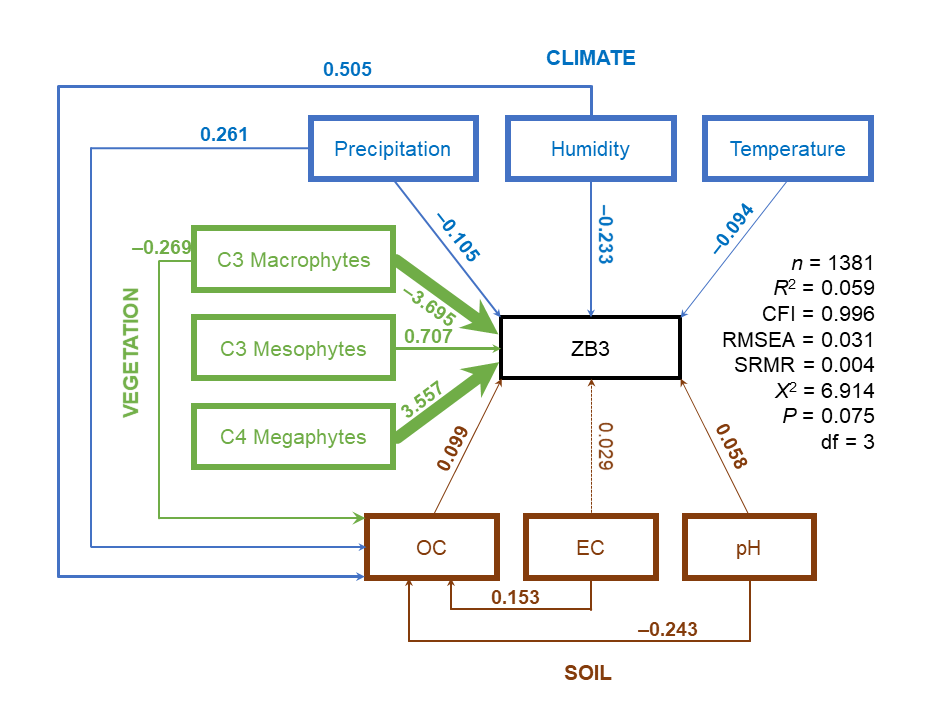

Supplement: Supplementary file 1 [file Data_Sheet_1.zip › Slide56.PNG]

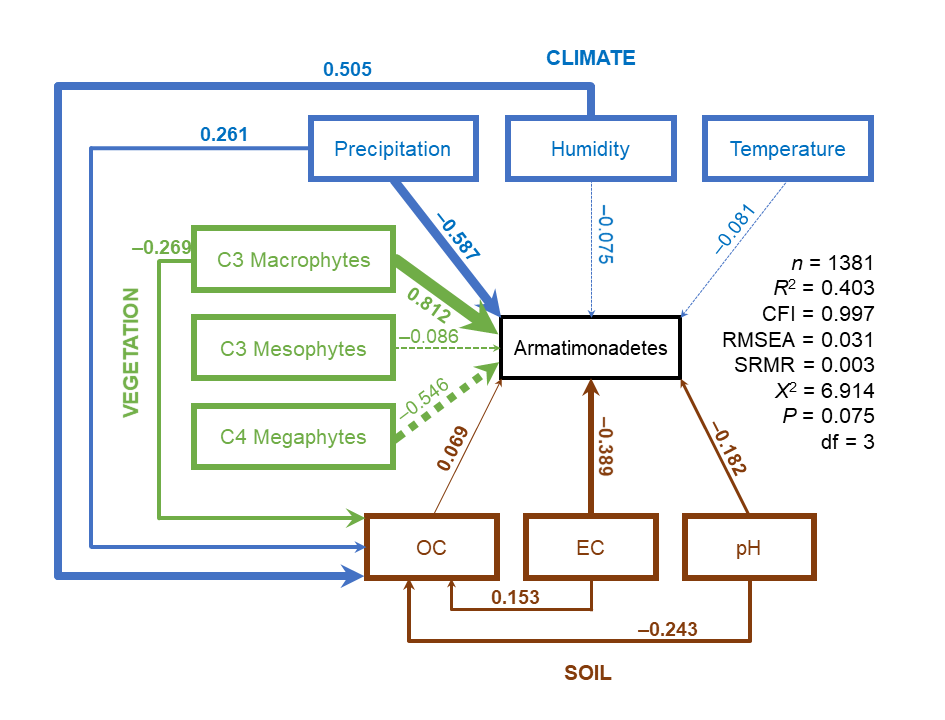

Supplement: Supplementary file 1 [file Data_Sheet_1.zip › Slide6.PNG]

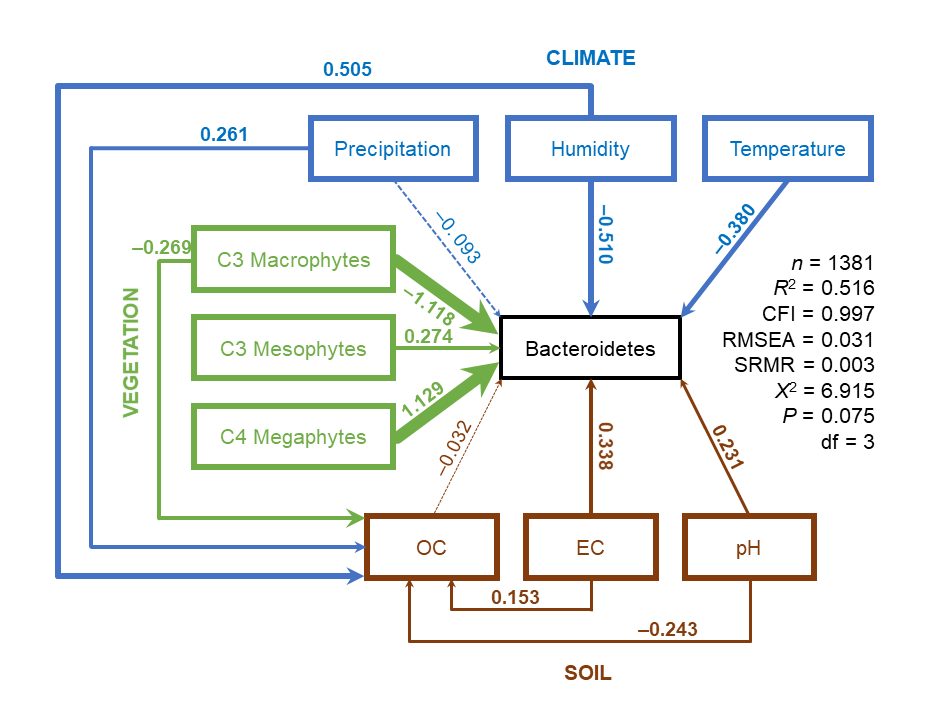

Supplement: Supplementary file 1 [file Data_Sheet_1.zip › Slide7.PNG]

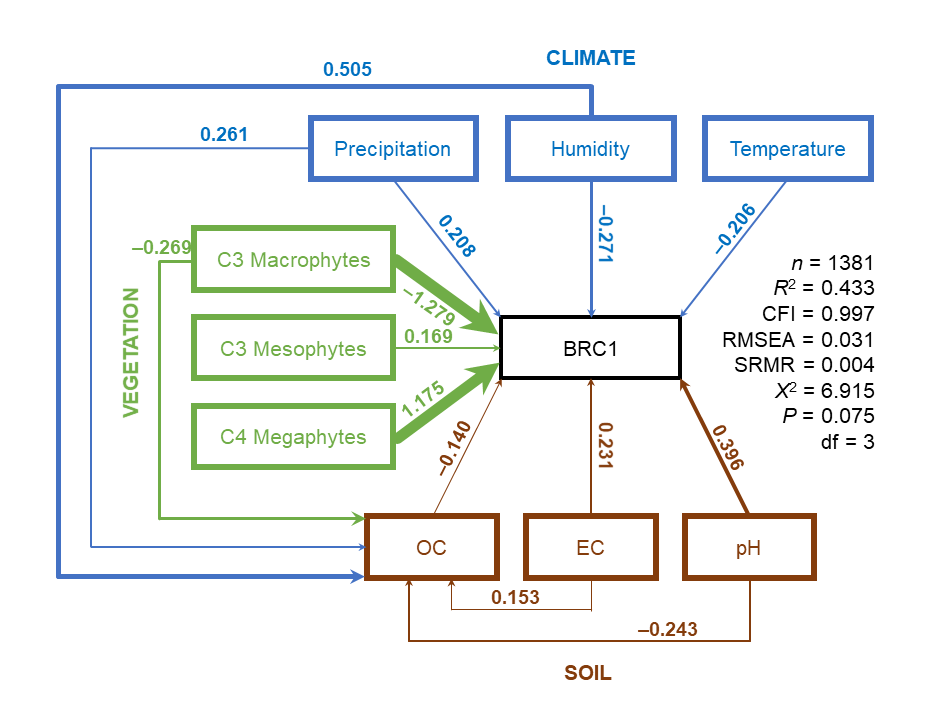

Supplement: Supplementary file 1 [file Data_Sheet_1.zip › Slide8.PNG]

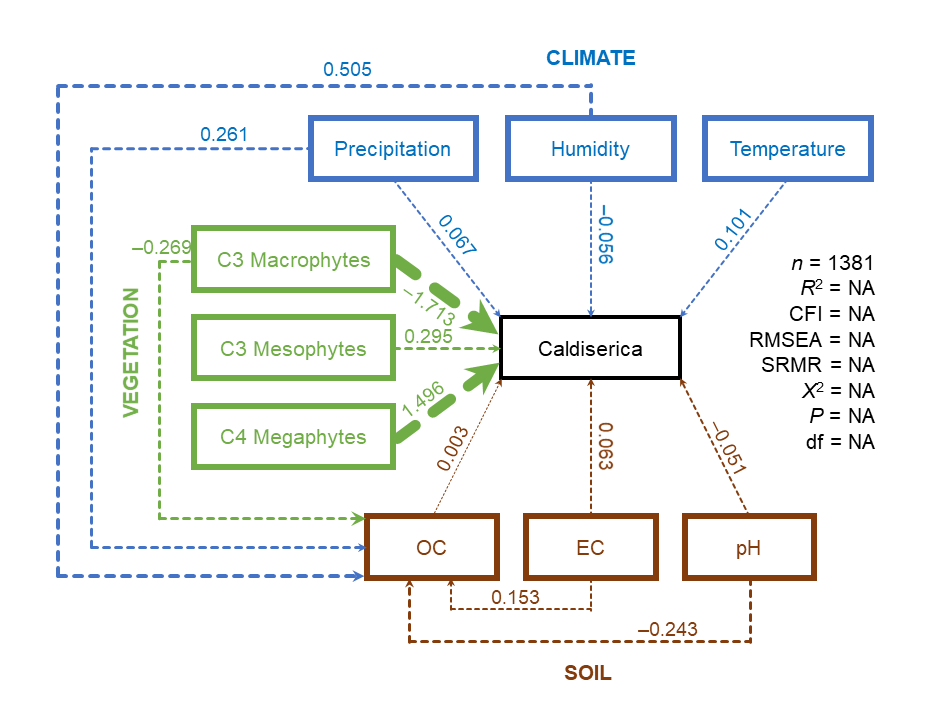

Supplement: Supplementary file 1 [file Data_Sheet_1.zip › Slide9.PNG]
